# Association between Blood Lipid Levels and Personality Traits in Young Korean Women

**DOI:** 10.1371/journal.pone.0108406

**Published:** 2014-09-30

**Authors:** Seung-Ju Roh, Han-Na Kim, Unjin Shim, Bo-Hye Kim, Su-Jin Kim, Hye Won Chung, Hyejin Lee, Yeon-Ah Sung, Hyung-Lae Kim

**Affiliations:** 1 Department of Biochemistry, School of Medicine, Ewha Womans University, Seoul, Korea; 2 Department of Internal Medicine, Seoul Seonam Hospital, Ewha Womans University Medical Center, Seoul, Korea; 3 Obstetrics and Gynecology, School of Medicine, Ewha Womans University, Seoul, Korea; 4 Internal Medicine, School of Medicine, Ewha Womans University, Seoul, Korea; Heart Research Institute, Australia

## Abstract

Abnormal lipid levels are important etiological factors associated with the development of atherosclerosis and with increased cardiovascular morbidity and mortality. Lipid levels are also influenced by lifestyle and behavioral factors, which suggests that personality traits might be related to abnormal lipid profiles. Studies on personality traits and lipid levels are relatively scarce in Korea. Therefore, the objective of this study was to examine the association between lipid levels and personality traits in young Korean women. A total of 1,701 young Korean women [mean age  = 24.9±4.6 years (range 17–39)] who volunteered for personality trait evaluation were recruited for this study. Lipid levels, including total cholesterol, high density lipoprotein (HDL) cholesterol, and triglyceride, were measured in all subjects after an overnight fast, and a low density lipoprotein (LDL) cholesterol level was calculated. The study population was divided into abnormal and normal lipid level groups according to the clinical criteria. Personality traits were measured using the Revised NEO Personality Inventory for the Five-Factor Model of personality. High neuroticism was associated with low HDL cholesterol levels. Low extraversion and openness were associated with high levels of triglyceride. At the facet level, the association between personality and lipid levels were generally consistent. Angry hostility, self-consciousness, vulnerability to stress, activity, and straightforwardness were associated with HDL cholesterol levels. Activity, positive emotion, aesthetics, actions, and deliberation were associated with triglyceride. When applying clinical criteria, conscientiousness was less likely to have abnormal total cholesterol levels. Our results showed that the women with the low HDL cholesterol levels are like to be more neurotic and the hyperglycemic women are prone to lower extraversion and openness in Korea. Understanding the associations between blood lipid levels and personality traits may have a beneficial effect for the managing of dyslipidemia.

## Introduction

Dyslipidemia is one of the major risk factors for metabolic syndrome and contributes to the progression of atherosclerosis and the development of cardiovascular diseases [1]. The prevalence of dyslipidemia has increased in younger people, especially in Korea [2], [3]. Atherosclerosis is a vascular disease caused by arterial wall inflammation that results in the accumulation of low density lipoprotein (LDL) cholesterol, monocytes, macrophages, and fat-laden foam cells at the location of the inflammation [4]. As plaques accumulate in the artery walls, the walls narrow and lose elasticity. This change leads to a decrease in the blood flow rate, and blocked coronary arteries will cause angina or even heart attack [5]. Dyslipidemia is an abnormally high amount of blood lipids and is characterized by elevated total cholesterol, LDL cholesterol, and triglyceride and decreased high density lipoprotein (HDL) cholesterol levels. Dyslipidemia is associated with lifestyle changes, such as changes in physical activity levels and in dietary intake patterns [6]–[8]. Increasing physical activity in overweight individuals reduces LDL and triglyceride levels, but increases HDL cholesterol levels. Healthy eating patterns (e.g., high fiber diet, cereal, and low fat dairy) are inversely associated with triglyceride levels [9]. A decrease in dietary fat intake also affects the size and density of LDL cholesterol and a decrease in dietary carbohydrate intake is associated with a reduction in hypertriglyceridemia [10].

Personality traits can influence lifestyle, cognition, motivation, and behaviors in a variety of situations [11]. Understanding personality traits may be important for maintaining health and beneficial behavior patterns. The Five-Factor Model (FFM) is often used for the assessment of personality traits [12]. The FFM is an empirically-derived model of personality that characterizes the individual's characteristic ways of thinking, feeling, and behaving along five broad dimensions [13]. Neuroticism is the tendency to experience negative emotions such as anxiety, anger, and depression. Extraversion is the tendency to be sociable, warm, active, assertive, cheerful, and to search for stimulation. Openness is the tendency to be imaginative, creative, unconventional, and emotionally and artistically sensitive. Agreeableness is the tendency to be altruistic, trusting, modest, and cooperative. Conscientiousness is the tendency to be strong-willed, persistent, reliable, and to comply with rules and ethical principles [12].

Lipid levels are influenced by lifestyle and behavioral factors that may be associated with personality traits, thus, being cross-linked with emotion and personality. Greater optimism is associated with high HDL cholesterol and lower triglyceride levels [14]. Lower HDL cholesterol and elevated triglyceride levels are associated with depression in women, but not in men [15]. High lipid levels in women are correlated with total score values for emotional state characteristics (i.e. tension, anxiety, depression, dejection, and confusion) [16]. Personality traits are important because they include emotion, thought, and behavior. Openness has a beneficial effect on lipid profiles and abdominal obesity [17]. Openness is positively correlated with favorable HDL cholesterol, triglyceride, and systolic blood pressure levels, and a smaller waist circumference [18]. Low conscientiousness is associated with lower HDL cholesterol and higher triglyceride levels [19]. Low HDL cholesterol and high triglyceride levels are also associated with high neuroticism, high extraversion, low conscientiousness, and low openness [20].

Studying the associations between blood lipid levels and personality traits may have important implications for future health outcomes, such as development of poor health and outbreaks of illness. However, most of the studies of personality traits and lipid levels have mainly been performed in Caucasian populations [19], and studies of Asian populations are relatively scarce. Some studies have found significant associations between metabolic syndrome and negative emotions, especially in women [21], [22]. Therefore, the objective of this study was to examine the relationships between personality traits and lipid profiles in young Korean women.

## Methods

### Participants

A total of 1,732 women who volunteered for a personality trait study that begin in 2008 at Ewha Womans University Mokdong Hospital (Seoul, Korea), were recruited for this study. After excluding data with missing values, and subjects with a history of medical treatment for psychiatric disorders or psychoactive drugs, a total of 1,701 women were evaluated (mean age  = 24.9±4.6 years (range 17–39); mean body mass index (BMI)  = 21.65±3.40 kg/m^2^).

### Statement of ethics

The Institutional Review Board of Ewha Womans University Mokdong Hospital approved this study. Written informed consent was obtained from all participants. For participants who were minors, written informed consent was obtained from the parents or caretakers. All applicable institutional and governmental regulations concerning the ethical use of human volunteers were followed during this research.

### Blood lipid levels

Lipid profiles that included total cholesterol, HDL cholesterol, and triglyceride levels were obtained using standard enzymatic methods. LDL cholesterol was calculated using the Friedewald equation [23]: LDL cholesterol  =  Total cholesterol – HDL cholesterol – (triglyceride/5). Abnormal lipid profiles were defined using the clinical criteria published in American Heart Association and National Cholesterol Education Program reports [1], [3], [24]. The cutoffs for abnormal lipid profiles were: total cholesterol ≥200 mg/dL, HDL cholesterol <40 mg/dL, LDL cholesterol ≥130 mg/dL, and triglyceride >150 mg/dL.

### Measurement

Personality traits were assessed using the Korean short version of the Revised NEO personality Inventory (NEO-PI-R, PSI Consulting Corp., Seoul, Korea), which is a 90-item questionnaire designed to operationalize the FFM. NEO-PI-R has a robust factor structure that has been replicated in Korea [25] and more than 50 cultures [26]. The Korean version of the NEO PI-R has been used in Korean population and demonstrated good reliability and validity [25]. The questionnaire consisted of 18 items per factor (i.e., neuroticism, extraversion, openness, agreeableness, and conscientiousness). Each factor consisted of six facets. Neuroticism consisted of anxiety (N1), angry hostility (N2), depression (N3), self-consciousness (N4), impulsiveness (N5), and vulnerability (N6). The facets for extraversion were warmth (E1), gregariousness (E2), assertiveness (E3), activity (E4), excitement-seeking (E5), and positive emotions (E6). Openness consisted of fantasy (O1), aesthetics (O2), feelings (O3), actions (O4), ideas (O5), and values (O6). Agreeableness consisted of the facets trust (A1), straightforwardness (A2), altruism (A3), compliance (A4), modesty (A5), and tender-mindedness (A6). Conscientiousness consisted of the facets competence (C1), order (C2), dutifulness (C3), achievement striving (C4), self-discipline (C5), and deliberation (C6) [12]. A 5-point Likert scale, ranging from “strongly disagree” to “strongly agree”, was used for the responses. In the present study, the Cronbach's alpha coefficients of neuroticism, extraversion, openness, agreeableness, and conscientiousness were 0.85, 0.86, 0.76, 0.69, and 0.78, respectively.

Smoking and drinking status were collected using a questionnaire. For smoking status, participants were asked to mark one of the three categories: ‘never smokers’, ‘former smokers’, or ‘current smokers’. We classified ‘never smokers’ as those who never smoked (n = 1527, 89.7%), ‘former smoker’ as those who have quit smoking (n = 84, 4.9%), ‘current smokers’ were those who reported smoking at the time of the most recent visit (90, 5.3%). Drinking status was also classified as ‘never drinking’ (n = 280, 16.5%), ‘former drinking’ (n = 177, 10.4%), and ‘current drinking’ (n = 1244, 73.1%).

### Statistical analysis

All statistical analyses were conducted using SAS version 9.3 for Windows (SAS Institute Inc., USA). Associations between blood lipid levels and personality traits were analyzed after controlling for age, BMI, systolic blood pressure, smoking status, and drinking status. We set dummy variables for smoking and drinking status because they are categorical variables. “Never smokers” and “current drinking” were set as the reference group, respectively. Linear regression analysis was used to evaluate the associations between blood lipid levels and the FFM, and the same analysis was used for each personality facet. The t-test was used to compare the mean of personality traits between the clinical abnormal and normal lipid groups. Personality traits predicting abnormal lipid levels were examined using logistic regression analysis after adjusting for potential confounders including age, BMI, systolic blood pressure, smoking status, and drinking status. This study was carried out on recommendation of Simmons et al. [27].

## Results

The mean values ± standard deviation of the mean (SD) for concentrations of total cholesterol, HDL cholesterol, LDL cholesterol, and triglyceride were 176.57±28.04 mg/dL, 50.72±11.61 mg/dL, 110.19±24.42 mg/dL, and 78.32±39.69 mg/dL, respectively (Table 1). The numbers present in each clinical category for lipid levels were: 315 (18.5%) women had high total cholesterol levels, 290 (17.0%) women had low HDL cholesterol levels, 317 (18.6%) women had high LDL cholesterol and 88 (5.1%) women had high triglyceride levels. The mean ± SD values for the personality traits neuroticism, extraversion, openness, agreeableness, and conscientiousness were 57.56±9.79, 60.59±9.55, 64.03±7.89, 59.62±7.53, and 60.91±8.31, respectively.

**Table 1:** Descriptive statistics results for study population demographics, blood lipid levels, and personality variables.

Variable		Sample characteristics
		Mean ± SD
**Demographics**		
	**Age**	24.93±4.55
	**Sex (female)**	100%
	**BMI**	21.65±3.40
		
	**Lipid levels**	
	**Total cholesterol (mg/dL)**	176.57±28.04
	**HDL cholesterol (mg/dL)**	50.72±11.61
	**Derived LDL cholesterol (mg/dL)**	110.19±24.42
	**Triglyceride (mg/dL)**	78.32±39.69
**Personality traits**		
	**Neuroticism**	57.56±9.79
	**Extraversion**	60.59±9.55
	**Openness**	64.03±7.89
	**Agreeableness**	59.62±7.53
	**Conscientiousness**	60.91±8.31

Note. N = 1701.

SD: Standard deviations.

The linear regression analysis used to examine the associations between personality traits and total cholesterol, HDL cholesterol, LDL cholesterol, and triglyceride levels. The factor and facet level of personality respectively were used as independent variables. High neuroticism was associated with low HDL cholesterol levels (*β* = −0.053, *p*<0.05, adjusted *R^2^* = 0.11) (Table 2). Low extraversion and low openness were associated with high triglyceride levels [*β* = −0.046, *p*<0.05, adjusted *R^2^* = 0.16 (extraversion), *β* = −0.059, *p*<0.01, adjusted *R^2^* = 0.16 (openness)]. Agreeableness and conscientiousness were not significantly association with lipid levels. At the facet level, three facets of neuroticism were associated negatively with HDL cholesterol [*β* = −0.051, *p*<0.05 (angry hostility, N2); *β* = −0.052, *p*<0.05 (self-consciousness, N4); *β* = −0.064, *p*<0.01 (vulnerability to stress, N6)]. Activity (E4) and straightforwardness (A2) were associated positively with HDL cholesterol levels (*β* = 0.057, *p*<0.05; *β* = 0.052, *p*<0.05, respectively). Low scores for the extraversion facets of activity and positive emotion were with high triglyceride level [*β* = −0.050, *p*<0.05 (activity); *β* = −0.054, *p*<0.05 (positive emotion)]. Low scores for the openness facets of aesthetics and actions were associated with high triglyceride levels [*β* = −0.050, *p*<0.05 (aesthetics); *β* = −0.084, *p*<0.01 (actions)]. The associations between facet levels of personality traits and lipid levels were generally consistent comparing with those of factor levels. The deliberation, facet of conscientiousness, was associated with triglyceride (*β* = 0.048, *p*<0.05) although conscientiousness of factor level was not shown significant correlation with that.

**Table 2:** Results of the associations between personality traits and blood lipid levels.

	Cholesterol (mg/dL)						Triglyceride (mg/dL)	
Personality traits	Total Cholesterol		HDL		Derived LDL			
	*β* a	*P*	*β* a	*P*	*β* a	*P*	*β* a	*P*
**Factor-levels**								
**Neuroticism**	−0.023	0.324	−0.053	**0.024** *	0.002	0.920	−0.009	0.696
**Extraversion**	−0.007	0.758	0.034	0.139	−0.011	0.643	−0.046	**0.040** *
**Openness**	−0.024	0.311	0.030	0.189	−0.022	0.348	−0.059	**0.009** **
**Agreeableness**	0.010	0.663	0.040	0.081	−0.009	0.705	0.003	0.891
**Conscientiousness**	−0.002	0.925	0.018	0.425	−0.020	0.405	0.022	0.321
**Facet-levels**								
**Angry Hostility (N2)**	−0.037	0.115	−0.051	**0.027** *	−0.017	0.480	−0.004	0.843
**Self-consciousness (N4)**	−0.030	0.208	−0.052	**0.024** *	−0.008	0.726	0.000	0.986
**Vulnerability to Stress (N6)**	−0.037	0.124	−0.064	**0.005** **	−0.007	0.764	−0.011	0.618
**Activity (E4)**	0.009	0.695	0.057	**0.012** *	−0.001	0.969	−0.050	**0.027** *
**Positive Emotion (E6)**	−0.006	0.790	0.043	0.063	−0.011	0.635	−0.054	**0.017** *
**Aesthetics (O2)**	−0.031	0.190	0.017	0.456	−0.026	0.258	−0.050	**0.024** *
**Actions (O4)**	−0.027	0.250	0.040	0.080	−0.025	0.290	−0.084	**0.000** **
**Straightforwardness (A2)**	0.003	0.897	0.052	**0.028** *	−0.018	0.456	−0.012	0.615
**Deliberation (C6)**	−0.017	0.486	−0.004	0.852	−0.032	0.167	0.048	**0.031** *

Note. N = 1701.

a Standardized regression coefficients adjusted with age, BMI, systolic blood pressure, current smoking status, and drinking status.

*P<0.05, **P<0.01. Significant results are bolded.

When applying clinical criteria for the lipid level, we found significant differences between the normal and abnormal groups for the factor-level conscientiousness and neuroticism (t-test *p* = 0.027, *p* = 0.038, respectively, Table S1). As noted earlier, a logistic regression analysis was used to examine whether personality traits could predict individuals falling in the at-risk for heart disease category for abnormal lipid levels, as has been identified by American Heart Association. After adjusting for potential confounders, individuals with high conscientiousness were less likely to have abnormal total cholesterol levels [odds ratio (OR)  = 0.982, 95% confidence interval (CI)  = 0.968–0.997] (Table 3). At the facet level of personality, individuals with high levels of dutifulness (C3) were less likely to have at-risk levels of total cholesterol levels (OR  = 0.918, 95% CI  = 0.858–0.981). Individual who ranked high in straightforwardness (A2) were more likely to have HDL levels above the protective threshold (OR  = 0.936, 95% CI  = 0.883–0.992). A study has reported that individual with higher straightforwardness were leaner [28].

**Table 3:** Results for the logistic regression analyses examining the associations between blood lipid level categories and personality traits.

	Cholesterol (mg/dL)						Triglyceride (mg/dL)^d^	
Personality traits	Total Cholesterol^a^		HDL^b^		Derived LDL^c^			
	OR	95%CI	OR	95%CI	OR	95%CI	OR	95%CI
**Factor-levels**								
**Neuroticism**	0.996	0.984–1.009	1.009	0.995–1.023	1.001	0.988–1.014	1.012	0.988–1.036
**Extraversion**	0.997	0.984–1.010	0.995	0.981–1.009	0.994	0.981–1.007	0.988	0.964–1.011
**Openness**	0.995	0.980–1.011	0.990	0.973–1.007	0.996	0.980–1.012	0.977	0.950–1.005
**Agreeableness**	0.990	0.974–1.007	0.986	0.968–1.004	0.993	0.976–1.010	0.996	0.966–1.027
**Conscientiousness**	0.982	**0.968–0.997** *	0.994	0.978–1.010	0.990	0.975–1.005	0.988	0.961–1.015
**Facet-levels**								
**Straightforwardness (A2)**	0.984	0.932–1.038	0.936	**0.883–0.992** *	0.977	0.925–1.032	0.918	0.830–1.015
**Dutifulness (C3)**	0.918	**0.858–0.981** *	1.018	0.948–1.094	0.948	0.886–1.015	0.917	0.813–1.034

Note. OR, odds ratio: CI, confidence interval.

Logistic regression analyses were performed after adjusted for age, BMI, systolic blood pressure, current smoking status, and drinking status.

a Normal N = 1386, Abnormal N = 315.

b Normal N = 1410, Abnormal N = 291.

c Normal N = 1384, Abnormal N = 317.

d Normal N = 1614, Abnormal N = 87.

*P<0.05. Significant results are bolded.

## Discussion

The results of our study indicated that high neuroticism was significantly and associated with low HDL cholesterol levels in young Korean women. There was also a significant negative association between extraversion and openness, and triglyceride levels. When clinical criteria were applied, individuals with lower conscientiousness showed abnormal lipid level. We analyzed the associations between personality traits and lipid levels and then performed a further analysis using the clinical lipid level categories. Because the population was comprised of young women, this 2-step analysis was meaningful and could be used to aid in the prediction and prevention of disease progression.

The facets of higher neuroticism include angry hostility, self-consciousness, and vulnerability to stress, which were associated with low HDL cholesterol levels. This finding is consistent with a previous study that found that higher neuroticism including sub facets was associated with lower levels of HDL cholesterol in women [20], [29]. Psychological traits such as negative emotions are also associated with low HDL cholesterol profiles [15]. High hostility level is related to increased risk of angiographically documented coronary atherosclerosis [30], essential hypertension [31], coronary artery disease incidence, and all-cause mortality [32], [33]. Neurotic individuals tend to be more anxious and impulsive, so they are more vulnerable to the negative effects of mental stress. Hostile individuals are also more likely to exhibit alcohol related behaviors [34]. This tendency could lead to overeating and excessive drinking, and consequently, dyslipidemia [35], [36]. In this study, adjustment for BMI and drinking status partially explained association between the facets of neuroticism and HDL cholesterol levels; suggesting that health behaviors are particularly important in lipid levels in individuals with high levels of neuroticism. These behavioral patterns can contribute to weight gain, which affects lifestyle and leads to cardiovascular and other diseases. Our findings provide a possible explanation for the association between neuroticism and coronary heart disease.

Low extraversion and openness were associated with high triglyceride levels. At the facet level, activity (E4), positive emotion (E6), aesthetics (O2), action (O4), and deliberation (C6) were associated with high triglyceride levels. Previous studies focusing on these two personality traits and triglyceride levels, have yielded conflicting results [19], [20]. Our findings for extraversion were consistent with the results of Sutin et al. [19] and them for openness were consistent with the results of Armon [20]. Individuals with higher extraversion and openness participate in more physical activity and have greater mental flexibility [37]. Individual with high scores for extraversion [38] considered themselves to be healthy. These characteristics may have positive implications for physical health. Increased physical activity occurs in individuals with higher extraversion and higher openness [39], and the elevated energy expenditure could reduce triglyceride levels. Reduced concentrations of triglyceride in people who regularly participate in physical activities may decrease the risk of metabolic syndrome. In a study that compared the dietary patterns of subjects divided into four groups (i.e., “Mediterranean style,” “health aware,” “convenience,” and “sweet foods” diets), low openness was associated with the consumption of a convenience diet and of sweet foods [40]. High openness is associated with healthy dietary habits [41]. These dietary habits was correlated with lower total cholesterol, higher HDL cholesterol, and lower triglyceride levels [42]. Individuals with high openness are more likely effectively overcome stress. Lower openness may increase the susceptibility to stress, and lead to increased food intake and development of obesity [43]. Dietary habits and physical activity are closely related to lipid levels, and abnormalities can lead to the development of metabolic syndrome. Extraversion and openness could be a beneficial personality trait resulting in decreased triglyceride levels, and a reduced risk of metabolic syndrome.

We found a significant association between high straightforwardness (A2) and high HDL cholesterol levels although no association were found between agreeableness and the serum lipid profile in previous studies [19], [20]. Lower HDL cholesterol levels is associated with increasing body mass index [44]. The findings for the association between agreeableness and obesity are inconsistent. Sutin et al. found low agreeableness predicted a greater increase in BMI [17], [28], [45], but Chapman et al. reported that higher agreeableness has been associated with higher BMI [46]. Agreeableness acts as a ‘self-regulatory’ trait that controls and regulated highly aroused negative emotions and therefore might protect against unhealthy lipid profiles [20].

Although negative association between conscientiousness and triglyceride has reported in other study [19], we found that deliberation (C6) was associated positively with triglyceride. This association, however, did not hold when applying clinical criteria. In the current study, conscientiousness was a significant predictor at clinical risk threshold for total cholesterol. Individuals with low conscientiousness tend to be less concerned about their health and exhibit poor self-control and unhealthy behaviors that lead to the development of obesity, diabetes, and metabolic syndrome [47]–[49]. On the contrary, conscientious individuals value health management and they are more likely to expend effort to lead a healthy life [50]. Dutiful individuals tend to adhere to their principles [12], including maintaining a healthy lifestyle, which may also help keep their total cholesterol level normal. Conscientiousness is associated with longevity and low mortality risk [51]. Elevated total cholesterol contributes to coronary atherosclerosis throughout life [52]. Our results suggest that the total cholesterol level may be partial mediator to the association of conscientiousness and coronary heart disease.

Similar to the results of Armon [20], LDL cholesterol levels was not associated with any of the personality factors in our study. LDL cholesterol is independent risk factor for cardiovascular morbidity and mortality and they are less susceptible to lifestyle habits compared with HDL cholesterol and triglyceride [20]. Our results support the suggestions of Armon [20] that LDL cholesterol levels are less affected by health behaviors, so they are less likely to be linked to personality factors.

This study is the first to evaluate the association between personality traits and serum lipid levels in young Korean women using the high quality measure of major dimensions of personality plus detailed assessment of specific facets. There is little research on the personality and health in Asia. Our results showed that the women with the low HDL cholesterol levels are like to be more neurotic and the hypertriglycemic women are prone to lower extraversion and openness in Korea. It would be significant in a sense that the knowing the individual's personality trait can be used a kind of intervention in caring the progress of dyslipidemia. Despite this strength, there are some limitations in our study. We cannot control for the confounding influence of time-invariant common causes [53] and infer causality of the personality traits because this study is based on a cross-sectioned data. Additionally, this study was confined to women, so we need further study for men to generalize the findings. Factors or facets of the personality significant in a linear regression analysis were not significant when they are performed in the logistic regression analyses. That could arise from in part the relatively small sample size of dyslipidemia. The use of the short version of the NEO-PI-R may have also imposed limitation on the interpretation of contribution of individual facet. However, when considering that the low proportion of dyslipidemia is attributed to the young and healthy woman, we would like to put meaning that there is the association between the dyslipidemia and the personality traits in healthy population.

In summary, personality traits were linked with serum lipid levels in Korean. Conscientiousness was significantly associated with abnormal total cholesterol level. Understanding personality traits will improve the understanding of the associations between psychological factors and abnormal lipid levels, and may contribute to future managing of dyslipidemia.

## Supporting Information

Table S1 **Comparison of the clinical characteristics of abnormal and normal lipid level groups.**
(DOC)

Data S1 **The data underlying the findings in this study.** The sheet 1 includes all data and the sheet 2 includes code of them.
(XLS)

Linear Regression S1 **The results of the linear regression using SAS program.** TC: Total Cholesterol, TG: Triglyceride, N: Neuroticism, E: extraversion, O: Openness, A: Agreeableness, C: Conscientiousness, BMI: Body Mass Index, SBP: Systolic pressure, D1_al: Dummy1 for alcohol, D2_al: Dummy2 for alcohol, D1_sm: Dummy1 for smoking, and D2_sm: Dummy2 for smoking.
(PDF)

Logistic Regression S1 **The results of the logistic regression using SAS program.** Response variables are TC_x (total cholesterol), TG_x (triglyceride), HDL_x, and LDL_x applying clinical criteria. The group “0” and “1” represents normal and abnormal groups, respectively.
(PDF)

T-Test Result S1 **The results of the t-test using SAS program.** There are significant differences between the normal (“0”) and abnormal groups (“1”) when applying clinical criteria for the lipid level.
(PDF)

## References

[1] Expert Panel on Detection E, Treatment of High Blood Cholesterol in A (2001) Executive Summary of The Third Report of The National Cholesterol Education Program (NCEP) Expert Panel on Detection, Evaluation, And Treatment of High Blood Cholesterol In Adults (Adult Treatment Panel III). JAMA: the journal of the American Medical Association 285: 2486–2497. 11368702 10.1001/jama.285.19.2486

[2] Lee MH, Kim HC, Ahn SV, Hur NW, Choi DP, et al (2012) Prevalence of Dyslipidemia among Korean Adults: Korea National Health and Nutrition Survey 1998–2005. Diabetes & metabolism journal 36: 43–55. 22363921 10.4093/dmj.2012.36.1.43 PMC3283826

[3] Yang S, Hwang JS, Park HK, Lee HS, Kim HS, et al (2012) Serum Lipid Concentrations, Prevalence of Dyslipidemia, and Percentage Eligible for Pharmacological Treatment of Korean Children and Adolescents; Data from the Korea National Health and Nutrition Examination Survey IV (2007–2009). PloS one 7: e49253. 23251338 10.1371/journal.pone.0049253 PMC3522657

[4] Cilla M, Pena E, Martinez MA (2014) Mathematical modelling of atheroma plaque formation and development in coronary arteries. Journal of the Royal Society, Interface/the Royal Society 11: 20130866. 10.1098/rsif.2013.0866 PMC3836327 24196695

[5] Xie X, Wang Y, Zhu H, Zhou H, Zhou J (2013) Impact of coronary tortuosity on coronary blood supply: a patient-specific study. PloS one 8: e64564. 23691249 10.1371/journal.pone.0064564 PMC3656900

[6] Delavar M, Lye M, Hassan S, Khor G, Hanachi P (2011) Physical activity, nutrition, and dyslipidemia in middle-aged women. Iranian journal of public health 40: 89–98. PMC3481733 23113107

[7] Montesi L, Moscatiello S, Malavolti M, Marzocchi R, Marchesini G (2013) Physical activity for the prevention and treatment of metabolic disorders. Internal and emergency medicine 10.1007/s11739-013-0953-7. 10.1007/s11739-013-0953-7 23657989

[8] Gupta N, Shah P, Nayyar S, Misra A (2013) Childhood obesity and the metabolic syndrome in developing countries. Indian journal of pediatrics 80 Suppl 1: S28–37. 23334584 10.1007/s12098-012-0923-5

[9] Newby PK, Muller D, Tucker KL (2004) Associations of empirically derived eating patterns with plasma lipid biomarkers: a comparison of factor and cluster analysis methods. The American journal of clinical nutrition 80: 759–767. 15321819 10.1093/ajcn/80.3.759

[10] Parks EJ, Hellerstein MK (2000) Carbohydrate-induced hypertriacylglycerolemia: historical perspective and review of biological mechanisms. The American journal of clinical nutrition 71: 412–433. 10648253 10.1093/ajcn/71.2.412

[11] Hoyt AL, Rhodes RE, Hausenblas HA, Giacobbi PR (2009) Integrating five-factor model facet-level traits with the theory of planned behavior and exercise. Psychol Sport Exerc 10: 565–572.

[12] Costa PT, McCrae RR, Psychological Assessment Resources I (1992) Revised NEO Personality Inventory (NEO PI-R) and NEO Five-Factor Inventory (NEO FFI): Professional Manual: Psychological Assesment Resources.

[13] McCrae RR, Costa PT (2003) Personality in Adulthood: A Five-Factor Theory Perspective: Guilford Press.

[14] Boehm JK, Williams DR, Rimm EB, Ryff C, Kubzansky LD (2013) Relation between optimism and lipids in midlife. The American journal of cardiology 111: 1425–1431. 23433765 10.1016/j.amjcard.2013.01.292 PMC3644345

[15] Kinder LS, Carnethon MR, Palaniappan LP, King AC, Fortmann SP (2004) Depression and the metabolic syndrome in young adults: Findings from the third national health and nutrition examination survey. Psychosomatic medicine 66: 316–322. 15184689 10.1097/01.psy.0000124755.91880.f4

[16] Lasaite L, Lasiene J, Kazanavicius G, Gostautas A (2009) [Associations of emotional state and quality of life with lipid concentration, duration of the disease, and the way of treating the disease in persons with type 2 diabetes mellitus]. Medicina 45: 85–94. 19289898

[17] Terracciano A, Sutin AR, McCrae RR, Deiana B, Ferrucci L, et al (2009) Facets of personality linked to underweight and overweight. Psychosomatic medicine 71: 682–689. 19414622 10.1097/PSY.0b013e3181a2925b PMC2711222

[18] Dortland AKBV, Giltay EJ, van Veen T, Zitman FG, Penninx BWJH (2012) Personality traits and childhood trauma as correlates of metabolic risk factors: The Netherlands Study of Depression and Anxiety (NESDA). Prog Neuro-Psychoph 36: 85–91. 10.1016/j.pnpbp.2011.10.001 22001949

[19] Sutin AR, Terracciano A, Deiana B, Uda M, Schlessinger D, et al (2010) Cholesterol, triglycerides, and the Five-Factor Model of personality. Biological psychology 84: 186–191. 20109519 10.1016/j.biopsycho.2010.01.012 PMC2933037

[20] Armon G (2013) Personality and Serum Lipids: Does Lifestyle Account for Their Concurrent and Long-term Relationships. European Journal of Personality 10.1002/per.1943: n/a-n/a.

[21] Elovainio M, Merjonen P, Pulkki-Raback L, Kivimaki M, Jokela M, et al (2011) Hostility, metabolic syndrome, inflammation and cardiac control in young adults: The Young Finns Study. Biological psychology 87: 234–240. 21419189 10.1016/j.biopsycho.2011.03.002

[22] Raikkonen K, Matthews KA, Sutton-Tyrrell K, Kuller LH (2004) Trait anger and the metabolic syndrome predict progression of carotid atherosclerosis in healthy middle-aged women. Psychosomatic medicine 66: 903–908. 15564356 10.1097/01.psy.0000143638.31297.11

[23] Friedewald WT, Levy RI, Fredrickson DS (1972) Estimation of the concentration of low-density lipoprotein cholesterol in plasma, without use of the preparative ultracentrifuge. Clinical chemistry 18: 499–502. 4337382

[24] Smith SC Jr, Blair SN, Bonow RO, Brass LM, Cerqueira MD, et al (2001) AHA/ACC Guidelines for Preventing Heart Attack and Death in Patients With Atherosclerotic Cardiovascular Disease: 2001 update. A statement for healthcare professionals from the American Heart Association and the American College of Cardiology. Journal of the American College of Cardiology 38: 1581–1583. 11691544 10.1016/s0735-1097(01)01682-5

[25] Ahn CK, Chae JH (1997) Standardization of the Korean version of the Revised NEO Personality Inventory. Korean Journal of Counseling & Psychotherapy 9: 443–473.

[26] McCrae RR, Terracciano A (2005) Universal features of personality traits from the observer's perspective: data from 50 cultures. Journal of personality and social psychology 88: 547–561. 15740445 10.1037/0022-3514.88.3.547

[27] Simmons JP, Nelson LD, Simonsohn U (2011) False-positive psychology: undisclosed flexibility in data collection and analysis allows presenting anything as significant. Psychological science 22: 1359–1366. 22006061 10.1177/0956797611417632

[28] Sutin AR, Ferrucci L, Zonderman AB, Terracciano A (2011) Personality and obesity across the adult life span. Journal of personality and social psychology 101: 579–592. 21744974 10.1037/a0024286 PMC3462003

[29] Richards JC, Hof A, Alvarenga M (2000) Serum lipids and their relationships with hostility and angry affect and behaviors in men. Health Psychology 19: 393–398. 10907658 10.1037//0278-6133.19.4.393

[30] Dembroski TM, MacDougall JM, Williams RB, Haney TL, Blumenthal JA (1985) Components of Type A, hostility, and anger-in: relationship to angiographic findings. Psychosomatic medicine 47: 219–233. 4001281 10.1097/00006842-198505000-00001

[31] Diamond EL (1982) The role of anger and hostility in essential hypertension and coronary heart disease. Psychol Bull 92: 410–433. 7146235

[32] Shekelle RB, Gale M, Ostfeld AM, Paul O (1983) Hostility, risk of coronary heart disease, and mortality. Psychosomatic medicine 45: 109–114. 6867229 10.1097/00006842-198305000-00003

[33] Barefoot JC, Dahlstrom WG, Williams RB Jr (1983) Hostility, CHD incidence, and total mortality: a 25-year follow-up study of 255 physicians. Psychosomatic medicine 45: 59–63. 6844529 10.1097/00006842-198303000-00008

[34] Nesic J, Duka T (2008) Effects of stress on emotional reactivity in hostile heavy social drinkers following dietary tryptophan enhancement. Alcohol and alcoholism 43: 151–162. 18218724 10.1093/alcalc/agm179

[35] Provencher V, Begin C, Gagnon-Girouard MP, Tremblay A, Boivin S, et al (2008) Personality traits in overweight and obese women: associations with BMI and eating behaviors. Eating behaviors 9: 294–302. 18549988 10.1016/j.eatbeh.2007.10.004

[36] Vainik U, Dagher A, Dube L, Fellows LK (2013) Neurobehavioural correlates of body mass index and eating behaviours in adults: a systematic review. Neuroscience and biobehavioral reviews 37: 279–299. 23261403 10.1016/j.neubiorev.2012.11.008 PMC4017079

[37] Courneya KS, Friedenreich CM, Sela RA, Quinney HA, Rhodes RE (2002) Correlates of adherence and contamination in a randomized controlled trial of exercise in cancer survivors: an application of the theory of planned behavior and the five factor model of personality. Ann Behav Med 24: 257–268. 12434937 10.1207/S15324796ABM2404_02

[38] Kikuchi Y, Watanabe S (2000) Personality and dietary habits. Journal of epidemiology/Japan Epidemiological Association 10: 191–198. 10.2188/jea.10.191 10860305

[39] Rhodes RE, Smith NE (2006) Personality correlates of physical activity: a review and meta-analysis. British journal of sports medicine 40: 958–965. 17124108 10.1136/bjsm.2006.028860 PMC2577457

[40] Mottus R, McNeill G, Jia X, Craig LC, Starr JM, et al (2013) The associations between personality, diet and body mass index in older people. Health psychology: official journal of the Division of Health Psychology, American Psychological Association 32: 353–360. 10.1037/a0025537 21928903

[41] Brummett BH, Siegler IC, Day RS, Costa PT (2008) Personality as a predictor of dietary quality in spouses during midlife. Behav Med 34: 5–10. 18400684 10.3200/BMED.34.1.5-10 PMC2391304

[42] Turley ML, Skeaff CM, Mann JI, Cox B (1998) The effect of a low-fat, high-carbohydrate diet on serum high density lipoprotein cholesterol and triglyceride. European journal of clinical nutrition 52: 728–732. 9805219 10.1038/sj.ejcn.1600634

[43] Torres SJ, Nowson CA (2007) Relationship between stress, eating behavior, and obesity. Nutrition 23: 887–894. 17869482 10.1016/j.nut.2007.08.008

[44] Pietrobelli A, Lee RC, Capristo E, Deckelbaum RJ, Heymsfield SB (1999) An independent, inverse association of high-density-lipoprotein-cholesterol concentration with nonadipose body mass. The American journal of clinical nutrition 69: 614–620. 10197562 10.1093/ajcn/69.4.614

[45] Brummett BH, Babyak MA, Williams RB, Barefoot JC, Costa PT, et al (2006) NEO personality domains and gender predict levels and trends in body mass index over 14 years during midlife. J Res Pers 40: 222–236.

[46] Chapman BP, Fiscella K, Duberstein P, Kawachi I, Coletta M (2009) Can the influence of childhood socioeconomic status on men's and women's adult body mass be explained by adult socioeconomic status or personality? Findings from a national sample. Health psychology: official journal of the Division of Health Psychology, American Psychological Association 28: 419–427. 10.1037/a0015212 PMC2732202 19594266

[47] Sutin AR, Costa PT Jr, Uda M, Ferrucci L, Schlessinger D, et al (2010) Personality and metabolic syndrome. Age 32: 513–519. 20567927 10.1007/s11357-010-9153-9 PMC2980597

[48] Jokela M, Hintsanen M, Hakulinen C, Batty GD, Nabi H, et al (2013) Association of personality with the development and persistence of obesity: a meta-analysis based on individual-participant data. Obesity reviews: an official journal of the International Association for the Study of Obesity 14: 315–323. 23176713 10.1111/obr.12007 PMC3717171

[49] Jokela M, Elovainio M, Nyberg ST, Tabak AG, Hintsa T, et al.. (2013) Personality and Risk of Diabetes in Adults: Pooled Analysis of 5 Cohort Studies. Health psychology: official journal of the Division of Health Psychology, American Psychological Association 10.1037/hea0000003. 10.1037/hea0000003 23957901

[50] Franks P, Chapman B, Duberstein P, Jerant A (2009) Five factor model personality factors moderated the effects of an intervention to enhance chronic disease management self-efficacy. British journal of health psychology 14: 473–487. 18808733 10.1348/135910708X360700 PMC2745728

[51] Terracciano A, Lockenhoff CE, Zonderman AB, Ferrucci L, Costa PT Jr (2008) Personality predictors of longevity: activity, emotional stability, and conscientiousness. Psychosomatic medicine 70: 621–627. 18596250 10.1097/PSY.0b013e31817b9371 PMC2505356

[52] National Cholesterol Education Program Expert Panel on Detection E, Treatment of High Blood Cholesterol in A (2002) Third Report of the National Cholesterol Education Program (NCEP) Expert Panel on Detection, Evaluation, and Treatment of High Blood Cholesterol in Adults (Adult Treatment Panel III) final report. Circulation 106: 3143–3421. 12485966

[53] Dormann C (2001) Modeling unmeasured third variables in longitudinal studies. Structural Equation Modeling: A Multidisciplinary Journal 8: 575–598.

