# Supplementary material for: Association between Blood Lipid Levels and Personality Traits in Young Korean Women
Source: PLoS One. 2014 Sep 30;9(9):e108406. doi: 10.1371/journal.pone.0108406 (PMC4182467; doi:10.1371/journal.pone.0108406)
Supplement: Table S1 — Comparison of the clinical characteristics of abnormal and normal lipid level groups. (DOC) [file pone.0108406.s001.doc]

**Table S1.** Comparison of the clinical characteristics of abnormal and normal lipid level groups.

|  |  |  | | | | | **Cholesterol (mg/dL)** | | | | | | |  | **Triglycerides (mg/dL)** | | |
| --- | --- | --- | --- | --- | --- | --- | --- | --- | --- | --- | --- | --- | --- | --- | --- | --- | --- |
|  | **Total Cholesterol** | | | |  | **HDL** | | | |  | **Derived LDL** | | |  |
| **Demographics** | **NL**  (n=1386) | | **AL**  (n=315) | ***P*** |  | **NL**  (n=1410) | | **AL**  (n=291) | ***P*** |  | **NL**  (n=1384) | **AL**  (n=317) | ***P*** |  | **NL**  (n=1614) | **AL**  (n=87 ) | ***P*** |
| **Age** | 24.7±4.5 | | 26.0±4.8 |  |  | 24.9±4.5 | | 25.2±4.9 |  |  | 24.6±4.4 | 26.5±4.8 |  |  | 24.9±4.5 | 26.1±5.3 |  |
| **BMI** | 21.4±3.1 | | 22.8±4.3 |  |  | 21.2±2.9 | | 24.1±4.4 |  |  | 21.3±3.1 | 23.2±4.2 |  |  | 21.4±3.1 | 26.1±5.2 |  |
| **Lipid levels** |  | |  |  |  |  | |  |  |  |  |  |  |  |  |  |  |
| **Total cholesterol** | 166.7±18.9 | | 219±19.3 |  |  |  | |  |  |  |  |  |  |  |  |  |  |
| **HDL cholesterol** |  | |  |  |  | 54.0±10.0 | | 35.0±3.3 |  |  |  |  |  |  |  |  |  |
| **Derived LDL cholesterol** |  | |  |  |  |  | |  |  |  | 101.6±16.1 | 147.7±18.3 |  |  |  |  |  |
| **Triglycerides** |  | |  |  |  |  | |  |  |  |  |  |  |  | 71.7±25.9 | 200.4±51.5 |  |
| **Personality traits** |  | |  |  |  |  | |  |  |  |  |  |  |  |  |  |  |
| **Neuroticism** | 57.7±9.8 | | 57.2±10.0 | 0.423 |  | 57.3±9.8 | | 58.6±9.9 | **0.038*** |  | 57.6±9.8 | 57.4±9.8 | 0.718 |  | 57.5±9.7 | 58.9±10.7 | 0.193 |
| **Extraversion** | 60.7±9.5 | | 60.2±9.7 | 0.382 |  | 60.7±9.5 | | 60.3±9.6 | 0.519 |  | 60.7±9.6 | 60.0±9.4 | 0.193 |  | 60.6±9.5 | 59.6±11.1 | 0.345 |
| **Openness** | 64.1±7.9 | | 63.6±7.8 | 0.292 |  | 64.2±7.9 | | 63.4±7.9 | 0.137 |  | 64.1±7.8 | 63.6±8.1 | 0.319 |  | 64.1±7.9 | 62.7±8.1 | 0.096 |
| **Agreeableness** | 59.7±7.5 | | 59.3±7.8 | 0.479 |  | 59.8±7.6 | | 58.9±7.2 | 0.088 |  | 59.6±7.5 | 59.5±7.8 | 0.724 |  | 59.6±7.5 | 59.5±7.2 | 0.841 |
| **Conscientiousness** | 61.1±8.4 | | 59.9±8.1 | **0.027*** |  | 61.1±8.2 | | 60.2±8.8 | 0.109 |  | 61.0±8.3 | 60.4±8.4 | 0.263 |  | 61.0±8.3 | 59.8±8.0 | 0.202 |

Note. NL: normal lipid level, AL: abnormal lipid level

^*^ P < 0.05. Significant results are bolded.
