# Supplementary material for: Association between Blood Lipid Levels and Personality Traits in Young Korean Women
Source: PLoS One. 2014 Sep 30;9(9):e108406. doi: 10.1371/journal.pone.0108406 (PMC4182467; doi:10.1371/journal.pone.0108406)
Supplement: Linear Regression S1 — The results of the linear regression using SAS program. TC: Total Cholesterol, TG: Triglyceride, N: Neuroticism, E: extraversion, O: Openness, A: Agreeableness, C: Conscientiousness, BMI: Body Mass Index, SBP: Systolic pressure, D1_al: Dummy1 for alcohol, D2_al: Dummy2 for alcohol, D1_sm: Dummy1 for smoking, and D2_sm: Dummy2 for smoking. (PDF) [file pone.0108406.s003.pdf]

Linear Regression Results

The REG Procedure
Model: Linear_Regression_Model
Dependent Variable: TC

Number of Observations Read	1701
Number of Observations Used	1701

Analysis of Variance					
Source	DF	Sum of Squares	Mean Square	F Value	Pr > F
Model	8	71294	8911.74913	11.92	<.0001
Error	1692	1265180	747.74257		
Corrected Total	1700	1336474			

Root MSE	27.34488	R-Square	0.0533
Dependent Mean	176.57202	Adj R-Sq	0.0489
Coeff Var	15.48653		

Parameter Estimates							
Variable	DF	Parameter Estimate	Standard Error	t Value	Pr > |t|	Standardized Estimate	Variance Inflation
Intercept	1	127.78981	9.38029	13.62	<.0001	0	0
N	1	-0.06796	0.06887	-0.99	0.3239	-0.02374	1.03449
age	1	1.06660	0.14939	7.14	<.0001	0.17291	1.04833
BMI	1	1.15610	0.20977	5.51	<.0001	0.14022	1.15696
SBP	1	0.01208	0.07393	0.16	0.8702	0.00414	1.14438
D1_al	1	0.08410	1.82513	0.05	0.9633	0.00111	1.04203
D2_al	1	0.31431	2.20719	0.14	0.8868	0.00342	1.03319
D1_sm	1	-3.73120	3.08058	-1.21	0.2260	-0.02884	1.01344
D2_sm	1	-1.56085	3.00894	-0.52	0.6040	-0.01247	1.03207

Linear Regression Results

The REG Procedure
Model: Linear_Regression_Model
Dependent Variable: TC

Number of Observations Read	1701
Number of Observations Used	1701

Analysis of Variance					
Source	DF	Sum of Squares	Mean Square	F Value	Pr > F
Model	8	70637	8829.61669	11.80	<.0001
Error	1692	1265837	748.13091		
Corrected Total	1700	1336474			

Root MSE	27.35198	R-Square	0.0529
Dependent Mean	176.57202	Adj R-Sq	0.0484
Coeff Var	15.49055		

Parameter Estimates							
Variable	DF	Parameter Estimate	Standard Error	t Value	Pr > |t|	Standardized Estimate	Variance Inflation
Intercept	1	124.89510	9.46740	13.19	<.0001	0	0
E	1	-0.02156	0.06994	-0.31	0.7580	-0.00734	1.01439
age	1	1.08581	0.14823	7.33	<.0001	0.17602	1.03148
BMI	1	1.14295	0.20936	5.46	<.0001	0.13862	1.15179
SBP	1	0.01307	0.07394	0.18	0.8597	0.00447	1.14418
D1_al	1	0.10992	1.82603	0.06	0.9520	0.00145	1.04252
D2_al	1	0.18361	2.20773	0.08	0.9337	0.00200	1.03316
D1_sm	1	-3.71326	3.08138	-1.21	0.2283	-0.02870	1.01344
D2_sm	1	-1.61941	3.00977	-0.54	0.5906	-0.01293	1.03210

Linear Regression Results

The REG Procedure
Model: Linear_Regression_Model
Dependent Variable: TC

Number of Observations Read	1701
Number of Observations Used	1701

Analysis of Variance					
Source	DF	Sum of Squares	Mean Square	F Value	Pr > F
Model	8	71332	8916.56049	11.93	<.0001
Error	1692	1265142	747.71983		
Corrected Total	1700	1336474			

Root MSE	27.34447	R-Square	0.0534
Dependent Mean	176.57202	Adj R-Sq	0.0489
Coeff Var	15.48630		

Parameter Estimates							
Variable	DF	Parameter Estimate	Standard Error	t Value	Pr > |t|	Standardized Estimate	Variance Inflation
Intercept	1	129.44687	10.17768	12.72	<.0001	0	0
O	1	-0.08555	0.08449	-1.01	0.3114	-0.02409	1.01164
age	1	1.07971	0.14781	7.30	<.0001	0.17503	1.02619
BMI	1	1.14040	0.20927	5.45	<.0001	0.13831	1.15143
SBP	1	0.01160	0.07394	0.16	0.8754	0.00397	1.14460
D1_al	1	0.04375	1.82646	0.02	0.9809	0.00057876	1.04359
D2_al	1	0.10947	2.20728	0.05	0.9605	0.00119	1.03331
D1_sm	1	-3.75127	3.08068	-1.22	0.2235	-0.02900	1.01354
D2_sm	1	-1.51769	3.01033	-0.50	0.6142	-0.01212	1.03306

Linear Regression Results

The REG Procedure
Model: Linear_Regression_Model
Dependent Variable: TC

Number of Observations Read	1701
Number of Observations Used	1701

Analysis of Variance					
Source	DF	Sum of Squares	Mean Square	F Value	Pr > F
Model	8	70708	8838.45194	11.81	<.0001
Error	1692	1265767	748.08913		
Corrected Total	1700	1336474			

Root MSE	27.35122	R-Square	0.0529
Dependent Mean	176.57202	Adj R-Sq	0.0484
Coeff Var	15.49012		

Parameter Estimates							
Variable	DF	Parameter Estimate	Standard Error	t Value	Pr > |t|	Standardized Estimate	Variance Inflation
Intercept	1	121.41653	9.59214	12.66	<.0001	0	0
A	1	0.03892	0.08942	0.44	0.6634	0.01045	1.02883
age	1	1.08333	0.14836	7.30	<.0001	0.17562	1.03343
BMI	1	1.14668	0.20962	5.47	<.0001	0.13908	1.15478
SBP	1	0.01153	0.07402	0.16	0.8762	0.00395	1.14669
D1_al	1	0.06944	1.83001	0.04	0.9697	0.00091867	1.04713
D2_al	1	0.17746	2.20703	0.08	0.9359	0.00193	1.03256
D1_sm	1	-3.66806	3.08336	-1.19	0.2344	-0.02835	1.01480
D2_sm	1	-1.55459	3.01583	-0.52	0.6063	-0.01242	1.03632

Linear Regression Results

The REG Procedure
Model: Linear_Regression_Model
Dependent Variable: TC

Number of Observations Read	1701
Number of Observations Used	1701

Analysis of Variance					
Source	DF	Sum of Squares	Mean Square	F Value	Pr > F
Model	8	70573	8821.57348	11.79	<.0001
Error	1692	1265902	748.16894		
Corrected Total	1700	1336474			

Root MSE	27.35268	R-Square	0.0528
Dependent Mean	176.57202	Adj R-Sq	0.0483
Coeff Var	15.49095		

Parameter Estimates							
Variable	DF	Parameter Estimate	Standard Error	t Value	Pr > |t|	Standardized Estimate	Variance Inflation
Intercept	1	123.94426	9.54797	12.98	<.0001	0	0
C	1	-0.00765	0.08074	-0.09	0.9245	-0.00227	1.02281
age	1	1.09202	0.14842	7.36	<.0001	0.17703	1.03417
BMI	1	1.14022	0.20994	5.43	<.0001	0.13829	1.15813
SBP	1	0.01317	0.07396	0.18	0.8586	0.00451	1.14462
D1_al	1	0.13434	1.82620	0.07	0.9414	0.00178	1.04266
D2_al	1	0.21326	2.20554	0.10	0.9230	0.00232	1.03106
D1_sm	1	-3.72985	3.08416	-1.21	0.2267	-0.02883	1.01522
D2_sm	1	-1.65837	3.01098	-0.55	0.5819	-0.01324	1.03288

Linear Regression Results

The REG Procedure
Model: Linear_Regression_Model
Dependent Variable: HDL

Number of Observations Read	1701
Number of Observations Used	1701

Analysis of Variance					
Source	DF	Sum of Squares	Mean Square	F Value	Pr > F
Model	8	27242	3405.30502	28.52	<.0001
Error	1692	202030	119.40316		
Corrected Total	1700	229273			

Root MSE	10.92718	R-Square	0.1188
Dependent Mean	50.72310	Adj R-Sq	0.1147
Coeff Var	21.54280		

Parameter Estimates							
Variable	DF	Parameter Estimate	Standard Error	t Value	Pr > |t|	Standardized Estimate	Variance Inflation
Intercept	1	81.66766	3.74842	21.79	<.0001	0	0
N	1	-0.06233	0.02752	-2.26	0.0237	-0.05257	1.03449
age	1	-0.13933	0.05970	-2.33	0.0197	-0.05453	1.04833
BMI	1	-1.13079	0.08383	-13.49	<.0001	-0.33112	1.15696
SBP	1	0.00863	0.02954	0.29	0.7703	0.00713	1.14438
D1_al	1	-1.68435	0.72933	-2.31	0.0210	-0.05380	1.04203
D2_al	1	-0.71850	0.88200	-0.81	0.4154	-0.01890	1.03319
D1_sm	1	0.05486	1.23102	0.04	0.9645	0.00102	1.01344
D2_sm	1	0.45246	1.20239	0.38	0.7067	0.00872	1.03207

Linear Regression Results

The REG Procedure
Model: Linear_Regression_Model
Dependent Variable: HDL

Number of Observations Read	1701
Number of Observations Used	1701

Analysis of Variance					
Source	DF	Sum of Squares	Mean Square	F Value	Pr > F
Model	8	26893	3361.58352	28.10	<.0001
Error	1692	202380	119.60988		
Corrected Total	1700	229273			

Root MSE	10.93663	R-Square	0.1173
Dependent Mean	50.72310	Adj R-Sq	0.1131
Coeff Var	21.56144		

Parameter Estimates							
Variable	DF	Parameter Estimate	Standard Error	t Value	Pr > |t|	Standardized Estimate	Variance Inflation
Intercept	1	75.04923	3.78552	19.83	<.0001	0	0
E	1	0.04144	0.02797	1.48	0.1386	0.03409	1.01439
age	1	-0.10859	0.05927	-1.83	0.0671	-0.04250	1.03148
BMI	1	-1.14629	0.08371	-13.69	<.0001	-0.33566	1.15179
SBP	1	0.00943	0.02957	0.32	0.7497	0.00780	1.14418
D1_al	1	-1.60840	0.73013	-2.20	0.0277	-0.05137	1.04252
D2_al	1	-0.75043	0.88276	-0.85	0.3954	-0.01974	1.03316
D1_sm	1	0.05913	1.23208	0.05	0.9617	0.00110	1.01344
D2_sm	1	0.32083	1.20345	0.27	0.7898	0.00619	1.03210

Linear Regression Results

The REG Procedure
Model: Linear_Regression_Model
Dependent Variable: HDL

Number of Observations Read	1701
Number of Observations Used	1701

Analysis of Variance					
Source	DF	Sum of Squares	Mean Square	F Value	Pr > F
Model	8	26836	3354.52484	28.04	<.0001
Error	1692	202436	119.64325		
Corrected Total	1700	229273			

Root MSE	10.93816	R-Square	0.1170
Dependent Mean	50.72310	Adj R-Sq	0.1129
Coeff Var	21.56444		

Parameter Estimates							
Variable	DF	Parameter Estimate	Standard Error	t Value	Pr > |t|	Standardized Estimate	Variance Inflation
Intercept	1	74.64820	4.07121	18.34	<.0001	0	0
O	1	0.04437	0.03380	1.31	0.1894	0.03016	1.01164
age	1	-0.11192	0.05912	-1.89	0.0585	-0.04380	1.02619
BMI	1	-1.14327	0.08371	-13.66	<.0001	-0.33478	1.15143
SBP	1	0.01025	0.02958	0.35	0.7290	0.00847	1.14460
D1_al	1	-1.59992	0.73061	-2.19	0.0287	-0.05110	1.04359
D2_al	1	-0.75546	0.88294	-0.86	0.3923	-0.01987	1.03331
D1_sm	1	0.08487	1.23231	0.07	0.9451	0.00158	1.01354
D2_sm	1	0.30660	1.20417	0.25	0.7991	0.00591	1.03306

Linear Regression Results

The REG Procedure
Model: Linear_Regression_Model
Dependent Variable: HDL

Number of Observations Read	1701
Number of Observations Used	1701

Analysis of Variance					
Source	DF	Sum of Squares	Mean Square	F Value	Pr > F
Model	8	26995	3374.35687	28.23	<.0001
Error	1692	202278	119.54948		
Corrected Total	1700	229273			

Root MSE	10.93387	R-Square	0.1177
Dependent Mean	50.72310	Adj R-Sq	0.1136
Coeff Var	21.55599		

Parameter Estimates							
Variable	DF	Parameter Estimate	Standard Error	t Value	Pr > |t|	Standardized Estimate	Variance Inflation
Intercept	1	74.39137	3.83453	19.40	<.0001	0	0
A	1	0.06244	0.03574	1.75	0.0808	0.04046	1.02883
age	1	-0.12887	0.05931	-2.17	0.0299	-0.05044	1.03343
BMI	1	-1.13603	0.08380	-13.56	<.0001	-0.33266	1.15478
SBP	1	0.00709	0.02959	0.24	0.8108	0.00585	1.14669
D1_al	1	-1.73826	0.73156	-2.38	0.0176	-0.05552	1.04713
D2_al	1	-0.86950	0.88228	-0.99	0.3245	-0.02287	1.03256
D1_sm	1	0.14676	1.23260	0.12	0.9052	0.00274	1.01480
D2_sm	1	0.52160	1.20560	0.43	0.6653	0.01006	1.03632

Linear Regression Results

The REG Procedure
Model: Linear_Regression_Model
Dependent Variable: HDL

Number of Observations Read	1701
Number of Observations Used	1701

Analysis of Variance					
Source	DF	Sum of Squares	Mean Square	F Value	Pr > F
Model	8	26706	3338.28061	27.88	<.0001
Error	1692	202566	119.72006		
Corrected Total	1700	229273			

Root MSE	10.94167	R-Square	0.1165
Dependent Mean	50.72310	Adj R-Sq	0.1123
Coeff Var	21.57137		

Parameter Estimates							
Variable	DF	Parameter Estimate	Standard Error	t Value	Pr > |t|	Standardized Estimate	Variance Inflation
Intercept	1	76.23415	3.81940	19.96	<.0001	0	0
C	1	0.02577	0.03230	0.80	0.4250	0.01844	1.02281
age	1	-0.12284	0.05937	-2.07	0.0387	-0.04808	1.03417
BMI	1	-1.13885	0.08398	-13.56	<.0001	-0.33348	1.15813
SBP	1	0.00904	0.02959	0.31	0.7601	0.00747	1.14462
D1_al	1	-1.66404	0.73052	-2.28	0.0229	-0.05315	1.04266
D2_al	1	-0.80553	0.88227	-0.91	0.3614	-0.02119	1.03106
D1_sm	1	0.10881	1.23373	0.09	0.9297	0.00203	1.01522
D2_sm	1	0.41235	1.20446	0.34	0.7321	0.00795	1.03288

Linear Regression Results

The REG Procedure
Model: Linear_Regression_Model
Dependent Variable: LDL

Number of Observations Read	1701
Number of Observations Used	1701

Analysis of Variance					
Source	DF	Sum of Squares	Mean Square	F Value	Pr > F
Model	8	84539	10567	19.25	<.0001
Error	1692	929001	549.05501		
Corrected Total	1700	1013541			

Root MSE	23.43192	R-Square	0.0834
Dependent Mean	110.19089	Adj R-Sq	0.0791
Coeff Var	21.26485		

Parameter Estimates							
Variable	DF	Parameter Estimate	Standard Error	t Value	Pr > |t|	Standardized Estimate	Variance Inflation
Intercept	1	55.54775	8.03800	6.91	<.0001	0	0
N	1	0.00591	0.05902	0.10	0.9203	0.00237	1.03449
age	1	1.11636	0.12802	8.72	<.0001	0.20781	1.04833
BMI	1	1.44798	0.17975	8.06	<.0001	0.20166	1.15696
SBP	1	-0.04309	0.06335	-0.68	0.4965	-0.01694	1.14438
D1_al	1	0.53518	1.56396	0.34	0.7322	0.00813	1.04203
D2_al	1	-0.04156	1.89135	-0.02	0.9825	-0.00051990	1.03319
D1_sm	1	-3.39547	2.63976	-1.29	0.1985	-0.03014	1.01344
D2_sm	1	-3.22587	2.57837	-1.25	0.2111	-0.02958	1.03207

Linear Regression Results

The REG Procedure
Model: Linear_Regression_Model
Dependent Variable: LDL

Number of Observations Read	1701
Number of Observations Used	1701

Analysis of Variance					
Source	DF	Sum of Squares	Mean Square	F Value	Pr > F
Model	8	84652	10581	19.27	<.0001
Error	1692	928889	548.98865		
Corrected Total	1700	1013541			

Root MSE	23.43051	R-Square	0.0835
Dependent Mean	110.19089	Adj R-Sq	0.0792
Coeff Var	21.26356		

Parameter Estimates							
Variable	DF	Parameter Estimate	Standard Error	t Value	Pr > |t|	Standardized Estimate	Variance Inflation
Intercept	1	57.71760	8.11005	7.12	<.0001	0	0
E	1	-0.02775	0.05991	-0.46	0.6433	-0.01086	1.01439
age	1	1.10834	0.12698	8.73	<.0001	0.20632	1.03148
BMI	1	1.45078	0.17934	8.09	<.0001	0.20205	1.15179
SBP	1	-0.04313	0.06334	-0.68	0.4960	-0.01695	1.14418
D1_al	1	0.50764	1.56423	0.32	0.7456	0.00771	1.04252
D2_al	1	-0.07276	1.89121	-0.04	0.9693	-0.00091011	1.03316
D1_sm	1	-3.39113	2.63960	-1.28	0.1991	-0.03010	1.01344
D2_sm	1	-3.18307	2.57826	-1.23	0.2172	-0.02919	1.03210

Linear Regression Results

The REG Procedure
Model: Linear_Regression_Model
Dependent Variable: LDL

Number of Observations Read	1701
Number of Observations Used	1701

Analysis of Variance					
Source	DF	Sum of Squares	Mean Square	F Value	Pr > F
Model	8	85018	10627	19.37	<.0001
Error	1692	928523	548.77244		
Corrected Total	1700	1013541			

Root MSE	23.42589	R-Square	0.0839
Dependent Mean	110.19089	Adj R-Sq	0.0796
Coeff Var	21.25937		

Parameter Estimates							
Variable	DF	Parameter Estimate	Standard Error	t Value	Pr > |t|	Standardized Estimate	Variance Inflation
Intercept	1	60.64458	8.71917	6.96	<.0001	0	0
O	1	-0.06795	0.07238	-0.94	0.3480	-0.02197	1.01164
age	1	1.10577	0.12662	8.73	<.0001	0.20584	1.02619
BMI	1	1.44816	0.17928	8.08	<.0001	0.20169	1.15143
SBP	1	-0.04432	0.06334	-0.70	0.4842	-0.01742	1.14460
D1_al	1	0.46416	1.56472	0.30	0.7668	0.00705	1.04359
D2_al	1	-0.11637	1.89097	-0.06	0.9509	-0.00146	1.03331
D1_sm	1	-3.42344	2.63921	-1.30	0.1948	-0.03039	1.01354
D2_sm	1	-3.11580	2.57894	-1.21	0.2271	-0.02857	1.03306

Linear Regression Results

The REG Procedure
Model: Linear_Regression_Model
Dependent Variable: LDL

Number of Observations Read	1701
Number of Observations Used	1701

Analysis of Variance					
Source	DF	Sum of Squares	Mean Square	F Value	Pr > F
Model	8	84613	10577	19.26	<.0001
Error	1692	928928	549.01157		
Corrected Total	1700	1013541			

Root MSE	23.43100	R-Square	0.0835
Dependent Mean	110.19089	Adj R-Sq	0.0791
Coeff Var	21.26401		

Parameter Estimates							
Variable	DF	Parameter Estimate	Standard Error	t Value	Pr > |t|	Standardized Estimate	Variance Inflation
Intercept	1	57.47544	8.21731	6.99	<.0001	0	0
A	1	-0.02906	0.07660	-0.38	0.7045	-0.00895	1.02883
age	1	1.11959	0.12710	8.81	<.0001	0.20841	1.03343
BMI	1	1.44553	0.17958	8.05	<.0001	0.20132	1.15478
SBP	1	-0.04205	0.06341	-0.66	0.5073	-0.01653	1.14669
D1_al	1	0.57530	1.56771	0.37	0.7137	0.00874	1.04713
D2_al	1	-0.00519	1.89070	-0.00	0.9978	-0.00006494	1.03256
D1_sm	1	-3.43359	2.64142	-1.30	0.1938	-0.03048	1.01480
D2_sm	1	-3.28727	2.58357	-1.27	0.2034	-0.03015	1.03632

Linear Regression Results

The REG Procedure
Model: Linear_Regression_Model
Dependent Variable: LDL

Number of Observations Read	1701
Number of Observations Used	1701

Analysis of Variance					
Source	DF	Sum of Squares	Mean Square	F Value	Pr > F
Model	8	84915	10614	19.34	<.0001
Error	1692	928625	548.83275		
Corrected Total	1700	1013541			

Root MSE	23.42718	R-Square	0.0838
Dependent Mean	110.19089	Adj R-Sq	0.0794
Coeff Var	21.26054		

Parameter Estimates							
Variable	DF	Parameter Estimate	Standard Error	t Value	Pr > |t|	Standardized Estimate	Variance Inflation
Intercept	1	59.27326	8.17770	7.25	<.0001	0	0
C	1	-0.05766	0.06916	-0.83	0.4045	-0.01962	1.02281
age	1	1.12628	0.12712	8.86	<.0001	0.20966	1.03417
BMI	1	1.43778	0.17981	8.00	<.0001	0.20024	1.15813
SBP	1	-0.04214	0.06335	-0.67	0.5060	-0.01656	1.14462
D1_al	1	0.57664	1.56411	0.37	0.7124	0.00876	1.04266
D2_al	1	-0.04282	1.88902	-0.02	0.9819	-0.00053564	1.03106
D1_sm	1	-3.48936	2.64154	-1.32	0.1867	-0.03097	1.01522
D2_sm	1	-3.30512	2.57886	-1.28	0.2002	-0.03031	1.03288

Linear Regression Results

The REG Procedure
Model: Linear_Regression_Model
Dependent Variable: TG

Number of Observations Read	1701
Number of Observations Used	1701

Analysis of Variance					
Source	DF	Sum of Squares	Mean Square	F Value	Pr > F
Model	8	427922	53490	40.22	<.0001
Error	1692	2250095	1329.84333		
Corrected Total	1700	2678017			

Root MSE	36.46702	R-Square	0.1598
Dependent Mean	78.31805	Adj R-Sq	0.1558
Coeff Var	46.56272		

Parameter Estimates							
Variable	DF	Parameter Estimate	Standard Error	t Value	Pr > |t|	Standardized Estimate	Variance Inflation
Intercept	1	-48.24147	12.50952	-3.86	0.0001	0	0
N	1	-0.03595	0.09185	-0.39	0.6956	-0.00887	1.03449
age	1	0.43447	0.19923	2.18	0.0293	0.04976	1.04833
BMI	1	4.17284	0.27975	14.92	<.0001	0.35753	1.15696
SBP	1	0.23825	0.09859	2.42	0.0158	0.05761	1.14438
D1_al	1	6.80143	2.43398	2.79	0.0053	0.06356	1.04203
D2_al	1	5.44993	2.94349	1.85	0.0643	0.04194	1.03319
D1_sm	1	-1.88519	4.10825	-0.46	0.6464	-0.01029	1.01344
D2_sm	1	5.80287	4.01271	1.45	0.1483	0.03274	1.03207

Linear Regression Results

The REG Procedure
Model: Linear_Regression_Model
Dependent Variable: TG

Number of Observations Read	1701
Number of Observations Used	1701

Analysis of Variance					
Source	DF	Sum of Squares	Mean Square	F Value	Pr > F
Model	8	433348	54169	40.83	<.0001
Error	1692	2244669	1326.63645		
Corrected Total	1700	2678017			

Root MSE	36.42302	R-Square	0.1618
Dependent Mean	78.31805	Adj R-Sq	0.1579
Coeff Var	46.50655		

Parameter Estimates							
Variable	DF	Parameter Estimate	Standard Error	t Value	Pr > |t|	Standardized Estimate	Variance Inflation
Intercept	1	-38.08729	12.60718	-3.02	0.0026	0	0
E	1	-0.19187	0.09314	-2.06	0.0396	-0.04618	1.01439
age	1	0.40594	0.19739	2.06	0.0399	0.04649	1.03148
BMI	1	4.17603	0.27879	14.98	<.0001	0.35780	1.15179
SBP	1	0.23907	0.09847	2.43	0.0153	0.05780	1.14418
D1_al	1	6.66100	2.43161	2.74	0.0062	0.06225	1.04252
D2_al	1	5.12156	2.93990	1.74	0.0817	0.03941	1.03316
D1_sm	1	-1.83981	4.10329	-0.45	0.6539	-0.01005	1.01344
D2_sm	1	6.00162	4.00794	1.50	0.1345	0.03386	1.03210

Linear Regression Results

The REG Procedure
Model: Linear_Regression_Model
Dependent Variable: TG

Number of Observations Read	1701
Number of Observations Used	1701

Analysis of Variance					
Source	DF	Sum of Squares	Mean Square	F Value	Pr > F
Model	8	436882	54610	41.23	<.0001
Error	1692	2241135	1324.54795		
Corrected Total	1700	2678017			

Root MSE	36.39434	R-Square	0.1631
Dependent Mean	78.31805	Adj R-Sq	0.1592
Coeff Var	46.46993		

Parameter Estimates							
Variable	DF	Parameter Estimate	Standard Error	t Value	Pr > |t|	Standardized Estimate	Variance Inflation
Intercept	1	-29.94797	13.54606	-2.21	0.0272	0	0
O	1	-0.29578	0.11245	-2.63	0.0086	-0.05884	1.01164
age	1	0.41002	0.19672	2.08	0.0373	0.04696	1.02619
BMI	1	4.16062	0.27853	14.94	<.0001	0.35648	1.15143
SBP	1	0.23379	0.09841	2.38	0.0176	0.05653	1.14460
D1_al	1	6.53240	2.43094	2.69	0.0073	0.06105	1.04359
D2_al	1	5.03381	2.93780	1.71	0.0868	0.03874	1.03331
D1_sm	1	-1.99458	4.10026	-0.49	0.6267	-0.01089	1.01354
D2_sm	1	6.20395	4.00663	1.55	0.1217	0.03500	1.03306

Linear Regression Results

The REG Procedure
Model: Linear_Regression_Model
Dependent Variable: TG

Number of Observations Read	1701
Number of Observations Used	1701

Analysis of Variance					
Source	DF	Sum of Squares	Mean Square	F Value	Pr > F
Model	8	427743	53468	40.20	<.0001
Error	1692	2250274	1329.94894		
Corrected Total	1700	2678017			

Root MSE	36.46846	R-Square	0.1597
Dependent Mean	78.31805	Adj R-Sq	0.1558
Coeff Var	46.56457		

Parameter Estimates							
Variable	DF	Parameter Estimate	Standard Error	t Value	Pr > |t|	Standardized Estimate	Variance Inflation
Intercept	1	-51.38604	12.78958	-4.02	<.0001	0	0
A	1	0.01636	0.11922	0.14	0.8909	0.00310	1.02883
age	1	0.44409	0.19782	2.24	0.0249	0.05086	1.03343
BMI	1	4.16732	0.27950	14.91	<.0001	0.35706	1.15478
SBP	1	0.23812	0.09870	2.41	0.0159	0.05757	1.14669
D1_al	1	6.80008	2.44002	2.79	0.0054	0.06355	1.04713
D2_al	1	5.38158	2.94272	1.83	0.0676	0.04141	1.03256
D1_sm	1	-1.85717	4.11116	-0.45	0.6515	-0.01014	1.01480
D2_sm	1	5.79615	4.02112	1.44	0.1496	0.03270	1.03632

Linear Regression Results

The REG Procedure
Model: Linear_Regression_Model
Dependent Variable: TG

Number of Observations Read	1701
Number of Observations Used	1701

Analysis of Variance					
Source	DF	Sum of Squares	Mean Square	F Value	Pr > F
Model	8	429026	53628	40.35	<.0001
Error	1692	2248991	1329.19062		
Corrected Total	1700	2678017			

Root MSE	36.45807	R-Square	0.1602
Dependent Mean	78.31805	Adj R-Sq	0.1562
Coeff Var	46.55130		

Parameter Estimates							
Variable	DF	Parameter Estimate	Standard Error	t Value	Pr > |t|	Standardized Estimate	Variance Inflation
Intercept	1	-56.71845	12.72638	-4.46	<.0001	0	0
C	1	0.10676	0.10762	0.99	0.3213	0.02235	1.02281
age	1	0.42487	0.19783	2.15	0.0319	0.04866	1.03417
BMI	1	4.18644	0.27983	14.96	<.0001	0.35869	1.15813
SBP	1	0.23683	0.09858	2.40	0.0164	0.05726	1.14462
D1_al	1	6.74094	2.43412	2.77	0.0057	0.06300	1.04266
D2_al	1	5.41556	2.93974	1.84	0.0656	0.04167	1.03106
D1_sm	1	-1.70632	4.11084	-0.42	0.6781	-0.00932	1.01522
D2_sm	1	5.91795	4.01330	1.47	0.1405	0.03339	1.03288

Linear Regression Results

The REG Procedure
Model: Linear_Regression_Model
Dependent Variable: TC

Number of Observations Read	1701
Number of Observations Used	1701

Analysis of Variance					
Source	DF	Sum of Squares	Mean Square	F Value	Pr > F
Model	8	70646	8830.79666	11.80	<.0001
Error	1692	1265828	748.12533		
Corrected Total	1700	1336474			

Root MSE	27.35188	R-Square	0.0529
Dependent Mean	176.57202	Adj R-Sq	0.0484
Coeff Var	15.49050		

Parameter Estimates							
Variable	DF	Parameter Estimate	Standard Error	t Value	Pr > |t|	Standardized Estimate	Variance Inflation
Intercept	1	124.74863	9.14471	13.64	<.0001	0	0
N1	1	-0.10169	0.30998	-0.33	0.7429	-0.00783	1.01663
age	1	1.08447	0.14858	7.30	<.0001	0.17580	1.03644
BMI	1	1.14068	0.20934	5.45	<.0001	0.13835	1.15165
SBP	1	0.01348	0.07396	0.18	0.8554	0.00461	1.14457
D1_al	1	0.12066	1.82519	0.07	0.9473	0.00160	1.04158
D2_al	1	0.24078	2.20688	0.11	0.9131	0.00262	1.03237
D1_sm	1	-3.70105	3.08175	-1.20	0.2299	-0.02861	1.01369
D2_sm	1	-1.63813	3.00856	-0.54	0.5862	-0.01308	1.03128

Linear Regression Results

The REG Procedure
Model: Linear_Regression_Model
Dependent Variable: HDL

Number of Observations Read	1701
Number of Observations Used	1701

Analysis of Variance					
Source	DF	Sum of Squares	Mean Square	F Value	Pr > F
Model	8	26744	3342.97597	27.93	<.0001
Error	1692	202529	119.69786		
Corrected Total	1700	229273			

Root MSE	10.94065	R-Square	0.1166
Dependent Mean	50.72310	Adj R-Sq	0.1125
Coeff Var	21.56937		

Parameter Estimates							
Variable	DF	Parameter Estimate	Standard Error	t Value	Pr > |t|	Standardized Estimate	Variance Inflation
Intercept	1	79.21811	3.65785	21.66	<.0001	0	0
N1	1	-0.12090	0.12399	-0.98	0.3297	-0.02246	1.01663
age	1	-0.12456	0.05943	-2.10	0.0362	-0.04875	1.03644
BMI	1	-1.14522	0.08374	-13.68	<.0001	-0.33535	1.15165
SBP	1	0.01003	0.02958	0.34	0.7345	0.00829	1.14457
D1_al	1	-1.65290	0.73007	-2.26	0.0237	-0.05280	1.04158
D2_al	1	-0.77881	0.88274	-0.88	0.3778	-0.02048	1.03237
D1_sm	1	0.08698	1.23269	0.07	0.9438	0.00162	1.01369
D2_sm	1	0.38395	1.20341	0.32	0.7497	0.00740	1.03128

Linear Regression Results

The REG Procedure
Model: Linear_Regression_Model
Dependent Variable: LDL

Number of Observations Read	1701
Number of Observations Used	1701

Analysis of Variance					
Source	DF	Sum of Squares	Mean Square	F Value	Pr > F
Model	8	84542	10568	19.25	<.0001
Error	1692	928999	549.05361		
Corrected Total	1700	1013541			

Root MSE	23.43189	R-Square	0.0834
Dependent Mean	110.19089	Adj R-Sq	0.0791
Coeff Var	21.26482		

Parameter Estimates							
Variable	DF	Parameter Estimate	Standard Error	t Value	Pr > |t|	Standardized Estimate	Variance Inflation
Intercept	1	55.53016	7.83412	7.09	<.0001	0	0
N1	1	0.03178	0.26555	0.12	0.9047	0.00281	1.01663
age	1	1.11615	0.12729	8.77	<.0001	0.20777	1.03644
BMI	1	1.44955	0.17934	8.08	<.0001	0.20188	1.15165
SBP	1	-0.04331	0.06336	-0.68	0.4943	-0.01702	1.14457
D1_al	1	0.53373	1.56361	0.34	0.7329	0.00811	1.04158
D2_al	1	-0.04109	1.89060	-0.02	0.9827	-0.00051392	1.03237
D1_sm	1	-3.40181	2.64008	-1.29	0.1977	-0.03019	1.01369
D2_sm	1	-3.22112	2.57738	-1.25	0.2116	-0.02954	1.03128

Linear Regression Results

The REG Procedure
Model: Linear_Regression_Model
Dependent Variable: TG

Number of Observations Read	1701
Number of Observations Used	1701

Analysis of Variance					
Source	DF	Sum of Squares	Mean Square	F Value	Pr > F
Model	8	427732	53467	40.20	<.0001
Error	1692	2250285	1329.95556		
Corrected Total	1700	2678017			

Root MSE	36.46856	R-Square	0.1597
Dependent Mean	78.31805	Adj R-Sq	0.1557
Coeff Var	46.56469		

Parameter Estimates							
Variable	DF	Parameter Estimate	Standard Error	t Value	Pr > |t|	Standardized Estimate	Variance Inflation
Intercept	1	-51.02839	12.19275	-4.19	<.0001	0	0
N1	1	0.04212	0.41330	0.10	0.9188	0.00229	1.01663
age	1	0.44955	0.19811	2.27	0.0234	0.05148	1.03644
BMI	1	4.16568	0.27912	14.92	<.0001	0.35692	1.15165
SBP	1	0.23857	0.09861	2.42	0.0156	0.05768	1.14457
D1_al	1	6.82800	2.43355	2.81	0.0051	0.06381	1.04158
D2_al	1	5.38632	2.94246	1.83	0.0673	0.04145	1.03237
D1_sm	1	-1.88480	4.10893	-0.46	0.6465	-0.01029	1.01369
D2_sm	1	5.75377	4.01135	1.43	0.1517	0.03246	1.03128

Linear Regression Results

The REG Procedure
Model: Linear_Regression_Model
Dependent Variable: TC

Number of Observations Read	1701
Number of Observations Used	1701

Analysis of Variance					
Source	DF	Sum of Squares	Mean Square	F Value	Pr > F
Model	8	72420	9052.44829	12.12	<.0001
Error	1692	1264055	747.07733		
Corrected Total	1700	1336474			

Root MSE	27.33272	R-Square	0.0542
Dependent Mean	176.57202	Adj R-Sq	0.0497
Coeff Var	15.47964		

Parameter Estimates							
Variable	DF	Parameter Estimate	Standard Error	t Value	Pr > |t|	Standardized Estimate	Variance Inflation
Intercept	1	127.06945	8.61165	14.76	<.0001	0	0
N2	1	-0.46921	0.29787	-1.58	0.1154	-0.03749	1.01321
age	1	1.08690	0.14738	7.37	<.0001	0.17620	1.02116
BMI	1	1.16716	0.20980	5.56	<.0001	0.14156	1.15824
SBP	1	0.01041	0.07391	0.14	0.8880	0.00356	1.14476
D1_al	1	0.04928	1.82445	0.03	0.9785	0.00065190	1.04219
D2_al	1	0.35254	2.20563	0.16	0.8730	0.00384	1.03265
D1_sm	1	-3.69008	3.07923	-1.20	0.2309	-0.02852	1.01345
D2_sm	1	-1.38058	3.01108	-0.46	0.6467	-0.01103	1.03446

Linear Regression Results

The REG Procedure
Model: Linear_Regression_Model
Dependent Variable: HDL

Number of Observations Read	1701
Number of Observations Used	1701

Analysis of Variance					
Source	DF	Sum of Squares	Mean Square	F Value	Pr > F
Model	8	27218	3402.28387	28.49	<.0001
Error	1692	202054	119.41744		
Corrected Total	1700	229273			

Root MSE	10.92783	R-Square	0.1187
Dependent Mean	50.72310	Adj R-Sq	0.1145
Coeff Var	21.54409		

Parameter Estimates							
Variable	DF	Parameter Estimate	Standard Error	t Value	Pr > |t|	Standardized Estimate	Variance Inflation
Intercept	1	79.74392	3.44300	23.16	<.0001	0	0
N2	1	-0.26432	0.11909	-2.22	0.0266	-0.05099	1.01321
age	1	-0.11946	0.05892	-2.03	0.0428	-0.04676	1.02116
BMI	1	-1.12964	0.08388	-13.47	<.0001	-0.33079	1.15824
SBP	1	0.00802	0.02955	0.27	0.7860	0.00663	1.14476
D1_al	1	-1.68832	0.72943	-2.31	0.0208	-0.05393	1.04219
D2_al	1	-0.73225	0.88183	-0.83	0.4064	-0.01926	1.03265
D1_sm	1	0.08285	1.23110	0.07	0.9464	0.00155	1.01345
D2_sm	1	0.52358	1.20385	0.43	0.6637	0.01010	1.03446

Linear Regression Results

The REG Procedure
Model: Linear_Regression_Model
Dependent Variable: LDL

Number of Observations Read	1701
Number of Observations Used	1701

Analysis of Variance					
Source	DF	Sum of Squares	Mean Square	F Value	Pr > F
Model	8	84807	10601	19.31	<.0001
Error	1692	928733	548.89664		
Corrected Total	1700	1013541			

Root MSE	23.42854	R-Square	0.0837
Dependent Mean	110.19089	Adj R-Sq	0.0793
Coeff Var	21.26178		

Parameter Estimates							
Variable	DF	Parameter Estimate	Standard Error	t Value	Pr > |t|	Standardized Estimate	Variance Inflation
Intercept	1	57.29192	7.38157	7.76	<.0001	0	0
N2	1	-0.18021	0.25532	-0.71	0.4804	-0.01653	1.01321
age	1	1.11293	0.12633	8.81	<.0001	0.20718	1.02116
BMI	1	1.45899	0.17983	8.11	<.0001	0.20320	1.15824
SBP	1	-0.04418	0.06335	-0.70	0.4856	-0.01736	1.14476
D1_al	1	0.50098	1.56385	0.32	0.7487	0.00761	1.04219
D2_al	1	0.02010	1.89058	0.01	0.9915	0.00025137	1.03265
D1_sm	1	-3.38610	2.63939	-1.28	0.1997	-0.03006	1.01345
D2_sm	1	-3.11612	2.58099	-1.21	0.2275	-0.02858	1.03446

Linear Regression Results

The REG Procedure
Model: Linear_Regression_Model
Dependent Variable: TG

Number of Observations Read	1701
Number of Observations Used	1701

Analysis of Variance					
Source	DF	Sum of Squares	Mean Square	F Value	Pr > F
Model	8	427770	53471	40.21	<.0001
Error	1692	2250247	1329.93293		
Corrected Total	1700	2678017			

Root MSE	36.46825	R-Square	0.1597
Dependent Mean	78.31805	Adj R-Sq	0.1558
Coeff Var	46.56429		

Parameter Estimates							
Variable	DF	Parameter Estimate	Standard Error	t Value	Pr > |t|	Standardized Estimate	Variance Inflation
Intercept	1	-49.91229	11.48996	-4.34	<.0001	0	0
N2	1	-0.07867	0.39743	-0.20	0.8431	-0.00444	1.01321
age	1	0.44648	0.19664	2.27	0.0233	0.05113	1.02116
BMI	1	4.16950	0.27992	14.90	<.0001	0.35724	1.15824
SBP	1	0.23832	0.09861	2.42	0.0158	0.05762	1.14476
D1_al	1	6.81157	2.43425	2.80	0.0052	0.06366	1.04219
D2_al	1	5.42031	2.94283	1.84	0.0657	0.04171	1.03265
D1_sm	1	-1.87336	4.10841	-0.46	0.6485	-0.01023	1.01345
D2_sm	1	5.80202	4.01749	1.44	0.1489	0.03273	1.03446

Linear Regression Results

The REG Procedure
Model: Linear_Regression_Model
Dependent Variable: TC

Number of Observations Read	1701
Number of Observations Used	1701

Analysis of Variance					
Source	DF	Sum of Squares	Mean Square	F Value	Pr > F
Model	8	70569	8821.14870	11.79	<.0001
Error	1692	1265905	748.17094		
Corrected Total	1700	1336474			

Root MSE	27.35271	R-Square	0.0528
Dependent Mean	176.57202	Adj R-Sq	0.0483
Coeff Var	15.49097		

Parameter Estimates							
Variable	DF	Parameter Estimate	Standard Error	t Value	Pr > |t|	Standardized Estimate	Variance Inflation
Intercept	1	123.67444	8.71963	14.18	<.0001	0	0
N3	1	-0.01645	0.24673	-0.07	0.9469	-0.00160	1.02479
age	1	1.08911	0.14881	7.32	<.0001	0.17655	1.03950
BMI	1	1.14266	0.20979	5.45	<.0001	0.13859	1.15648
SBP	1	0.01296	0.07395	0.18	0.8609	0.00444	1.14443
D1_al	1	0.12493	1.82581	0.07	0.9455	0.00165	1.04221
D2_al	1	0.22011	2.20706	0.10	0.9206	0.00240	1.03248
D1_sm	1	-3.72002	3.08165	-1.21	0.2275	-0.02875	1.01357
D2_sm	1	-1.64321	3.00903	-0.55	0.5851	-0.01312	1.03154

Linear Regression Results

The REG Procedure
Model: Linear_Regression_Model
Dependent Variable: HDL

Number of Observations Read	1701
Number of Observations Used	1701

Analysis of Variance					
Source	DF	Sum of Squares	Mean Square	F Value	Pr > F
Model	8	26966	3370.75618	28.19	<.0001
Error	1692	202307	119.56651		
Corrected Total	1700	229273			

Root MSE	10.93465	R-Square	0.1176
Dependent Mean	50.72310	Adj R-Sq	0.1134
Coeff Var	21.55753		

Parameter Estimates							
Variable	DF	Parameter Estimate	Standard Error	t Value	Pr > |t|	Standardized Estimate	Variance Inflation
Intercept	1	79.49408	3.48580	22.81	<.0001	0	0
N3	1	-0.16535	0.09863	-1.68	0.0938	-0.03876	1.02479
age	1	-0.13081	0.05949	-2.20	0.0280	-0.05120	1.03950
BMI	1	-1.13463	0.08387	-13.53	<.0001	-0.33225	1.15648
SBP	1	0.00877	0.02956	0.30	0.7667	0.00725	1.14443
D1_al	1	-1.67791	0.72989	-2.30	0.0216	-0.05359	1.04221
D2_al	1	-0.75436	0.88231	-0.85	0.3927	-0.01984	1.03248
D1_sm	1	0.04245	1.23194	0.03	0.9725	0.00079226	1.01357
D2_sm	1	0.41021	1.20290	0.34	0.7331	0.00791	1.03154

Linear Regression Results

The REG Procedure
Model: Linear_Regression_Model
Dependent Variable: LDL

Number of Observations Read	1701
Number of Observations Used	1701

Analysis of Variance					
Source	DF	Sum of Squares	Mean Square	F Value	Pr > F
Model	8	84787	10598	19.31	<.0001
Error	1692	928754	548.90894		
Corrected Total	1700	1013541			

Root MSE	23.42881	R-Square	0.0837
Dependent Mean	110.19089	Adj R-Sq	0.0793
Coeff Var	21.26202		

Parameter Estimates							
Variable	DF	Parameter Estimate	Standard Error	t Value	Pr > |t|	Standardized Estimate	Variance Inflation
Intercept	1	54.39343	7.46875	7.28	<.0001	0	0
N3	1	0.14337	0.21133	0.68	0.4976	0.01598	1.02479
age	1	1.12584	0.12746	8.83	<.0001	0.20958	1.03950
BMI	1	1.44113	0.17969	8.02	<.0001	0.20071	1.15648
SBP	1	-0.04254	0.06334	-0.67	0.5019	-0.01672	1.14443
D1_al	1	0.56092	1.56389	0.36	0.7199	0.00852	1.04221
D2_al	1	-0.08111	1.89045	-0.04	0.9658	-0.00101	1.03248
D1_sm	1	-3.37504	2.63957	-1.28	0.2012	-0.02996	1.01357
D2_sm	1	-3.25015	2.57737	-1.26	0.2075	-0.02981	1.03154

Linear Regression Results

The REG Procedure
Model: Linear_Regression_Model
Dependent Variable: TG

Number of Observations Read	1701
Number of Observations Used	1701

Analysis of Variance					
Source	DF	Sum of Squares	Mean Square	F Value	Pr > F
Model	8	427757	53470	40.20	<.0001
Error	1692	2250260	1329.94066		
Corrected Total	1700	2678017			

Root MSE	36.46835	R-Square	0.1597
Dependent Mean	78.31805	Adj R-Sq	0.1558
Coeff Var	46.56443		

Parameter Estimates							
Variable	DF	Parameter Estimate	Standard Error	t Value	Pr > |t|	Standardized Estimate	Variance Inflation
Intercept	1	-51.11124	11.62556	-4.40	<.0001	0	0
N3	1	0.05636	0.32895	0.17	0.8640	0.00387	1.02479
age	1	0.45162	0.19840	2.28	0.0230	0.05172	1.03950
BMI	1	4.16206	0.27970	14.88	<.0001	0.35661	1.15648
SBP	1	0.23900	0.09860	2.42	0.0155	0.05779	1.14443
D1_al	1	6.83645	2.43428	2.81	0.0050	0.06389	1.04221
D2_al	1	5.37822	2.94259	1.83	0.0678	0.04139	1.03248
D1_sm	1	-1.86947	4.10866	-0.46	0.6492	-0.01021	1.01357
D2_sm	1	5.74489	4.01183	1.43	0.1523	0.03241	1.03154

Linear Regression Results

The REG Procedure
Model: Linear_Regression_Model
Dependent Variable: TC

Number of Observations Read	1701
Number of Observations Used	1701

Analysis of Variance					
Source	DF	Sum of Squares	Mean Square	F Value	Pr > F
Model	8	71752	8968.97525	12.00	<.0001
Error	1692	1264723	747.47200		
Corrected Total	1700	1336474			

Root MSE	27.33993	R-Square	0.0537
Dependent Mean	176.57202	Adj R-Sq	0.0492
Coeff Var	15.48373		

Parameter Estimates							
Variable	DF	Parameter Estimate	Standard Error	t Value	Pr > |t|	Standardized Estimate	Variance Inflation
Intercept	1	127.75240	8.97003	14.24	<.0001	0	0
N4	1	-0.38696	0.30720	-1.26	0.2080	-0.03002	1.01548
age	1	1.06968	0.14832	7.21	<.0001	0.17341	1.03368
BMI	1	1.14320	0.20923	5.46	<.0001	0.13865	1.15142
SBP	1	0.01359	0.07391	0.18	0.8542	0.00465	1.14422
D1_al	1	0.14628	1.82430	0.08	0.9361	0.00194	1.04147
D2_al	1	0.25285	2.20468	0.11	0.9087	0.00275	1.03122
D1_sm	1	-3.84469	3.08164	-1.25	0.2123	-0.02972	1.01451
D2_sm	1	-1.75442	3.00834	-0.58	0.5598	-0.01401	1.03203

Linear Regression Results

The REG Procedure
Model: Linear_Regression_Model
Dependent Variable: HDL

Number of Observations Read	1701
Number of Observations Used	1701

Analysis of Variance					
Source	DF	Sum of Squares	Mean Square	F Value	Pr > F
Model	8	27239	3404.81716	28.51	<.0001
Error	1692	202034	119.40546		
Corrected Total	1700	229273			

Root MSE	10.92728	R-Square	0.1188
Dependent Mean	50.72310	Adj R-Sq	0.1146
Coeff Var	21.54300		

Parameter Estimates							
Variable	DF	Parameter Estimate	Standard Error	t Value	Pr > |t|	Standardized Estimate	Variance Inflation
Intercept	1	80.77935	3.58516	22.53	<.0001	0	0
N4	1	-0.27719	0.12278	-2.26	0.0241	-0.05192	1.01548
age	1	-0.13234	0.05928	-2.23	0.0257	-0.05180	1.03368
BMI	1	-1.14292	0.08363	-13.67	<.0001	-0.33468	1.15142
SBP	1	0.00990	0.02954	0.33	0.7377	0.00818	1.14422
D1_al	1	-1.63092	0.72914	-2.24	0.0254	-0.05209	1.04147
D2_al	1	-0.78255	0.88117	-0.89	0.3746	-0.02058	1.03122
D1_sm	1	-0.02370	1.23168	-0.02	0.9846	-0.00044237	1.01451
D2_sm	1	0.29654	1.20238	0.25	0.8052	0.00572	1.03203

Linear Regression Results

The REG Procedure
Model: Linear_Regression_Model
Dependent Variable: LDL

Number of Observations Read	1701
Number of Observations Used	1701

Analysis of Variance					
Source	DF	Sum of Squares	Mean Square	F Value	Pr > F
Model	8	84601	10575	19.26	<.0001
Error	1692	928939	549.01837		
Corrected Total	1700	1013541			

Root MSE	23.43114	R-Square	0.0835
Dependent Mean	110.19089	Adj R-Sq	0.0791
Coeff Var	21.26414		

Parameter Estimates							
Variable	DF	Parameter Estimate	Standard Error	t Value	Pr > |t|	Standardized Estimate	Variance Inflation
Intercept	1	56.93526	7.68758	7.41	<.0001	0	0
N4	1	-0.09231	0.26328	-0.35	0.7259	-0.00822	1.01548
age	1	1.10934	0.12711	8.73	<.0001	0.20651	1.03368
BMI	1	1.44957	0.17932	8.08	<.0001	0.20189	1.15142
SBP	1	-0.04304	0.06334	-0.68	0.4969	-0.01692	1.14422
D1_al	1	0.53562	1.56348	0.34	0.7320	0.00814	1.04147
D2_al	1	-0.02376	1.88948	-0.01	0.9900	-0.00029725	1.03122
D1_sm	1	-3.42698	2.64106	-1.30	0.1946	-0.03042	1.01451
D2_sm	1	-3.24405	2.57824	-1.26	0.2085	-0.02975	1.03203

Linear Regression Results

The REG Procedure
Model: Linear_Regression_Model
Dependent Variable: TG

Number of Observations Read	1701
Number of Observations Used	1701

Analysis of Variance					
Source	DF	Sum of Squares	Mean Square	F Value	Pr > F
Model	8	427719	53465	40.20	<.0001
Error	1692	2250298	1329.96349		
Corrected Total	1700	2678017			

Root MSE	36.46866	R-Square	0.1597
Dependent Mean	78.31805	Adj R-Sq	0.1557
Coeff Var	46.56483		

Parameter Estimates							
Variable	DF	Parameter Estimate	Standard Error	t Value	Pr > |t|	Standardized Estimate	Variance Inflation
Intercept	1	-50.43202	11.96509	-4.21	<.0001	0	0
N4	1	-0.00718	0.40978	-0.02	0.9860	-0.00039321	1.01548
age	1	0.44669	0.19784	2.26	0.0241	0.05116	1.03368
BMI	1	4.16527	0.27909	14.92	<.0001	0.35688	1.15142
SBP	1	0.23877	0.09859	2.42	0.0155	0.05773	1.14422
D1_al	1	6.82516	2.43343	2.80	0.0051	0.06379	1.04147
D2_al	1	5.39789	2.94082	1.84	0.0666	0.04154	1.03122
D1_sm	1	-1.88032	4.11060	-0.46	0.6474	-0.01027	1.01451
D2_sm	1	5.75538	4.01282	1.43	0.1517	0.03247	1.03203

Linear Regression Results

The REG Procedure
Model: Linear_Regression_Model
Dependent Variable: TC

Number of Observations Read	1701
Number of Observations Used	1701

Analysis of Variance					
Source	DF	Sum of Squares	Mean Square	F Value	Pr > F
Model	8	70686	8835.78677	11.81	<.0001
Error	1692	1265788	748.10173		
Corrected Total	1700	1336474			

Root MSE	27.35145	R-Square	0.0529
Dependent Mean	176.57202	Adj R-Sq	0.0484
Coeff Var	15.49025		

Parameter Estimates							
Variable	DF	Parameter Estimate	Standard Error	t Value	Pr > |t|	Standardized Estimate	Variance Inflation
Intercept	1	122.08559	9.02952	13.52	<.0001	0	0
N5	1	0.12788	0.31872	0.40	0.6883	0.00984	1.07504
age	1	1.10265	0.15058	7.32	<.0001	0.17875	1.06450
BMI	1	1.12841	0.21193	5.32	<.0001	0.13686	1.18035
SBP	1	0.01473	0.07406	0.20	0.8424	0.00504	1.14791
D1_al	1	0.14677	1.82559	0.08	0.9359	0.00194	1.04207
D2_al	1	0.19266	2.20608	0.09	0.9304	0.00210	1.03165
D1_sm	1	-3.74932	3.08231	-1.22	0.2240	-0.02898	1.01409
D2_sm	1	-1.73499	3.01641	-0.58	0.5652	-0.01386	1.03670

Linear Regression Results

The REG Procedure
Model: Linear_Regression_Model
Dependent Variable: HDL

Number of Observations Read	1701
Number of Observations Used	1701

Analysis of Variance					
Source	DF	Sum of Squares	Mean Square	F Value	Pr > F
Model	8	26636	3329.54579	27.80	<.0001
Error	1692	202636	119.76136		
Corrected Total	1700	229273			

Root MSE	10.94355	R-Square	0.1162
Dependent Mean	50.72310	Adj R-Sq	0.1120
Coeff Var	21.57509		

Parameter Estimates							
Variable	DF	Parameter Estimate	Standard Error	t Value	Pr > |t|	Standardized Estimate	Variance Inflation
Intercept	1	77.40795	3.61279	21.43	<.0001	0	0
N5	1	0.02938	0.12752	0.23	0.8178	0.00546	1.07504
age	1	-0.11467	0.06025	-1.90	0.0572	-0.04488	1.06450
BMI	1	-1.14702	0.08480	-13.53	<.0001	-0.33588	1.18035
SBP	1	0.00989	0.02963	0.33	0.7386	0.00817	1.14791
D1_al	1	-1.63955	0.73044	-2.24	0.0249	-0.05237	1.04207
D2_al	1	-0.81500	0.88267	-0.92	0.3560	-0.02143	1.03165
D1_sm	1	0.06008	1.23326	0.05	0.9612	0.00112	1.01409
D2_sm	1	0.35334	1.20689	0.29	0.7697	0.00681	1.03670

Linear Regression Results

The REG Procedure
Model: Linear_Regression_Model
Dependent Variable: LDL

Number of Observations Read	1701
Number of Observations Used	1701

Analysis of Variance					
Source	DF	Sum of Squares	Mean Square	F Value	Pr > F
Model	8	84964	10620	19.35	<.0001
Error	1692	928577	548.80422		
Corrected Total	1700	1013541			

Root MSE	23.42657	R-Square	0.0838
Dependent Mean	110.19089	Adj R-Sq	0.0795
Coeff Var	21.25999		

Parameter Estimates							
Variable	DF	Parameter Estimate	Standard Error	t Value	Pr > |t|	Standardized Estimate	Variance Inflation
Intercept	1	53.24998	7.73380	6.89	<.0001	0	0
N5	1	0.24159	0.27298	0.88	0.3763	0.02135	1.07504
age	1	1.13738	0.12897	8.82	<.0001	0.21173	1.06450
BMI	1	1.42406	0.18152	7.85	<.0001	0.19833	1.18035
SBP	1	-0.03997	0.06343	-0.63	0.5287	-0.01571	1.14791
D1_al	1	0.56618	1.56362	0.36	0.7173	0.00860	1.04207
D2_al	1	-0.07429	1.88951	-0.04	0.9686	-0.00092921	1.03165
D1_sm	1	-3.45668	2.64000	-1.31	0.1906	-0.03068	1.01409
D2_sm	1	-3.38490	2.58356	-1.31	0.1903	-0.03104	1.03670

Linear Regression Results

The REG Procedure
Model: Linear_Regression_Model
Dependent Variable: TG

Number of Observations Read	1701
Number of Observations Used	1701

Analysis of Variance					
Source	DF	Sum of Squares	Mean Square	F Value	Pr > F
Model	8	430339	53792	40.49	<.0001
Error	1692	2247678	1328.41470		
Corrected Total	1700	2678017			

Root MSE	36.44742	R-Square	0.1607
Dependent Mean	78.31805	Adj R-Sq	0.1567
Coeff Var	46.53771		

Parameter Estimates							
Variable	DF	Parameter Estimate	Standard Error	t Value	Pr > |t|	Standardized Estimate	Variance Inflation
Intercept	1	-43.91605	12.03236	-3.65	0.0003	0	0
N5	1	-0.59657	0.42471	-1.40	0.1603	-0.03244	1.07504
age	1	0.39005	0.20065	1.94	0.0521	0.04467	1.06450
BMI	1	4.22738	0.28241	14.97	<.0001	0.36220	1.18035
SBP	1	0.23085	0.09869	2.34	0.0194	0.05582	1.14791
D1_al	1	6.73878	2.43271	2.77	0.0057	0.06298	1.04207
D2_al	1	5.49939	2.93973	1.87	0.0616	0.04232	1.03165
D1_sm	1	-1.72973	4.10735	-0.42	0.6737	-0.00945	1.01409
D2_sm	1	6.16855	4.01954	1.53	0.1251	0.03480	1.03670

Linear Regression Results

The REG Procedure
Model: Linear_Regression_Model
Dependent Variable: TC

Number of Observations Read	1701
Number of Observations Used	1701

Analysis of Variance					
Source	DF	Sum of Squares	Mean Square	F Value	Pr > F
Model	8	72340	9042.46399	12.10	<.0001
Error	1692	1264135	747.12454		
Corrected Total	1700	1336474			

Root MSE	27.33358	R-Square	0.0541
Dependent Mean	176.57202	Adj R-Sq	0.0497
Coeff Var	15.48013		

Parameter Estimates							
Variable	DF	Parameter Estimate	Standard Error	t Value	Pr > |t|	Standardized Estimate	Variance Inflation
Intercept	1	128.62577	8.94971	14.37	<.0001	0	0
N6	1	-0.48653	0.31576	-1.54	0.1235	-0.03673	1.01632
age	1	1.06381	0.14838	7.17	<.0001	0.17245	1.03496
BMI	1	1.14329	0.20918	5.47	<.0001	0.13866	1.15141
SBP	1	0.01469	0.07390	0.20	0.8424	0.00503	1.14442
D1_al	1	0.07835	1.82411	0.04	0.9657	0.00104	1.04173
D2_al	1	0.36502	2.20612	0.17	0.8686	0.00398	1.03305
D1_sm	1	-3.81036	3.07986	-1.24	0.2162	-0.02945	1.01381
D2_sm	1	-1.65687	3.00644	-0.55	0.5816	-0.01323	1.03121

Linear Regression Results

The REG Procedure
Model: Linear_Regression_Model
Dependent Variable: HDL

Number of Observations Read	1701
Number of Observations Used	1701

Analysis of Variance					
Source	DF	Sum of Squares	Mean Square	F Value	Pr > F
Model	8	27565	3445.66528	28.90	<.0001
Error	1692	201707	119.21233		
Corrected Total	1700	229273			

Root MSE	10.91844	R-Square	0.1202
Dependent Mean	50.72310	Adj R-Sq	0.1161
Coeff Var	21.52558		

Parameter Estimates							
Variable	DF	Parameter Estimate	Standard Error	t Value	Pr > |t|	Standardized Estimate	Variance Inflation
Intercept	1	81.45528	3.57497	22.78	<.0001	0	0
N6	1	-0.35329	0.12613	-2.80	0.0052	-0.06439	1.01632
age	1	-0.13681	0.05927	-2.31	0.0211	-0.05355	1.03496
BMI	1	-1.14283	0.08356	-13.68	<.0001	-0.33465	1.15141
SBP	1	0.01070	0.02952	0.36	0.7169	0.00885	1.14442
D1_al	1	-1.68008	0.72864	-2.31	0.0212	-0.05366	1.04173
D2_al	1	-0.70072	0.88124	-0.80	0.4266	-0.01843	1.03305
D1_sm	1	-0.00002087	1.23026	-0.00	1.0000	-3.89478E-7	1.01381
D2_sm	1	0.36631	1.20093	0.31	0.7604	0.00706	1.03121

Linear Regression Results

The REG Procedure
Model: Linear_Regression_Model
Dependent Variable: LDL

Number of Observations Read	1701
Number of Observations Used	1701

Analysis of Variance					
Source	DF	Sum of Squares	Mean Square	F Value	Pr > F
Model	8	84583	10573	19.26	<.0001
Error	1692	928957	549.02900		
Corrected Total	1700	1013541			

Root MSE	23.43137	R-Square	0.0835
Dependent Mean	110.19089	Adj R-Sq	0.0791
Coeff Var	21.26434		

Parameter Estimates							
Variable	DF	Parameter Estimate	Standard Error	t Value	Pr > |t|	Standardized Estimate	Variance Inflation
Intercept	1	56.77708	7.67202	7.40	<.0001	0	0
N6	1	-0.08128	0.27068	-0.30	0.7640	-0.00705	1.01632
age	1	1.10984	0.12719	8.73	<.0001	0.20660	1.03496
BMI	1	1.44948	0.17932	8.08	<.0001	0.20187	1.15141
SBP	1	-0.04290	0.06335	-0.68	0.4984	-0.01686	1.14442
D1_al	1	0.52299	1.56370	0.33	0.7381	0.00795	1.04173
D2_al	1	-0.00776	1.89117	-0.00	0.9967	-0.00009709	1.03305
D1_sm	1	-3.41216	2.64017	-1.29	0.1964	-0.03029	1.01381
D2_sm	1	-3.22007	2.57723	-1.25	0.2117	-0.02953	1.03121

Linear Regression Results

The REG Procedure
Model: Linear_Regression_Model
Dependent Variable: TG

Number of Observations Read	1701
Number of Observations Used	1701

Analysis of Variance					
Source	DF	Sum of Squares	Mean Square	F Value	Pr > F
Model	8	428049	53506	40.24	<.0001
Error	1692	2249968	1329.76815		
Corrected Total	1700	2678017			

Root MSE	36.46599	R-Square	0.1598
Dependent Mean	78.31805	Adj R-Sq	0.1559
Coeff Var	46.56141		

Parameter Estimates							
Variable	DF	Parameter Estimate	Standard Error	t Value	Pr > |t|	Standardized Estimate	Variance Inflation
Intercept	1	-48.29662	11.93989	-4.04	<.0001	0	0
N6	1	-0.21015	0.42125	-0.50	0.6179	-0.01121	1.01632
age	1	0.43558	0.19795	2.20	0.0279	0.04988	1.03496
BMI	1	4.16591	0.27907	14.93	<.0001	0.35694	1.15141
SBP	1	0.23947	0.09859	2.43	0.0152	0.05790	1.14442
D1_al	1	6.80324	2.43356	2.80	0.0052	0.06358	1.04173
D2_al	1	5.46216	2.94321	1.86	0.0636	0.04203	1.03305
D1_sm	1	-1.91805	4.10888	-0.47	0.6407	-0.01047	1.01381
D2_sm	1	5.75305	4.01092	1.43	0.1517	0.03246	1.03121

Linear Regression Results

The REG Procedure
Model: Linear_Regression_Model
Dependent Variable: TC

Number of Observations Read	1701
Number of Observations Used	1701

Analysis of Variance					
Source	DF	Sum of Squares	Mean Square	F Value	Pr > F
Model	8	71020	8877.44998	11.87	<.0001
Error	1692	1265455	747.90474		
Corrected Total	1700	1336474			

Root MSE	27.34785	R-Square	0.0531
Dependent Mean	176.57202	Adj R-Sq	0.0487
Coeff Var	15.48821		

Parameter Estimates							
Variable	DF	Parameter Estimate	Standard Error	t Value	Pr > |t|	Standardized Estimate	Variance Inflation
Intercept	1	126.30986	9.06207	13.94	<.0001	0	0
E1	1	-0.25256	0.32425	-0.78	0.4362	-0.01851	1.00916
age	1	1.08264	0.14778	7.33	<.0001	0.17551	1.02562
BMI	1	1.15021	0.20957	5.49	<.0001	0.13950	1.15449
SBP	1	0.01305	0.07393	0.18	0.8599	0.00447	1.14418
D1_al	1	0.09415	1.82530	0.05	0.9589	0.00125	1.04201
D2_al	1	0.16029	2.20621	0.07	0.9421	0.00175	1.03205
D1_sm	1	-3.71338	3.08089	-1.21	0.2283	-0.02870	1.01342
D2_sm	1	-1.61110	3.00835	-0.54	0.5923	-0.01287	1.03144

Linear Regression Results

The REG Procedure
Model: Linear_Regression_Model
Dependent Variable: HDL

Number of Observations Read	1701
Number of Observations Used	1701

Analysis of Variance					
Source	DF	Sum of Squares	Mean Square	F Value	Pr > F
Model	8	26907	3363.43710	28.12	<.0001
Error	1692	202365	119.60111		
Corrected Total	1700	229273			

Root MSE	10.93623	R-Square	0.1174
Dependent Mean	50.72310	Adj R-Sq	0.1132
Coeff Var	21.56065		

Parameter Estimates							
Variable	DF	Parameter Estimate	Standard Error	t Value	Pr > |t|	Standardized Estimate	Variance Inflation
Intercept	1	75.53473	3.62386	20.84	<.0001	0	0
E1	1	0.19751	0.12967	1.52	0.1279	0.03495	1.00916
age	1	-0.11139	0.05910	-1.88	0.0596	-0.04360	1.02562
BMI	1	-1.15059	0.08381	-13.73	<.0001	-0.33692	1.15449
SBP	1	0.00949	0.02956	0.32	0.7483	0.00784	1.14418
D1_al	1	-1.61706	0.72993	-2.22	0.0269	-0.05165	1.04201
D2_al	1	-0.76751	0.88225	-0.87	0.3845	-0.02019	1.03205
D1_sm	1	0.06412	1.23203	0.05	0.9585	0.00120	1.01342
D2_sm	1	0.34562	1.20302	0.29	0.7739	0.00666	1.03144

Linear Regression Results

The REG Procedure
Model: Linear_Regression_Model
Dependent Variable: LDL

Number of Observations Read	1701
Number of Observations Used	1701

Analysis of Variance					
Source	DF	Sum of Squares	Mean Square	F Value	Pr > F
Model	8	85176	10647	19.40	<.0001
Error	1692	928365	548.67881		
Corrected Total	1700	1013541			

Root MSE	23.42389	R-Square	0.0840
Dependent Mean	110.19089	Adj R-Sq	0.0797
Coeff Var	21.25756		

Parameter Estimates							
Variable	DF	Parameter Estimate	Standard Error	t Value	Pr > |t|	Standardized Estimate	Variance Inflation
Intercept	1	59.26398	7.76182	7.64	<.0001	0	0
E1	1	-0.30042	0.27773	-1.08	0.2795	-0.02528	1.00916
age	1	1.10502	0.12658	8.73	<.0001	0.20570	1.02562
BMI	1	1.45930	0.17950	8.13	<.0001	0.20324	1.15449
SBP	1	-0.04316	0.06332	-0.68	0.4956	-0.01696	1.14418
D1_al	1	0.49068	1.56340	0.31	0.7537	0.00745	1.04201
D2_al	1	-0.09747	1.88966	-0.05	0.9589	-0.00122	1.03205
D1_sm	1	-3.39170	2.63883	-1.29	0.1989	-0.03011	1.01342
D2_sm	1	-3.17586	2.57670	-1.23	0.2179	-0.02912	1.03144

Linear Regression Results

The REG Procedure
Model: Linear_Regression_Model
Dependent Variable: TG

Number of Observations Read	1701
Number of Observations Used	1701

Analysis of Variance					
Source	DF	Sum of Squares	Mean Square	F Value	Pr > F
Model	8	432019	54002	40.68	<.0001
Error	1692	2245998	1327.42191		
Corrected Total	1700	2678017			

Root MSE	36.43380	R-Square	0.1613
Dependent Mean	78.31805	Adj R-Sq	0.1574
Coeff Var	46.52031		

Parameter Estimates							
Variable	DF	Parameter Estimate	Standard Error	t Value	Pr > |t|	Standardized Estimate	Variance Inflation
Intercept	1	-41.85773	12.07282	-3.47	0.0005	0	0
E1	1	-0.77756	0.43198	-1.80	0.0720	-0.04026	1.00916
age	1	0.42310	0.19688	2.15	0.0318	0.04845	1.02562
BMI	1	4.19133	0.27920	15.01	<.0001	0.35911	1.15449
SBP	1	0.23880	0.09849	2.42	0.0154	0.05774	1.14418
D1_al	1	6.71960	2.43173	2.76	0.0058	0.06280	1.04201
D2_al	1	5.23004	2.93919	1.78	0.0754	0.04025	1.03205
D1_sm	1	-1.86514	4.10447	-0.45	0.6496	-0.01018	1.01342
D2_sm	1	5.86746	4.00783	1.46	0.1434	0.03310	1.03144

Linear Regression Results

The REG Procedure
Model: Linear_Regression_Model
Dependent Variable: TC

Number of Observations Read	1701
Number of Observations Used	1701

Analysis of Variance					
Source	DF	Sum of Squares	Mean Square	F Value	Pr > F
Model	8	70763	8845.35849	11.82	<.0001
Error	1692	1265712	748.05648		
Corrected Total	1700	1336474			

Root MSE	27.35062	R-Square	0.0529
Dependent Mean	176.57202	Adj R-Sq	0.0485
Coeff Var	15.48978		

Parameter Estimates							
Variable	DF	Parameter Estimate	Standard Error	t Value	Pr > |t|	Standardized Estimate	Variance Inflation
Intercept	1	125.10778	8.88475	14.08	<.0001	0	0
E2	1	-0.15953	0.31086	-0.51	0.6079	-0.01221	1.01216
age	1	1.08314	0.14814	7.31	<.0001	0.17559	1.03041
BMI	1	1.14114	0.20931	5.45	<.0001	0.13840	1.15142
SBP	1	0.01384	0.07396	0.19	0.8516	0.00474	1.14469
D1_al	1	0.09124	1.82639	0.05	0.9602	0.00121	1.04304
D2_al	1	0.17107	2.20696	0.08	0.9382	0.00186	1.03255
D1_sm	1	-3.71039	3.08123	-1.20	0.2287	-0.02868	1.01344
D2_sm	1	-1.62406	3.00863	-0.54	0.5894	-0.01297	1.03143

Linear Regression Results

The REG Procedure
Model: Linear_Regression_Model
Dependent Variable: HDL

Number of Observations Read	1701
Number of Observations Used	1701

Analysis of Variance					
Source	DF	Sum of Squares	Mean Square	F Value	Pr > F
Model	8	26675	3334.41990	27.85	<.0001
Error	1692	202597	119.73831		
Corrected Total	1700	229273			

Root MSE	10.94250	R-Square	0.1163
Dependent Mean	50.72310	Adj R-Sq	0.1122
Coeff Var	21.57301		

Parameter Estimates							
Variable	DF	Parameter Estimate	Standard Error	t Value	Pr > |t|	Standardized Estimate	Variance Inflation
Intercept	1	76.96096	3.55463	21.65	<.0001	0	0
E2	1	0.07654	0.12437	0.62	0.5384	0.01415	1.01216
age	1	-0.11398	0.05927	-1.92	0.0546	-0.04461	1.03041
BMI	1	-1.14368	0.08374	-13.66	<.0001	-0.33490	1.15142
SBP	1	0.00912	0.02959	0.31	0.7581	0.00753	1.14469
D1_al	1	-1.62599	0.73071	-2.23	0.0262	-0.05194	1.04304
D2_al	1	-0.78910	0.88297	-0.89	0.3716	-0.02075	1.03255
D1_sm	1	0.06394	1.23274	0.05	0.9586	0.00119	1.01344
D2_sm	1	0.36265	1.20370	0.30	0.7632	0.00699	1.03143

Linear Regression Results

The REG Procedure
Model: Linear_Regression_Model
Dependent Variable: LDL

Number of Observations Read	1701
Number of Observations Used	1701

Analysis of Variance					
Source	DF	Sum of Squares	Mean Square	F Value	Pr > F
Model	8	84726	10591	19.29	<.0001
Error	1692	928814	548.94455		
Corrected Total	1700	1013541			

Root MSE	23.42957	R-Square	0.0836
Dependent Mean	110.19089	Adj R-Sq	0.0793
Coeff Var	21.26271		

Parameter Estimates							
Variable	DF	Parameter Estimate	Standard Error	t Value	Pr > |t|	Standardized Estimate	Variance Inflation
Intercept	1	57.51024	7.61101	7.56	<.0001	0	0
E2	1	-0.15765	0.26630	-0.59	0.5539	-0.01386	1.01216
age	1	1.10708	0.12691	8.72	<.0001	0.20609	1.03041
BMI	1	1.44864	0.17931	8.08	<.0001	0.20176	1.15142
SBP	1	-0.04238	0.06335	-0.67	0.5036	-0.01666	1.14469
D1_al	1	0.49469	1.56455	0.32	0.7519	0.00752	1.04304
D2_al	1	-0.07588	1.89057	-0.04	0.9680	-0.00094920	1.03255
D1_sm	1	-3.38958	2.63949	-1.28	0.1993	-0.03009	1.01344
D2_sm	1	-3.19587	2.57731	-1.24	0.2151	-0.02931	1.03143

Linear Regression Results

The REG Procedure
Model: Linear_Regression_Model
Dependent Variable: TG

Number of Observations Read	1701
Number of Observations Used	1701

Analysis of Variance					
Source	DF	Sum of Squares	Mean Square	F Value	Pr > F
Model	8	429302	53663	40.38	<.0001
Error	1692	2248714	1329.02747		
Corrected Total	1700	2678017			

Root MSE	36.45583	R-Square	0.1603
Dependent Mean	78.31805	Adj R-Sq	0.1563
Coeff Var	46.54844		

Parameter Estimates							
Variable	DF	Parameter Estimate	Standard Error	t Value	Pr > |t|	Standardized Estimate	Variance Inflation
Intercept	1	-45.94955	11.84254	-3.88	0.0001	0	0
E2	1	-0.45238	0.41435	-1.09	0.2751	-0.02447	1.01216
age	1	0.42639	0.19746	2.16	0.0310	0.04883	1.03041
BMI	1	4.16356	0.27900	14.92	<.0001	0.35673	1.15142
SBP	1	0.24103	0.09858	2.45	0.0146	0.05828	1.14469
D1_al	1	6.71965	2.43441	2.76	0.0058	0.06280	1.04304
D2_al	1	5.27381	2.94167	1.79	0.0732	0.04058	1.03255
D1_sm	1	-1.85766	4.10699	-0.45	0.6511	-0.01014	1.01344
D2_sm	1	5.82201	4.01023	1.45	0.1467	0.03285	1.03143

Linear Regression Results

The REG Procedure
Model: Linear_Regression_Model
Dependent Variable: TC

Number of Observations Read	1701
Number of Observations Used	1701

Analysis of Variance					
Source	DF	Sum of Squares	Mean Square	F Value	Pr > F
Model	8	71061	8882.57241	11.88	<.0001
Error	1692	1265414	747.88053		
Corrected Total	1700	1336474			

Root MSE	27.34740	R-Square	0.0532
Dependent Mean	176.57202	Adj R-Sq	0.0487
Coeff Var	15.48796		

Parameter Estimates							
Variable	DF	Parameter Estimate	Standard Error	t Value	Pr > |t|	Standardized Estimate	Variance Inflation
Intercept	1	121.21345	8.77475	13.81	<.0001	0	0
E3	1	0.22869	0.28118	0.81	0.4161	0.01934	1.01069
age	1	1.09871	0.14779	7.43	<.0001	0.17811	1.02579
BMI	1	1.13293	0.20957	5.41	<.0001	0.13741	1.15446
SBP	1	0.01287	0.07393	0.17	0.8618	0.00441	1.14419
D1_al	1	0.17921	1.82582	0.10	0.9218	0.00237	1.04263
D2_al	1	0.27308	2.20625	0.12	0.9015	0.00297	1.03212
D1_sm	1	-3.80597	3.08275	-1.23	0.2171	-0.02942	1.01468
D2_sm	1	-1.67488	3.00815	-0.56	0.5778	-0.01338	1.03134

Linear Regression Results

The REG Procedure
Model: Linear_Regression_Model
Dependent Variable: HDL

Number of Observations Read	1701
Number of Observations Used	1701

Analysis of Variance					
Source	DF	Sum of Squares	Mean Square	F Value	Pr > F
Model	8	26630	3328.75135	27.79	<.0001
Error	1692	202643	119.76511		
Corrected Total	1700	229273			

Root MSE	10.94372	R-Square	0.1162
Dependent Mean	50.72310	Adj R-Sq	0.1120
Coeff Var	21.57542		

Parameter Estimates							
Variable	DF	Parameter Estimate	Standard Error	t Value	Pr > |t|	Standardized Estimate	Variance Inflation
Intercept	1	77.73095	3.51143	22.14	<.0001	0	0
E3	1	0.00017544	0.11252	0.00	0.9988	0.00003583	1.01069
age	1	-0.11747	0.05914	-1.99	0.0472	-0.04598	1.02579
BMI	1	-1.14397	0.08386	-13.64	<.0001	-0.33498	1.15446
SBP	1	0.00950	0.02959	0.32	0.7481	0.00785	1.14419
D1_al	1	-1.64375	0.73064	-2.25	0.0246	-0.05250	1.04263
D2_al	1	-0.80992	0.88288	-0.92	0.3591	-0.02130	1.03212
D1_sm	1	0.06731	1.23364	0.05	0.9565	0.00126	1.01468
D2_sm	1	0.37356	1.20378	0.31	0.7564	0.00720	1.03134

Linear Regression Results

The REG Procedure
Model: Linear_Regression_Model
Dependent Variable: LDL

Number of Observations Read	1701
Number of Observations Used	1701

Analysis of Variance					
Source	DF	Sum of Squares	Mean Square	F Value	Pr > F
Model	8	85377	10672	19.45	<.0001
Error	1692	928163	548.55970		
Corrected Total	1700	1013541			

Root MSE	23.42135	R-Square	0.0842
Dependent Mean	110.19089	Adj R-Sq	0.0799
Coeff Var	21.25525		

Parameter Estimates							
Variable	DF	Parameter Estimate	Standard Error	t Value	Pr > |t|	Standardized Estimate	Variance Inflation
Intercept	1	52.93588	7.51503	7.04	<.0001	0	0
E3	1	0.29862	0.24081	1.24	0.2151	0.02900	1.01069
age	1	1.12509	0.12658	8.89	<.0001	0.20944	1.02579
BMI	1	1.43773	0.17948	8.01	<.0001	0.20024	1.15446
SBP	1	-0.04339	0.06332	-0.69	0.4933	-0.01705	1.14419
D1_al	1	0.59778	1.56370	0.38	0.7023	0.00908	1.04263
D2_al	1	0.04350	1.88951	0.02	0.9816	0.00054417	1.03212
D1_sm	1	-3.51212	2.64018	-1.33	0.1836	-0.03117	1.01468
D2_sm	1	-3.25499	2.57629	-1.26	0.2066	-0.02985	1.03134

Linear Regression Results

The REG Procedure
Model: Linear_Regression_Model
Dependent Variable: TG

Number of Observations Read	1701
Number of Observations Used	1701

Analysis of Variance					
Source	DF	Sum of Squares	Mean Square	F Value	Pr > F
Model	8	429200	53650	40.37	<.0001
Error	1692	2248817	1329.08805		
Corrected Total	1700	2678017			

Root MSE	36.45666	R-Square	0.1603
Dependent Mean	78.31805	Adj R-Sq	0.1563
Coeff Var	46.54950		

Parameter Estimates							
Variable	DF	Parameter Estimate	Standard Error	t Value	Pr > |t|	Standardized Estimate	Variance Inflation
Intercept	1	-46.55511	11.69757	-3.98	<.0001	0	0
E3	1	-0.39576	0.37483	-1.06	0.2912	-0.02365	1.01069
age	1	0.43275	0.19702	2.20	0.0282	0.04956	1.02579
BMI	1	4.18047	0.27937	14.96	<.0001	0.35818	1.15446
SBP	1	0.23903	0.09856	2.43	0.0154	0.05780	1.14419
D1_al	1	6.73677	2.43398	2.77	0.0057	0.06296	1.04263
D2_al	1	5.29592	2.94113	1.80	0.0719	0.04075	1.03212
D1_sm	1	-1.72494	4.10960	-0.42	0.6747	-0.00942	1.01468
D2_sm	1	5.80588	4.01015	1.45	0.1479	0.03276	1.03134

Linear Regression Results

The REG Procedure
Model: Linear_Regression_Model
Dependent Variable: TC

Number of Observations Read	1701
Number of Observations Used	1701

Analysis of Variance					
Source	DF	Sum of Squares	Mean Square	F Value	Pr > F
Model	8	70681	8835.12626	11.81	<.0001
Error	1692	1265793	748.10486		
Corrected Total	1700	1336474			

Root MSE	27.35151	R-Square	0.0529
Dependent Mean	176.57202	Adj R-Sq	0.0484
Coeff Var	15.49028		

Parameter Estimates							
Variable	DF	Parameter Estimate	Standard Error	t Value	Pr > |t|	Standardized Estimate	Variance Inflation
Intercept	1	122.30181	8.85630	13.81	<.0001	0	0
E4	1	0.11483	0.29268	0.39	0.6949	0.00930	1.00398
age	1	1.09286	0.14759	7.40	<.0001	0.17716	1.02273
BMI	1	1.14240	0.20932	5.46	<.0001	0.13856	1.15146
SBP	1	0.01342	0.07395	0.18	0.8560	0.00459	1.14438
D1_al	1	0.14311	1.82541	0.08	0.9375	0.00189	1.04185
D2_al	1	0.23193	2.20585	0.11	0.9163	0.00253	1.03144
D1_sm	1	-3.69789	3.08170	-1.20	0.2303	-0.02858	1.01369
D2_sm	1	-1.67981	3.00958	-0.56	0.5768	-0.01342	1.03200

Linear Regression Results

The REG Procedure
Model: Linear_Regression_Model
Dependent Variable: HDL

Number of Observations Read	1701
Number of Observations Used	1701

Analysis of Variance					
Source	DF	Sum of Squares	Mean Square	F Value	Pr > F
Model	8	27385	3423.09974	28.69	<.0001
Error	1692	201888	119.31902		
Corrected Total	1700	229273			

Root MSE	10.92332	R-Square	0.1194
Dependent Mean	50.72310	Adj R-Sq	0.1153
Coeff Var	21.53521		

Parameter Estimates							
Variable	DF	Parameter Estimate	Standard Error	t Value	Pr > |t|	Standardized Estimate	Variance Inflation
Intercept	1	74.66696	3.53693	21.11	<.0001	0	0
E4	1	0.29399	0.11689	2.52	0.0120	0.05749	1.00398
age	1	-0.11125	0.05894	-1.89	0.0593	-0.04354	1.02273
BMI	1	-1.14225	0.08360	-13.66	<.0001	-0.33448	1.15146
SBP	1	0.01049	0.02953	0.36	0.7224	0.00867	1.14438
D1_al	1	-1.60593	0.72901	-2.20	0.0277	-0.05129	1.04185
D2_al	1	-0.76553	0.88095	-0.87	0.3850	-0.02013	1.03144
D1_sm	1	0.11770	1.23073	0.10	0.9238	0.00220	1.01369
D2_sm	1	0.28921	1.20193	0.24	0.8099	0.00558	1.03200

Linear Regression Results

The REG Procedure
Model: Linear_Regression_Model
Dependent Variable: LDL

Number of Observations Read	1701
Number of Observations Used	1701

Analysis of Variance					
Source	DF	Sum of Squares	Mean Square	F Value	Pr > F
Model	8	84535	10567	19.25	<.0001
Error	1692	929006	549.05776		
Corrected Total	1700	1013541			

Root MSE	23.43198	R-Square	0.0834
Dependent Mean	110.19089	Adj R-Sq	0.0791
Coeff Var	21.26490		

Parameter Estimates							
Variable	DF	Parameter Estimate	Standard Error	t Value	Pr > |t|	Standardized Estimate	Variance Inflation
Intercept	1	56.02271	7.58718	7.38	<.0001	0	0
E4	1	-0.00978	0.25074	-0.04	0.9689	-0.00091003	1.00398
age	1	1.11408	0.12644	8.81	<.0001	0.20739	1.02273
BMI	1	1.44917	0.17933	8.08	<.0001	0.20183	1.15146
SBP	1	-0.04321	0.06335	-0.68	0.4953	-0.01698	1.14438
D1_al	1	0.53008	1.56382	0.34	0.7347	0.00805	1.04185
D2_al	1	-0.03437	1.88975	-0.02	0.9855	-0.00042996	1.03144
D1_sm	1	-3.39833	2.64009	-1.29	0.1982	-0.03016	1.01369
D2_sm	1	-3.21559	2.57830	-1.25	0.2125	-0.02949	1.03200

Linear Regression Results

The REG Procedure
Model: Linear_Regression_Model
Dependent Variable: TG

Number of Observations Read	1701
Number of Observations Used	1701

Analysis of Variance					
Source	DF	Sum of Squares	Mean Square	F Value	Pr > F
Model	8	434191	54274	40.93	<.0001
Error	1692	2243826	1326.13848		
Corrected Total	1700	2678017			

Root MSE	36.41618	R-Square	0.1621
Dependent Mean	78.31805	Adj R-Sq	0.1582
Coeff Var	46.49782		

Parameter Estimates							
Variable	DF	Parameter Estimate	Standard Error	t Value	Pr > |t|	Standardized Estimate	Variance Inflation
Intercept	1	-41.53341	11.79141	-3.52	0.0004	0	0
E4	1	-0.86089	0.38968	-2.21	0.0273	-0.04926	1.00398
age	1	0.42885	0.19651	2.18	0.0292	0.04911	1.02273
BMI	1	4.16022	0.27870	14.93	<.0001	0.35645	1.15146
SBP	1	0.23586	0.09846	2.40	0.0167	0.05703	1.14438
D1_al	1	6.71396	2.43037	2.76	0.0058	0.06275	1.04185
D2_al	1	5.26705	2.93690	1.79	0.0731	0.04053	1.03144
D1_sm	1	-2.02533	4.10302	-0.49	0.6216	-0.01106	1.01369
D2_sm	1	6.00444	4.00699	1.50	0.1342	0.03388	1.03200

Linear Regression Results

The REG Procedure
Model: Linear_Regression_Model
Dependent Variable: TC

Number of Observations Read	1701
Number of Observations Used	1701

Analysis of Variance					
Source	DF	Sum of Squares	Mean Square	F Value	Pr > F
Model	8	71375	8921.82695	11.93	<.0001
Error	1692	1265100	747.69492		
Corrected Total	1700	1336474			

Root MSE	27.34401	R-Square	0.0534
Dependent Mean	176.57202	Adj R-Sq	0.0489
Coeff Var	15.48604		

Parameter Estimates							
Variable	DF	Parameter Estimate	Standard Error	t Value	Pr > |t|	Standardized Estimate	Variance Inflation
Intercept	1	126.94533	8.94760	14.19	<.0001	0	0
E5	1	-0.29403	0.28272	-1.04	0.2985	-0.02470	1.00825
age	1	1.08104	0.14770	7.32	<.0001	0.17525	1.02476
BMI	1	1.13864	0.20928	5.44	<.0001	0.13810	1.15162
SBP	1	0.01278	0.07392	0.17	0.8627	0.00437	1.14419
D1_al	1	0.04814	1.82615	0.03	0.9790	0.00063693	1.04326
D2_al	1	0.13167	2.20624	0.06	0.9524	0.00143	1.03237
D1_sm	1	-3.67247	3.08075	-1.19	0.2334	-0.02839	1.01362
D2_sm	1	-1.52879	3.00972	-0.51	0.6116	-0.01221	1.03267

Linear Regression Results

The REG Procedure
Model: Linear_Regression_Model
Dependent Variable: HDL

Number of Observations Read	1701
Number of Observations Used	1701

Analysis of Variance					
Source	DF	Sum of Squares	Mean Square	F Value	Pr > F
Model	8	26632	3328.97190	27.80	<.0001
Error	1692	202641	119.76407		
Corrected Total	1700	229273			

Root MSE	10.94368	R-Square	0.1162
Dependent Mean	50.72310	Adj R-Sq	0.1120
Coeff Var	21.57533		

Parameter Estimates							
Variable	DF	Parameter Estimate	Standard Error	t Value	Pr > |t|	Standardized Estimate	Variance Inflation
Intercept	1	77.89368	3.58103	21.75	<.0001	0	0
E5	1	-0.01373	0.11315	-0.12	0.9034	-0.00279	1.00825
age	1	-0.11791	0.05911	-1.99	0.0462	-0.04615	1.02476
BMI	1	-1.14411	0.08376	-13.66	<.0001	-0.33503	1.15162
SBP	1	0.00949	0.02959	0.32	0.7484	0.00784	1.14419
D1_al	1	-1.64753	0.73086	-2.25	0.0243	-0.05262	1.04326
D2_al	1	-0.81384	0.88299	-0.92	0.3568	-0.02140	1.03237
D1_sm	1	0.06948	1.23299	0.06	0.9551	0.00130	1.01362
D2_sm	1	0.37910	1.20456	0.31	0.7530	0.00731	1.03267

Linear Regression Results

The REG Procedure
Model: Linear_Regression_Model
Dependent Variable: LDL

Number of Observations Read	1701
Number of Observations Used	1701

Analysis of Variance					
Source	DF	Sum of Squares	Mean Square	F Value	Pr > F
Model	8	85248	10656	19.42	<.0001
Error	1692	928292	548.63599		
Corrected Total	1700	1013541			

Root MSE	23.42298	R-Square	0.0841
Dependent Mean	110.19089	Adj R-Sq	0.0798
Coeff Var	21.25673		

Parameter Estimates							
Variable	DF	Parameter Estimate	Standard Error	t Value	Pr > |t|	Standardized Estimate	Variance Inflation
Intercept	1	59.15969	7.66455	7.72	<.0001	0	0
E5	1	-0.27637	0.24218	-1.14	0.2540	-0.02666	1.00825
age	1	1.10546	0.12652	8.74	<.0001	0.20578	1.02476
BMI	1	1.44631	0.17927	8.07	<.0001	0.20143	1.15162
SBP	1	-0.04341	0.06332	-0.69	0.4931	-0.01706	1.14419
D1_al	1	0.45597	1.56428	0.29	0.7707	0.00693	1.04326
D2_al	1	-0.11082	1.88987	-0.06	0.9532	-0.00139	1.03237
D1_sm	1	-3.35429	2.63899	-1.27	0.2039	-0.02977	1.01362
D2_sm	1	-3.10742	2.57814	-1.21	0.2283	-0.02850	1.03267

Linear Regression Results

The REG Procedure
Model: Linear_Regression_Model
Dependent Variable: TG

Number of Observations Read	1701
Number of Observations Used	1701

Analysis of Variance					
Source	DF	Sum of Squares	Mean Square	F Value	Pr > F
Model	8	427803	53475	40.21	<.0001
Error	1692	2250214	1329.91394		
Corrected Total	1700	2678017			

Root MSE	36.46799	R-Square	0.1597
Dependent Mean	78.31805	Adj R-Sq	0.1558
Coeff Var	46.56396		

Parameter Estimates							
Variable	DF	Parameter Estimate	Standard Error	t Value	Pr > |t|	Standardized Estimate	Variance Inflation
Intercept	1	-49.39872	11.93318	-4.14	<.0001	0	0
E5	1	-0.09489	0.37705	-0.25	0.8013	-0.00563	1.00825
age	1	0.44404	0.19698	2.25	0.0243	0.05085	1.02476
BMI	1	4.16424	0.27911	14.92	<.0001	0.35679	1.15162
SBP	1	0.23867	0.09859	2.42	0.0156	0.05771	1.14419
D1_al	1	6.79895	2.43548	2.79	0.0053	0.06354	1.04326
D2_al	1	5.37042	2.94241	1.83	0.0682	0.04133	1.03237
D1_sm	1	-1.86342	4.10872	-0.45	0.6502	-0.01018	1.01362
D2_sm	1	5.79548	4.01398	1.44	0.1490	0.03270	1.03267

Linear Regression Results

The REG Procedure
Model: Linear_Regression_Model
Dependent Variable: TC

Number of Observations Read	1701
Number of Observations Used	1701

Analysis of Variance					
Source	DF	Sum of Squares	Mean Square	F Value	Pr > F
Model	8	70619	8827.36858	11.80	<.0001
Error	1692	1265855	748.14154		
Corrected Total	1700	1336474			

Root MSE	27.35218	R-Square	0.0528
Dependent Mean	176.57202	Adj R-Sq	0.0484
Coeff Var	15.49066		

Parameter Estimates							
Variable	DF	Parameter Estimate	Standard Error	t Value	Pr > |t|	Standardized Estimate	Variance Inflation
Intercept	1	124.38780	8.95819	13.89	<.0001	0	0
E6	1	-0.07797	0.29270	-0.27	0.7900	-0.00634	1.01332
age	1	1.08665	0.14815	7.33	<.0001	0.17616	1.03041
BMI	1	1.14207	0.20933	5.46	<.0001	0.13852	1.15142
SBP	1	0.01304	0.07394	0.18	0.8600	0.00446	1.14418
D1_al	1	0.13908	1.82551	0.08	0.9393	0.00184	1.04191
D2_al	1	0.19431	2.20677	0.09	0.9298	0.00212	1.03225
D1_sm	1	-3.73558	3.08211	-1.21	0.2257	-0.02887	1.01391
D2_sm	1	-1.63302	3.00893	-0.54	0.5874	-0.01304	1.03151

Linear Regression Results

The REG Procedure
Model: Linear_Regression_Model
Dependent Variable: HDL

Number of Observations Read	1701
Number of Observations Used	1701

Analysis of Variance					
Source	DF	Sum of Squares	Mean Square	F Value	Pr > F
Model	8	27043	3380.41830	28.28	<.0001
Error	1692	202229	119.52082		
Corrected Total	1700	229273			

Root MSE	10.93256	R-Square	0.1180
Dependent Mean	50.72310	Adj R-Sq	0.1138
Coeff Var	21.55341		

Parameter Estimates							
Variable	DF	Parameter Estimate	Standard Error	t Value	Pr > |t|	Standardized Estimate	Variance Inflation
Intercept	1	75.25325	3.58055	21.02	<.0001	0	0
E6	1	0.21756	0.11699	1.86	0.0631	0.04274	1.01332
age	1	-0.10691	0.05922	-1.81	0.0712	-0.04184	1.03041
BMI	1	-1.14489	0.08367	-13.68	<.0001	-0.33525	1.15142
SBP	1	0.00949	0.02955	0.32	0.7483	0.00784	1.14418
D1_al	1	-1.67379	0.72965	-2.29	0.0219	-0.05346	1.04191
D2_al	1	-0.75342	0.88204	-0.85	0.3931	-0.01981	1.03225
D1_sm	1	0.11770	1.23191	0.10	0.9239	0.00220	1.01391
D2_sm	1	0.33498	1.20266	0.28	0.7806	0.00646	1.03151

Linear Regression Results

The REG Procedure
Model: Linear_Regression_Model
Dependent Variable: LDL

Number of Observations Read	1701
Number of Observations Used	1701

Analysis of Variance					
Source	DF	Sum of Squares	Mean Square	F Value	Pr > F
Model	8	84658	10582	19.28	<.0001
Error	1692	928883	548.98495		
Corrected Total	1700	1013541			

Root MSE	23.43043	R-Square	0.0835
Dependent Mean	110.19089	Adj R-Sq	0.0792
Coeff Var	21.26349		

Parameter Estimates							
Variable	DF	Parameter Estimate	Standard Error	t Value	Pr > |t|	Standardized Estimate	Variance Inflation
Intercept	1	57.27894	7.67377	7.46	<.0001	0	0
E6	1	-0.11918	0.25073	-0.48	0.6346	-0.01114	1.01332
age	1	1.10850	0.12691	8.73	<.0001	0.20635	1.03041
BMI	1	1.44973	0.17931	8.08	<.0001	0.20191	1.15142
SBP	1	-0.04317	0.06334	-0.68	0.4957	-0.01697	1.14418
D1_al	1	0.54778	1.56377	0.35	0.7262	0.00832	1.04191
D2_al	1	-0.06387	1.89036	-0.03	0.9730	-0.00079897	1.03225
D1_sm	1	-3.42422	2.64020	-1.30	0.1948	-0.03039	1.01391
D2_sm	1	-3.19725	2.57751	-1.24	0.2150	-0.02932	1.03151

Linear Regression Results

The REG Procedure
Model: Linear_Regression_Model
Dependent Variable: TG

Number of Observations Read	1701
Number of Observations Used	1701

Analysis of Variance					
Source	DF	Sum of Squares	Mean Square	F Value	Pr > F
Model	8	435343	54418	41.06	<.0001
Error	1692	2242674	1325.45755		
Corrected Total	1700	2678017			

Root MSE	36.40683	R-Square	0.1626
Dependent Mean	78.31805	Adj R-Sq	0.1586
Coeff Var	46.48588		

Parameter Estimates							
Variable	DF	Parameter Estimate	Standard Error	t Value	Pr > |t|	Standardized Estimate	Variance Inflation
Intercept	1	-39.86188	11.92371	-3.34	0.0008	0	0
E6	1	-0.93441	0.38960	-2.40	0.0166	-0.05371	1.01332
age	1	0.40171	0.19720	2.04	0.0418	0.04600	1.03041
BMI	1	4.16923	0.27862	14.96	<.0001	0.35722	1.15142
SBP	1	0.23882	0.09842	2.43	0.0153	0.05774	1.14418
D1_al	1	6.95370	2.42982	2.86	0.0043	0.06499	1.04191
D2_al	1	5.15429	2.93730	1.75	0.0795	0.03966	1.03225
D1_sm	1	-2.09411	4.10242	-0.51	0.6098	-0.01143	1.01391
D2_sm	1	5.92319	4.00500	1.48	0.1393	0.03342	1.03151

Linear Regression Results

The REG Procedure
Model: Linear_Regression_Model
Dependent Variable: TC

Number of Observations Read	1701
Number of Observations Used	1701

Analysis of Variance					
Source	DF	Sum of Squares	Mean Square	F Value	Pr > F
Model	8	70661	8832.64578	11.81	<.0001
Error	1692	1265813	748.11658		
Corrected Total	1700	1336474			

Root MSE	27.35172	R-Square	0.0529
Dependent Mean	176.57202	Adj R-Sq	0.0484
Coeff Var	15.49040		

Parameter Estimates							
Variable	DF	Parameter Estimate	Standard Error	t Value	Pr > |t|	Standardized Estimate	Variance Inflation
Intercept	1	124.57614	8.84439	14.09	<.0001	0	0
O1	1	-0.09664	0.27075	-0.36	0.7212	-0.00850	1.01425
age	1	1.08603	0.14798	7.34	<.0001	0.17606	1.02808
BMI	1	1.14476	0.20949	5.46	<.0001	0.13884	1.15327
SBP	1	0.01336	0.07395	0.18	0.8566	0.00457	1.14435
D1_al	1	0.11505	1.82541	0.06	0.9498	0.00152	1.04184
D2_al	1	0.18460	2.20702	0.08	0.9333	0.00201	1.03252
D1_sm	1	-3.72775	3.08145	-1.21	0.2265	-0.02881	1.01351
D2_sm	1	-1.59229	3.01231	-0.53	0.5972	-0.01272	1.03386

Linear Regression Results

The REG Procedure
Model: Linear_Regression_Model
Dependent Variable: HDL

Number of Observations Read	1701
Number of Observations Used	1701

Analysis of Variance					
Source	DF	Sum of Squares	Mean Square	F Value	Pr > F
Model	8	26697	3337.09715	27.87	<.0001
Error	1692	202576	119.72565		
Corrected Total	1700	229273			

Root MSE	10.94192	R-Square	0.1164
Dependent Mean	50.72310	Adj R-Sq	0.1123
Coeff Var	21.57187		

Parameter Estimates							
Variable	DF	Parameter Estimate	Standard Error	t Value	Pr > |t|	Standardized Estimate	Variance Inflation
Intercept	1	76.83133	3.53816	21.72	<.0001	0	0
O1	1	0.08089	0.10831	0.75	0.4553	0.01719	1.01425
age	1	-0.11379	0.05920	-1.92	0.0548	-0.04454	1.02808
BMI	1	-1.14650	0.08381	-13.68	<.0001	-0.33572	1.15327
SBP	1	0.00923	0.02958	0.31	0.7551	0.00763	1.14435
D1_al	1	-1.63267	0.73025	-2.24	0.0255	-0.05215	1.04184
D2_al	1	-0.78488	0.88291	-0.89	0.3741	-0.02064	1.03252
D1_sm	1	0.07592	1.23272	0.06	0.9509	0.00142	1.01351
D2_sm	1	0.32792	1.20506	0.27	0.7856	0.00632	1.03386

Linear Regression Results

The REG Procedure
Model: Linear_Regression_Model
Dependent Variable: LDL

Number of Observations Read	1701
Number of Observations Used	1701

Analysis of Variance					
Source	DF	Sum of Squares	Mean Square	F Value	Pr > F
Model	8	84606	10576	19.26	<.0001
Error	1692	928934	549.01551		
Corrected Total	1700	1013541			

Root MSE	23.43108	R-Square	0.0835
Dependent Mean	110.19089	Adj R-Sq	0.0791
Coeff Var	21.26408		

Parameter Estimates							
Variable	DF	Parameter Estimate	Standard Error	t Value	Pr > |t|	Standardized Estimate	Variance Inflation
Intercept	1	56.85887	7.57662	7.50	<.0001	0	0
O1	1	-0.08419	0.23194	-0.36	0.7167	-0.00851	1.01425
age	1	1.11045	0.12677	8.76	<.0001	0.20671	1.02808
BMI	1	1.45186	0.17946	8.09	<.0001	0.20220	1.15327
SBP	1	-0.04289	0.06335	-0.68	0.4984	-0.01686	1.14435
D1_al	1	0.51977	1.56375	0.33	0.7396	0.00790	1.04184
D2_al	1	-0.05901	1.89066	-0.03	0.9751	-0.00073807	1.03252
D1_sm	1	-3.40554	2.63975	-1.29	0.1972	-0.03023	1.01351
D2_sm	1	-3.17086	2.58052	-1.23	0.2193	-0.02908	1.03386

Linear Regression Results

The REG Procedure
Model: Linear_Regression_Model
Dependent Variable: TG

Number of Observations Read	1701
Number of Observations Used	1701

Analysis of Variance					
Source	DF	Sum of Squares	Mean Square	F Value	Pr > F
Model	8	429614	53702	40.41	<.0001
Error	1692	2248403	1328.84312		
Corrected Total	1700	2678017			

Root MSE	36.45330	R-Square	0.1604
Dependent Mean	78.31805	Adj R-Sq	0.1565
Coeff Var	46.54521		

Parameter Estimates							
Variable	DF	Parameter Estimate	Standard Error	t Value	Pr > |t|	Standardized Estimate	Variance Inflation
Intercept	1	-45.70745	11.78746	-3.88	0.0001	0	0
O1	1	-0.43104	0.36085	-1.19	0.2324	-0.02680	1.01425
age	1	0.42743	0.19722	2.17	0.0304	0.04895	1.02808
BMI	1	4.17874	0.27920	14.97	<.0001	0.35803	1.15327
SBP	1	0.24020	0.09855	2.44	0.0149	0.05808	1.14435
D1_al	1	6.76559	2.43284	2.78	0.0055	0.06323	1.04184
D2_al	1	5.26349	2.94143	1.79	0.0737	0.04050	1.03252
D1_sm	1	-1.92348	4.10684	-0.47	0.6396	-0.01050	1.01351
D2_sm	1	6.00075	4.01469	1.49	0.1352	0.03385	1.03386

Linear Regression Results

The REG Procedure
Model: Linear_Regression_Model
Dependent Variable: TC

Number of Observations Read	1701
Number of Observations Used	1701

Analysis of Variance					
Source	DF	Sum of Squares	Mean Square	F Value	Pr > F
Model	8	71850	8981.30507	12.02	<.0001
Error	1692	1264624	747.41370		
Corrected Total	1700	1336474			

Root MSE	27.33887	R-Square	0.0538
Dependent Mean	176.57202	Adj R-Sq	0.0493
Coeff Var	15.48313		

Parameter Estimates							
Variable	DF	Parameter Estimate	Standard Error	t Value	Pr > |t|	Standardized Estimate	Variance Inflation
Intercept	1	127.32086	8.80637	14.46	<.0001	0	0
O2	1	-0.33922	0.25875	-1.31	0.1900	-0.03107	1.00410
age	1	1.09953	0.14756	7.45	<.0001	0.17824	1.02319
BMI	1	1.13253	0.20934	5.41	<.0001	0.13736	1.15268
SBP	1	0.01277	0.07391	0.17	0.8628	0.00437	1.14419
D1_al	1	0.15574	1.82429	0.09	0.9320	0.00206	1.04154
D2_al	1	0.16615	2.20469	0.08	0.9399	0.00181	1.03131
D1_sm	1	-3.79468	3.08043	-1.23	0.2182	-0.02933	1.01379
D2_sm	1	-1.63018	3.00704	-0.54	0.5878	-0.01302	1.03122

Linear Regression Results

The REG Procedure
Model: Linear_Regression_Model
Dependent Variable: HDL

Number of Observations Read	1701
Number of Observations Used	1701

Analysis of Variance					
Source	DF	Sum of Squares	Mean Square	F Value	Pr > F
Model	8	26697	3337.07209	27.87	<.0001
Error	1692	202576	119.72577		
Corrected Total	1700	229273			

Root MSE	10.94193	R-Square	0.1164
Dependent Mean	50.72310	Adj R-Sq	0.1123
Coeff Var	21.57188		

Parameter Estimates							
Variable	DF	Parameter Estimate	Standard Error	t Value	Pr > |t|	Standardized Estimate	Variance Inflation
Intercept	1	76.86276	3.52460	21.81	<.0001	0	0
O2	1	0.07722	0.10356	0.75	0.4560	0.01707	1.00410
age	1	-0.11955	0.05906	-2.02	0.0431	-0.04679	1.02319
BMI	1	-1.14187	0.08378	-13.63	<.0001	-0.33437	1.15268
SBP	1	0.00956	0.02958	0.32	0.7465	0.00790	1.14419
D1_al	1	-1.65003	0.73014	-2.26	0.0240	-0.05270	1.04154
D2_al	1	-0.79895	0.88239	-0.91	0.3654	-0.02101	1.03131
D1_sm	1	0.08494	1.23289	0.07	0.9451	0.00159	1.01379
D2_sm	1	0.36979	1.20352	0.31	0.7587	0.00713	1.03122

Linear Regression Results

The REG Procedure
Model: Linear_Regression_Model
Dependent Variable: LDL

Number of Observations Read	1701
Number of Observations Used	1701

Analysis of Variance					
Source	DF	Sum of Squares	Mean Square	F Value	Pr > F
Model	8	85237	10655	19.42	<.0001
Error	1692	928304	548.64279		
Corrected Total	1700	1013541			

Root MSE	23.42313	R-Square	0.0841
Dependent Mean	110.19089	Adj R-Sq	0.0798
Coeff Var	21.25686		

Parameter Estimates							
Variable	DF	Parameter Estimate	Standard Error	t Value	Pr > |t|	Standardized Estimate	Variance Inflation
Intercept	1	58.74773	7.54504	7.79	<.0001	0	0
O2	1	-0.25094	0.22169	-1.13	0.2578	-0.02639	1.00410
age	1	1.12101	0.12642	8.87	<.0001	0.20868	1.02319
BMI	1	1.44241	0.17935	8.04	<.0001	0.20089	1.15268
SBP	1	-0.04337	0.06332	-0.68	0.4935	-0.01705	1.14419
D1_al	1	0.55161	1.56300	0.35	0.7242	0.00838	1.04154
D2_al	1	-0.06871	1.88892	-0.04	0.9710	-0.00085951	1.03131
D1_sm	1	-3.45372	2.63922	-1.31	0.1908	-0.03066	1.01379
D2_sm	1	-3.20606	2.57634	-1.24	0.2135	-0.02940	1.03122

Linear Regression Results

The REG Procedure
Model: Linear_Regression_Model
Dependent Variable: TG

Number of Observations Read	1701
Number of Observations Used	1701

Analysis of Variance					
Source	DF	Sum of Squares	Mean Square	F Value	Pr > F
Model	8	434454	54307	40.96	<.0001
Error	1692	2243563	1325.98296		
Corrected Total	1700	2678017			

Root MSE	36.41405	R-Square	0.1622
Dependent Mean	78.31805	Adj R-Sq	0.1583
Coeff Var	46.49509		

Parameter Estimates							
Variable	DF	Parameter Estimate	Standard Error	t Value	Pr > |t|	Standardized Estimate	Variance Inflation
Intercept	1	-41.76002	11.72966	-3.56	0.0004	0	0
O2	1	-0.77675	0.34464	-2.25	0.0243	-0.05025	1.00410
age	1	0.46791	0.19654	2.38	0.0174	0.05358	1.02319
BMI	1	4.14416	0.27883	14.86	<.0001	0.35507	1.15268
SBP	1	0.23815	0.09844	2.42	0.0157	0.05758	1.14419
D1_al	1	6.88758	2.42987	2.83	0.0046	0.06437	1.04154
D2_al	1	5.28630	2.93655	1.80	0.0720	0.04068	1.03131
D1_sm	1	-2.05460	4.10299	-0.50	0.6166	-0.01122	1.01379
D2_sm	1	5.79556	4.00523	1.45	0.1481	0.03270	1.03122

Linear Regression Results

The REG Procedure
Model: Linear_Regression_Model
Dependent Variable: TC

Number of Observations Read	1701
Number of Observations Used	1701

Analysis of Variance					
Source	DF	Sum of Squares	Mean Square	F Value	Pr > F
Model	8	70858	8857.29304	11.84	<.0001
Error	1692	1265616	748.00005		
Corrected Total	1700	1336474			

Root MSE	27.34959	R-Square	0.0530
Dependent Mean	176.57202	Adj R-Sq	0.0485
Coeff Var	15.48920		

Parameter Estimates							
Variable	DF	Parameter Estimate	Standard Error	t Value	Pr > |t|	Standardized Estimate	Variance Inflation
Intercept	1	120.56274	9.54787	12.63	<.0001	0	0
O3	1	0.23104	0.36947	0.63	0.5319	0.01490	1.01504
age	1	1.10026	0.14829	7.42	<.0001	0.17836	1.03254
BMI	1	1.13924	0.20934	5.44	<.0001	0.13817	1.15180
SBP	1	0.01372	0.07394	0.19	0.8528	0.00470	1.14443
D1_al	1	0.15708	1.82547	0.09	0.9314	0.00208	1.04207
D2_al	1	0.23610	2.20552	0.11	0.9148	0.00257	1.03127
D1_sm	1	-3.77981	3.08269	-1.23	0.2203	-0.02922	1.01448
D2_sm	1	-1.72746	3.01095	-0.57	0.5662	-0.01380	1.03309

Linear Regression Results

The REG Procedure
Model: Linear_Regression_Model
Dependent Variable: HDL

Number of Observations Read	1701
Number of Observations Used	1701

Analysis of Variance					
Source	DF	Sum of Squares	Mean Square	F Value	Pr > F
Model	8	26803	3350.35039	28.00	<.0001
Error	1692	202470	119.66299		
Corrected Total	1700	229273			

Root MSE	10.93906	R-Square	0.1169
Dependent Mean	50.72310	Adj R-Sq	0.1127
Coeff Var	21.56622		

Parameter Estimates							
Variable	DF	Parameter Estimate	Standard Error	t Value	Pr > |t|	Standardized Estimate	Variance Inflation
Intercept	1	75.47564	3.81887	19.76	<.0001	0	0
O3	1	0.17758	0.14778	1.20	0.2297	0.02766	1.01504
age	1	-0.10992	0.05931	-1.85	0.0640	-0.04302	1.03254
BMI	1	-1.14588	0.08373	-13.69	<.0001	-0.33555	1.15180
SBP	1	0.01003	0.02958	0.34	0.7346	0.00829	1.14443
D1_al	1	-1.62169	0.73014	-2.22	0.0265	-0.05180	1.04207
D2_al	1	-0.79343	0.88214	-0.90	0.3686	-0.02087	1.03127
D1_sm	1	0.01952	1.23299	0.02	0.9874	0.00036423	1.01448
D2_sm	1	0.31163	1.20430	0.26	0.7958	0.00601	1.03309

Linear Regression Results

The REG Procedure
Model: Linear_Regression_Model
Dependent Variable: LDL

Number of Observations Read	1701
Number of Observations Used	1701

Analysis of Variance					
Source	DF	Sum of Squares	Mean Square	F Value	Pr > F
Model	8	84644	10581	19.27	<.0001
Error	1692	928896	548.99303		
Corrected Total	1700	1013541			

Root MSE	23.43060	R-Square	0.0835
Dependent Mean	110.19089	Adj R-Sq	0.0792
Coeff Var	21.26365		

Parameter Estimates							
Variable	DF	Parameter Estimate	Standard Error	t Value	Pr > |t|	Standardized Estimate	Variance Inflation
Intercept	1	54.11680	8.17973	6.62	<.0001	0	0
O3	1	0.14192	0.31653	0.45	0.6539	0.01051	1.01504
age	1	1.12033	0.12704	8.82	<.0001	0.20855	1.03254
BMI	1	1.44769	0.17934	8.07	<.0001	0.20162	1.15180
SBP	1	-0.04275	0.06335	-0.67	0.4998	-0.01680	1.14443
D1_al	1	0.54900	1.56389	0.35	0.7256	0.00834	1.04207
D2_al	1	-0.01967	1.88948	-0.01	0.9917	-0.00024609	1.03127
D1_sm	1	-3.43490	2.64096	-1.30	0.1936	-0.03049	1.01448
D2_sm	1	-3.26791	2.57951	-1.27	0.2054	-0.02997	1.03309

Linear Regression Results

The REG Procedure
Model: Linear_Regression_Model
Dependent Variable: TG

Number of Observations Read	1701
Number of Observations Used	1701

Analysis of Variance					
Source	DF	Sum of Squares	Mean Square	F Value	Pr > F
Model	8	428535	53567	40.29	<.0001
Error	1692	2249482	1329.48101		
Corrected Total	1700	2678017			

Root MSE	36.46205	R-Square	0.1600
Dependent Mean	78.31805	Adj R-Sq	0.1560
Coeff Var	46.55638		

Parameter Estimates							
Variable	DF	Parameter Estimate	Standard Error	t Value	Pr > |t|	Standardized Estimate	Variance Inflation
Intercept	1	-45.60373	12.72907	-3.58	0.0003	0	0
O3	1	-0.38608	0.49258	-0.78	0.4333	-0.01759	1.01504
age	1	0.43064	0.19770	2.18	0.0295	0.04932	1.03254
BMI	1	4.16942	0.27909	14.94	<.0001	0.35724	1.15180
SBP	1	0.23761	0.09858	2.41	0.0160	0.05745	1.14443
D1_al	1	6.77677	2.43369	2.78	0.0054	0.06333	1.04207
D2_al	1	5.36121	2.94036	1.82	0.0684	0.04126	1.03127
D1_sm	1	-1.77392	4.10979	-0.43	0.6661	-0.00969	1.01448
D2_sm	1	5.89207	4.01416	1.47	0.1423	0.03324	1.03309

Linear Regression Results

The REG Procedure
Model: Linear_Regression_Model
Dependent Variable: TC

Number of Observations Read	1701
Number of Observations Used	1701

Analysis of Variance					
Source	DF	Sum of Squares	Mean Square	F Value	Pr > F
Model	8	71556	8944.48494	11.96	<.0001
Error	1692	1264919	747.58779		
Corrected Total	1700	1336474			

Root MSE	27.34205	R-Square	0.0535
Dependent Mean	176.57202	Adj R-Sq	0.0491
Coeff Var	15.48493		

Parameter Estimates							
Variable	DF	Parameter Estimate	Standard Error	t Value	Pr > |t|	Standardized Estimate	Variance Inflation
Intercept	1	127.47365	8.99993	14.16	<.0001	0	0
O4	1	-0.35455	0.30810	-1.15	0.2500	-0.02735	1.01012
age	1	1.08944	0.14742	7.39	<.0001	0.17661	1.02096
BMI	1	1.13379	0.20936	5.42	<.0001	0.13751	1.15264
SBP	1	0.00955	0.07398	0.13	0.8973	0.00327	1.14610
D1_al	1	-0.00292	1.82795	-0.00	0.9987	-0.00003868	1.04547
D2_al	1	0.12250	2.20609	0.06	0.9557	0.00133	1.03238
D1_sm	1	-3.84882	3.08234	-1.25	0.2120	-0.02975	1.01481
D2_sm	1	-1.63262	3.00739	-0.54	0.5873	-0.01304	1.03122

Linear Regression Results

The REG Procedure
Model: Linear_Regression_Model
Dependent Variable: HDL

Number of Observations Read	1701
Number of Observations Used	1701

Analysis of Variance					
Source	DF	Sum of Squares	Mean Square	F Value	Pr > F
Model	8	26996	3374.55951	28.23	<.0001
Error	1692	202276	119.54853		
Corrected Total	1700	229273			

Root MSE	10.93382	R-Square	0.1177
Dependent Mean	50.72310	Adj R-Sq	0.1136
Coeff Var	21.55591		

Parameter Estimates							
Variable	DF	Parameter Estimate	Standard Error	t Value	Pr > |t|	Standardized Estimate	Variance Inflation
Intercept	1	75.31464	3.59898	20.93	<.0001	0	0
O4	1	0.21571	0.12321	1.75	0.0802	0.04018	1.01012
age	1	-0.11687	0.05895	-1.98	0.0476	-0.04574	1.02096
BMI	1	-1.13913	0.08372	-13.61	<.0001	-0.33357	1.15264
SBP	1	0.01162	0.02958	0.39	0.6944	0.00961	1.14610
D1_al	1	-1.56393	0.73098	-2.14	0.0325	-0.04995	1.04547
D2_al	1	-0.75395	0.88220	-0.85	0.3929	-0.01983	1.03238
D1_sm	1	0.14725	1.23260	0.12	0.9049	0.00275	1.01481
D2_sm	1	0.36493	1.20263	0.30	0.7616	0.00704	1.03122

Linear Regression Results

The REG Procedure
Model: Linear_Regression_Model
Dependent Variable: LDL

Number of Observations Read	1701
Number of Observations Used	1701

Analysis of Variance					
Source	DF	Sum of Squares	Mean Square	F Value	Pr > F
Model	8	85149	10644	19.40	<.0001
Error	1692	928391	548.69451		
Corrected Total	1700	1013541			

Root MSE	23.42423	R-Square	0.0840
Dependent Mean	110.19089	Adj R-Sq	0.0797
Coeff Var	21.25786		

Parameter Estimates							
Variable	DF	Parameter Estimate	Standard Error	t Value	Pr > |t|	Standardized Estimate	Variance Inflation
Intercept	1	59.05433	7.71033	7.66	<.0001	0	0
O4	1	-0.27955	0.26395	-1.06	0.2897	-0.02477	1.01012
age	1	1.11350	0.12629	8.82	<.0001	0.20728	1.02096
BMI	1	1.44296	0.17936	8.05	<.0001	0.20096	1.15264
SBP	1	-0.04592	0.06338	-0.72	0.4688	-0.01805	1.14610
D1_al	1	0.42785	1.56602	0.27	0.7847	0.00650	1.04547
D2_al	1	-0.10549	1.88998	-0.06	0.9555	-0.00132	1.03238
D1_sm	1	-3.50016	2.64067	-1.33	0.1852	-0.03107	1.01481
D2_sm	1	-3.20717	2.57646	-1.24	0.2134	-0.02941	1.03122

Linear Regression Results

The REG Procedure
Model: Linear_Regression_Model
Dependent Variable: TG

Number of Observations Read	1701
Number of Observations Used	1701

Analysis of Variance					
Source	DF	Sum of Squares	Mean Square	F Value	Pr > F
Model	8	446549	55819	42.32	<.0001
Error	1692	2231468	1318.83461		
Corrected Total	1700	2678017			

Root MSE	36.31576	R-Square	0.1667
Dependent Mean	78.31805	Adj R-Sq	0.1628
Coeff Var	46.36960		

Parameter Estimates							
Variable	DF	Parameter Estimate	Standard Error	t Value	Pr > |t|	Standardized Estimate	Variance Inflation
Intercept	1	-33.17751	11.95372	-2.78	0.0056	0	0
O4	1	-1.54629	0.40922	-3.78	0.0002	-0.08428	1.01012
age	1	0.44274	0.19580	2.26	0.0239	0.05070	1.02096
BMI	1	4.13060	0.27807	14.85	<.0001	0.35391	1.15264
SBP	1	0.22354	0.09826	2.28	0.0230	0.05405	1.14610
D1_al	1	6.25241	2.42788	2.58	0.0101	0.05843	1.04547
D2_al	1	4.99563	2.93014	1.70	0.0884	0.03844	1.03238
D1_sm	1	-2.45050	4.09397	-0.60	0.5495	-0.01338	1.01481
D2_sm	1	5.81945	3.99442	1.46	0.1453	0.03283	1.03122

Linear Regression Results

The REG Procedure
Model: Linear_Regression_Model
Dependent Variable: TC

Number of Observations Read	1701
Number of Observations Used	1701

Analysis of Variance					
Source	DF	Sum of Squares	Mean Square	F Value	Pr > F
Model	8	71640	8955.04917	11.98	<.0001
Error	1692	1264834	747.53785		
Corrected Total	1700	1336474			

Root MSE	27.34114	R-Square	0.0536
Dependent Mean	176.57202	Adj R-Sq	0.0491
Coeff Var	15.48441		

Parameter Estimates							
Variable	DF	Parameter Estimate	Standard Error	t Value	Pr > |t|	Standardized Estimate	Variance Inflation
Intercept	1	127.36488	8.91414	14.29	<.0001	0	0
O5	1	-0.34336	0.28639	-1.20	0.2307	-0.02846	1.00728
age	1	1.07881	0.14773	7.30	<.0001	0.17489	1.02534
BMI	1	1.13744	0.20927	5.44	<.0001	0.13795	1.15172
SBP	1	0.01113	0.07393	0.15	0.8803	0.00381	1.14471
D1_al	1	0.08622	1.82466	0.05	0.9623	0.00114	1.04179
D2_al	1	0.18470	2.20471	0.08	0.9332	0.00201	1.03115
D1_sm	1	-3.72505	3.08013	-1.21	0.2267	-0.02879	1.01342
D2_sm	1	-1.50978	3.00944	-0.50	0.6160	-0.01206	1.03269

Linear Regression Results

The REG Procedure
Model: Linear_Regression_Model
Dependent Variable: HDL

Number of Observations Read	1701
Number of Observations Used	1701

Analysis of Variance					
Source	DF	Sum of Squares	Mean Square	F Value	Pr > F
Model	8	26634	3329.22641	27.80	<.0001
Error	1692	202639	119.76287		
Corrected Total	1700	229273			

Root MSE	10.94362	R-Square	0.1162
Dependent Mean	50.72310	Adj R-Sq	0.1120
Coeff Var	21.57522		

Parameter Estimates							
Variable	DF	Parameter Estimate	Standard Error	t Value	Pr > |t|	Standardized Estimate	Variance Inflation
Intercept	1	77.96261	3.56799	21.85	<.0001	0	0
O5	1	-0.02042	0.11463	-0.18	0.8586	-0.00409	1.00728
age	1	-0.11817	0.05913	-2.00	0.0458	-0.04625	1.02534
BMI	1	-1.14422	0.08376	-13.66	<.0001	-0.33506	1.15172
SBP	1	0.00939	0.02959	0.32	0.7511	0.00776	1.14471
D1_al	1	-1.64629	0.73034	-2.25	0.0243	-0.05258	1.04179
D2_al	1	-0.81174	0.88246	-0.92	0.3578	-0.02135	1.03115
D1_sm	1	0.06693	1.23286	0.05	0.9567	0.00125	1.01342
D2_sm	1	0.38174	1.20456	0.32	0.7514	0.00736	1.03269

Linear Regression Results

The REG Procedure
Model: Linear_Regression_Model
Dependent Variable: LDL

Number of Observations Read	1701
Number of Observations Used	1701

Analysis of Variance					
Source	DF	Sum of Squares	Mean Square	F Value	Pr > F
Model	8	85212	10651	19.41	<.0001
Error	1692	928329	548.65774		
Corrected Total	1700	1013541			

Root MSE	23.42344	R-Square	0.0841
Dependent Mean	110.19089	Adj R-Sq	0.0797
Coeff Var	21.25715		

Parameter Estimates							
Variable	DF	Parameter Estimate	Standard Error	t Value	Pr > |t|	Standardized Estimate	Variance Inflation
Intercept	1	58.99058	7.63684	7.72	<.0001	0	0
O5	1	-0.27268	0.24535	-1.11	0.2666	-0.02595	1.00728
age	1	1.10505	0.12656	8.73	<.0001	0.20571	1.02534
BMI	1	1.44581	0.17928	8.06	<.0001	0.20136	1.15172
SBP	1	-0.04468	0.06334	-0.71	0.4806	-0.01756	1.14471
D1_al	1	0.49790	1.56321	0.32	0.7501	0.00756	1.04179
D2_al	1	-0.05662	1.88880	-0.03	0.9761	-0.00070821	1.03115
D1_sm	1	-3.40261	2.63878	-1.29	0.1974	-0.03020	1.01342
D2_sm	1	-3.10953	2.57822	-1.21	0.2280	-0.02852	1.03269

Linear Regression Results

The REG Procedure
Model: Linear_Regression_Model
Dependent Variable: TG

Number of Observations Read	1701
Number of Observations Used	1701

Analysis of Variance					
Source	DF	Sum of Squares	Mean Square	F Value	Pr > F
Model	8	428036	53504	40.24	<.0001
Error	1692	2249981	1329.77597		
Corrected Total	1700	2678017			

Root MSE	36.46609	R-Square	0.1598
Dependent Mean	78.31805	Adj R-Sq	0.1559
Coeff Var	46.56155		

Parameter Estimates							
Variable	DF	Parameter Estimate	Standard Error	t Value	Pr > |t|	Standardized Estimate	Variance Inflation
Intercept	1	-48.40898	11.88919	-4.07	<.0001	0	0
O5	1	-0.18670	0.38197	-0.49	0.6251	-0.01093	1.00728
age	1	0.44076	0.19703	2.24	0.0254	0.05048	1.02534
BMI	1	4.16290	0.27911	14.91	<.0001	0.35668	1.15172
SBP	1	0.23772	0.09860	2.41	0.0160	0.05748	1.14471
D1_al	1	6.80193	2.43364	2.79	0.0052	0.06357	1.04179
D2_al	1	5.38093	2.94052	1.83	0.0674	0.04141	1.03115
D1_sm	1	-1.88205	4.10811	-0.46	0.6469	-0.01028	1.01342
D2_sm	1	5.83191	4.01382	1.45	0.1464	0.03290	1.03269

Linear Regression Results

The REG Procedure
Model: Linear_Regression_Model
Dependent Variable: TC

Number of Observations Read	1701
Number of Observations Used	1701

Analysis of Variance					
Source	DF	Sum of Squares	Mean Square	F Value	Pr > F
Model	8	70611	8826.39637	11.80	<.0001
Error	1692	1265863	748.14613		
Corrected Total	1700	1336474			

Root MSE	27.35226	R-Square	0.0528
Dependent Mean	176.57202	Adj R-Sq	0.0484
Coeff Var	15.49071		

Parameter Estimates							
Variable	DF	Parameter Estimate	Standard Error	t Value	Pr > |t|	Standardized Estimate	Variance Inflation
Intercept	1	122.34344	9.54890	12.81	<.0001	0	0
O6	1	0.09685	0.39355	0.25	0.8056	0.00586	1.01258
age	1	1.09307	0.14786	7.39	<.0001	0.17720	1.02634
BMI	1	1.14132	0.20933	5.45	<.0001	0.13842	1.15146
SBP	1	0.01314	0.07394	0.18	0.8590	0.00450	1.14422
D1_al	1	0.15881	1.82927	0.09	0.9308	0.00210	1.04620
D2_al	1	0.24710	2.20942	0.11	0.9110	0.00269	1.03472
D1_sm	1	-3.72349	3.08147	-1.21	0.2271	-0.02878	1.01348
D2_sm	1	-1.65730	3.00879	-0.55	0.5818	-0.01324	1.03141

Linear Regression Results

The REG Procedure
Model: Linear_Regression_Model
Dependent Variable: HDL

Number of Observations Read	1701
Number of Observations Used	1701

Analysis of Variance					
Source	DF	Sum of Squares	Mean Square	F Value	Pr > F
Model	8	26677	3334.56628	27.85	<.0001
Error	1692	202596	119.73762		
Corrected Total	1700	229273			

Root MSE	10.94247	R-Square	0.1164
Dependent Mean	50.72310	Adj R-Sq	0.1122
Coeff Var	21.57295		

Parameter Estimates							
Variable	DF	Parameter Estimate	Standard Error	t Value	Pr > |t|	Standardized Estimate	Variance Inflation
Intercept	1	76.56153	3.82011	20.04	<.0001	0	0
O6	1	0.09814	0.15744	0.62	0.5332	0.01433	1.01258
age	1	-0.11480	0.05915	-1.94	0.0525	-0.04493	1.02634
BMI	1	-1.14439	0.08374	-13.67	<.0001	-0.33511	1.15146
SBP	1	0.00960	0.02958	0.32	0.7455	0.00794	1.14422
D1_al	1	-1.61290	0.73181	-2.20	0.0277	-0.05152	1.04620
D2_al	1	-0.77701	0.88389	-0.88	0.3795	-0.02044	1.03472
D1_sm	1	0.06135	1.23277	0.05	0.9603	0.00115	1.01348
D2_sm	1	0.36300	1.20369	0.30	0.7630	0.00700	1.03141

Linear Regression Results

The REG Procedure
Model: Linear_Regression_Model
Dependent Variable: LDL

Number of Observations Read	1701
Number of Observations Used	1701

Analysis of Variance					
Source	DF	Sum of Squares	Mean Square	F Value	Pr > F
Model	8	84576	10572	19.26	<.0001
Error	1692	928964	549.03322		
Corrected Total	1700	1013541			

Root MSE	23.43146	R-Square	0.0834
Dependent Mean	110.19089	Adj R-Sq	0.0791
Coeff Var	21.26442		

Parameter Estimates							
Variable	DF	Parameter Estimate	Standard Error	t Value	Pr > |t|	Standardized Estimate	Variance Inflation
Intercept	1	54.80296	8.18012	6.70	<.0001	0	0
O6	1	0.09366	0.33714	0.28	0.7812	0.00651	1.01258
age	1	1.11684	0.12666	8.82	<.0001	0.20790	1.02634
BMI	1	1.44882	0.17932	8.08	<.0001	0.20178	1.15146
SBP	1	-0.04308	0.06335	-0.68	0.4966	-0.01693	1.14422
D1_al	1	0.56082	1.56705	0.36	0.7205	0.00852	1.04620
D2_al	1	-0.00144	1.89271	-0.00	0.9994	-0.00001806	1.03472
D1_sm	1	-3.40240	2.63976	-1.29	0.1976	-0.03020	1.01348
D2_sm	1	-3.22849	2.57749	-1.25	0.2105	-0.02961	1.03141

Linear Regression Results

The REG Procedure
Model: Linear_Regression_Model
Dependent Variable: TG

Number of Observations Read	1701
Number of Observations Used	1701

Analysis of Variance					
Source	DF	Sum of Squares	Mean Square	F Value	Pr > F
Model	8	428478	53560	40.29	<.0001
Error	1692	2249539	1329.51448		
Corrected Total	1700	2678017			

Root MSE	36.46251	R-Square	0.1600
Dependent Mean	78.31805	Adj R-Sq	0.1560
Coeff Var	46.55697		

Parameter Estimates							
Variable	DF	Parameter Estimate	Standard Error	t Value	Pr > |t|	Standardized Estimate	Variance Inflation
Intercept	1	-45.77672	12.72937	-3.60	0.0003	0	0
O6	1	-0.39669	0.52463	-0.76	0.4497	-0.01695	1.01258
age	1	0.43625	0.19711	2.21	0.0270	0.04996	1.02634
BMI	1	4.16696	0.27905	14.93	<.0001	0.35703	1.15146
SBP	1	0.23835	0.09857	2.42	0.0157	0.05763	1.14422
D1_al	1	6.69996	2.43854	2.75	0.0061	0.06262	1.04620
D2_al	1	5.26396	2.94532	1.79	0.0741	0.04051	1.03472
D1_sm	1	-1.85363	4.10782	-0.45	0.6519	-0.01012	1.01348
D2_sm	1	5.80015	4.01092	1.45	0.1483	0.03272	1.03141

Linear Regression Results

The REG Procedure
Model: Linear_Regression_Model
Dependent Variable: TC

Number of Observations Read	1701
Number of Observations Used	1701

Analysis of Variance					
Source	DF	Sum of Squares	Mean Square	F Value	Pr > F
Model	8	70758	8844.70937	11.82	<.0001
Error	1692	1265717	748.05955		
Corrected Total	1700	1336474			

Root MSE	27.35068	R-Square	0.0529
Dependent Mean	176.57202	Adj R-Sq	0.0485
Coeff Var	15.48981		

Parameter Estimates							
Variable	DF	Parameter Estimate	Standard Error	t Value	Pr > |t|	Standardized Estimate	Variance Inflation
Intercept	1	122.00302	8.82299	13.83	<.0001	0	0
A1	1	0.17608	0.34774	0.51	0.6127	0.01202	1.00671
age	1	1.08611	0.14771	7.35	<.0001	0.17607	1.02434
BMI	1	1.14518	0.20942	5.47	<.0001	0.13889	1.15260
SBP	1	0.01205	0.07397	0.16	0.8707	0.00412	1.14498
D1_al	1	0.13101	1.82497	0.07	0.9428	0.00173	1.04141
D2_al	1	0.20335	2.20545	0.09	0.9265	0.00222	1.03113
D1_sm	1	-3.71220	3.08122	-1.20	0.2285	-0.02869	1.01343
D2_sm	1	-1.58603	3.01071	-0.53	0.5984	-0.01267	1.03285

Linear Regression Results

The REG Procedure
Model: Linear_Regression_Model
Dependent Variable: HDL

Number of Observations Read	1701
Number of Observations Used	1701

Analysis of Variance					
Source	DF	Sum of Squares	Mean Square	F Value	Pr > F
Model	8	26797	3349.59634	27.99	<.0001
Error	1692	202476	119.66656		
Corrected Total	1700	229273			

Root MSE	10.93922	R-Square	0.1169
Dependent Mean	50.72310	Adj R-Sq	0.1127
Coeff Var	21.56654		

Parameter Estimates							
Variable	DF	Parameter Estimate	Standard Error	t Value	Pr > |t|	Standardized Estimate	Variance Inflation
Intercept	1	76.33761	3.52886	21.63	<.0001	0	0
A1	1	0.16418	0.13908	1.18	0.2380	0.02706	1.00671
age	1	-0.12150	0.05908	-2.06	0.0399	-0.04756	1.02434
BMI	1	-1.14076	0.08376	-13.62	<.0001	-0.33404	1.15260
SBP	1	0.00858	0.02958	0.29	0.7719	0.00709	1.14498
D1_al	1	-1.64129	0.72992	-2.25	0.0247	-0.05242	1.04141
D2_al	1	-0.82044	0.88210	-0.93	0.3525	-0.02158	1.03113
D1_sm	1	0.07236	1.23237	0.06	0.9532	0.00135	1.01343
D2_sm	1	0.43030	1.20417	0.36	0.7209	0.00830	1.03285

Linear Regression Results

The REG Procedure
Model: Linear_Regression_Model
Dependent Variable: LDL

Number of Observations Read	1701
Number of Observations Used	1701

Analysis of Variance					
Source	DF	Sum of Squares	Mean Square	F Value	Pr > F
Model	8	84535	10567	19.25	<.0001
Error	1692	929005	549.05759		
Corrected Total	1700	1013541			

Root MSE	23.43198	R-Square	0.0834
Dependent Mean	110.19089	Adj R-Sq	0.0791
Coeff Var	21.26490		

Parameter Estimates							
Variable	DF	Parameter Estimate	Standard Error	t Value	Pr > |t|	Standardized Estimate	Variance Inflation
Intercept	1	56.03513	7.55886	7.41	<.0001	0	0
A1	1	-0.01347	0.29791	-0.05	0.9639	-0.00106	1.00671
age	1	1.11462	0.12654	8.81	<.0001	0.20749	1.02434
BMI	1	1.44896	0.17942	8.08	<.0001	0.20180	1.15260
SBP	1	-0.04310	0.06337	-0.68	0.4965	-0.01694	1.14498
D1_al	1	0.53113	1.56350	0.34	0.7341	0.00807	1.04141
D2_al	1	-0.03204	1.88946	-0.02	0.9865	-0.00040072	1.03113
D1_sm	1	-3.39706	2.63975	-1.29	0.1983	-0.03015	1.01343
D2_sm	1	-3.22305	2.57935	-1.25	0.2116	-0.02956	1.03285

Linear Regression Results

The REG Procedure
Model: Linear_Regression_Model
Dependent Variable: TG

Number of Observations Read	1701
Number of Observations Used	1701

Analysis of Variance					
Source	DF	Sum of Squares	Mean Square	F Value	Pr > F
Model	8	427719	53465	40.20	<.0001
Error	1692	2250298	1329.96336		
Corrected Total	1700	2678017			

Root MSE	36.46866	R-Square	0.1597
Dependent Mean	78.31805	Adj R-Sq	0.1557
Coeff Var	46.56483		

Parameter Estimates							
Variable	DF	Parameter Estimate	Standard Error	t Value	Pr > |t|	Standardized Estimate	Variance Inflation
Intercept	1	-50.42557	11.76433	-4.29	<.0001	0	0
A1	1	-0.01004	0.46366	-0.02	0.9827	-0.00048422	1.00671
age	1	0.44732	0.19695	2.27	0.0233	0.05123	1.02434
BMI	1	4.16505	0.27924	14.92	<.0001	0.35686	1.15260
SBP	1	0.23881	0.09862	2.42	0.0156	0.05774	1.14498
D1_al	1	6.82467	2.43337	2.80	0.0051	0.06378	1.04141
D2_al	1	5.39782	2.94069	1.84	0.0666	0.04154	1.03113
D1_sm	1	-1.87827	4.10842	-0.46	0.6476	-0.01026	1.01343
D2_sm	1	5.75391	4.01440	1.43	0.1520	0.03246	1.03285

Linear Regression Results

The REG Procedure
Model: Linear_Regression_Model
Dependent Variable: TC

Number of Observations Read	1701
Number of Observations Used	1701

Analysis of Variance					
Source	DF	Sum of Squares	Mean Square	F Value	Pr > F
Model	8	70578	8822.29190	11.79	<.0001
Error	1692	1265896	748.16554		
Corrected Total	1700	1336474			

Root MSE	27.35261	R-Square	0.0528
Dependent Mean	176.57202	Adj R-Sq	0.0483
Coeff Var	15.49091		

Parameter Estimates							
Variable	DF	Parameter Estimate	Standard Error	t Value	Pr > |t|	Standardized Estimate	Variance Inflation
Intercept	1	123.28775	8.47392	14.55	<.0001	0	0
A2	1	0.03674	0.28455	0.13	0.8973	0.00315	1.06578
age	1	1.08616	0.15113	7.19	<.0001	0.17608	1.07222
BMI	1	1.14373	0.20990	5.45	<.0001	0.13872	1.15766
SBP	1	0.01234	0.07414	0.17	0.8679	0.00422	1.15034
D1_al	1	0.11268	1.82911	0.06	0.9509	0.00149	1.04600
D2_al	1	0.20289	2.20735	0.09	0.9268	0.00221	1.03276
D1_sm	1	-3.70128	3.08399	-1.20	0.2302	-0.02861	1.01511
D2_sm	1	-1.63775	3.00935	-0.54	0.5864	-0.01308	1.03177

Linear Regression Results

The REG Procedure
Model: Linear_Regression_Model
Dependent Variable: HDL

Number of Observations Read	1701
Number of Observations Used	1701

Analysis of Variance					
Source	DF	Sum of Squares	Mean Square	F Value	Pr > F
Model	8	27210	3401.29778	28.48	<.0001
Error	1692	202062	119.42210		
Corrected Total	1700	229273			

Root MSE	10.92804	R-Square	0.1187
Dependent Mean	50.72310	Adj R-Sq	0.1145
Coeff Var	21.54451		

Parameter Estimates							
Variable	DF	Parameter Estimate	Standard Error	t Value	Pr > |t|	Standardized Estimate	Variance Inflation
Intercept	1	76.28996	3.38554	22.53	<.0001	0	0
A2	1	0.25062	0.11369	2.20	0.0276	0.05194	1.06578
age	1	-0.14659	0.06038	-2.43	0.0153	-0.05737	1.07222
BMI	1	-1.13035	0.08386	-13.48	<.0001	-0.33100	1.15766
SBP	1	0.00472	0.02962	0.16	0.8733	0.00390	1.15034
D1_al	1	-1.75055	0.73078	-2.40	0.0167	-0.05591	1.04600
D2_al	1	-0.88967	0.88189	-1.01	0.3132	-0.02340	1.03276
D1_sm	1	0.17835	1.23213	0.14	0.8849	0.00333	1.01511
D2_sm	1	0.43570	1.20231	0.36	0.7171	0.00840	1.03177

Linear Regression Results

The REG Procedure
Model: Linear_Regression_Model
Dependent Variable: LDL

Number of Observations Read	1701
Number of Observations Used	1701

Analysis of Variance					
Source	DF	Sum of Squares	Mean Square	F Value	Pr > F
Model	8	84839	10605	19.32	<.0001
Error	1692	928701	548.87786		
Corrected Total	1700	1013541			

Root MSE	23.42814	R-Square	0.0837
Dependent Mean	110.19089	Adj R-Sq	0.0794
Coeff Var	21.26142		

Parameter Estimates							
Variable	DF	Parameter Estimate	Standard Error	t Value	Pr > |t|	Standardized Estimate	Variance Inflation
Intercept	1	56.96695	7.25811	7.85	<.0001	0	0
A2	1	-0.18175	0.24373	-0.75	0.4559	-0.01792	1.06578
age	1	1.13540	0.12945	8.77	<.0001	0.21136	1.07222
BMI	1	1.43935	0.17978	8.01	<.0001	0.20046	1.15766
SBP	1	-0.03971	0.06351	-0.63	0.5319	-0.01561	1.15034
D1_al	1	0.60877	1.56668	0.39	0.6976	0.00925	1.04600
D2_al	1	0.02490	1.89065	0.01	0.9895	0.00031150	1.03276
D1_sm	1	-3.47713	2.64151	-1.32	0.1882	-0.03086	1.01511
D2_sm	1	-3.26344	2.57758	-1.27	0.2057	-0.02993	1.03177

Linear Regression Results

The REG Procedure
Model: Linear_Regression_Model
Dependent Variable: TG

Number of Observations Read	1701
Number of Observations Used	1701

Analysis of Variance					
Source	DF	Sum of Squares	Mean Square	F Value	Pr > F
Model	8	428055	53507	40.24	<.0001
Error	1692	2249962	1329.76489		
Corrected Total	1700	2678017			

Root MSE	36.46594	R-Square	0.1598
Dependent Mean	78.31805	Adj R-Sq	0.1559
Coeff Var	46.56135		

Parameter Estimates							
Variable	DF	Parameter Estimate	Standard Error	t Value	Pr > |t|	Standardized Estimate	Variance Inflation
Intercept	1	-49.41243	11.29726	-4.37	<.0001	0	0
A2	1	-0.19081	0.37936	-0.50	0.6150	-0.01157	1.06578
age	1	0.46924	0.20148	2.33	0.0200	0.05374	1.07222
BMI	1	4.15488	0.27983	14.85	<.0001	0.35599	1.15766
SBP	1	0.24239	0.09885	2.45	0.0143	0.05861	1.15034
D1_al	1	6.90611	2.43854	2.83	0.0047	0.06454	1.04600
D2_al	1	5.45786	2.94279	1.85	0.0638	0.04200	1.03276
D1_sm	1	-1.96246	4.11152	-0.48	0.6332	-0.01072	1.01511
D2_sm	1	5.71008	4.01201	1.42	0.1548	0.03221	1.03177

Linear Regression Results

The REG Procedure
Model: Linear_Regression_Model
Dependent Variable: TC

Number of Observations Read	1701
Number of Observations Used	1701

Analysis of Variance					
Source	DF	Sum of Squares	Mean Square	F Value	Pr > F
Model	8	70614	8826.76247	11.80	<.0001
Error	1692	1265860	748.14440		
Corrected Total	1700	1336474			

Root MSE	27.35223	R-Square	0.0528
Dependent Mean	176.57202	Adj R-Sq	0.0484
Coeff Var	15.49069		

Parameter Estimates							
Variable	DF	Parameter Estimate	Standard Error	t Value	Pr > |t|	Standardized Estimate	Variance Inflation
Intercept	1	122.42110	9.33547	13.11	<.0001	0	0
A3	1	0.09432	0.37145	0.25	0.7996	0.00602	1.00331
age	1	1.09124	0.14750	7.40	<.0001	0.17690	1.02141
BMI	1	1.14068	0.20936	5.45	<.0001	0.13835	1.15183
SBP	1	0.01322	0.07395	0.18	0.8581	0.00453	1.14429
D1_al	1	0.11214	1.82618	0.06	0.9510	0.00148	1.04268
D2_al	1	0.21196	2.20549	0.10	0.9234	0.00231	1.03104
D1_sm	1	-3.72092	3.08140	-1.21	0.2274	-0.02876	1.01344
D2_sm	1	-1.62351	3.00989	-0.54	0.5897	-0.01297	1.03216

Linear Regression Results

The REG Procedure
Model: Linear_Regression_Model
Dependent Variable: HDL

Number of Observations Read	1701
Number of Observations Used	1701

Analysis of Variance					
Source	DF	Sum of Squares	Mean Square	F Value	Pr > F
Model	8	26884	3360.53536	28.09	<.0001
Error	1692	202388	119.61483		
Corrected Total	1700	229273			

Root MSE	10.93686	R-Square	0.1173
Dependent Mean	50.72310	Adj R-Sq	0.1131
Coeff Var	21.56188		

Parameter Estimates							
Variable	DF	Parameter Estimate	Standard Error	t Value	Pr > |t|	Standardized Estimate	Variance Inflation
Intercept	1	75.25733	3.73281	20.16	<.0001	0	0
A3	1	0.21655	0.14852	1.46	0.1450	0.03336	1.00331
age	1	-0.11561	0.05898	-1.96	0.0501	-0.04525	1.02141
BMI	1	-1.14638	0.08371	-13.69	<.0001	-0.33569	1.15183
SBP	1	0.00993	0.02957	0.34	0.7371	0.00820	1.14429
D1_al	1	-1.68096	0.73020	-2.30	0.0215	-0.05369	1.04268
D2_al	1	-0.81596	0.88187	-0.93	0.3550	-0.02146	1.03104
D1_sm	1	0.05962	1.23211	0.05	0.9614	0.00111	1.01344
D2_sm	1	0.42718	1.20351	0.35	0.7227	0.00824	1.03216

Linear Regression Results

The REG Procedure
Model: Linear_Regression_Model
Dependent Variable: LDL

Number of Observations Read	1701
Number of Observations Used	1701

Analysis of Variance					
Source	DF	Sum of Squares	Mean Square	F Value	Pr > F
Model	8	84538	10567	19.25	<.0001
Error	1692	929002	549.05556		
Corrected Total	1700	1013541			

Root MSE	23.43193	R-Square	0.0834
Dependent Mean	110.19089	Adj R-Sq	0.0791
Coeff Var	21.26486		

Parameter Estimates							
Variable	DF	Parameter Estimate	Standard Error	t Value	Pr > |t|	Standardized Estimate	Variance Inflation
Intercept	1	56.25201	7.99745	7.03	<.0001	0	0
A3	1	-0.02899	0.31821	-0.09	0.9274	-0.00212	1.00331
age	1	1.11404	0.12636	8.82	<.0001	0.20738	1.02141
BMI	1	1.44955	0.17936	8.08	<.0001	0.20188	1.15183
SBP	1	-0.04323	0.06335	-0.68	0.4951	-0.01699	1.14429
D1_al	1	0.53631	1.56444	0.34	0.7318	0.00815	1.04268
D2_al	1	-0.03209	1.88938	-0.02	0.9865	-0.00040143	1.03104
D1_sm	1	-3.39561	2.63976	-1.29	0.1985	-0.03014	1.01344
D2_sm	1	-3.22557	2.57849	-1.25	0.2111	-0.02958	1.03216

Linear Regression Results

The REG Procedure
Model: Linear_Regression_Model
Dependent Variable: TG

Number of Observations Read	1701
Number of Observations Used	1701

Analysis of Variance					
Source	DF	Sum of Squares	Mean Square	F Value	Pr > F
Model	8	428953	53619	40.34	<.0001
Error	1692	2249064	1329.23418		
Corrected Total	1700	2678017			

Root MSE	36.45866	R-Square	0.1602
Dependent Mean	78.31805	Adj R-Sq	0.1562
Coeff Var	46.55206		

Parameter Estimates							
Variable	DF	Parameter Estimate	Standard Error	t Value	Pr > |t|	Standardized Estimate	Variance Inflation
Intercept	1	-45.05685	12.44354	-3.62	0.0003	0	0
A3	1	-0.47712	0.49511	-0.96	0.3354	-0.02150	1.00331
age	1	0.44296	0.19661	2.25	0.0244	0.05073	1.02141
BMI	1	4.17056	0.27907	14.94	<.0001	0.35733	1.15183
SBP	1	0.23782	0.09857	2.41	0.0159	0.05750	1.14429
D1_al	1	6.90672	2.43417	2.84	0.0046	0.06455	1.04268
D2_al	1	5.41038	2.93976	1.84	0.0659	0.04163	1.03104
D1_sm	1	-1.86087	4.10730	-0.45	0.6506	-0.01016	1.01344
D2_sm	1	5.63930	4.01197	1.41	0.1600	0.03182	1.03216

Linear Regression Results

The REG Procedure
Model: Linear_Regression_Model
Dependent Variable: TC

Number of Observations Read	1701
Number of Observations Used	1701

Analysis of Variance					
Source	DF	Sum of Squares	Mean Square	F Value	Pr > F
Model	8	70994	8874.22144	11.87	<.0001
Error	1692	1265481	747.92001		
Corrected Total	1700	1336474			

Root MSE	27.34813	R-Square	0.0531
Dependent Mean	176.57202	Adj R-Sq	0.0486
Coeff Var	15.48837		

Parameter Estimates							
Variable	DF	Parameter Estimate	Standard Error	t Value	Pr > |t|	Standardized Estimate	Variance Inflation
Intercept	1	121.47811	8.73163	13.91	<.0001	0	0
A4	1	0.26490	0.35022	0.76	0.4495	0.01806	1.01819
age	1	1.08140	0.14793	7.31	<.0001	0.17531	1.02762
BMI	1	1.14634	0.20938	5.47	<.0001	0.13903	1.15236
SBP	1	0.01121	0.07397	0.15	0.8796	0.00384	1.14540
D1_al	1	0.03816	1.82868	0.02	0.9834	0.00050484	1.04585
D2_al	1	0.17787	2.20567	0.08	0.9357	0.00194	1.03152
D1_sm	1	-3.63290	3.08295	-1.18	0.2388	-0.02808	1.01476
D2_sm	1	-1.49347	3.01486	-0.50	0.6204	-0.01193	1.03589

Linear Regression Results

The REG Procedure
Model: Linear_Regression_Model
Dependent Variable: HDL

Number of Observations Read	1701
Number of Observations Used	1701

Analysis of Variance					
Source	DF	Sum of Squares	Mean Square	F Value	Pr > F
Model	8	26632	3328.95136	27.80	<.0001
Error	1692	202641	119.76417		
Corrected Total	1700	229273			

Root MSE	10.94368	R-Square	0.1162
Dependent Mean	50.72310	Adj R-Sq	0.1120
Coeff Var	21.57534		

Parameter Estimates							
Variable	DF	Parameter Estimate	Standard Error	t Value	Pr > |t|	Standardized Estimate	Variance Inflation
Intercept	1	77.60910	3.49407	22.21	<.0001	0	0
A4	1	0.01620	0.14014	0.12	0.9080	0.00267	1.01819
age	1	-0.11803	0.05920	-1.99	0.0463	-0.04620	1.02762
BMI	1	-1.14368	0.08379	-13.65	<.0001	-0.33490	1.15236
SBP	1	0.00939	0.02960	0.32	0.7511	0.00776	1.14540
D1_al	1	-1.64930	0.73177	-2.25	0.0243	-0.05268	1.04585
D2_al	1	-0.81221	0.88262	-0.92	0.3576	-0.02136	1.03152
D1_sm	1	0.07255	1.23368	0.06	0.9531	0.00135	1.01476
D2_sm	1	0.38296	1.20643	0.32	0.7510	0.00738	1.03589

Linear Regression Results

The REG Procedure
Model: Linear_Regression_Model
Dependent Variable: LDL

Number of Observations Read	1701
Number of Observations Used	1701

Analysis of Variance					
Source	DF	Sum of Squares	Mean Square	F Value	Pr > F
Model	8	84767	10596	19.30	<.0001
Error	1692	928774	548.92062		
Corrected Total	1700	1013541			

Root MSE	23.42906	R-Square	0.0836
Dependent Mean	110.19089	Adj R-Sq	0.0793
Coeff Var	21.26224		

Parameter Estimates							
Variable	DF	Parameter Estimate	Standard Error	t Value	Pr > |t|	Standardized Estimate	Variance Inflation
Intercept	1	54.42966	7.48036	7.28	<.0001	0	0
A4	1	0.19542	0.30003	0.65	0.5149	0.01530	1.01819
age	1	1.10763	0.12673	8.74	<.0001	0.20619	1.02762
BMI	1	1.45262	0.17938	8.10	<.0001	0.20231	1.15236
SBP	1	-0.04452	0.06337	-0.70	0.4824	-0.01750	1.14540
D1_al	1	0.46482	1.56663	0.30	0.7667	0.00706	1.04585
D2_al	1	-0.05997	1.88959	-0.03	0.9747	-0.00075011	1.03152
D1_sm	1	-3.33421	2.64115	-1.26	0.2070	-0.02959	1.01476
D2_sm	1	-3.10524	2.58282	-1.20	0.2294	-0.02848	1.03589

Linear Regression Results

The REG Procedure
Model: Linear_Regression_Model
Dependent Variable: TG

Number of Observations Read	1701
Number of Observations Used	1701

Analysis of Variance					
Source	DF	Sum of Squares	Mean Square	F Value	Pr > F
Model	8	428136	53517	40.25	<.0001
Error	1692	2249881	1329.71684		
Corrected Total	1700	2678017			

Root MSE	36.46528	R-Square	0.1599
Dependent Mean	78.31805	Adj R-Sq	0.1559
Coeff Var	46.56051		

Parameter Estimates							
Variable	DF	Parameter Estimate	Standard Error	t Value	Pr > |t|	Standardized Estimate	Variance Inflation
Intercept	1	-52.50784	11.64252	-4.51	<.0001	0	0
A4	1	0.26174	0.46697	0.56	0.5752	0.01260	1.01819
age	1	0.43816	0.19724	2.22	0.0265	0.05018	1.02762
BMI	1	4.16980	0.27918	14.94	<.0001	0.35727	1.15236
SBP	1	0.23695	0.09863	2.40	0.0164	0.05729	1.14540
D1_al	1	6.73573	2.43832	2.76	0.0058	0.06295	1.04585
D2_al	1	5.36092	2.94098	1.82	0.0685	0.04125	1.03152
D1_sm	1	-1.79433	4.11072	-0.44	0.6625	-0.00980	1.01476
D2_sm	1	5.90892	4.01993	1.47	0.1418	0.03334	1.03589

Linear Regression Results

The REG Procedure
Model: Linear_Regression_Model
Dependent Variable: TC

Number of Observations Read	1701
Number of Observations Used	1701

Analysis of Variance					
Source	DF	Sum of Squares	Mean Square	F Value	Pr > F
Model	8	70792	8849.01500	11.83	<.0001
Error	1692	1265682	748.03919		
Corrected Total	1700	1336474			

Root MSE	27.35031	R-Square	0.0530
Dependent Mean	176.57202	Adj R-Sq	0.0485
Coeff Var	15.48960		

Parameter Estimates							
Variable	DF	Parameter Estimate	Standard Error	t Value	Pr > |t|	Standardized Estimate	Variance Inflation
Intercept	1	125.33636	8.95939	13.99	<.0001	0	0
A5	1	-0.19502	0.35459	-0.55	0.5824	-0.01315	1.02118
age	1	1.09646	0.14786	7.42	<.0001	0.17775	1.02656
BMI	1	1.13626	0.20954	5.42	<.0001	0.13781	1.15399
SBP	1	0.01451	0.07399	0.20	0.8446	0.00497	1.14568
D1_al	1	0.16520	1.82617	0.09	0.9279	0.00219	1.04281
D2_al	1	0.26051	2.20689	0.12	0.9060	0.00284	1.03250
D1_sm	1	-3.77361	3.08285	-1.22	0.2211	-0.02917	1.01453
D2_sm	1	-1.80063	3.02124	-0.60	0.5513	-0.01438	1.04011

Linear Regression Results

The REG Procedure
Model: Linear_Regression_Model
Dependent Variable: HDL

Number of Observations Read	1701
Number of Observations Used	1701

Analysis of Variance					
Source	DF	Sum of Squares	Mean Square	F Value	Pr > F
Model	8	26689	3336.16756	27.86	<.0001
Error	1692	202583	119.73005		
Corrected Total	1700	229273			

Root MSE	10.94212	R-Square	0.1164
Dependent Mean	50.72310	Adj R-Sq	0.1122
Coeff Var	21.57227		

Parameter Estimates							
Variable	DF	Parameter Estimate	Standard Error	t Value	Pr > |t|	Standardized Estimate	Variance Inflation
Intercept	1	76.79196	3.58441	21.42	<.0001	0	0
A5	1	0.09986	0.14186	0.70	0.4816	0.01626	1.02118
age	1	-0.12056	0.05916	-2.04	0.0417	-0.04719	1.02656
BMI	1	-1.14116	0.08383	-13.61	<.0001	-0.33416	1.15399
SBP	1	0.00875	0.02960	0.30	0.7676	0.00723	1.14568
D1_al	1	-1.66267	0.73060	-2.28	0.0230	-0.05311	1.04281
D2_al	1	-0.83349	0.88292	-0.94	0.3453	-0.02192	1.03250
D1_sm	1	0.09609	1.23336	0.08	0.9379	0.00179	1.01453
D2_sm	1	0.45233	1.20872	0.37	0.7083	0.00872	1.04011

Linear Regression Results

The REG Procedure
Model: Linear_Regression_Model
Dependent Variable: LDL

Number of Observations Read	1701
Number of Observations Used	1701

Analysis of Variance					
Source	DF	Sum of Squares	Mean Square	F Value	Pr > F
Model	8	85317	10665	19.44	<.0001
Error	1692	928223	548.59531		
Corrected Total	1700	1013541			

Root MSE	23.42211	R-Square	0.0842
Dependent Mean	110.19089	Adj R-Sq	0.0798
Coeff Var	21.25594		

Parameter Estimates							
Variable	DF	Parameter Estimate	Standard Error	t Value	Pr > |t|	Standardized Estimate	Variance Inflation
Intercept	1	59.33892	7.67260	7.73	<.0001	0	0
A5	1	-0.36286	0.30367	-1.19	0.2323	-0.02809	1.02118
age	1	1.12550	0.12663	8.89	<.0001	0.20951	1.02656
BMI	1	1.43903	0.17945	8.02	<.0001	0.20042	1.15399
SBP	1	-0.04044	0.06336	-0.64	0.5234	-0.01589	1.14568
D1_al	1	0.59995	1.56388	0.38	0.7013	0.00911	1.04281
D2_al	1	0.05258	1.88992	0.03	0.9778	0.00065768	1.03250
D1_sm	1	-3.50097	2.64007	-1.33	0.1850	-0.03107	1.01453
D2_sm	1	-3.50451	2.58731	-1.35	0.1758	-0.03214	1.04011

Linear Regression Results

The REG Procedure
Model: Linear_Regression_Model
Dependent Variable: TG

Number of Observations Read	1701
Number of Observations Used	1701

Analysis of Variance					
Source	DF	Sum of Squares	Mean Square	F Value	Pr > F
Model	8	428649	53581	40.30	<.0001
Error	1692	2249368	1329.41378		
Corrected Total	1700	2678017			

Root MSE	36.46113	R-Square	0.1601
Dependent Mean	78.31805	Adj R-Sq	0.1561
Coeff Var	46.55520		

Parameter Estimates							
Variable	DF	Parameter Estimate	Standard Error	t Value	Pr > |t|	Standardized Estimate	Variance Inflation
Intercept	1	-54.23650	11.94391	-4.54	<.0001	0	0
A5	1	0.39548	0.47272	0.84	0.4029	0.01884	1.02118
age	1	0.43486	0.19712	2.21	0.0275	0.04980	1.02656
BMI	1	4.17635	0.27935	14.95	<.0001	0.35783	1.15399
SBP	1	0.23577	0.09863	2.39	0.0169	0.05701	1.14568
D1_al	1	6.75005	2.43449	2.77	0.0056	0.06308	1.04281
D2_al	1	5.30402	2.94204	1.80	0.0716	0.04082	1.03250
D1_sm	1	-1.76426	4.10979	-0.43	0.6678	-0.00963	1.01453
D2_sm	1	6.06922	4.02766	1.51	0.1320	0.03424	1.04011

Linear Regression Results

The REG Procedure
Model: Linear_Regression_Model
Dependent Variable: TC

Number of Observations Read	1701
Number of Observations Used	1701

Analysis of Variance					
Source	DF	Sum of Squares	Mean Square	F Value	Pr > F
Model	8	70748	8843.50364	11.82	<.0001
Error	1692	1265726	748.06525		
Corrected Total	1700	1336474			

Root MSE	27.35078	R-Square	0.0529
Dependent Mean	176.57202	Adj R-Sq	0.0485
Coeff Var	15.48987		

Parameter Estimates							
Variable	DF	Parameter Estimate	Standard Error	t Value	Pr > |t|	Standardized Estimate	Variance Inflation
Intercept	1	121.92475	8.90477	13.69	<.0001	0	0
A6	1	0.14613	0.29614	0.49	0.6217	0.01172	1.00768
age	1	1.09255	0.14752	7.41	<.0001	0.17711	1.02179
BMI	1	1.14450	0.20939	5.47	<.0001	0.13881	1.15221
SBP	1	0.01281	0.07394	0.17	0.8624	0.00439	1.14422
D1_al	1	0.06973	1.82883	0.04	0.9696	0.00092247	1.04581
D2_al	1	0.18975	2.20592	0.09	0.9315	0.00207	1.03156
D1_sm	1	-3.68438	3.08195	-1.20	0.2321	-0.02848	1.01390
D2_sm	1	-1.62318	3.00871	-0.54	0.5896	-0.01296	1.03146

Linear Regression Results

The REG Procedure
Model: Linear_Regression_Model
Dependent Variable: HDL

Number of Observations Read	1701
Number of Observations Used	1701

Analysis of Variance					
Source	DF	Sum of Squares	Mean Square	F Value	Pr > F
Model	8	26679	3334.83274	27.85	<.0001
Error	1692	202594	119.73636		
Corrected Total	1700	229273			

Root MSE	10.94241	R-Square	0.1164
Dependent Mean	50.72310	Adj R-Sq	0.1122
Coeff Var	21.57283		

Parameter Estimates							
Variable	DF	Parameter Estimate	Standard Error	t Value	Pr > |t|	Standardized Estimate	Variance Inflation
Intercept	1	76.91902	3.56259	21.59	<.0001	0	0
A6	1	0.07552	0.11848	0.64	0.5239	0.01462	1.00768
age	1	-0.11638	0.05902	-1.97	0.0488	-0.04555	1.02179
BMI	1	-1.14254	0.08377	-13.64	<.0001	-0.33456	1.15221
SBP	1	0.00939	0.02958	0.32	0.7511	0.00776	1.14422
D1_al	1	-1.67407	0.73167	-2.29	0.0223	-0.05347	1.04581
D2_al	1	-0.82280	0.88254	-0.93	0.3513	-0.02164	1.03156
D1_sm	1	0.08451	1.23301	0.07	0.9454	0.00158	1.01390
D2_sm	1	0.38582	1.20371	0.32	0.7486	0.00744	1.03146

Linear Regression Results

The REG Procedure
Model: Linear_Regression_Model
Dependent Variable: LDL

Number of Observations Read	1701
Number of Observations Used	1701

Analysis of Variance					
Source	DF	Sum of Squares	Mean Square	F Value	Pr > F
Model	8	84537	10567	19.25	<.0001
Error	1692	929003	549.05630		
Corrected Total	1700	1013541			

Root MSE	23.43195	R-Square	0.0834
Dependent Mean	110.19089	Adj R-Sq	0.0791
Coeff Var	21.26487		

Parameter Estimates							
Variable	DF	Parameter Estimate	Standard Error	t Value	Pr > |t|	Standardized Estimate	Variance Inflation
Intercept	1	55.70820	7.62889	7.30	<.0001	0	0
A6	1	0.01972	0.25371	0.08	0.9381	0.00182	1.00768
age	1	1.11457	0.12639	8.82	<.0001	0.20748	1.02179
BMI	1	1.44960	0.17939	8.08	<.0001	0.20189	1.15221
SBP	1	-0.04320	0.06335	-0.68	0.4953	-0.01698	1.14422
D1_al	1	0.52343	1.56679	0.33	0.7384	0.00795	1.04581
D2_al	1	-0.03624	1.88985	-0.02	0.9847	-0.00045337	1.03156
D1_sm	1	-3.39218	2.64036	-1.28	0.1991	-0.03011	1.01390
D2_sm	1	-3.21520	2.57762	-1.25	0.2124	-0.02949	1.03146

Linear Regression Results

The REG Procedure
Model: Linear_Regression_Model
Dependent Variable: TG

Number of Observations Read	1701
Number of Observations Used	1701

Analysis of Variance					
Source	DF	Sum of Squares	Mean Square	F Value	Pr > F
Model	8	428184	53523	40.25	<.0001
Error	1692	2249833	1329.68828		
Corrected Total	1700	2678017			

Root MSE	36.46489	R-Square	0.1599
Dependent Mean	78.31805	Adj R-Sq	0.1559
Coeff Var	46.56001		

Parameter Estimates							
Variable	DF	Parameter Estimate	Standard Error	t Value	Pr > |t|	Standardized Estimate	Variance Inflation
Intercept	1	-53.02932	11.87212	-4.47	<.0001	0	0
A6	1	0.23375	0.39482	0.59	0.5539	0.01324	1.00768
age	1	0.45047	0.19668	2.29	0.0221	0.05159	1.02179
BMI	1	4.16966	0.27916	14.94	<.0001	0.35726	1.15221
SBP	1	0.23840	0.09858	2.42	0.0157	0.05764	1.14422
D1_al	1	6.73109	2.43825	2.76	0.0058	0.06291	1.04581
D2_al	1	5.35746	2.94100	1.82	0.0687	0.04123	1.03156
D1_sm	1	-1.82492	4.10894	-0.44	0.6570	-0.00997	1.01390
D2_sm	1	5.79525	4.01130	1.44	0.1487	0.03270	1.03146

Linear Regression Results

The REG Procedure
Model: Linear_Regression_Model
Dependent Variable: TC

Number of Observations Read	1701
Number of Observations Used	1701

Analysis of Variance					
Source	DF	Sum of Squares	Mean Square	F Value	Pr > F
Model	8	71069	8883.64356	11.88	<.0001
Error	1692	1265405	747.87546		
Corrected Total	1700	1336474			

Root MSE	27.34731	R-Square	0.0532
Dependent Mean	176.57202	Adj R-Sq	0.0487
Coeff Var	15.48791		

Parameter Estimates							
Variable	DF	Parameter Estimate	Standard Error	t Value	Pr > |t|	Standardized Estimate	Variance Inflation
Intercept	1	126.58188	9.12240	13.88	<.0001	0	0
C1	1	-0.29383	0.35819	-0.82	0.4121	-0.01948	1.00759
age	1	1.09078	0.14744	7.40	<.0001	0.17683	1.02093
BMI	1	1.14493	0.20932	5.47	<.0001	0.13886	1.15178
SBP	1	0.01073	0.07398	0.14	0.8847	0.00367	1.14584
D1_al	1	0.05771	1.82677	0.03	0.9748	0.00076349	1.04372
D2_al	1	0.10819	2.20888	0.05	0.9609	0.00118	1.03459
D1_sm	1	-3.70845	3.08084	-1.20	0.2289	-0.02867	1.01343
D2_sm	1	-1.58433	3.00891	-0.53	0.5986	-0.01265	1.03186

Linear Regression Results

The REG Procedure
Model: Linear_Regression_Model
Dependent Variable: HDL

Number of Observations Read	1701
Number of Observations Used	1701

Analysis of Variance					
Source	DF	Sum of Squares	Mean Square	F Value	Pr > F
Model	8	26935	3366.81724	28.15	<.0001
Error	1692	202338	119.58513		
Corrected Total	1700	229273			

Root MSE	10.93550	R-Square	0.1175
Dependent Mean	50.72310	Adj R-Sq	0.1133
Coeff Var	21.55921		

Parameter Estimates							
Variable	DF	Parameter Estimate	Standard Error	t Value	Pr > |t|	Standardized Estimate	Variance Inflation
Intercept	1	75.33482	3.64782	20.65	<.0001	0	0
C1	1	0.22856	0.14323	1.60	0.1107	0.03658	1.00759
age	1	-0.11775	0.05896	-2.00	0.0460	-0.04609	1.02093
BMI	1	-1.14645	0.08370	-13.70	<.0001	-0.33571	1.15178
SBP	1	0.01130	0.02958	0.38	0.7026	0.00934	1.14584
D1_al	1	-1.58886	0.73048	-2.18	0.0298	-0.05075	1.04372
D2_al	1	-0.72722	0.88327	-0.82	0.4104	-0.01913	1.03459
D1_sm	1	0.06030	1.23195	0.05	0.9610	0.00113	1.01343
D2_sm	1	0.32495	1.20319	0.27	0.7871	0.00627	1.03186

Linear Regression Results

The REG Procedure
Model: Linear_Regression_Model
Dependent Variable: LDL

Number of Observations Read	1701
Number of Observations Used	1701

Analysis of Variance					
Source	DF	Sum of Squares	Mean Square	F Value	Pr > F
Model	8	85478	10685	19.48	<.0001
Error	1692	928063	548.50058		
Corrected Total	1700	1013541			

Root MSE	23.42009	R-Square	0.0843
Dependent Mean	110.19089	Adj R-Sq	0.0800
Coeff Var	21.25411		

Parameter Estimates							
Variable	DF	Parameter Estimate	Standard Error	t Value	Pr > |t|	Standardized Estimate	Variance Inflation
Intercept	1	60.14160	7.81237	7.70	<.0001	0	0
C1	1	-0.40233	0.30675	-1.31	0.1898	-0.03063	1.00759
age	1	1.11477	0.12627	8.83	<.0001	0.20752	1.02093
BMI	1	1.45360	0.17926	8.11	<.0001	0.20245	1.15178
SBP	1	-0.04634	0.06336	-0.73	0.4647	-0.01821	1.14584
D1_al	1	0.43465	1.56443	0.28	0.7812	0.00660	1.04372
D2_al	1	-0.17856	1.89167	-0.09	0.9248	-0.00223	1.03459
D1_sm	1	-3.38419	2.63842	-1.28	0.1998	-0.03004	1.01343
D2_sm	1	-3.13278	2.57681	-1.22	0.2242	-0.02873	1.03186

Linear Regression Results

The REG Procedure
Model: Linear_Regression_Model
Dependent Variable: TG

Number of Observations Read	1701
Number of Observations Used	1701

Analysis of Variance					
Source	DF	Sum of Squares	Mean Square	F Value	Pr > F
Model	8	429946	53743	40.45	<.0001
Error	1692	2248071	1328.64703		
Corrected Total	1700	2678017			

Root MSE	36.45061	R-Square	0.1605
Dependent Mean	78.31805	Adj R-Sq	0.1566
Coeff Var	46.54178		

Parameter Estimates							
Variable	DF	Parameter Estimate	Standard Error	t Value	Pr > |t|	Standardized Estimate	Variance Inflation
Intercept	1	-44.02515	12.15904	-3.62	0.0003	0	0
C1	1	-0.61821	0.47742	-1.29	0.1955	-0.02895	1.00759
age	1	0.44782	0.19652	2.28	0.0228	0.05128	1.02093
BMI	1	4.17197	0.27900	14.95	<.0001	0.35745	1.15178
SBP	1	0.23390	0.09861	2.37	0.0178	0.05655	1.14584
D1_al	1	6.67625	2.43486	2.74	0.0062	0.06239	1.04372
D2_al	1	5.17336	2.94416	1.76	0.0791	0.03981	1.03459
D1_sm	1	-1.85882	4.10638	-0.45	0.6508	-0.01015	1.01343
D2_sm	1	5.88893	4.01050	1.47	0.1422	0.03322	1.03186

Linear Regression Results

The REG Procedure
Model: Linear_Regression_Model
Dependent Variable: TC

Number of Observations Read	1701
Number of Observations Used	1701

Analysis of Variance					
Source	DF	Sum of Squares	Mean Square	F Value	Pr > F
Model	8	70911	8863.85788	11.85	<.0001
Error	1692	1265564	747.96901		
Corrected Total	1700	1336474			

Root MSE	27.34902	R-Square	0.0531
Dependent Mean	176.57202	Adj R-Sq	0.0486
Coeff Var	15.48888		

Parameter Estimates							
Variable	DF	Parameter Estimate	Standard Error	t Value	Pr > |t|	Standardized Estimate	Variance Inflation
Intercept	1	121.70927	8.72079	13.96	<.0001	0	0
C2	1	0.20682	0.30452	0.68	0.4971	0.01623	1.02045
age	1	1.07768	0.14864	7.25	<.0001	0.17470	1.03747
BMI	1	1.14939	0.20960	5.48	<.0001	0.13940	1.15472
SBP	1	0.01185	0.07396	0.16	0.8727	0.00406	1.14482
D1_al	1	0.10337	1.82522	0.06	0.9548	0.00137	1.04183
D2_al	1	0.17864	2.20584	0.08	0.9355	0.00195	1.03162
D1_sm	1	-3.66560	3.08196	-1.19	0.2345	-0.02833	1.01404
D2_sm	1	-1.63182	3.00821	-0.54	0.5876	-0.01303	1.03126

Linear Regression Results

The REG Procedure
Model: Linear_Regression_Model
Dependent Variable: HDL

Number of Observations Read	1701
Number of Observations Used	1701

Analysis of Variance					
Source	DF	Sum of Squares	Mean Square	F Value	Pr > F
Model	8	26726	3340.69268	27.91	<.0001
Error	1692	202547	119.70865		
Corrected Total	1700	229273			

Root MSE	10.94114	R-Square	0.1166
Dependent Mean	50.72310	Adj R-Sq	0.1124
Coeff Var	21.57034		

Parameter Estimates							
Variable	DF	Parameter Estimate	Standard Error	t Value	Pr > |t|	Standardized Estimate	Variance Inflation
Intercept	1	78.67461	3.48881	22.55	<.0001	0	0
C2	1	-0.10883	0.12183	-0.89	0.3718	-0.02062	1.02045
age	1	-0.11077	0.05946	-1.86	0.0627	-0.04335	1.03747
BMI	1	-1.14799	0.08385	-13.69	<.0001	-0.33616	1.15472
SBP	1	0.01013	0.02959	0.34	0.7322	0.00837	1.14482
D1_al	1	-1.63065	0.73019	-2.23	0.0257	-0.05208	1.04183
D2_al	1	-0.79106	0.88246	-0.90	0.3702	-0.02080	1.03162
D1_sm	1	0.04004	1.23296	0.03	0.9741	0.00074729	1.01404
D2_sm	1	0.36567	1.20345	0.30	0.7613	0.00705	1.03126

Linear Regression Results

The REG Procedure
Model: Linear_Regression_Model
Dependent Variable: LDL

Number of Observations Read	1701
Number of Observations Used	1701

Analysis of Variance					
Source	DF	Sum of Squares	Mean Square	F Value	Pr > F
Model	8	84935	10617	19.34	<.0001
Error	1692	928605	548.82115		
Corrected Total	1700	1013541			

Root MSE	23.42693	R-Square	0.0838
Dependent Mean	110.19089	Adj R-Sq	0.0795
Coeff Var	21.26032		

Parameter Estimates							
Variable	DF	Parameter Estimate	Standard Error	t Value	Pr > |t|	Standardized Estimate	Variance Inflation
Intercept	1	53.99045	7.47015	7.23	<.0001	0	0
C2	1	0.22302	0.26085	0.85	0.3927	0.02010	1.02045
age	1	1.10054	0.12732	8.64	<.0001	0.20487	1.03747
BMI	1	1.45748	0.17954	8.12	<.0001	0.20299	1.15472
SBP	1	-0.04445	0.06335	-0.70	0.4829	-0.01747	1.14482
D1_al	1	0.50442	1.56347	0.32	0.7470	0.00766	1.04183
D2_al	1	-0.07165	1.88950	-0.04	0.9698	-0.00089617	1.03162
D1_sm	1	-3.34064	2.63998	-1.27	0.2059	-0.02965	1.01404
D2_sm	1	-3.20218	2.57681	-1.24	0.2142	-0.02937	1.03126

Linear Regression Results

The REG Procedure
Model: Linear_Regression_Model
Dependent Variable: TG

Number of Observations Read	1701
Number of Observations Used	1701

Analysis of Variance					
Source	DF	Sum of Squares	Mean Square	F Value	Pr > F
Model	8	429142	53643	40.36	<.0001
Error	1692	2248875	1329.12225		
Corrected Total	1700	2678017			

Root MSE	36.45713	R-Square	0.1602
Dependent Mean	78.31805	Adj R-Sq	0.1563
Coeff Var	46.55010		

Parameter Estimates							
Variable	DF	Parameter Estimate	Standard Error	t Value	Pr > |t|	Standardized Estimate	Variance Inflation
Intercept	1	-54.14717	11.62510	-4.66	<.0001	0	0
C2	1	0.42014	0.40594	1.03	0.3008	0.02329	1.02045
age	1	0.42118	0.19814	2.13	0.0337	0.04823	1.03747
BMI	1	4.18079	0.27941	14.96	<.0001	0.35821	1.15472
SBP	1	0.23635	0.09859	2.40	0.0166	0.05715	1.14482
D1_al	1	6.77412	2.43308	2.78	0.0054	0.06331	1.04183
D2_al	1	5.32417	2.94045	1.81	0.0704	0.04097	1.03162
D1_sm	1	-1.77245	4.10836	-0.43	0.6662	-0.00968	1.01404
D2_sm	1	5.78792	4.01004	1.44	0.1491	0.03265	1.03126

Linear Regression Results

The REG Procedure
Model: Linear_Regression_Model
Dependent Variable: TC

Number of Observations Read	1701
Number of Observations Used	1701

Analysis of Variance					
Source	DF	Sum of Squares	Mean Square	F Value	Pr > F
Model	8	70644	8830.47391	11.80	<.0001
Error	1692	1265831	748.12685		
Corrected Total	1700	1336474			

Root MSE	27.35191	R-Square	0.0529
Dependent Mean	176.57202	Adj R-Sq	0.0484
Coeff Var	15.49051		

Parameter Estimates							
Variable	DF	Parameter Estimate	Standard Error	t Value	Pr > |t|	Standardized Estimate	Variance Inflation
Intercept	1	124.53529	8.91210	13.97	<.0001	0	0
C3	1	-0.11635	0.36050	-0.32	0.7469	-0.00778	1.03835
age	1	1.09781	0.14923	7.36	<.0001	0.17797	1.04550
BMI	1	1.14003	0.20939	5.44	<.0001	0.13827	1.15212
SBP	1	0.01394	0.07400	0.19	0.8505	0.00477	1.14584
D1_al	1	0.17386	1.83049	0.09	0.9243	0.00230	1.04763
D2_al	1	0.21900	2.20548	0.10	0.9209	0.00239	1.03106
D1_sm	1	-3.76928	3.08551	-1.22	0.2220	-0.02914	1.01616
D2_sm	1	-1.72012	3.01700	-0.57	0.5687	-0.01374	1.03707

Linear Regression Results

The REG Procedure
Model: Linear_Regression_Model
Dependent Variable: HDL

Number of Observations Read	1701
Number of Observations Used	1701

Analysis of Variance					
Source	DF	Sum of Squares	Mean Square	F Value	Pr > F
Model	8	26646	3330.78573	27.81	<.0001
Error	1692	202626	119.75549		
Corrected Total	1700	229273			

Root MSE	10.94329	R-Square	0.1162
Dependent Mean	50.72310	Adj R-Sq	0.1120
Coeff Var	21.57456		

Parameter Estimates							
Variable	DF	Parameter Estimate	Standard Error	t Value	Pr > |t|	Standardized Estimate	Variance Inflation
Intercept	1	77.25923	3.56566	21.67	<.0001	0	0
C3	1	0.05317	0.14423	0.37	0.7124	0.00859	1.03835
age	1	-0.12085	0.05971	-2.02	0.0431	-0.04730	1.04550
BMI	1	-1.14318	0.08377	-13.65	<.0001	-0.33475	1.15212
SBP	1	0.00909	0.02961	0.31	0.7590	0.00751	1.14584
D1_al	1	-1.66459	0.73236	-2.27	0.0232	-0.05317	1.04763
D2_al	1	-0.81199	0.88240	-0.92	0.3576	-0.02136	1.03106
D1_sm	1	0.09102	1.23449	0.07	0.9412	0.00170	1.01616
D2_sm	1	0.40707	1.20708	0.34	0.7360	0.00785	1.03707

Linear Regression Results

The REG Procedure
Model: Linear_Regression_Model
Dependent Variable: LDL

Number of Observations Read	1701
Number of Observations Used	1701

Analysis of Variance					
Source	DF	Sum of Squares	Mean Square	F Value	Pr > F
Model	8	84917	10615	19.34	<.0001
Error	1692	928624	548.83188		
Corrected Total	1700	1013541			

Root MSE	23.42716	R-Square	0.0838
Dependent Mean	110.19089	Adj R-Sq	0.0795
Coeff Var	21.26052		

Parameter Estimates							
Variable	DF	Parameter Estimate	Standard Error	t Value	Pr > |t|	Standardized Estimate	Variance Inflation
Intercept	1	58.21763	7.63330	7.63	<.0001	0	0
C3	1	-0.25795	0.30877	-0.84	0.4036	-0.01981	1.03835
age	1	1.13066	0.12782	8.85	<.0001	0.21047	1.04550
BMI	1	1.44544	0.17934	8.06	<.0001	0.20131	1.15212
SBP	1	-0.04116	0.06338	-0.65	0.5162	-0.01618	1.14584
D1_al	1	0.63228	1.56783	0.40	0.6868	0.00961	1.04763
D2_al	1	-0.02309	1.88901	-0.01	0.9902	-0.00028884	1.03106
D1_sm	1	-3.51134	2.64276	-1.33	0.1841	-0.03117	1.01616
D2_sm	1	-3.38082	2.58409	-1.31	0.1909	-0.03100	1.03707

Linear Regression Results

The REG Procedure
Model: Linear_Regression_Model
Dependent Variable: TG

Number of Observations Read	1701
Number of Observations Used	1701

Analysis of Variance					
Source	DF	Sum of Squares	Mean Square	F Value	Pr > F
Model	8	428841	53605	40.33	<.0001
Error	1692	2249176	1329.30030		
Corrected Total	1700	2678017			

Root MSE	36.45957	R-Square	0.1601
Dependent Mean	78.31805	Adj R-Sq	0.1562
Coeff Var	46.55322		

Parameter Estimates							
Variable	DF	Parameter Estimate	Standard Error	t Value	Pr > |t|	Standardized Estimate	Variance Inflation
Intercept	1	-54.44307	11.87966	-4.58	<.0001	0	0
C3	1	0.44158	0.48054	0.92	0.3583	0.02086	1.03835
age	1	0.41905	0.19892	2.11	0.0353	0.04799	1.04550
BMI	1	4.17172	0.27911	14.95	<.0001	0.35743	1.15212
SBP	1	0.23531	0.09864	2.39	0.0172	0.05689	1.14584
D1_al	1	6.65202	2.44001	2.73	0.0065	0.06217	1.04763
D2_al	1	5.38039	2.93986	1.83	0.0674	0.04140	1.03106
D1_sm	1	-1.68163	4.11292	-0.41	0.6827	-0.00918	1.01616
D2_sm	1	6.03543	4.02160	1.50	0.1336	0.03405	1.03707

Linear Regression Results

The REG Procedure
Model: Linear_Regression_Model
Dependent Variable: TC

Number of Observations Read	1701
Number of Observations Used	1701

Analysis of Variance					
Source	DF	Sum of Squares	Mean Square	F Value	Pr > F
Model	8	70579	8822.37435	11.79	<.0001
Error	1692	1265895	748.16515		
Corrected Total	1700	1336474			

Root MSE	27.35261	R-Square	0.0528
Dependent Mean	176.57202	Adj R-Sq	0.0483
Coeff Var	15.49091		

Parameter Estimates							
Variable	DF	Parameter Estimate	Standard Error	t Value	Pr > |t|	Standardized Estimate	Variance Inflation
Intercept	1	123.03024	9.03644	13.61	<.0001	0	0
C4	1	0.04214	0.31810	0.13	0.8946	0.00316	1.01769
age	1	1.09002	0.14750	7.39	<.0001	0.17670	1.02139
BMI	1	1.14462	0.21046	5.44	<.0001	0.13883	1.16387
SBP	1	0.01307	0.07395	0.18	0.8597	0.00447	1.14419
D1_al	1	0.12045	1.82606	0.07	0.9474	0.00159	1.04251
D2_al	1	0.20374	2.20701	0.09	0.9265	0.00222	1.03244
D1_sm	1	-3.70332	3.08329	-1.20	0.2299	-0.02863	1.01465
D2_sm	1	-1.63593	3.00965	-0.54	0.5868	-0.01306	1.03198

Linear Regression Results

The REG Procedure
Model: Linear_Regression_Model
Dependent Variable: HDL

Number of Observations Read	1701
Number of Observations Used	1701

Analysis of Variance					
Source	DF	Sum of Squares	Mean Square	F Value	Pr > F
Model	8	27065	3383.07692	28.31	<.0001
Error	1692	202208	119.50825		
Corrected Total	1700	229273			

Root MSE	10.93198	R-Square	0.1180
Dependent Mean	50.72310	Adj R-Sq	0.1139
Coeff Var	21.55228		

Parameter Estimates							
Variable	DF	Parameter Estimate	Standard Error	t Value	Pr > |t|	Standardized Estimate	Variance Inflation
Intercept	1	75.03447	3.61158	20.78	<.0001	0	0
C4	1	0.24244	0.12713	1.91	0.0567	0.04392	1.01769
age	1	-0.11986	0.05895	-2.03	0.0422	-0.04691	1.02139
BMI	1	-1.12735	0.08411	-13.40	<.0001	-0.33012	1.16387
SBP	1	0.00969	0.02955	0.33	0.7429	0.00801	1.14419
D1_al	1	-1.68912	0.72982	-2.31	0.0208	-0.05395	1.04251
D2_al	1	-0.87229	0.88207	-0.99	0.3228	-0.02294	1.03244
D1_sm	1	0.14923	1.23229	0.12	0.9036	0.00278	1.01465
D2_sm	1	0.43645	1.20286	0.36	0.7168	0.00842	1.03198

Linear Regression Results

The REG Procedure
Model: Linear_Regression_Model
Dependent Variable: LDL

Number of Observations Read	1701
Number of Observations Used	1701

Analysis of Variance					
Source	DF	Sum of Squares	Mean Square	F Value	Pr > F
Model	8	85061	10633	19.38	<.0001
Error	1692	928480	548.74675		
Corrected Total	1700	1013541			

Root MSE	23.42534	R-Square	0.0839
Dependent Mean	110.19089	Adj R-Sq	0.0796
Coeff Var	21.25888		

Parameter Estimates							
Variable	DF	Parameter Estimate	Standard Error	t Value	Pr > |t|	Standardized Estimate	Variance Inflation
Intercept	1	58.89211	7.73900	7.61	<.0001	0	0
C4	1	-0.26699	0.27243	-0.98	0.3272	-0.02300	1.01769
age	1	1.11692	0.12633	8.84	<.0001	0.20792	1.02139
BMI	1	1.43093	0.18024	7.94	<.0001	0.19929	1.16387
SBP	1	-0.04339	0.06333	-0.69	0.4934	-0.01705	1.14419
D1_al	1	0.58127	1.56388	0.37	0.7102	0.00883	1.04251
D2_al	1	0.03574	1.89013	0.02	0.9849	0.00044706	1.03244
D1_sm	1	-3.48679	2.64059	-1.32	0.1869	-0.03095	1.01465
D2_sm	1	-3.28762	2.57753	-1.28	0.2023	-0.03015	1.03198

Linear Regression Results

The REG Procedure
Model: Linear_Regression_Model
Dependent Variable: TG

Number of Observations Read	1701
Number of Observations Used	1701

Analysis of Variance					
Source	DF	Sum of Squares	Mean Square	F Value	Pr > F
Model	8	428119	53515	40.24	<.0001
Error	1692	2249898	1329.72719		
Corrected Total	1700	2678017			

Root MSE	36.46542	R-Square	0.1599
Dependent Mean	78.31805	Adj R-Sq	0.1559
Coeff Var	46.56069		

Parameter Estimates							
Variable	DF	Parameter Estimate	Standard Error	t Value	Pr > |t|	Standardized Estimate	Variance Inflation
Intercept	1	-53.10019	12.04703	-4.41	<.0001	0	0
C4	1	0.23266	0.42408	0.55	0.5833	0.01233	1.01769
age	1	0.44478	0.19665	2.26	0.0238	0.05094	1.02139
BMI	1	4.18119	0.28057	14.90	<.0001	0.35824	1.16387
SBP	1	0.23894	0.09858	2.42	0.0155	0.05777	1.14419
D1_al	1	6.78132	2.43443	2.79	0.0054	0.06338	1.04251
D2_al	1	5.33737	2.94230	1.81	0.0699	0.04107	1.03244
D1_sm	1	-1.79942	4.11052	-0.44	0.6616	-0.00983	1.01465
D2_sm	1	5.81770	4.01235	1.45	0.1473	0.03282	1.03198

Linear Regression Results

The REG Procedure
Model: Linear_Regression_Model
Dependent Variable: TC

Number of Observations Read	1701
Number of Observations Used	1701

Analysis of Variance					
Source	DF	Sum of Squares	Mean Square	F Value	Pr > F
Model	8	70760	8844.94395	11.82	<.0001
Error	1692	1265715	748.05844		
Corrected Total	1700	1336474			

Root MSE	27.35066	R-Square	0.0529
Dependent Mean	176.57202	Adj R-Sq	0.0485
Coeff Var	15.48980		

Parameter Estimates							
Variable	DF	Parameter Estimate	Standard Error	t Value	Pr > |t|	Standardized Estimate	Variance Inflation
Intercept	1	122.11937	8.74459	13.97	<.0001	0	0
C5	1	0.16193	0.31823	0.51	0.6109	0.01220	1.02777
age	1	1.08033	0.14879	7.26	<.0001	0.17513	1.03944
BMI	1	1.15154	0.21020	5.48	<.0001	0.13966	1.16114
SBP	1	0.01135	0.07401	0.15	0.8781	0.00389	1.14647
D1_al	1	0.11069	1.82529	0.06	0.9517	0.00146	1.04178
D2_al	1	0.23073	2.20556	0.10	0.9167	0.00251	1.03124
D1_sm	1	-3.67795	3.08218	-1.19	0.2329	-0.02843	1.01407
D2_sm	1	-1.62640	3.00858	-0.54	0.5889	-0.01299	1.03138

Linear Regression Results

The REG Procedure
Model: Linear_Regression_Model
Dependent Variable: HDL

Number of Observations Read	1701
Number of Observations Used	1701

Analysis of Variance					
Source	DF	Sum of Squares	Mean Square	F Value	Pr > F
Model	8	26676	3334.50921	27.85	<.0001
Error	1692	202597	119.73789		
Corrected Total	1700	229273			

Root MSE	10.94248	R-Square	0.1164
Dependent Mean	50.72310	Adj R-Sq	0.1122
Coeff Var	21.57297		

Parameter Estimates							
Variable	DF	Parameter Estimate	Standard Error	t Value	Pr > |t|	Standardized Estimate	Variance Inflation
Intercept	1	77.05979	3.49855	22.03	<.0001	0	0
C5	1	0.07897	0.12732	0.62	0.5352	0.01437	1.02777
age	1	-0.12240	0.05953	-2.06	0.0399	-0.04791	1.03944
BMI	1	-1.13918	0.08410	-13.55	<.0001	-0.33358	1.16114
SBP	1	0.00868	0.02961	0.29	0.7694	0.00717	1.14647
D1_al	1	-1.65239	0.73026	-2.26	0.0238	-0.05278	1.04178
D2_al	1	-0.80209	0.88240	-0.91	0.3635	-0.02109	1.03124
D1_sm	1	0.08668	1.23312	0.07	0.9440	0.00162	1.01407
D2_sm	1	0.38356	1.20368	0.32	0.7500	0.00740	1.03138

Linear Regression Results

The REG Procedure
Model: Linear_Regression_Model
Dependent Variable: LDL

Number of Observations Read	1701
Number of Observations Used	1701

Analysis of Variance					
Source	DF	Sum of Squares	Mean Square	F Value	Pr > F
Model	8	84541	10568	19.25	<.0001
Error	1692	929000	549.05430		
Corrected Total	1700	1013541			

Root MSE	23.43191	R-Square	0.0834
Dependent Mean	110.19089	Adj R-Sq	0.0791
Coeff Var	21.26483		

Parameter Estimates							
Variable	DF	Parameter Estimate	Standard Error	t Value	Pr > |t|	Standardized Estimate	Variance Inflation
Intercept	1	55.66414	7.49169	7.43	<.0001	0	0
C5	1	0.03010	0.27264	0.11	0.9121	0.00261	1.02777
age	1	1.11241	0.12747	8.73	<.0001	0.20708	1.03944
BMI	1	1.45105	0.18008	8.06	<.0001	0.20209	1.16114
SBP	1	-0.04349	0.06341	-0.69	0.4929	-0.01709	1.14647
D1_al	1	0.52806	1.56377	0.34	0.7356	0.00802	1.04178
D2_al	1	-0.02989	1.88956	-0.02	0.9874	-0.00037388	1.03124
D1_sm	1	-3.38929	2.64057	-1.28	0.1995	-0.03008	1.01407
D2_sm	1	-3.21459	2.57751	-1.25	0.2125	-0.02948	1.03138

Linear Regression Results

The REG Procedure
Model: Linear_Regression_Model
Dependent Variable: TG

Number of Observations Read	1701
Number of Observations Used	1701

Analysis of Variance					
Source	DF	Sum of Squares	Mean Square	F Value	Pr > F
Model	8	427965	53496	40.23	<.0001
Error	1692	2250052	1329.81790		
Corrected Total	1700	2678017			

Root MSE	36.46667	R-Square	0.1598
Dependent Mean	78.31805	Adj R-Sq	0.1558
Coeff Var	46.56228		

Parameter Estimates							
Variable	DF	Parameter Estimate	Standard Error	t Value	Pr > |t|	Standardized Estimate	Variance Inflation
Intercept	1	-52.06831	11.65918	-4.47	<.0001	0	0
C5	1	0.18277	0.42430	0.43	0.6667	0.00973	1.02777
age	1	0.43567	0.19838	2.20	0.0282	0.04989	1.03944
BMI	1	4.17631	0.28025	14.90	<.0001	0.35783	1.16114
SBP	1	0.23686	0.09868	2.40	0.0165	0.05727	1.14647
D1_al	1	6.80491	2.43366	2.80	0.0052	0.06360	1.04178
D2_al	1	5.41542	2.94068	1.84	0.0657	0.04167	1.03124
D1_sm	1	-1.83328	4.10948	-0.45	0.6556	-0.01001	1.01407
D2_sm	1	5.78046	4.01134	1.44	0.1498	0.03261	1.03138

Linear Regression Results

The REG Procedure
Model: Linear_Regression_Model
Dependent Variable: TC

Number of Observations Read	1701
Number of Observations Used	1701

Analysis of Variance					
Source	DF	Sum of Squares	Mean Square	F Value	Pr > F
Model	8	70930	8866.23805	11.85	<.0001
Error	1692	1265545	747.95776		
Corrected Total	1700	1336474			

Root MSE	27.34882	R-Square	0.0531
Dependent Mean	176.57202	Adj R-Sq	0.0486
Coeff Var	15.48876		

Parameter Estimates							
Variable	DF	Parameter Estimate	Standard Error	t Value	Pr > |t|	Standardized Estimate	Variance Inflation
Intercept	1	125.77310	8.92906	14.09	<.0001	0	0
C6	1	-0.21778	0.31216	-0.70	0.4855	-0.01659	1.01080
age	1	1.09249	0.14748	7.41	<.0001	0.17710	1.02133
BMI	1	1.13549	0.20949	5.42	<.0001	0.13772	1.15349
SBP	1	0.01363	0.07394	0.18	0.8538	0.00466	1.14433
D1_al	1	0.17582	1.82611	0.10	0.9233	0.00233	1.04285
D2_al	1	0.17556	2.20590	0.08	0.9366	0.00191	1.03169
D1_sm	1	-3.79611	3.08305	-1.23	0.2184	-0.02934	1.01477
D2_sm	1	-1.78380	3.01451	-0.59	0.5541	-0.01425	1.03559

Linear Regression Results

The REG Procedure
Model: Linear_Regression_Model
Dependent Variable: HDL

Number of Observations Read	1701
Number of Observations Used	1701

Analysis of Variance					
Source	DF	Sum of Squares	Mean Square	F Value	Pr > F
Model	8	26634	3329.27154	27.80	<.0001
Error	1692	202638	119.76265		
Corrected Total	1700	229273			

Root MSE	10.94361	R-Square	0.1162
Dependent Mean	50.72310	Adj R-Sq	0.1120
Coeff Var	21.57520		

Parameter Estimates							
Variable	DF	Parameter Estimate	Standard Error	t Value	Pr > |t|	Standardized Estimate	Variance Inflation
Intercept	1	77.97583	3.57296	21.82	<.0001	0	0
C6	1	-0.02329	0.12491	-0.19	0.8521	-0.00428	1.01080
age	1	-0.11726	0.05901	-1.99	0.0471	-0.04589	1.02133
BMI	1	-1.14463	0.08383	-13.65	<.0001	-0.33518	1.15349
SBP	1	0.00956	0.02959	0.32	0.7465	0.00790	1.14433
D1_al	1	-1.63871	0.73072	-2.24	0.0251	-0.05234	1.04285
D2_al	1	-0.81414	0.88269	-0.92	0.3565	-0.02141	1.03169
D1_sm	1	0.05897	1.23368	0.05	0.9619	0.00110	1.01477
D2_sm	1	0.35894	1.20625	0.30	0.7661	0.00692	1.03559

Linear Regression Results

The REG Procedure
Model: Linear_Regression_Model
Dependent Variable: LDL

Number of Observations Read	1701
Number of Observations Used	1701

Analysis of Variance					
Source	DF	Sum of Squares	Mean Square	F Value	Pr > F
Model	8	85580	10698	19.51	<.0001
Error	1692	927960	548.43979		
Corrected Total	1700	1013541			

Root MSE	23.41879	R-Square	0.0844
Dependent Mean	110.19089	Adj R-Sq	0.0801
Coeff Var	21.25293		

Parameter Estimates							
Variable	DF	Parameter Estimate	Standard Error	t Value	Pr > |t|	Standardized Estimate	Variance Inflation
Intercept	1	59.77589	7.64595	7.82	<.0001	0	0
C6	1	-0.36923	0.26730	-1.38	0.1674	-0.03231	1.01080
age	1	1.11777	0.12629	8.85	<.0001	0.20808	1.02133
BMI	1	1.43864	0.17938	8.02	<.0001	0.20036	1.15349
SBP	1	-0.04217	0.06331	-0.67	0.5055	-0.01657	1.14433
D1_al	1	0.61186	1.56370	0.39	0.6956	0.00930	1.04285
D2_al	1	-0.09905	1.88891	-0.05	0.9582	-0.00124	1.03169
D1_sm	1	-3.52986	2.64001	-1.34	0.1814	-0.03133	1.01477
D2_sm	1	-3.45058	2.58132	-1.34	0.1815	-0.03164	1.03559

Linear Regression Results

The REG Procedure
Model: Linear_Regression_Model
Dependent Variable: TG

Number of Observations Read	1701
Number of Observations Used	1701

Analysis of Variance					
Source	DF	Sum of Squares	Mean Square	F Value	Pr > F
Model	8	433840	54230	40.89	<.0001
Error	1692	2244177	1326.34563		
Corrected Total	1700	2678017			

Root MSE	36.41903	R-Square	0.1620
Dependent Mean	78.31805	Adj R-Sq	0.1580
Coeff Var	46.50145		

Parameter Estimates							
Variable	DF	Parameter Estimate	Standard Error	t Value	Pr > |t|	Standardized Estimate	Variance Inflation
Intercept	1	-59.83548	11.89037	-5.03	<.0001	0	0
C6	1	0.89305	0.41569	2.15	0.0318	0.04807	1.01080
age	1	0.43865	0.19639	2.23	0.0256	0.05023	1.02133
BMI	1	4.19084	0.27896	15.02	<.0001	0.35907	1.15349
SBP	1	0.23632	0.09846	2.40	0.0165	0.05714	1.14433
D1_al	1	6.63005	2.43173	2.73	0.0065	0.06196	1.04285
D2_al	1	5.55718	2.93748	1.89	0.0587	0.04276	1.03169
D1_sm	1	-1.55578	4.10554	-0.38	0.7048	-0.00850	1.01477
D2_sm	1	6.31897	4.01426	1.57	0.1156	0.03565	1.03559
