# Supplementary material for: Association between Blood Lipid Levels and Personality Traits in Young Korean Women
Source: PLoS One. 2014 Sep 30;9(9):e108406. doi: 10.1371/journal.pone.0108406 (PMC4182467; doi:10.1371/journal.pone.0108406)
Supplement: Logistic Regression S1 — The results of the logistic regression using SAS program. Response variables are TC_x (total cholesterol), TG_x (triglyceride), HDL_x, and LDL_x applying clinical criteria. The group “0” and “1” represents normal and abnormal groups, respectively. (PDF) [file pone.0108406.s004.pdf]

Logistic Regression Results

The LOGISTIC Procedure

Model Information	
Data Set	WORK.SORTTEMPTABLESORTED
Response Variable	TC_x
Number of Response Levels	2
Model	binary logit
Optimization Technique	Fisher's scoring

Number of Observations Read	1701
Number of Observations Used	1701

Response Profile		
Ordered Value	TC_x	Total Frequency
1	0	1386
2	1	315

Probability modeled is TC_x='1'.

Model Convergence Status
Convergence criterion (GCONV=1E-8) satisfied.

Model Fit Statistics		
Criterion	Intercept Only	Intercept and Covariates
AIC	1632.121	1583.097
SC	1637.560	1632.048
-2 Log L	1630.121	1565.097

Testing Global Null Hypothesis: BETA=0			
Test	Chi-Square	DF	Pr > ChiSq
Likelihood Ratio	65.0242	8	<.0001
Score	69.7838	8	<.0001
Wald	64.3704	8	<.0001

Logistic Regression Results

The LOGISTIC Procedure

Analysis of Maximum Likelihood Estimates					
Parameter	DF	Estimate	Standard Error	Wald Chi-Square	Pr > ChiSq
Intercept	1	-4.4732	0.8769	26.0208	<.0001
N	1	-0.00356	0.00662	0.2894	0.5906
age	1	0.0594	0.0138	18.6015	<.0001
BMI	1	0.1147	0.0187	37.6359	<.0001
SBP	1	-0.00839	0.00701	1.4329	0.2313
D1_al	1	0.1658	0.1743	0.9051	0.3414
D2_al	1	0.3071	0.1980	2.4060	0.1209
D1_sm	1	0.1634	0.2805	0.3391	0.5603
D2_sm	1	-0.0979	0.2860	0.1173	0.7320

Odds Ratio Estimates			
Effect	Point Estimate	95% Wald Confidence Limits	
N	0.996	0.984	1.009
age	1.061	1.033	1.090
BMI	1.122	1.081	1.163
SBP	0.992	0.978	1.005
D1_al	1.180	0.839	1.661
D2_al	1.359	0.922	2.004
D1_sm	1.177	0.679	2.041
D2_sm	0.907	0.518	1.588

Association of Predicted Probabilities and Observed Responses			
Percent Concordant	62.0	Somers' D	0.250
Percent Discordant	37.1	Gamma	0.252
Percent Tied	0.9	Tau-a	0.075
Pairs	436590	c	0.625

Logistic Regression Results

The LOGISTIC Procedure

Model Information	
Data Set	WORK.SORTTEMPTABLESORTED
Response Variable	TC_x
Number of Response Levels	2
Model	binary logit
Optimization Technique	Fisher's scoring

Number of Observations Read	1701
Number of Observations Used	1701

Response Profile		
Ordered Value	TC_x	Total Frequency
1	0	1386
2	1	315

Probability modeled is TC_x='1'.

Model Convergence Status
Convergence criterion (GCONV=1E-8) satisfied.

Model Fit Statistics		
Criterion	Intercept Only	Intercept and Covariates
AIC	1632.121	1583.175
SC	1637.560	1632.126
-2 Log L	1630.121	1565.175

Testing Global Null Hypothesis: BETA=0			
Test	Chi-Square	DF	Pr > ChiSq
Likelihood Ratio	64.9467	8	<.0001
Score	69.6349	8	<.0001
Wald	64.3684	8	<.0001

Logistic Regression Results

The LOGISTIC Procedure

Analysis of Maximum Likelihood Estimates					
Parameter	DF	Estimate	Standard Error	Wald Chi-Square	Pr > ChiSq
Intercept	1	-4.5025	0.8807	26.1388	<.0001
E	1	-0.00309	0.00670	0.2123	0.6450
age	1	0.0601	0.0136	19.5003	<.0001
BMI	1	0.1141	0.0186	37.5422	<.0001
SBP	1	-0.00832	0.00700	1.4095	0.2351
D1_al	1	0.1668	0.1741	0.9174	0.3381
D2_al	1	0.2976	0.1979	2.2616	0.1326
D1_sm	1	0.1622	0.2809	0.3335	0.5636
D2_sm	1	-0.0975	0.2858	0.1164	0.7330

Odds Ratio Estimates			
Effect	Point Estimate	95% Wald Confidence Limits	
E	0.997	0.984	1.010
age	1.062	1.034	1.091
BMI	1.121	1.081	1.163
SBP	0.992	0.978	1.005
D1_al	1.182	0.840	1.662
D2_al	1.347	0.914	1.985
D1_sm	1.176	0.678	2.039
D2_sm	0.907	0.518	1.588

Association of Predicted Probabilities and Observed Responses			
Percent Concordant	62.0	Somers' D	0.248
Percent Discordant	37.1	Gamma	0.250
Percent Tied	0.9	Tau-a	0.075
Pairs	436590	c	0.624

Logistic Regression Results

The LOGISTIC Procedure

Model Information	
Data Set	WORK.SORTTEMPTABLESORTED
Response Variable	TC_x
Number of Response Levels	2
Model	binary logit
Optimization Technique	Fisher's scoring

Number of Observations Read	1701
Number of Observations Used	1701

Response Profile		
Ordered Value	TC_x	Total Frequency
1	0	1386
2	1	315

Probability modeled is TC_x='1'.

Model Convergence Status
Convergence criterion (GCONV=1E-8) satisfied.

Model Fit Statistics		
Criterion	Intercept Only	Intercept and Covariates
AIC	1632.121	1583.068
SC	1637.560	1632.019
-2 Log L	1630.121	1565.068

Testing Global Null Hypothesis: BETA=0			
Test	Chi-Square	DF	Pr > ChiSq
Likelihood Ratio	65.0533	8	<.0001
Score	69.7359	8	<.0001
Wald	64.4643	8	<.0001

Logistic Regression Results

The LOGISTIC Procedure

Analysis of Maximum Likelihood Estimates					
Parameter	DF	Estimate	Standard Error	Wald Chi-Square	Pr > ChiSq
Intercept	1	-4.3870	0.9481	21.4120	<.0001
O	1	-0.00456	0.00807	0.3190	0.5722
age	1	0.0602	0.0136	19.6343	<.0001
BMI	1	0.1139	0.0186	37.3972	<.0001
SBP	1	-0.00838	0.00701	1.4295	0.2318
D1_al	1	0.1649	0.1742	0.8955	0.3440
D2_al	1	0.2964	0.1980	2.2416	0.1343
D1_sm	1	0.1614	0.2807	0.3305	0.5654
D2_sm	1	-0.0927	0.2860	0.1051	0.7458

Odds Ratio Estimates			
Effect	Point Estimate	95% Wald Confidence Limits	
O	0.995	0.980	1.011
age	1.062	1.034	1.091
BMI	1.121	1.080	1.162
SBP	0.992	0.978	1.005
D1_al	1.179	0.838	1.659
D2_al	1.345	0.912	1.983
D1_sm	1.175	0.678	2.037
D2_sm	0.911	0.520	1.597

Association of Predicted Probabilities and Observed Responses			
Percent Concordant	62.1	Somers' D	0.251
Percent Discordant	37.0	Gamma	0.253
Percent Tied	0.9	Tau-a	0.076
Pairs	436590	c	0.626

Logistic Regression Results

The LOGISTIC Procedure

Model Information	
Data Set	WORK.SORTTEMPTABLESORTED
Response Variable	TC_x
Number of Response Levels	2
Model	binary logit
Optimization Technique	Fisher's scoring

Number of Observations Read	1701
Number of Observations Used	1701

Response Profile		
Ordered Value	TC_x	Total Frequency
1	0	1386
2	1	315

Probability modeled is TC_x='1'.

Model Convergence Status
Convergence criterion (GCONV=1E-8) satisfied.

Model Fit Statistics		
Criterion	Intercept Only	Intercept and Covariates
AIC	1632.121	1582.158
SC	1637.560	1631.109
-2 Log L	1630.121	1564.158

Testing Global Null Hypothesis: BETA=0			
Test	Chi-Square	DF	Pr > ChiSq
Likelihood Ratio	65.9636	8	<.0001
Score	70.3804	8	<.0001
Wald	65.1607	8	<.0001

Logistic Regression Results

The LOGISTIC Procedure

Analysis of Maximum Likelihood Estimates					
Parameter	DF	Estimate	Standard Error	Wald Chi-Square	Pr > ChiSq
Intercept	1	-4.2097	0.8864	22.5527	<.0001
A	1	-0.00958	0.00863	1.2305	0.2673
age	1	0.0628	0.0137	21.0476	<.0001
BMI	1	0.1132	0.0186	36.8591	<.0001
SBP	1	-0.00793	0.00701	1.2788	0.2581
D1_al	1	0.1833	0.1746	1.1025	0.2937
D2_al	1	0.3111	0.1979	2.4696	0.1161
D1_sm	1	0.1521	0.2808	0.2932	0.5882
D2_sm	1	-0.1284	0.2870	0.2000	0.6548

Odds Ratio Estimates			
Effect	Point Estimate	95% Wald Confidence Limits	
A	0.990	0.974	1.007
age	1.065	1.037	1.094
BMI	1.120	1.080	1.161
SBP	0.992	0.979	1.006
D1_al	1.201	0.853	1.691
D2_al	1.365	0.926	2.012
D1_sm	1.164	0.671	2.019
D2_sm	0.880	0.501	1.544

Association of Predicted Probabilities and Observed Responses			
Percent Concordant	62.3	Somers' D	0.254
Percent Discordant	36.9	Gamma	0.257
Percent Tied	0.8	Tau-a	0.077
Pairs	436590	c	0.627

Logistic Regression Results

The LOGISTIC Procedure

Model Information	
Data Set	WORK.SORTTEMPTABLESORTED
Response Variable	TC_x
Number of Response Levels	2
Model	binary logit
Optimization Technique	Fisher's scoring

Number of Observations Read	1701
Number of Observations Used	1701

Response Profile		
Ordered Value	TC_x	Total Frequency
1	0	1386
2	1	315

Probability modeled is TC_x='1'.

Model Convergence Status
Convergence criterion (GCONV=1E-8) satisfied.

Model Fit Statistics		
Criterion	Intercept Only	Intercept and Covariates
AIC	1632.121	1578.054
SC	1637.560	1627.004
-2 Log L	1630.121	1560.054

Testing Global Null Hypothesis: BETA=0			
Test	Chi-Square	DF	Pr > ChiSq
Likelihood Ratio	70.0677	8	<.0001
Score	74.3309	8	<.0001
Wald	68.9040	8	<.0001

Logistic Regression Results

The LOGISTIC Procedure

Analysis of Maximum Likelihood Estimates					
Parameter	DF	Estimate	Standard Error	Wald Chi-Square	Pr > ChiSq
Intercept	1	-3.7047	0.8802	17.7158	<.0001
C	1	-0.0178	0.00772	5.3172	0.0211
age	1	0.0651	0.0137	22.5832	<.0001
BMI	1	0.1109	0.0187	35.2552	<.0001
SBP	1	-0.00798	0.00703	1.2867	0.2567
D1_al	1	0.1867	0.1744	1.1458	0.2844
D2_al	1	0.2946	0.1984	2.2057	0.1375
D1_sm	1	0.1374	0.2813	0.2387	0.6251
D2_sm	1	-0.1187	0.2847	0.1738	0.6768

Odds Ratio Estimates			
Effect	Point Estimate	95% Wald Confidence Limits	
C	0.982	0.968	0.997
age	1.067	1.039	1.096
BMI	1.117	1.077	1.159
SBP	0.992	0.978	1.006
D1_al	1.205	0.856	1.697
D2_al	1.343	0.910	1.981
D1_sm	1.147	0.661	1.991
D2_sm	0.888	0.508	1.552

Association of Predicted Probabilities and Observed Responses			
Percent Concordant	62.7	Somers' D	0.262
Percent Discordant	36.5	Gamma	0.264
Percent Tied	0.8	Tau-a	0.079
Pairs	436590	c	0.631

Logistic Regression Results

The LOGISTIC Procedure

Model Information	
Data Set	WORK.SORTTEMPTABLESORTED
Response Variable	HDL_x
Number of Response Levels	2
Model	binary logit
Optimization Technique	Fisher's scoring

Number of Observations Read	1701
Number of Observations Used	1701

Response Profile		
Ordered Value	HDL_x	Total Frequency
1	0	1410
2	1	291

Probability modeled is HDL_x='1'.

Model Convergence Status
Convergence criterion (GCONV=1E-8) satisfied.

Model Fit Statistics		
Criterion	Intercept Only	Intercept and Covariates
AIC	1558.714	1411.650
SC	1564.153	1460.601
-2 Log L	1556.714	1393.650

Testing Global Null Hypothesis: BETA=0			
Test	Chi-Square	DF	Pr > ChiSq
Likelihood Ratio	163.0645	8	<.0001
Score	186.0353	8	<.0001
Wald	146.2729	8	<.0001

Logistic Regression Results

The LOGISTIC Procedure

Analysis of Maximum Likelihood Estimates					
Parameter	DF	Estimate	Standard Error	Wald Chi-Square	Pr > ChiSq
Intercept	1	-6.9019	0.9359	54.3838	<.0001
N	1	0.00905	0.00703	1.6561	0.1981
age	1	0.0129	0.0148	0.7648	0.3818
BMI	1	0.2222	0.0203	119.5594	<.0001
SBP	1	-0.00553	0.00735	0.5671	0.4514
D1_al	1	0.2859	0.1830	2.4404	0.1182
D2_al	1	0.3771	0.2111	3.1904	0.0741
D1_sm	1	0.2384	0.3017	0.6245	0.4294
D2_sm	1	-0.0951	0.3040	0.0979	0.7543

Odds Ratio Estimates			
Effect	Point Estimate	95% Wald Confidence Limits	
N	1.009	0.995	1.023
age	1.013	0.984	1.043
BMI	1.249	1.200	1.300
SBP	0.994	0.980	1.009
D1_al	1.331	0.930	1.905
D2_al	1.458	0.964	2.205
D1_sm	1.269	0.703	2.293
D2_sm	0.909	0.501	1.650

Association of Predicted Probabilities and Observed Responses			
Percent Concordant	72.3	Somers' D	0.452
Percent Discordant	27.1	Gamma	0.455
Percent Tied	0.6	Tau-a	0.128
Pairs	410310	c	0.726

Logistic Regression Results

The LOGISTIC Procedure

Model Information	
Data Set	WORK.SORTTEMPTABLESORTED
Response Variable	HDL_x
Number of Response Levels	2
Model	binary logit
Optimization Technique	Fisher's scoring

Number of Observations Read	1701
Number of Observations Used	1701

Response Profile		
Ordered Value	HDL_x	Total Frequency
1	0	1410
2	1	291

Probability modeled is HDL_x='1'.

Model Convergence Status
Convergence criterion (GCONV=1E-8) satisfied.

Model Fit Statistics		
Criterion	Intercept Only	Intercept and Covariates
AIC	1558.714	1412.834
SC	1564.153	1461.785
-2 Log L	1556.714	1394.834

Testing Global Null Hypothesis: BETA=0			
Test	Chi-Square	DF	Pr > ChiSq
Likelihood Ratio	161.8801	8	<.0001
Score	185.0185	8	<.0001
Wald	145.2842	8	<.0001

Logistic Regression Results

The LOGISTIC Procedure

Analysis of Maximum Likelihood Estimates					
Parameter	DF	Estimate	Standard Error	Wald Chi-Square	Pr > ChiSq
Intercept	1	-5.9970	0.9345	41.1853	<.0001
E	1	-0.00489	0.00711	0.4731	0.4916
age	1	0.00840	0.0146	0.3334	0.5637
BMI	1	0.2243	0.0203	122.4053	<.0001
SBP	1	-0.00572	0.00734	0.6068	0.4360
D1_al	1	0.2751	0.1830	2.2603	0.1327
D2_al	1	0.3838	0.2108	3.3155	0.0686
D1_sm	1	0.2403	0.3011	0.6368	0.4249
D2_sm	1	-0.0798	0.3049	0.0685	0.7935

Odds Ratio Estimates			
Effect	Point Estimate	95% Wald Confidence Limits	
E	0.995	0.981	1.009
age	1.008	0.980	1.038
BMI	1.251	1.203	1.302
SBP	0.994	0.980	1.009
D1_al	1.317	0.920	1.885
D2_al	1.468	0.971	2.219
D1_sm	1.272	0.705	2.294
D2_sm	0.923	0.508	1.678

Association of Predicted Probabilities and Observed Responses			
Percent Concordant	72.0	Somers' D	0.447
Percent Discordant	27.3	Gamma	0.450
Percent Tied	0.6	Tau-a	0.127
Pairs	410310	c	0.724

Logistic Regression Results

The LOGISTIC Procedure

Model Information	
Data Set	WORK.SORTTEMPTABLESORTED
Response Variable	HDL_x
Number of Response Levels	2
Model	binary logit
Optimization Technique	Fisher's scoring

Number of Observations Read	1701
Number of Observations Used	1701

Response Profile		
Ordered Value	HDL_x	Total Frequency
1	0	1410
2	1	291

Probability modeled is HDL_x='1'.

Model Convergence Status
Convergence criterion (GCONV=1E-8) satisfied.

Model Fit Statistics		
Criterion	Intercept Only	Intercept and Covariates
AIC	1558.714	1411.887
SC	1564.153	1460.838
-2 Log L	1556.714	1393.887

Testing Global Null Hypothesis: BETA=0			
Test	Chi-Square	DF	Pr > ChiSq
Likelihood Ratio	162.8275	8	<.0001
Score	185.8127	8	<.0001
Wald	146.1153	8	<.0001

Logistic Regression Results

The LOGISTIC Procedure

Analysis of Maximum Likelihood Estimates					
Parameter	DF	Estimate	Standard Error	Wald Chi-Square	Pr > ChiSq
Intercept	1	-5.6148	1.0004	31.5021	<.0001
O	1	-0.0103	0.00862	1.4209	0.2333
age	1	0.00822	0.0145	0.3207	0.5712
BMI	1	0.2240	0.0203	122.1226	<.0001
SBP	1	-0.00580	0.00735	0.6214	0.4305
D1_al	1	0.2668	0.1832	2.1202	0.1454
D2_al	1	0.3760	0.2110	3.1755	0.0748
D1_sm	1	0.2370	0.3010	0.6203	0.4309
D2_sm	1	-0.0718	0.3052	0.0554	0.8139

Odds Ratio Estimates			
Effect	Point Estimate	95% Wald Confidence Limits	
O	0.990	0.973	1.007
age	1.008	0.980	1.037
BMI	1.251	1.202	1.302
SBP	0.994	0.980	1.009
D1_al	1.306	0.912	1.870
D2_al	1.457	0.963	2.203
D1_sm	1.267	0.703	2.286
D2_sm	0.931	0.512	1.693

Association of Predicted Probabilities and Observed Responses			
Percent Concordant	72.3	Somers' D	0.453
Percent Discordant	27.1	Gamma	0.455
Percent Tied	0.6	Tau-a	0.128
Pairs	410310	c	0.726

Logistic Regression Results

The LOGISTIC Procedure

Model Information	
Data Set	WORK.SORTTEMPTABLESORTED
Response Variable	HDL_x
Number of Response Levels	2
Model	binary logit
Optimization Technique	Fisher's scoring

Number of Observations Read	1701
Number of Observations Used	1701

Response Profile		
Ordered Value	HDL_x	Total Frequency
1	0	1410
2	1	291

Probability modeled is HDL_x='1'.

Model Convergence Status
Convergence criterion (GCONV=1E-8) satisfied.

Model Fit Statistics		
Criterion	Intercept Only	Intercept and Covariates
AIC	1558.714	1410.964
SC	1564.153	1459.914
-2 Log L	1556.714	1392.964

Testing Global Null Hypothesis: BETA=0			
Test	Chi-Square	DF	Pr > ChiSq
Likelihood Ratio	163.7506	8	<.0001
Score	186.2465	8	<.0001
Wald	146.7284	8	<.0001

Logistic Regression Results

The LOGISTIC Procedure

Analysis of Maximum Likelihood Estimates					
Parameter	DF	Estimate	Standard Error	Wald Chi-Square	Pr > ChiSq
Intercept	1	-5.5947	0.9375	35.6112	<.0001
A	1	-0.0141	0.00919	2.3430	0.1258
age	1	0.0126	0.0147	0.7351	0.3912
BMI	1	0.2230	0.0203	120.9074	<.0001
SBP	1	-0.00517	0.00735	0.4947	0.4818
D1_al	1	0.3018	0.1837	2.7008	0.1003
D2_al	1	0.4042	0.2111	3.6665	0.0555
D1_sm	1	0.2231	0.3014	0.5478	0.4592
D2_sm	1	-0.1291	0.3069	0.1768	0.6741

Odds Ratio Estimates			
Effect	Point Estimate	95% Wald Confidence Limits	
A	0.986	0.968	1.004
age	1.013	0.984	1.042
BMI	1.250	1.201	1.300
SBP	0.995	0.981	1.009
D1_al	1.352	0.944	1.938
D2_al	1.498	0.991	2.266
D1_sm	1.250	0.692	2.256
D2_sm	0.879	0.482	1.604

Association of Predicted Probabilities and Observed Responses			
Percent Concordant	72.4	Somers' D	0.454
Percent Discordant	27.0	Gamma	0.457
Percent Tied	0.6	Tau-a	0.129
Pairs	410310	c	0.727

Logistic Regression Results

The LOGISTIC Procedure

Model Information	
Data Set	WORK.SORTTEMPTABLESORTED
Response Variable	HDL_x
Number of Response Levels	2
Model	binary logit
Optimization Technique	Fisher's scoring

Number of Observations Read	1701
Number of Observations Used	1701

Response Profile		
Ordered Value	HDL_x	Total Frequency
1	0	1410
2	1	291

Probability modeled is HDL_x='1'.

Model Convergence Status
Convergence criterion (GCONV=1E-8) satisfied.

Model Fit Statistics		
Criterion	Intercept Only	Intercept and Covariates
AIC	1558.714	1412.821
SC	1564.153	1461.771
-2 Log L	1556.714	1394.821

Testing Global Null Hypothesis: BETA=0			
Test	Chi-Square	DF	Pr > ChiSq
Likelihood Ratio	161.8937	8	<.0001
Score	184.9499	8	<.0001
Wald	145.4288	8	<.0001

Logistic Regression Results

The LOGISTIC Procedure

Analysis of Maximum Likelihood Estimates					
Parameter	DF	Estimate	Standard Error	Wald Chi-Square	Pr > ChiSq
Intercept	1	-5.9913	0.9358	40.9900	<.0001
C	1	-0.00572	0.00820	0.4867	0.4854
age	1	0.0108	0.0146	0.5456	0.4601
BMI	1	0.2229	0.0203	120.1861	<.0001
SBP	1	-0.00557	0.00735	0.5748	0.4484
D1_al	1	0.2818	0.1830	2.3698	0.1237
D2_al	1	0.3849	0.2111	3.3250	0.0682
D1_sm	1	0.2321	0.3012	0.5940	0.4409
D2_sm	1	-0.0935	0.3041	0.0946	0.7585

Odds Ratio Estimates			
Effect	Point Estimate	95% Wald Confidence Limits	
C	0.994	0.978	1.010
age	1.011	0.982	1.040
BMI	1.250	1.201	1.301
SBP	0.994	0.980	1.009
D1_al	1.325	0.926	1.897
D2_al	1.469	0.972	2.222
D1_sm	1.261	0.699	2.276
D2_sm	0.911	0.502	1.653

Association of Predicted Probabilities and Observed Responses			
Percent Concordant	72.2	Somers' D	0.451
Percent Discordant	27.1	Gamma	0.454
Percent Tied	0.6	Tau-a	0.128
Pairs	410310	c	0.725

Logistic Regression Results

The LOGISTIC Procedure

Model Information	
Data Set	WORK.SORTTEMPTABLESORTED
Response Variable	LDL_x
Number of Response Levels	2
Model	binary logit
Optimization Technique	Fisher's scoring

Number of Observations Read	1701
Number of Observations Used	1701

Response Profile		
Ordered Value	LDL_x	Total Frequency
1	0	1384
2	1	317

Probability modeled is LDL_x='1'.

Model Convergence Status
Convergence criterion (GCONV=1E-8) satisfied.

Model Fit Statistics		
Criterion	Intercept Only	Intercept and Covariates
AIC	1638.032	1543.545
SC	1643.471	1592.496
-2 Log L	1636.032	1525.545

Testing Global Null Hypothesis: BETA=0			
Test	Chi-Square	DF	Pr > ChiSq
Likelihood Ratio	110.4872	8	<.0001
Score	120.2870	8	<.0001
Wald	104.1387	8	<.0001

Logistic Regression Results

The LOGISTIC Procedure

Analysis of Maximum Likelihood Estimates					
Parameter	DF	Estimate	Standard Error	Wald Chi-Square	Pr > ChiSq
Intercept	1	-6.9048	0.8987	59.0256	<.0001
N	1	0.000634	0.00671	0.0090	0.9246
age	1	0.0843	0.0140	36.4537	<.0001
BMI	1	0.1342	0.0189	50.5495	<.0001
SBP	1	0.00223	0.00702	0.1005	0.7512
D1_al	1	0.0421	0.1818	0.0537	0.8167
D2_al	1	0.1630	0.2044	0.6360	0.4252
D1_sm	1	0.0950	0.2892	0.1080	0.7424
D2_sm	1	0.1807	0.2715	0.4433	0.5055

Odds Ratio Estimates			
Effect	Point Estimate	95% Wald Confidence Limits	
N	1.001	0.988	1.014
age	1.088	1.059	1.118
BMI	1.144	1.102	1.187
SBP	1.002	0.989	1.016
D1_al	1.043	0.730	1.490
D2_al	1.177	0.789	1.757
D1_sm	1.100	0.624	1.938
D2_sm	1.198	0.704	2.040

Association of Predicted Probabilities and Observed Responses			
Percent Concordant	67.0	Somers' D	0.347
Percent Discordant	32.3	Gamma	0.349
Percent Tied	0.7	Tau-a	0.105
Pairs	438728	c	0.673

Logistic Regression Results

The LOGISTIC Procedure

Model Information	
Data Set	WORK.SORTTEMPTABLESORTED
Response Variable	LDL_x
Number of Response Levels	2
Model	binary logit
Optimization Technique	Fisher's scoring

Number of Observations Read	1701
Number of Observations Used	1701

Response Profile		
Ordered Value	LDL_x	Total Frequency
1	0	1384
2	1	317

Probability modeled is LDL_x='1'.

Model Convergence Status
Convergence criterion (GCONV=1E-8) satisfied.

Model Fit Statistics		
Criterion	Intercept Only	Intercept and Covariates
AIC	1638.032	1542.805
SC	1643.471	1591.756
-2 Log L	1636.032	1524.805

Testing Global Null Hypothesis: BETA=0			
Test	Chi-Square	DF	Pr > ChiSq
Likelihood Ratio	111.2275	8	<.0001
Score	120.9637	8	<.0001
Wald	104.7751	8	<.0001

Logistic Regression Results

The LOGISTIC Procedure

Analysis of Maximum Likelihood Estimates					
Parameter	DF	Estimate	Standard Error	Wald Chi-Square	Pr > ChiSq
Intercept	1	-6.4866	0.8989	52.0751	<.0001
E	1	-0.00590	0.00681	0.7498	0.3865
age	1	0.0829	0.0138	36.1717	<.0001
BMI	1	0.1346	0.0188	51.1693	<.0001
SBP	1	0.00224	0.00701	0.1020	0.7494
D1_al	1	0.0372	0.1818	0.0419	0.8378
D2_al	1	0.1564	0.2043	0.5859	0.4440
D1_sm	1	0.0936	0.2896	0.1044	0.7466
D2_sm	1	0.1915	0.2715	0.4977	0.4805

Odds Ratio Estimates			
Effect	Point Estimate	95% Wald Confidence Limits	
E	0.994	0.981	1.007
age	1.086	1.057	1.116
BMI	1.144	1.103	1.187
SBP	1.002	0.989	1.016
D1_al	1.038	0.727	1.482
D2_al	1.169	0.783	1.745
D1_sm	1.098	0.622	1.937
D2_sm	1.211	0.711	2.062

Association of Predicted Probabilities and Observed Responses			
Percent Concordant	67.1	Somers' D	0.349
Percent Discordant	32.2	Gamma	0.351
Percent Tied	0.7	Tau-a	0.106
Pairs	438728	c	0.674

Logistic Regression Results

The LOGISTIC Procedure

Model Information	
Data Set	WORK.SORTTEMPTABLESORTED
Response Variable	LDL_x
Number of Response Levels	2
Model	binary logit
Optimization Technique	Fisher's scoring

Number of Observations Read	1701
Number of Observations Used	1701

Response Profile		
Ordered Value	LDL_x	Total Frequency
1	0	1384
2	1	317

Probability modeled is LDL_x='1'.

Model Convergence Status
Convergence criterion (GCONV=1E-8) satisfied.

Model Fit Statistics		
Criterion	Intercept Only	Intercept and Covariates
AIC	1638.032	1543.329
SC	1643.471	1592.280
-2 Log L	1636.032	1525.329

Testing Global Null Hypothesis: BETA=0			
Test	Chi-Square	DF	Pr > ChiSq
Likelihood Ratio	110.7030	8	<.0001
Score	120.4620	8	<.0001
Wald	104.2791	8	<.0001

Logistic Regression Results

The LOGISTIC Procedure

Analysis of Maximum Likelihood Estimates					
Parameter	DF	Estimate	Standard Error	Wald Chi-Square	Pr > ChiSq
Intercept	1	-6.5985	0.9662	46.6415	<.0001
O	1	-0.00389	0.00820	0.2249	0.6353
age	1	0.0835	0.0138	36.8867	<.0001
BMI	1	0.1343	0.0188	50.9059	<.0001
SBP	1	0.00219	0.00702	0.0973	0.7550
D1_al	1	0.0379	0.1818	0.0434	0.8350
D2_al	1	0.1596	0.2044	0.6097	0.4349
D1_sm	1	0.0936	0.2893	0.1047	0.7463
D2_sm	1	0.1902	0.2718	0.4900	0.4839

Odds Ratio Estimates			
Effect	Point Estimate	95% Wald Confidence Limits	
O	0.996	0.980	1.012
age	1.087	1.058	1.117
BMI	1.144	1.102	1.187
SBP	1.002	0.988	1.016
D1_al	1.039	0.727	1.483
D2_al	1.173	0.786	1.751
D1_sm	1.098	0.623	1.936
D2_sm	1.210	0.710	2.060

Association of Predicted Probabilities and Observed Responses			
Percent Concordant	67.1	Somers' D	0.349
Percent Discordant	32.2	Gamma	0.351
Percent Tied	0.7	Tau-a	0.106
Pairs	438728	c	0.674

Logistic Regression Results

The LOGISTIC Procedure

Model Information	
Data Set	WORK.SORTTEMPTABLESORTED
Response Variable	LDL_x
Number of Response Levels	2
Model	binary logit
Optimization Technique	Fisher's scoring

Number of Observations Read	1701
Number of Observations Used	1701

Response Profile		
Ordered Value	LDL_x	Total Frequency
1	0	1384
2	1	317

Probability modeled is LDL_x='1'.

Model Convergence Status
Convergence criterion (GCONV=1E-8) satisfied.

Model Fit Statistics		
Criterion	Intercept Only	Intercept and Covariates
AIC	1638.032	1542.844
SC	1643.471	1591.795
-2 Log L	1636.032	1524.844

Testing Global Null Hypothesis: BETA=0			
Test	Chi-Square	DF	Pr > ChiSq
Likelihood Ratio	111.1881	8	<.0001
Score	120.6987	8	<.0001
Wald	104.6024	8	<.0001

Logistic Regression Results

The LOGISTIC Procedure

Analysis of Maximum Likelihood Estimates					
Parameter	DF	Estimate	Standard Error	Wald Chi-Square	Pr > ChiSq
Intercept	1	-6.4878	0.9049	51.4080	<.0001
A	1	-0.00738	0.00876	0.7106	0.3992
age	1	0.0857	0.0139	38.1406	<.0001
BMI	1	0.1337	0.0188	50.4205	<.0001
SBP	1	0.00253	0.00703	0.1299	0.7185
D1_al	1	0.0530	0.1822	0.0846	0.7712
D2_al	1	0.1708	0.2043	0.6988	0.4032
D1_sm	1	0.0868	0.2893	0.0901	0.7640
D2_sm	1	0.1617	0.2726	0.3519	0.5530

Odds Ratio Estimates			
Effect	Point Estimate	95% Wald Confidence Limits	
A	0.993	0.976	1.010
age	1.089	1.060	1.119
BMI	1.143	1.102	1.186
SBP	1.003	0.989	1.016
D1_al	1.054	0.738	1.507
D2_al	1.186	0.795	1.770
D1_sm	1.091	0.619	1.923
D2_sm	1.176	0.689	2.006

Association of Predicted Probabilities and Observed Responses			
Percent Concordant	67.0	Somers' D	0.347
Percent Discordant	32.3	Gamma	0.350
Percent Tied	0.7	Tau-a	0.105
Pairs	438728	c	0.674

Logistic Regression Results

The LOGISTIC Procedure

Model Information	
Data Set	WORK.SORTTEMPTABLESORTED
Response Variable	LDL_x
Number of Response Levels	2
Model	binary logit
Optimization Technique	Fisher's scoring

Number of Observations Read	1701
Number of Observations Used	1701

Response Profile		
Ordered Value	LDL_x	Total Frequency
1	0	1384
2	1	317

Probability modeled is LDL_x='1'.

Model Convergence Status
Convergence criterion (GCONV=1E-8) satisfied.

Model Fit Statistics		
Criterion	Intercept Only	Intercept and Covariates
AIC	1638.032	1541.859
SC	1643.471	1590.809
-2 Log L	1636.032	1523.859

Testing Global Null Hypothesis: BETA=0			
Test	Chi-Square	DF	Pr > ChiSq
Likelihood Ratio	112.1737	8	<.0001
Score	121.5685	8	<.0001
Wald	105.5398	8	<.0001

Logistic Regression Results

The LOGISTIC Procedure

Analysis of Maximum Likelihood Estimates					
Parameter	DF	Estimate	Standard Error	Wald Chi-Square	Pr > ChiSq
Intercept	1	-6.2969	0.8987	49.0958	<.0001
C	1	-0.0102	0.00781	1.6951	0.1929
age	1	0.0865	0.0139	38.9415	<.0001
BMI	1	0.1325	0.0189	49.4106	<.0001
SBP	1	0.00247	0.00703	0.1232	0.7256
D1_al	1	0.0511	0.1818	0.0790	0.7786
D2_al	1	0.1591	0.2045	0.6055	0.4365
D1_sm	1	0.0815	0.2894	0.0793	0.7783
D2_sm	1	0.1723	0.2706	0.4056	0.5242

Odds Ratio Estimates			
Effect	Point Estimate	95% Wald Confidence Limits	
C	0.990	0.975	1.005
age	1.090	1.061	1.120
BMI	1.142	1.100	1.185
SBP	1.002	0.989	1.016
D1_al	1.052	0.737	1.503
D2_al	1.172	0.785	1.750
D1_sm	1.085	0.615	1.913
D2_sm	1.188	0.699	2.019

Association of Predicted Probabilities and Observed Responses			
Percent Concordant	67.1	Somers' D	0.349
Percent Discordant	32.2	Gamma	0.351
Percent Tied	0.7	Tau-a	0.106
Pairs	438728	c	0.674

Logistic Regression Results

The LOGISTIC Procedure

Model Information	
Data Set	WORK.SORTTEMPTABLESORTED
Response Variable	TG_x
Number of Response Levels	2
Model	binary logit
Optimization Technique	Fisher's scoring

Number of Observations Read	1701
Number of Observations Used	1701

Response Profile		
Ordered Value	TG_x	Total Frequency
1	0	1614
2	1	87

Probability modeled is TG_x='1'.

Model Convergence Status
Convergence criterion (GCONV=1E-8) satisfied.

Model Fit Statistics		
Criterion	Intercept Only	Intercept and Covariates
AIC	688.785	588.285
SC	694.224	637.236
-2 Log L	686.785	570.285

Testing Global Null Hypothesis: BETA=0			
Test	Chi-Square	DF	Pr > ChiSq
Likelihood Ratio	116.5003	8	<.0001
Score	167.4281	8	<.0001
Wald	113.8155	8	<.0001

Logistic Regression Results

The LOGISTIC Procedure

Analysis of Maximum Likelihood Estimates					
Parameter	DF	Estimate	Standard Error	Wald Chi-Square	Pr > ChiSq
Intercept	1	-10.8947	1.5383	50.1574	<.0001
N	1	0.0119	0.0121	0.9627	0.3265
age	1	0.0462	0.0241	3.6554	0.0559
BMI	1	0.2482	0.0289	73.7452	<.0001
SBP	1	0.00215	0.0118	0.0334	0.8550
D1_al	1	0.1197	0.3236	0.1367	0.7116
D2_al	1	0.4122	0.3416	1.4566	0.2275
D1_sm	1	-0.2182	0.6197	0.1240	0.7247
D2_sm	1	0.3313	0.4062	0.6650	0.4148

Odds Ratio Estimates			
Effect	Point Estimate	95% Wald Confidence Limits	
N	1.012	0.988	1.036
age	1.047	0.999	1.098
BMI	1.282	1.211	1.356
SBP	1.002	0.979	1.026
D1_al	1.127	0.598	2.125
D2_al	1.510	0.773	2.950
D1_sm	0.804	0.239	2.708
D2_sm	1.393	0.628	3.088

Association of Predicted Probabilities and Observed Responses			
Percent Concordant	77.3	Somers' D	0.561
Percent Discordant	21.2	Gamma	0.570
Percent Tied	1.5	Tau-a	0.054
Pairs	140418	c	0.780

Logistic Regression Results

The LOGISTIC Procedure

Model Information	
Data Set	WORK.SORTTEMPTABLESORTED
Response Variable	TG_x
Number of Response Levels	2
Model	binary logit
Optimization Technique	Fisher's scoring

Number of Observations Read	1701
Number of Observations Used	1701

Response Profile		
Ordered Value	TG_x	Total Frequency
1	0	1614
2	1	87

Probability modeled is TG_x='1'.

Model Convergence Status
Convergence criterion (GCONV=1E-8) satisfied.

Model Fit Statistics		
Criterion	Intercept Only	Intercept and Covariates
AIC	688.785	588.188
SC	694.224	637.139
-2 Log L	686.785	570.188

Testing Global Null Hypothesis: BETA=0			
Test	Chi-Square	DF	Pr > ChiSq
Likelihood Ratio	116.5975	8	<.0001
Score	167.9093	8	<.0001
Wald	113.4083	8	<.0001

Logistic Regression Results

The LOGISTIC Procedure

Analysis of Maximum Likelihood Estimates					
Parameter	DF	Estimate	Standard Error	Wald Chi-Square	Pr > ChiSq
Intercept	1	-9.2888	1.4771	39.5451	<.0001
E	1	-0.0125	0.0121	1.0631	0.3025
age	1	0.0387	0.0234	2.7383	0.0980
BMI	1	0.2514	0.0289	75.5422	<.0001
SBP	1	0.00175	0.0117	0.0225	0.8807
D1_al	1	0.0949	0.3243	0.0857	0.7697
D2_al	1	0.4184	0.3407	1.5078	0.2195
D1_sm	1	-0.2227	0.6206	0.1287	0.7197
D2_sm	1	0.3509	0.4088	0.7368	0.3907

Odds Ratio Estimates			
Effect	Point Estimate	95% Wald Confidence Limits	
E	0.988	0.964	1.011
age	1.039	0.993	1.088
BMI	1.286	1.215	1.361
SBP	1.002	0.979	1.025
D1_al	1.100	0.582	2.076
D2_al	1.519	0.779	2.963
D1_sm	0.800	0.237	2.701
D2_sm	1.420	0.637	3.165

Association of Predicted Probabilities and Observed Responses			
Percent Concordant	77.6	Somers' D	0.567
Percent Discordant	20.9	Gamma	0.575
Percent Tied	1.5	Tau-a	0.055
Pairs	140418	c	0.783

Logistic Regression Results

The LOGISTIC Procedure

Model Information	
Data Set	WORK.SORTTEMPTABLESORTED
Response Variable	TG_x
Number of Response Levels	2
Model	binary logit
Optimization Technique	Fisher's scoring

Number of Observations Read	1701
Number of Observations Used	1701

Response Profile		
Ordered Value	TG_x	Total Frequency
1	0	1614
2	1	87

Probability modeled is TG_x='1'.

Model Convergence Status
Convergence criterion (GCONV=1E-8) satisfied.

Model Fit Statistics		
Criterion	Intercept Only	Intercept and Covariates
AIC	688.785	586.737
SC	694.224	635.688
-2 Log L	686.785	568.737

Testing Global Null Hypothesis: BETA=0			
Test	Chi-Square	DF	Pr > ChiSq
Likelihood Ratio	118.0486	8	<.0001
Score	168.8503	8	<.0001
Wald	114.1103	8	<.0001

Logistic Regression Results

The LOGISTIC Procedure

Analysis of Maximum Likelihood Estimates					
Parameter	DF	Estimate	Standard Error	Wald Chi-Square	Pr > ChiSq
Intercept	1	-8.5787	1.5750	29.6688	<.0001
O	1	-0.0231	0.0145	2.5203	0.1124
age	1	0.0385	0.0233	2.7148	0.0994
BMI	1	0.2513	0.0290	75.1390	<.0001
SBP	1	0.00185	0.0118	0.0247	0.8752
D1_al	1	0.0785	0.3242	0.0587	0.8086
D2_al	1	0.4021	0.3406	1.3937	0.2378
D1_sm	1	-0.2224	0.6199	0.1287	0.7198
D2_sm	1	0.3657	0.4096	0.7971	0.3720

Odds Ratio Estimates			
Effect	Point Estimate	95% Wald Confidence Limits	
O	0.977	0.950	1.005
age	1.039	0.993	1.088
BMI	1.286	1.215	1.361
SBP	1.002	0.979	1.025
D1_al	1.082	0.573	2.042
D2_al	1.495	0.767	2.914
D1_sm	0.801	0.238	2.698
D2_sm	1.442	0.646	3.217

Association of Predicted Probabilities and Observed Responses			
Percent Concordant	78.0	Somers' D	0.574
Percent Discordant	20.6	Gamma	0.583
Percent Tied	1.4	Tau-a	0.056
Pairs	140418	c	0.787

Logistic Regression Results

The LOGISTIC Procedure

Model Information	
Data Set	WORK.SORTTEMPTABLESORTED
Response Variable	TG_x
Number of Response Levels	2
Model	binary logit
Optimization Technique	Fisher's scoring

Number of Observations Read	1701
Number of Observations Used	1701

Response Profile		
Ordered Value	TG_x	Total Frequency
1	0	1614
2	1	87

Probability modeled is TG_x='1'.

Model Convergence Status
Convergence criterion (GCONV=1E-8) satisfied.

Model Fit Statistics		
Criterion	Intercept Only	Intercept and Covariates
AIC	688.785	589.192
SC	694.224	638.143
-2 Log L	686.785	571.192

Testing Global Null Hypothesis: BETA=0			
Test	Chi-Square	DF	Pr > ChiSq
Likelihood Ratio	115.5936	8	<.0001
Score	166.9775	8	<.0001
Wald	113.0071	8	<.0001

Logistic Regression Results

The LOGISTIC Procedure

Analysis of Maximum Likelihood Estimates					
Parameter	DF	Estimate	Standard Error	Wald Chi-Square	Pr > ChiSq
Intercept	1	-9.8914	1.4952	43.7632	<.0001
A	1	-0.00370	0.0157	0.0558	0.8133
age	1	0.0413	0.0238	3.0165	0.0824
BMI	1	0.2505	0.0289	75.1414	<.0001
SBP	1	0.00198	0.0117	0.0284	0.8662
D1_al	1	0.1098	0.3248	0.1143	0.7354
D2_al	1	0.4303	0.3415	1.5872	0.2077
D1_sm	1	-0.2071	0.6185	0.1121	0.7378
D2_sm	1	0.3222	0.4113	0.6138	0.4334

Odds Ratio Estimates			
Effect	Point Estimate	95% Wald Confidence Limits	
A	0.996	0.966	1.027
age	1.042	0.995	1.092
BMI	1.285	1.214	1.359
SBP	1.002	0.979	1.025
D1_al	1.116	0.591	2.109
D2_al	1.538	0.787	3.003
D1_sm	0.813	0.242	2.732
D2_sm	1.380	0.616	3.091

Association of Predicted Probabilities and Observed Responses			
Percent Concordant	77.3	Somers' D	0.561
Percent Discordant	21.2	Gamma	0.570
Percent Tied	1.5	Tau-a	0.054
Pairs	140418	c	0.781

Logistic Regression Results

The LOGISTIC Procedure

Model Information	
Data Set	WORK.SORTTEMPTABLESORTED
Response Variable	TG_x
Number of Response Levels	2
Model	binary logit
Optimization Technique	Fisher's scoring

Number of Observations Read	1701
Number of Observations Used	1701

Response Profile		
Ordered Value	TG_x	Total Frequency
1	0	1614
2	1	87

Probability modeled is TG_x='1'.

Model Convergence Status
Convergence criterion (GCONV=1E-8) satisfied.

Model Fit Statistics		
Criterion	Intercept Only	Intercept and Covariates
AIC	688.785	588.500
SC	694.224	637.450
-2 Log L	686.785	570.500

Testing Global Null Hypothesis: BETA=0			
Test	Chi-Square	DF	Pr > ChiSq
Likelihood Ratio	116.2858	8	<.0001
Score	167.2417	8	<.0001
Wald	113.7099	8	<.0001

Logistic Regression Results

The LOGISTIC Procedure

Analysis of Maximum Likelihood Estimates					
Parameter	DF	Estimate	Standard Error	Wald Chi-Square	Pr > ChiSq
Intercept	1	-9.4180	1.4798	40.5055	<.0001
C	1	-0.0122	0.0141	0.7511	0.3861
age	1	0.0439	0.0238	3.4160	0.0646
BMI	1	0.2486	0.0290	73.6725	<.0001
SBP	1	0.00217	0.0118	0.0339	0.8539
D1_al	1	0.1085	0.3240	0.1121	0.7377
D2_al	1	0.4094	0.3416	1.4361	0.2308
D1_sm	1	-0.2243	0.6191	0.1313	0.7171
D2_sm	1	0.3328	0.4054	0.6738	0.4117

Odds Ratio Estimates			
Effect	Point Estimate	95% Wald Confidence Limits	
C	0.988	0.961	1.015
age	1.045	0.997	1.095
BMI	1.282	1.211	1.357
SBP	1.002	0.979	1.026
D1_al	1.115	0.591	2.103
D2_al	1.506	0.771	2.941
D1_sm	0.799	0.237	2.689
D2_sm	1.395	0.630	3.088

Association of Predicted Probabilities and Observed Responses			
Percent Concordant	77.7	Somers' D	0.568
Percent Discordant	20.9	Gamma	0.576
Percent Tied	1.4	Tau-a	0.055
Pairs	140418	c	0.784

Logistic Regression Results

The LOGISTIC Procedure

Model Information	
Data Set	WORK.SORTTEMPTABLESORTED
Response Variable	TC_x
Number of Response Levels	2
Model	binary logit
Optimization Technique	Fisher's scoring

Number of Observations Read	1701
Number of Observations Used	1701

Response Profile		
Ordered Value	TC_x	Total Frequency
1	0	1386
2	1	315

Probability modeled is TC_x='1'.

Model Convergence Status
Convergence criterion (GCONV=1E-8) satisfied.

Model Fit Statistics		
Criterion	Intercept Only	Intercept and Covariates
AIC	1632.121	1582.548
SC	1637.560	1631.499
-2 Log L	1630.121	1564.548

Testing Global Null Hypothesis: BETA=0			
Test	Chi-Square	DF	Pr > ChiSq
Likelihood Ratio	65.5734	8	<.0001
Score	70.1870	8	<.0001
Wald	64.7726	8	<.0001

Logistic Regression Results

The LOGISTIC Procedure

Analysis of Maximum Likelihood Estimates					
Parameter	DF	Estimate	Standard Error	Wald Chi-Square	Pr > ChiSq
Intercept	1	-4.3689	0.8488	26.4916	<.0001
N1	1	-0.0271	0.0296	0.8407	0.3592
age	1	0.0592	0.0137	18.7680	<.0001
BMI	1	0.1140	0.0187	37.3461	<.0001
SBP	1	-0.00828	0.00701	1.3953	0.2375
D1_al	1	0.1666	0.1742	0.9148	0.3389
D2_al	1	0.3091	0.1979	2.4384	0.1184
D1_sm	1	0.1700	0.2805	0.3672	0.5445
D2_sm	1	-0.1018	0.2861	0.1267	0.7219

Odds Ratio Estimates			
Effect	Point Estimate	95% Wald Confidence Limits	
N1	0.973	0.918	1.031
age	1.061	1.033	1.090
BMI	1.121	1.081	1.163
SBP	0.992	0.978	1.005
D1_al	1.181	0.840	1.662
D2_al	1.362	0.924	2.008
D1_sm	1.185	0.684	2.054
D2_sm	0.903	0.516	1.582

Association of Predicted Probabilities and Observed Responses			
Percent Concordant	62.1	Somers' D	0.252
Percent Discordant	37.0	Gamma	0.254
Percent Tied	0.9	Tau-a	0.076
Pairs	436590	c	0.626

Logistic Regression Results

The LOGISTIC Procedure

Model Information	
Data Set	WORK.SORTTEMPTABLESORTED
Response Variable	HDL_x
Number of Response Levels	2
Model	binary logit
Optimization Technique	Fisher's scoring

Number of Observations Read	1701
Number of Observations Used	1701

Response Profile		
Ordered Value	HDL_x	Total Frequency
1	0	1410
2	1	291

Probability modeled is HDL_x='1'.

Model Convergence Status
Convergence criterion (GCONV=1E-8) satisfied.

Model Fit Statistics		
Criterion	Intercept Only	Intercept and Covariates
AIC	1558.714	1413.150
SC	1564.153	1462.101
-2 Log L	1556.714	1395.150

Testing Global Null Hypothesis: BETA=0			
Test	Chi-Square	DF	Pr > ChiSq
Likelihood Ratio	161.5640	8	<.0001
Score	184.7386	8	<.0001
Wald	145.0254	8	<.0001

Logistic Regression Results

The LOGISTIC Procedure

Analysis of Maximum Likelihood Estimates					
Parameter	DF	Estimate	Standard Error	Wald Chi-Square	Pr > ChiSq
Intercept	1	-6.4696	0.9025	51.3875	<.0001
N1	1	0.0126	0.0319	0.1565	0.6924
age	1	0.0100	0.0146	0.4719	0.4921
BMI	1	0.2241	0.0203	122.2044	<.0001
SBP	1	-0.00572	0.00734	0.6067	0.4360
D1_al	1	0.2793	0.1829	2.3327	0.1267
D2_al	1	0.3865	0.2109	3.3584	0.0669
D1_sm	1	0.2374	0.3012	0.6211	0.4306
D2_sm	1	-0.0861	0.3044	0.0801	0.7772

Odds Ratio Estimates			
Effect	Point Estimate	95% Wald Confidence Limits	
N1	1.013	0.951	1.078
age	1.010	0.982	1.039
BMI	1.251	1.202	1.302
SBP	0.994	0.980	1.009
D1_al	1.322	0.924	1.892
D2_al	1.472	0.973	2.225
D1_sm	1.268	0.703	2.288
D2_sm	0.917	0.505	1.666

Association of Predicted Probabilities and Observed Responses			
Percent Concordant	72.2	Somers' D	0.449
Percent Discordant	27.2	Gamma	0.452
Percent Tied	0.6	Tau-a	0.128
Pairs	410310	c	0.725

Logistic Regression Results

The LOGISTIC Procedure

Model Information	
Data Set	WORK.SORTTEMPTABLESORTED
Response Variable	LDL_x
Number of Response Levels	2
Model	binary logit
Optimization Technique	Fisher's scoring

Number of Observations Read	1701
Number of Observations Used	1701

Response Profile		
Ordered Value	LDL_x	Total Frequency
1	0	1384
2	1	317

Probability modeled is LDL_x='1'.

Model Convergence Status
Convergence criterion (GCONV=1E-8) satisfied.

Model Fit Statistics		
Criterion	Intercept Only	Intercept and Covariates
AIC	1638.032	1543.539
SC	1643.471	1592.490
-2 Log L	1636.032	1525.539

Testing Global Null Hypothesis: BETA=0			
Test	Chi-Square	DF	Pr > ChiSq
Likelihood Ratio	110.4935	8	<.0001
Score	120.3078	8	<.0001
Wald	104.1480	8	<.0001

Logistic Regression Results

The LOGISTIC Procedure

Analysis of Maximum Likelihood Estimates					
Parameter	DF	Estimate	Standard Error	Wald Chi-Square	Pr > ChiSq
Intercept	1	-6.9101	0.8729	62.6618	<.0001
N1	1	0.00372	0.0301	0.0152	0.9017
age	1	0.0843	0.0138	37.0204	<.0001
BMI	1	0.1343	0.0188	50.9402	<.0001
SBP	1	0.00221	0.00702	0.0991	0.7529
D1_al	1	0.0419	0.1817	0.0532	0.8176
D2_al	1	0.1630	0.2043	0.6367	0.4249
D1_sm	1	0.0942	0.2893	0.1060	0.7448
D2_sm	1	0.1815	0.2713	0.4477	0.5034

Odds Ratio Estimates			
Effect	Point Estimate	95% Wald Confidence Limits	
N1	1.004	0.946	1.065
age	1.088	1.059	1.118
BMI	1.144	1.102	1.187
SBP	1.002	0.989	1.016
D1_al	1.043	0.730	1.489
D2_al	1.177	0.789	1.757
D1_sm	1.099	0.623	1.937
D2_sm	1.199	0.705	2.041

Association of Predicted Probabilities and Observed Responses			
Percent Concordant	67.0	Somers' D	0.347
Percent Discordant	32.3	Gamma	0.349
Percent Tied	0.7	Tau-a	0.105
Pairs	438728	c	0.673

Logistic Regression Results

The LOGISTIC Procedure

Model Information	
Data Set	WORK.SORTTEMPTABLESORTED
Response Variable	TG_x
Number of Response Levels	2
Model	binary logit
Optimization Technique	Fisher's scoring

Number of Observations Read	1701
Number of Observations Used	1701

Response Profile		
Ordered Value	TG_x	Total Frequency
1	0	1614
2	1	87

Probability modeled is TG_x='1'.

Model Convergence Status
Convergence criterion (GCONV=1E-8) satisfied.

Model Fit Statistics		
Criterion	Intercept Only	Intercept and Covariates
AIC	688.785	589.132
SC	694.224	638.083
-2 Log L	686.785	571.132

Testing Global Null Hypothesis: BETA=0			
Test	Chi-Square	DF	Pr > ChiSq
Likelihood Ratio	115.6532	8	<.0001
Score	167.0798	8	<.0001
Wald	113.1470	8	<.0001

Logistic Regression Results

The LOGISTIC Procedure

Analysis of Maximum Likelihood Estimates					
Parameter	DF	Estimate	Standard Error	Wald Chi-Square	Pr > ChiSq
Intercept	1	-10.3105	1.4566	50.1054	<.0001
N1	1	0.0187	0.0552	0.1150	0.7345
age	1	0.0415	0.0237	3.0784	0.0793
BMI	1	0.2504	0.0289	75.2321	<.0001
SBP	1	0.00186	0.0117	0.0251	0.8742
D1_al	1	0.1063	0.3238	0.1078	0.7427
D2_al	1	0.4235	0.3414	1.5389	0.2148
D1_sm	1	-0.2132	0.6198	0.1183	0.7308
D2_sm	1	0.3408	0.4072	0.7003	0.4027

Odds Ratio Estimates			
Effect	Point Estimate	95% Wald Confidence Limits	
N1	1.019	0.914	1.135
age	1.042	0.995	1.092
BMI	1.285	1.214	1.359
SBP	1.002	0.979	1.025
D1_al	1.112	0.590	2.098
D2_al	1.527	0.782	2.982
D1_sm	0.808	0.240	2.723
D2_sm	1.406	0.633	3.123

Association of Predicted Probabilities and Observed Responses			
Percent Concordant	77.3	Somers' D	0.562
Percent Discordant	21.1	Gamma	0.571
Percent Tied	1.5	Tau-a	0.055
Pairs	140418	c	0.781

Logistic Regression Results

The LOGISTIC Procedure

Model Information	
Data Set	WORK.SORTTEMPTABLESORTED
Response Variable	TC_x
Number of Response Levels	2
Model	binary logit
Optimization Technique	Fisher's scoring

Number of Observations Read	1701
Number of Observations Used	1701

Response Profile		
Ordered Value	TC_x	Total Frequency
1	0	1386
2	1	315

Probability modeled is TC_x='1'.

Model Convergence Status
Convergence criterion (GCONV=1E-8) satisfied.

Model Fit Statistics		
Criterion	Intercept Only	Intercept and Covariates
AIC	1632.121	1583.125
SC	1637.560	1632.076
-2 Log L	1630.121	1565.125

Testing Global Null Hypothesis: BETA=0			
Test	Chi-Square	DF	Pr > ChiSq
Likelihood Ratio	64.9966	8	<.0001
Score	69.7582	8	<.0001
Wald	64.3774	8	<.0001

Logistic Regression Results

The LOGISTIC Procedure

Analysis of Maximum Likelihood Estimates					
Parameter	DF	Estimate	Standard Error	Wald Chi-Square	Pr > ChiSq
Intercept	1	-4.5814	0.8029	32.5618	<.0001
N2	1	-0.0147	0.0288	0.2616	0.6090
age	1	0.0605	0.0136	19.9452	<.0001
BMI	1	0.1148	0.0187	37.6203	<.0001
SBP	1	-0.00846	0.00701	1.4577	0.2273
D1_al	1	0.1659	0.1742	0.9062	0.3411
D2_al	1	0.3063	0.1980	2.3946	0.1218
D1_sm	1	0.1635	0.2807	0.3396	0.5601
D2_sm	1	-0.0931	0.2862	0.1057	0.7451

Odds Ratio Estimates			
Effect	Point Estimate	95% Wald Confidence Limits	
N2	0.985	0.931	1.043
age	1.062	1.035	1.091
BMI	1.122	1.081	1.164
SBP	0.992	0.978	1.005
D1_al	1.180	0.839	1.661
D2_al	1.358	0.922	2.002
D1_sm	1.178	0.679	2.041
D2_sm	0.911	0.520	1.597

Association of Predicted Probabilities and Observed Responses			
Percent Concordant	62.1	Somers' D	0.251
Percent Discordant	37.0	Gamma	0.253
Percent Tied	0.9	Tau-a	0.076
Pairs	436590	c	0.626

Logistic Regression Results

The LOGISTIC Procedure

Model Information	
Data Set	WORK.SORTTEMPTABLESORTED
Response Variable	HDL_x
Number of Response Levels	2
Model	binary logit
Optimization Technique	Fisher's scoring

Number of Observations Read	1701
Number of Observations Used	1701

Response Profile		
Ordered Value	HDL_x	Total Frequency
1	0	1410
2	1	291

Probability modeled is HDL_x='1'.

Model Convergence Status
Convergence criterion (GCONV=1E-8) satisfied.

Model Fit Statistics		
Criterion	Intercept Only	Intercept and Covariates
AIC	1558.714	1411.470
SC	1564.153	1460.421
-2 Log L	1556.714	1393.470

Testing Global Null Hypothesis: BETA=0			
Test	Chi-Square	DF	Pr > ChiSq
Likelihood Ratio	163.2445	8	<.0001
Score	186.0969	8	<.0001
Wald	146.0924	8	<.0001

Logistic Regression Results

The LOGISTIC Procedure

Analysis of Maximum Likelihood Estimates					
Parameter	DF	Estimate	Standard Error	Wald Chi-Square	Pr > ChiSq
Intercept	1	-6.6749	0.8604	60.1795	<.0001
N2	1	0.0413	0.0305	1.8406	0.1749
age	1	0.0101	0.0145	0.4880	0.4848
BMI	1	0.2220	0.0203	119.2934	<.0001
SBP	1	-0.00520	0.00736	0.4986	0.4801
D1_al	1	0.2874	0.1831	2.4647	0.1164
D2_al	1	0.3774	0.2112	3.1934	0.0739
D1_sm	1	0.2375	0.3013	0.6211	0.4306
D2_sm	1	-0.1135	0.3054	0.1380	0.7103

Odds Ratio Estimates			
Effect	Point Estimate	95% Wald Confidence Limits	
N2	1.042	0.982	1.106
age	1.010	0.982	1.039
BMI	1.249	1.200	1.299
SBP	0.995	0.981	1.009
D1_al	1.333	0.931	1.908
D2_al	1.458	0.964	2.206
D1_sm	1.268	0.703	2.289
D2_sm	0.893	0.491	1.624

Association of Predicted Probabilities and Observed Responses			
Percent Concordant	72.5	Somers' D	0.456
Percent Discordant	26.9	Gamma	0.459
Percent Tied	0.6	Tau-a	0.130
Pairs	410310	c	0.728

Logistic Regression Results

The LOGISTIC Procedure

Model Information	
Data Set	WORK.SORTTEMPTABLESORTED
Response Variable	LDL_x
Number of Response Levels	2
Model	binary logit
Optimization Technique	Fisher's scoring

Number of Observations Read	1701
Number of Observations Used	1701

Response Profile		
Ordered Value	LDL_x	Total Frequency
1	0	1384
2	1	317

Probability modeled is LDL_x='1'.

Model Convergence Status
Convergence criterion (GCONV=1E-8) satisfied.

Model Fit Statistics		
Criterion	Intercept Only	Intercept and Covariates
AIC	1638.032	1543.543
SC	1643.471	1592.494
-2 Log L	1636.032	1525.543

Testing Global Null Hypothesis: BETA=0			
Test	Chi-Square	DF	Pr > ChiSq
Likelihood Ratio	110.4889	8	<.0001
Score	120.3383	8	<.0001
Wald	104.1142	8	<.0001

Logistic Regression Results

The LOGISTIC Procedure

Analysis of Maximum Likelihood Estimates					
Parameter	DF	Estimate	Standard Error	Wald Chi-Square	Pr > ChiSq
Intercept	1	-6.8391	0.8249	68.7385	<.0001
N2	1	-0.00301	0.0291	0.0107	0.9176
age	1	0.0840	0.0137	37.3979	<.0001
BMI	1	0.1345	0.0189	50.6724	<.0001
SBP	1	0.00218	0.00702	0.0968	0.7557
D1_al	1	0.0409	0.1818	0.0507	0.8219
D2_al	1	0.1649	0.2043	0.6514	0.4196
D1_sm	1	0.0952	0.2892	0.1084	0.7420
D2_sm	1	0.1836	0.2720	0.4557	0.4996

Odds Ratio Estimates			
Effect	Point Estimate	95% Wald Confidence Limits	
N2	0.997	0.942	1.056
age	1.088	1.059	1.117
BMI	1.144	1.102	1.187
SBP	1.002	0.988	1.016
D1_al	1.042	0.729	1.488
D2_al	1.179	0.790	1.760
D1_sm	1.100	0.624	1.939
D2_sm	1.202	0.705	2.048

Association of Predicted Probabilities and Observed Responses			
Percent Concordant	67.0	Somers' D	0.347
Percent Discordant	32.3	Gamma	0.350
Percent Tied	0.7	Tau-a	0.105
Pairs	438728	c	0.674

Logistic Regression Results

The LOGISTIC Procedure

Model Information	
Data Set	WORK.SORTTEMPTABLESORTED
Response Variable	TG_x
Number of Response Levels	2
Model	binary logit
Optimization Technique	Fisher's scoring

Number of Observations Read	1701
Number of Observations Used	1701

Response Profile		
Ordered Value	TG_x	Total Frequency
1	0	1614
2	1	87

Probability modeled is TG_x='1'.

Model Convergence Status
Convergence criterion (GCONV=1E-8) satisfied.

Model Fit Statistics		
Criterion	Intercept Only	Intercept and Covariates
AIC	688.785	589.150
SC	694.224	638.101
-2 Log L	686.785	571.150

Testing Global Null Hypothesis: BETA=0			
Test	Chi-Square	DF	Pr > ChiSq
Likelihood Ratio	115.6350	8	<.0001
Score	166.9695	8	<.0001
Wald	113.0606	8	<.0001

Logistic Regression Results

The LOGISTIC Procedure

Analysis of Maximum Likelihood Estimates					
Parameter	DF	Estimate	Standard Error	Wald Chi-Square	Pr > ChiSq
Intercept	1	-10.2290	1.3730	55.5075	<.0001
N2	1	0.0161	0.0518	0.0973	0.7551
age	1	0.0409	0.0235	3.0375	0.0814
BMI	1	0.2496	0.0290	73.9521	<.0001
SBP	1	0.00213	0.0118	0.0327	0.8565
D1_al	1	0.1079	0.3239	0.1109	0.7391
D2_al	1	0.4210	0.3417	1.5187	0.2178
D1_sm	1	-0.2052	0.6184	0.1101	0.7400
D2_sm	1	0.3247	0.4087	0.6310	0.4270

Odds Ratio Estimates			
Effect	Point Estimate	95% Wald Confidence Limits	
N2	1.016	0.918	1.125
age	1.042	0.995	1.091
BMI	1.284	1.213	1.359
SBP	1.002	0.979	1.026
D1_al	1.114	0.590	2.102
D2_al	1.524	0.780	2.976
D1_sm	0.814	0.242	2.737
D2_sm	1.384	0.621	3.083

Association of Predicted Probabilities and Observed Responses			
Percent Concordant	77.3	Somers' D	0.561
Percent Discordant	21.2	Gamma	0.570
Percent Tied	1.6	Tau-a	0.054
Pairs	140418	c	0.780

Logistic Regression Results

The LOGISTIC Procedure

Model Information	
Data Set	WORK.SORTTEMPTABLESORTED
Response Variable	TC_x
Number of Response Levels	2
Model	binary logit
Optimization Technique	Fisher's scoring

Number of Observations Read	1701
Number of Observations Used	1701

Response Profile		
Ordered Value	TC_x	Total Frequency
1	0	1386
2	1	315

Probability modeled is TC_x='1'.

Model Convergence Status
Convergence criterion (GCONV=1E-8) satisfied.

Model Fit Statistics		
Criterion	Intercept Only	Intercept and Covariates
AIC	1632.121	1583.034
SC	1637.560	1631.985
-2 Log L	1630.121	1565.034

Testing Global Null Hypothesis: BETA=0			
Test	Chi-Square	DF	Pr > ChiSq
Likelihood Ratio	65.0872	8	<.0001
Score	69.7172	8	<.0001
Wald	64.5106	8	<.0001

Logistic Regression Results

The LOGISTIC Procedure

Analysis of Maximum Likelihood Estimates					
Parameter	DF	Estimate	Standard Error	Wald Chi-Square	Pr > ChiSq
Intercept	1	-4.8535	0.8119	35.7330	<.0001
N3	1	0.0142	0.0238	0.3525	0.5527
age	1	0.0620	0.0137	20.4321	<.0001
BMI	1	0.1132	0.0187	36.8205	<.0001
SBP	1	-0.00829	0.00701	1.3981	0.2370
D1_al	1	0.1728	0.1742	0.9844	0.3211
D2_al	1	0.2970	0.1979	2.2512	0.1335
D1_sm	1	0.1640	0.2809	0.3411	0.5592
D2_sm	1	-0.1045	0.2855	0.1340	0.7143

Odds Ratio Estimates			
Effect	Point Estimate	95% Wald Confidence Limits	
N3	1.014	0.968	1.063
age	1.064	1.036	1.093
BMI	1.120	1.080	1.162
SBP	0.992	0.978	1.005
D1_al	1.189	0.845	1.672
D2_al	1.346	0.913	1.984
D1_sm	1.178	0.679	2.043
D2_sm	0.901	0.515	1.576

Association of Predicted Probabilities and Observed Responses			
Percent Concordant	62.0	Somers' D	0.249
Percent Discordant	37.1	Gamma	0.252
Percent Tied	0.9	Tau-a	0.075
Pairs	436590	c	0.625

Logistic Regression Results

The LOGISTIC Procedure

Model Information	
Data Set	WORK.SORTTEMPTABLESORTED
Response Variable	HDL_x
Number of Response Levels	2
Model	binary logit
Optimization Technique	Fisher's scoring

Number of Observations Read	1701
Number of Observations Used	1701

Response Profile		
Ordered Value	HDL_x	Total Frequency
1	0	1410
2	1	291

Probability modeled is HDL_x='1'.

Model Convergence Status
Convergence criterion (GCONV=1E-8) satisfied.

Model Fit Statistics		
Criterion	Intercept Only	Intercept and Covariates
AIC	1558.714	1412.604
SC	1564.153	1461.554
-2 Log L	1556.714	1394.604

Testing Global Null Hypothesis: BETA=0			
Test	Chi-Square	DF	Pr > ChiSq
Likelihood Ratio	162.1108	8	<.0001
Score	185.1455	8	<.0001
Wald	145.5512	8	<.0001

Logistic Regression Results

The LOGISTIC Procedure

Analysis of Maximum Likelihood Estimates					
Parameter	DF	Estimate	Standard Error	Wald Chi-Square	Pr > ChiSq
Intercept	1	-6.5429	0.8587	58.0636	<.0001
N3	1	0.0214	0.0255	0.7031	0.4018
age	1	0.0112	0.0147	0.5851	0.4443
BMI	1	0.2229	0.0203	120.2768	<.0001
SBP	1	-0.00564	0.00735	0.5895	0.4426
D1_al	1	0.2836	0.1830	2.4018	0.1212
D2_al	1	0.3834	0.2109	3.3054	0.0691
D1_sm	1	0.2418	0.3012	0.6443	0.4221
D2_sm	1	-0.0882	0.3041	0.0842	0.7717

Odds Ratio Estimates			
Effect	Point Estimate	95% Wald Confidence Limits	
N3	1.022	0.972	1.074
age	1.011	0.983	1.041
BMI	1.250	1.201	1.300
SBP	0.994	0.980	1.009
D1_al	1.328	0.928	1.901
D2_al	1.467	0.971	2.218
D1_sm	1.274	0.706	2.298
D2_sm	0.916	0.504	1.662

Association of Predicted Probabilities and Observed Responses			
Percent Concordant	72.2	Somers' D	0.450
Percent Discordant	27.2	Gamma	0.453
Percent Tied	0.6	Tau-a	0.128
Pairs	410310	c	0.725

Logistic Regression Results

The LOGISTIC Procedure

Model Information	
Data Set	WORK.SORTTEMPTABLESORTED
Response Variable	LDL_x
Number of Response Levels	2
Model	binary logit
Optimization Technique	Fisher's scoring

Number of Observations Read	1701
Number of Observations Used	1701

Response Profile		
Ordered Value	LDL_x	Total Frequency
1	0	1384
2	1	317

Probability modeled is LDL_x='1'.

Model Convergence Status
Convergence criterion (GCONV=1E-8) satisfied.

Model Fit Statistics		
Criterion	Intercept Only	Intercept and Covariates
AIC	1638.032	1541.452
SC	1643.471	1590.403
-2 Log L	1636.032	1523.452

Testing Global Null Hypothesis: BETA=0			
Test	Chi-Square	DF	Pr > ChiSq
Likelihood Ratio	112.5799	8	<.0001
Score	122.0818	8	<.0001
Wald	106.1059	8	<.0001

Logistic Regression Results

The LOGISTIC Procedure

Analysis of Maximum Likelihood Estimates					
Parameter	DF	Estimate	Standard Error	Wald Chi-Square	Pr > ChiSq
Intercept	1	-7.2467	0.8347	75.3791	<.0001
N3	1	0.0352	0.0243	2.0979	0.1475
age	1	0.0871	0.0139	39.2106	<.0001
BMI	1	0.1325	0.0188	49.4757	<.0001
SBP	1	0.00231	0.00703	0.1085	0.7419
D1_al	1	0.0508	0.1817	0.0780	0.7800
D2_al	1	0.1522	0.2045	0.5540	0.4567
D1_sm	1	0.0992	0.2897	0.1172	0.7321
D2_sm	1	0.1737	0.2711	0.4103	0.5218

Odds Ratio Estimates			
Effect	Point Estimate	95% Wald Confidence Limits	
N3	1.036	0.988	1.086
age	1.091	1.062	1.121
BMI	1.142	1.100	1.185
SBP	1.002	0.989	1.016
D1_al	1.052	0.737	1.502
D2_al	1.164	0.780	1.738
D1_sm	1.104	0.626	1.948
D2_sm	1.190	0.699	2.024

Association of Predicted Probabilities and Observed Responses			
Percent Concordant	67.3	Somers' D	0.353
Percent Discordant	32.0	Gamma	0.355
Percent Tied	0.7	Tau-a	0.107
Pairs	438728	c	0.676

Logistic Regression Results

The LOGISTIC Procedure

Model Information	
Data Set	WORK.SORTTEMPTABLESORTED
Response Variable	TG_x
Number of Response Levels	2
Model	binary logit
Optimization Technique	Fisher's scoring

Number of Observations Read	1701
Number of Observations Used	1701

Response Profile		
Ordered Value	TG_x	Total Frequency
1	0	1614
2	1	87

Probability modeled is TG_x='1'.

Model Convergence Status
Convergence criterion (GCONV=1E-8) satisfied.

Model Fit Statistics		
Criterion	Intercept Only	Intercept and Covariates
AIC	688.785	587.494
SC	694.224	636.445
-2 Log L	686.785	569.494

Testing Global Null Hypothesis: BETA=0			
Test	Chi-Square	DF	Pr > ChiSq
Likelihood Ratio	117.2915	8	<.0001
Score	167.9677	8	<.0001
Wald	114.2158	8	<.0001

Logistic Regression Results

The LOGISTIC Procedure

Analysis of Maximum Likelihood Estimates					
Parameter	DF	Estimate	Standard Error	Wald Chi-Square	Pr > ChiSq
Intercept	1	-10.7753	1.3939	59.7583	<.0001
N3	1	0.0585	0.0443	1.7482	0.1861
age	1	0.0466	0.0239	3.8000	0.0513
BMI	1	0.2479	0.0289	73.7833	<.0001
SBP	1	0.00217	0.0118	0.0342	0.8533
D1_al	1	0.1280	0.3237	0.1563	0.6926
D2_al	1	0.4167	0.3413	1.4905	0.2221
D1_sm	1	-0.2105	0.6194	0.1154	0.7340
D2_sm	1	0.3424	0.4060	0.7112	0.3991

Odds Ratio Estimates			
Effect	Point Estimate	95% Wald Confidence Limits	
N3	1.060	0.972	1.156
age	1.048	1.000	1.098
BMI	1.281	1.211	1.356
SBP	1.002	0.979	1.026
D1_al	1.136	0.603	2.143
D2_al	1.517	0.777	2.961
D1_sm	0.810	0.241	2.728
D2_sm	1.408	0.635	3.121

Association of Predicted Probabilities and Observed Responses			
Percent Concordant	77.5	Somers' D	0.565
Percent Discordant	21.0	Gamma	0.573
Percent Tied	1.5	Tau-a	0.055
Pairs	140418	c	0.782

Logistic Regression Results

The LOGISTIC Procedure

Model Information	
Data Set	WORK.SORTTEMPTABLESORTED
Response Variable	TC_x
Number of Response Levels	2
Model	binary logit
Optimization Technique	Fisher's scoring

Number of Observations Read	1701
Number of Observations Used	1701

Response Profile		
Ordered Value	TC_x	Total Frequency
1	0	1386
2	1	315

Probability modeled is TC_x='1'.

Model Convergence Status
Convergence criterion (GCONV=1E-8) satisfied.

Model Fit Statistics		
Criterion	Intercept Only	Intercept and Covariates
AIC	1632.121	1581.317
SC	1637.560	1630.268
-2 Log L	1630.121	1563.317

Testing Global Null Hypothesis: BETA=0			
Test	Chi-Square	DF	Pr > ChiSq
Likelihood Ratio	66.8046	8	<.0001
Score	71.6516	8	<.0001
Wald	65.9414	8	<.0001

Logistic Regression Results

The LOGISTIC Procedure

Analysis of Maximum Likelihood Estimates					
Parameter	DF	Estimate	Standard Error	Wald Chi-Square	Pr > ChiSq
Intercept	1	-4.2320	0.8340	25.7475	<.0001
N4	1	-0.0425	0.0295	2.0694	0.1503
age	1	0.0582	0.0137	18.1628	<.0001
BMI	1	0.1141	0.0187	37.3480	<.0001
SBP	1	-0.00825	0.00701	1.3860	0.2391
D1_al	1	0.1681	0.1744	0.9296	0.3350
D2_al	1	0.3056	0.1979	2.3853	0.1225
D1_sm	1	0.1526	0.2804	0.2960	0.5864
D2_sm	1	-0.1136	0.2865	0.1571	0.6918

Odds Ratio Estimates			
Effect	Point Estimate	95% Wald Confidence Limits	
N4	0.958	0.905	1.016
age	1.060	1.032	1.089
BMI	1.121	1.081	1.163
SBP	0.992	0.978	1.005
D1_al	1.183	0.841	1.665
D2_al	1.357	0.921	2.001
D1_sm	1.165	0.672	2.018
D2_sm	0.893	0.509	1.565

Association of Predicted Probabilities and Observed Responses			
Percent Concordant	62.4	Somers' D	0.256
Percent Discordant	36.7	Gamma	0.259
Percent Tied	0.9	Tau-a	0.077
Pairs	436590	c	0.628

Logistic Regression Results

The LOGISTIC Procedure

Model Information	
Data Set	WORK.SORTTEMPTABLESORTED
Response Variable	HDL_x
Number of Response Levels	2
Model	binary logit
Optimization Technique	Fisher's scoring

Number of Observations Read	1701
Number of Observations Used	1701

Response Profile		
Ordered Value	HDL_x	Total Frequency
1	0	1410
2	1	291

Probability modeled is HDL_x='1'.

Model Convergence Status
Convergence criterion (GCONV=1E-8) satisfied.

Model Fit Statistics		
Criterion	Intercept Only	Intercept and Covariates
AIC	1558.714	1412.559
SC	1564.153	1461.510
-2 Log L	1556.714	1394.559

Testing Global Null Hypothesis: BETA=0			
Test	Chi-Square	DF	Pr > ChiSq
Likelihood Ratio	162.1554	8	<.0001
Score	185.1599	8	<.0001
Wald	145.4291	8	<.0001

Logistic Regression Results

The LOGISTIC Procedure

Analysis of Maximum Likelihood Estimates					
Parameter	DF	Estimate	Standard Error	Wald Chi-Square	Pr > ChiSq
Intercept	1	-6.6242	0.8899	55.4127	<.0001
N4	1	0.0273	0.0315	0.7466	0.3876
age	1	0.0111	0.0146	0.5770	0.4475
BMI	1	0.2241	0.0203	122.0597	<.0001
SBP	1	-0.00574	0.00734	0.6119	0.4341
D1_al	1	0.2782	0.1828	2.3146	0.1282
D2_al	1	0.3885	0.2107	3.3986	0.0653
D1_sm	1	0.2474	0.3014	0.6739	0.4117
D2_sm	1	-0.0802	0.3044	0.0694	0.7923

Odds Ratio Estimates			
Effect	Point Estimate	95% Wald Confidence Limits	
N4	1.028	0.966	1.093
age	1.011	0.983	1.041
BMI	1.251	1.202	1.302
SBP	0.994	0.980	1.009
D1_al	1.321	0.923	1.890
D2_al	1.475	0.976	2.229
D1_sm	1.281	0.709	2.312
D2_sm	0.923	0.508	1.676

Association of Predicted Probabilities and Observed Responses			
Percent Concordant	72.1	Somers' D	0.448
Percent Discordant	27.3	Gamma	0.450
Percent Tied	0.6	Tau-a	0.127
Pairs	410310	c	0.724

Logistic Regression Results

The LOGISTIC Procedure

Model Information	
Data Set	WORK.SORTTEMPTABLESORTED
Response Variable	LDL_x
Number of Response Levels	2
Model	binary logit
Optimization Technique	Fisher's scoring

Number of Observations Read	1701
Number of Observations Used	1701

Response Profile		
Ordered Value	LDL_x	Total Frequency
1	0	1384
2	1	317

Probability modeled is LDL_x='1'.

Model Convergence Status
Convergence criterion (GCONV=1E-8) satisfied.

Model Fit Statistics		
Criterion	Intercept Only	Intercept and Covariates
AIC	1638.032	1542.084
SC	1643.471	1591.035
-2 Log L	1636.032	1524.084

Testing Global Null Hypothesis: BETA=0			
Test	Chi-Square	DF	Pr > ChiSq
Likelihood Ratio	111.9478	8	<.0001
Score	121.8903	8	<.0001
Wald	105.0679	8	<.0001

Logistic Regression Results

The LOGISTIC Procedure

Analysis of Maximum Likelihood Estimates					
Parameter	DF	Estimate	Standard Error	Wald Chi-Square	Pr > ChiSq
Intercept	1	-6.4681	0.8524	57.5808	<.0001
N4	1	-0.0363	0.0300	1.4693	0.2255
age	1	0.0819	0.0138	35.0608	<.0001
BMI	1	0.1345	0.0189	50.8846	<.0001
SBP	1	0.00231	0.00702	0.1081	0.7423
D1_al	1	0.0416	0.1820	0.0522	0.8193
D2_al	1	0.1663	0.2043	0.6631	0.4155
D1_sm	1	0.0872	0.2888	0.0912	0.7626
D2_sm	1	0.1744	0.2719	0.4114	0.5213

Odds Ratio Estimates			
Effect	Point Estimate	95% Wald Confidence Limits	
N4	0.964	0.909	1.023
age	1.085	1.056	1.115
BMI	1.144	1.102	1.187
SBP	1.002	0.989	1.016
D1_al	1.042	0.730	1.489
D2_al	1.181	0.791	1.762
D1_sm	1.091	0.620	1.922
D2_sm	1.191	0.699	2.028

Association of Predicted Probabilities and Observed Responses			
Percent Concordant	67.2	Somers' D	0.351
Percent Discordant	32.1	Gamma	0.354
Percent Tied	0.7	Tau-a	0.107
Pairs	438728	c	0.676

Logistic Regression Results

The LOGISTIC Procedure

Model Information	
Data Set	WORK.SORTTEMPTABLESORTED
Response Variable	TG_x
Number of Response Levels	2
Model	binary logit
Optimization Technique	Fisher's scoring

Number of Observations Read	1701
Number of Observations Used	1701

Response Profile		
Ordered Value	TG_x	Total Frequency
1	0	1614
2	1	87

Probability modeled is TG_x='1'.

Model Convergence Status
Convergence criterion (GCONV=1E-8) satisfied.

Model Fit Statistics		
Criterion	Intercept Only	Intercept and Covariates
AIC	688.785	589.169
SC	694.224	638.119
-2 Log L	686.785	571.169

Testing Global Null Hypothesis: BETA=0			
Test	Chi-Square	DF	Pr > ChiSq
Likelihood Ratio	115.6168	8	<.0001
Score	166.9621	8	<.0001
Wald	113.0940	8	<.0001

Logistic Regression Results

The LOGISTIC Procedure

Analysis of Maximum Likelihood Estimates					
Parameter	DF	Estimate	Standard Error	Wald Chi-Square	Pr > ChiSq
Intercept	1	-10.2582	1.4362	51.0137	<.0001
N4	1	0.0153	0.0545	0.0788	0.7790
age	1	0.0416	0.0238	3.0434	0.0811
BMI	1	0.2505	0.0289	75.2257	<.0001
SBP	1	0.00185	0.0117	0.0250	0.8744
D1_al	1	0.1054	0.3235	0.1062	0.7445
D2_al	1	0.4270	0.3413	1.5651	0.2109
D1_sm	1	-0.2032	0.6187	0.1079	0.7426
D2_sm	1	0.3409	0.4074	0.7005	0.4026

Odds Ratio Estimates			
Effect	Point Estimate	95% Wald Confidence Limits	
N4	1.015	0.913	1.130
age	1.042	0.995	1.092
BMI	1.285	1.214	1.359
SBP	1.002	0.979	1.025
D1_al	1.111	0.589	2.095
D2_al	1.533	0.785	2.992
D1_sm	0.816	0.243	2.744
D2_sm	1.406	0.633	3.125

Association of Predicted Probabilities and Observed Responses			
Percent Concordant	77.3	Somers' D	0.562
Percent Discordant	21.1	Gamma	0.571
Percent Tied	1.5	Tau-a	0.055
Pairs	140418	c	0.781

Logistic Regression Results

The LOGISTIC Procedure

Model Information	
Data Set	WORK.SORTTEMPTABLESORTED
Response Variable	TC_x
Number of Response Levels	2
Model	binary logit
Optimization Technique	Fisher's scoring

Number of Observations Read	1701
Number of Observations Used	1701

Response Profile		
Ordered Value	TC_x	Total Frequency
1	0	1386
2	1	315

Probability modeled is TC_x='1'.

Model Convergence Status
Convergence criterion (GCONV=1E-8) satisfied.

Model Fit Statistics		
Criterion	Intercept Only	Intercept and Covariates
AIC	1632.121	1583.260
SC	1637.560	1632.210
-2 Log L	1630.121	1565.260

Testing Global Null Hypothesis: BETA=0			
Test	Chi-Square	DF	Pr > ChiSq
Likelihood Ratio	64.8619	8	<.0001
Score	69.5088	8	<.0001
Wald	64.2795	8	<.0001

Logistic Regression Results

The LOGISTIC Procedure

Analysis of Maximum Likelihood Estimates					
Parameter	DF	Estimate	Standard Error	Wald Chi-Square	Pr > ChiSq
Intercept	1	-4.8268	0.8471	32.4686	<.0001
N5	1	0.0110	0.0309	0.1273	0.7212
age	1	0.0618	0.0139	19.8231	<.0001
BMI	1	0.1129	0.0189	35.8601	<.0001
SBP	1	-0.00818	0.00702	1.3564	0.2442
D1_al	1	0.1712	0.1742	0.9657	0.3258
D2_al	1	0.2993	0.1979	2.2882	0.1304
D1_sm	1	0.1596	0.2808	0.3230	0.5698
D2_sm	1	-0.1099	0.2865	0.1471	0.7013

Odds Ratio Estimates			
Effect	Point Estimate	95% Wald Confidence Limits	
N5	1.011	0.952	1.074
age	1.064	1.035	1.093
BMI	1.120	1.079	1.162
SBP	0.992	0.978	1.006
D1_al	1.187	0.843	1.669
D2_al	1.349	0.915	1.988
D1_sm	1.173	0.676	2.034
D2_sm	0.896	0.511	1.571

Association of Predicted Probabilities and Observed Responses			
Percent Concordant	62.0	Somers' D	0.249
Percent Discordant	37.1	Gamma	0.252
Percent Tied	0.9	Tau-a	0.075
Pairs	436590	c	0.625

Logistic Regression Results

The LOGISTIC Procedure

Model Information	
Data Set	WORK.SORTTEMPTABLESORTED
Response Variable	HDL_x
Number of Response Levels	2
Model	binary logit
Optimization Technique	Fisher's scoring

Number of Observations Read	1701
Number of Observations Used	1701

Response Profile		
Ordered Value	HDL_x	Total Frequency
1	0	1410
2	1	291

Probability modeled is HDL_x='1'.

Model Convergence Status
Convergence criterion (GCONV=1E-8) satisfied.

Model Fit Statistics		
Criterion	Intercept Only	Intercept and Covariates
AIC	1558.714	1412.857
SC	1564.153	1461.808
-2 Log L	1556.714	1394.857

Testing Global Null Hypothesis: BETA=0			
Test	Chi-Square	DF	Pr > ChiSq
Likelihood Ratio	161.8570	8	<.0001
Score	184.7412	8	<.0001
Wald	145.2592	8	<.0001

Logistic Regression Results

The LOGISTIC Procedure

Analysis of Maximum Likelihood Estimates					
Parameter	DF	Estimate	Standard Error	Wald Chi-Square	Pr > ChiSq
Intercept	1	-6.5731	0.9012	53.2004	<.0001
N5	1	0.0223	0.0333	0.4494	0.5026
age	1	0.0116	0.0149	0.6116	0.4342
BMI	1	0.2220	0.0205	117.2735	<.0001
SBP	1	-0.00539	0.00736	0.5371	0.4636
D1_al	1	0.2818	0.1829	2.3739	0.1234
D2_al	1	0.3846	0.2110	3.3211	0.0684
D1_sm	1	0.2334	0.3011	0.6008	0.4383
D2_sm	1	-0.1027	0.3051	0.1132	0.7365

Odds Ratio Estimates			
Effect	Point Estimate	95% Wald Confidence Limits	
N5	1.023	0.958	1.091
age	1.012	0.983	1.042
BMI	1.249	1.199	1.300
SBP	0.995	0.980	1.009
D1_al	1.326	0.926	1.897
D2_al	1.469	0.971	2.221
D1_sm	1.263	0.700	2.279
D2_sm	0.902	0.496	1.641

Association of Predicted Probabilities and Observed Responses			
Percent Concordant	72.3	Somers' D	0.452
Percent Discordant	27.1	Gamma	0.455
Percent Tied	0.6	Tau-a	0.128
Pairs	410310	c	0.726

Logistic Regression Results

The LOGISTIC Procedure

Model Information	
Data Set	WORK.SORTTEMPTABLESORTED
Response Variable	LDL_x
Number of Response Levels	2
Model	binary logit
Optimization Technique	Fisher's scoring

Number of Observations Read	1701
Number of Observations Used	1701

Response Profile		
Ordered Value	LDL_x	Total Frequency
1	0	1384
2	1	317

Probability modeled is LDL_x='1'.

Model Convergence Status
Convergence criterion (GCONV=1E-8) satisfied.

Model Fit Statistics		
Criterion	Intercept Only	Intercept and Covariates
AIC	1638.032	1543.514
SC	1643.471	1592.464
-2 Log L	1636.032	1525.514

Testing Global Null Hypothesis: BETA=0			
Test	Chi-Square	DF	Pr > ChiSq
Likelihood Ratio	110.5186	8	<.0001
Score	120.2949	8	<.0001
Wald	104.1646	8	<.0001

Logistic Regression Results

The LOGISTIC Procedure

Analysis of Maximum Likelihood Estimates					
Parameter	DF	Estimate	Standard Error	Wald Chi-Square	Pr > ChiSq
Intercept	1	-6.9359	0.8665	64.0731	<.0001
N5	1	0.00631	0.0314	0.0404	0.8407
age	1	0.0846	0.0141	36.2349	<.0001
BMI	1	0.1337	0.0191	49.2668	<.0001
SBP	1	0.00230	0.00703	0.1072	0.7433
D1_al	1	0.0429	0.1818	0.0557	0.8135
D2_al	1	0.1626	0.2043	0.6334	0.4261
D1_sm	1	0.0934	0.2893	0.1041	0.7469
D2_sm	1	0.1768	0.2724	0.4212	0.5163

Odds Ratio Estimates			
Effect	Point Estimate	95% Wald Confidence Limits	
N5	1.006	0.946	1.070
age	1.088	1.059	1.119
BMI	1.143	1.101	1.187
SBP	1.002	0.989	1.016
D1_al	1.044	0.731	1.491
D2_al	1.177	0.788	1.756
D1_sm	1.098	0.623	1.936
D2_sm	1.193	0.700	2.035

Association of Predicted Probabilities and Observed Responses			
Percent Concordant	67.1	Somers' D	0.348
Percent Discordant	32.3	Gamma	0.350
Percent Tied	0.7	Tau-a	0.106
Pairs	438728	c	0.674

Logistic Regression Results

The LOGISTIC Procedure

Model Information	
Data Set	WORK.SORTTEMPTABLESORTED
Response Variable	TG_x
Number of Response Levels	2
Model	binary logit
Optimization Technique	Fisher's scoring

Number of Observations Read	1701
Number of Observations Used	1701

Response Profile		
Ordered Value	TG_x	Total Frequency
1	0	1614
2	1	87

Probability modeled is TG_x='1'.

Model Convergence Status
Convergence criterion (GCONV=1E-8) satisfied.

Model Fit Statistics		
Criterion	Intercept Only	Intercept and Covariates
AIC	688.785	587.724
SC	694.224	636.675
-2 Log L	686.785	569.724

Testing Global Null Hypothesis: BETA=0			
Test	Chi-Square	DF	Pr > ChiSq
Likelihood Ratio	117.0612	8	<.0001
Score	167.5183	8	<.0001
Wald	113.3763	8	<.0001

Logistic Regression Results

The LOGISTIC Procedure

Analysis of Maximum Likelihood Estimates					
Parameter	DF	Estimate	Standard Error	Wald Chi-Square	Pr > ChiSq
Intercept	1	-10.9537	1.4756	55.1054	<.0001
N5	1	0.0714	0.0579	1.5220	0.2173
age	1	0.0485	0.0243	3.9864	0.0459
BMI	1	0.2456	0.0291	70.9824	<.0001
SBP	1	0.00268	0.0118	0.0519	0.8199
D1_al	1	0.1296	0.3234	0.1606	0.6886
D2_al	1	0.4079	0.3425	1.4189	0.2336
D1_sm	1	-0.2248	0.6176	0.1325	0.7159
D2_sm	1	0.2912	0.4082	0.5089	0.4756

Odds Ratio Estimates			
Effect	Point Estimate	95% Wald Confidence Limits	
N5	1.074	0.959	1.203
age	1.050	1.001	1.101
BMI	1.278	1.207	1.353
SBP	1.003	0.980	1.026
D1_al	1.138	0.604	2.146
D2_al	1.504	0.769	2.942
D1_sm	0.799	0.238	2.680
D2_sm	1.338	0.601	2.978

Association of Predicted Probabilities and Observed Responses			
Percent Concordant	77.7	Somers' D	0.568
Percent Discordant	20.9	Gamma	0.576
Percent Tied	1.4	Tau-a	0.055
Pairs	140418	c	0.784

Logistic Regression Results

The LOGISTIC Procedure

Model Information	
Data Set	WORK.SORTTEMPTABLESORTED
Response Variable	TC_x
Number of Response Levels	2
Model	binary logit
Optimization Technique	Fisher's scoring

Number of Observations Read	1701
Number of Observations Used	1701

Response Profile		
Ordered Value	TC_x	Total Frequency
1	0	1386
2	1	315

Probability modeled is TC_x='1'.

Model Convergence Status
Convergence criterion (GCONV=1E-8) satisfied.

Model Fit Statistics		
Criterion	Intercept Only	Intercept and Covariates
AIC	1632.121	1583.008
SC	1637.560	1631.959
-2 Log L	1630.121	1565.008

Testing Global Null Hypothesis: BETA=0			
Test	Chi-Square	DF	Pr > ChiSq
Likelihood Ratio	65.1135	8	<.0001
Score	69.8528	8	<.0001
Wald	64.4388	8	<.0001

Logistic Regression Results

The LOGISTIC Procedure

Analysis of Maximum Likelihood Estimates					
Parameter	DF	Estimate	Standard Error	Wald Chi-Square	Pr > ChiSq
Intercept	1	-4.5079	0.8302	29.4815	<.0001
N6	1	-0.0186	0.0302	0.3792	0.5380
age	1	0.0597	0.0137	19.0695	<.0001
BMI	1	0.1140	0.0186	37.4182	<.0001
SBP	1	-0.00823	0.00701	1.3776	0.2405
D1_al	1	0.1670	0.1741	0.9195	0.3376
D2_al	1	0.3076	0.1980	2.4134	0.1203
D1_sm	1	0.1603	0.2806	0.3262	0.5679
D2_sm	1	-0.1035	0.2860	0.1310	0.7174

Odds Ratio Estimates			
Effect	Point Estimate	95% Wald Confidence Limits	
N6	0.982	0.925	1.041
age	1.061	1.033	1.090
BMI	1.121	1.081	1.162
SBP	0.992	0.978	1.006
D1_al	1.182	0.840	1.662
D2_al	1.360	0.923	2.005
D1_sm	1.174	0.677	2.035
D2_sm	0.902	0.515	1.579

Association of Predicted Probabilities and Observed Responses			
Percent Concordant	62.1	Somers' D	0.251
Percent Discordant	37.0	Gamma	0.254
Percent Tied	0.9	Tau-a	0.076
Pairs	436590	c	0.626

Logistic Regression Results

The LOGISTIC Procedure

Model Information	
Data Set	WORK.SORTTEMPTABLESORTED
Response Variable	HDL_x
Number of Response Levels	2
Model	binary logit
Optimization Technique	Fisher's scoring

Number of Observations Read	1701
Number of Observations Used	1701

Response Profile		
Ordered Value	HDL_x	Total Frequency
1	0	1410
2	1	291

Probability modeled is HDL_x='1'.

Model Convergence Status
Convergence criterion (GCONV=1E-8) satisfied.

Model Fit Statistics		
Criterion	Intercept Only	Intercept and Covariates
AIC	1558.714	1411.151
SC	1564.153	1460.102
-2 Log L	1556.714	1393.151

Testing Global Null Hypothesis: BETA=0			
Test	Chi-Square	DF	Pr > ChiSq
Likelihood Ratio	163.5635	8	<.0001
Score	186.6466	8	<.0001
Wald	146.5948	8	<.0001

Logistic Regression Results

The LOGISTIC Procedure

Analysis of Maximum Likelihood Estimates					
Parameter	DF	Estimate	Standard Error	Wald Chi-Square	Pr > ChiSq
Intercept	1	-6.8102	0.8831	59.4748	<.0001
N6	1	0.0472	0.0322	2.1468	0.1429
age	1	0.0123	0.0146	0.7101	0.3994
BMI	1	0.2241	0.0203	121.9377	<.0001
SBP	1	-0.00601	0.00735	0.6688	0.4135
D1_al	1	0.2824	0.1831	2.3791	0.1230
D2_al	1	0.3743	0.2111	3.1429	0.0763
D1_sm	1	0.2485	0.3012	0.6809	0.4093
D2_sm	1	-0.0827	0.3043	0.0739	0.7857

Odds Ratio Estimates			
Effect	Point Estimate	95% Wald Confidence Limits	
N6	1.048	0.984	1.117
age	1.012	0.984	1.042
BMI	1.251	1.202	1.302
SBP	0.994	0.980	1.008
D1_al	1.326	0.926	1.899
D2_al	1.454	0.961	2.199
D1_sm	1.282	0.710	2.314
D2_sm	0.921	0.507	1.671

Association of Predicted Probabilities and Observed Responses			
Percent Concordant	72.4	Somers' D	0.454
Percent Discordant	27.0	Gamma	0.457
Percent Tied	0.6	Tau-a	0.129
Pairs	410310	c	0.727

Logistic Regression Results

The LOGISTIC Procedure

Model Information	
Data Set	WORK.SORTTEMPTABLESORTED
Response Variable	LDL_x
Number of Response Levels	2
Model	binary logit
Optimization Technique	Fisher's scoring

Number of Observations Read	1701
Number of Observations Used	1701

Response Profile		
Ordered Value	LDL_x	Total Frequency
1	0	1384
2	1	317

Probability modeled is LDL_x='1'.

Model Convergence Status
Convergence criterion (GCONV=1E-8) satisfied.

Model Fit Statistics		
Criterion	Intercept Only	Intercept and Covariates
AIC	1638.032	1543.416
SC	1643.471	1592.367
-2 Log L	1636.032	1525.416

Testing Global Null Hypothesis: BETA=0			
Test	Chi-Square	DF	Pr > ChiSq
Likelihood Ratio	110.6165	8	<.0001
Score	120.4517	8	<.0001
Wald	104.1367	8	<.0001

Logistic Regression Results

The LOGISTIC Procedure

Analysis of Maximum Likelihood Estimates					
Parameter	DF	Estimate	Standard Error	Wald Chi-Square	Pr > ChiSq
Intercept	1	-6.7468	0.8492	63.1217	<.0001
N6	1	-0.0114	0.0306	0.1383	0.7100
age	1	0.0834	0.0138	36.3089	<.0001
BMI	1	0.1344	0.0188	50.9260	<.0001
SBP	1	0.00228	0.00702	0.1055	0.7453
D1_al	1	0.0406	0.1817	0.0499	0.8232
D2_al	1	0.1677	0.2044	0.6733	0.4119
D1_sm	1	0.0936	0.2891	0.1049	0.7460
D2_sm	1	0.1811	0.2715	0.4450	0.5047

Odds Ratio Estimates			
Effect	Point Estimate	95% Wald Confidence Limits	
N6	0.989	0.931	1.050
age	1.087	1.058	1.117
BMI	1.144	1.102	1.187
SBP	1.002	0.989	1.016
D1_al	1.041	0.729	1.487
D2_al	1.183	0.792	1.765
D1_sm	1.098	0.623	1.936
D2_sm	1.199	0.704	2.041

Association of Predicted Probabilities and Observed Responses			
Percent Concordant	67.1	Somers' D	0.348
Percent Discordant	32.3	Gamma	0.351
Percent Tied	0.7	Tau-a	0.106
Pairs	438728	c	0.674

Logistic Regression Results

The LOGISTIC Procedure

Model Information	
Data Set	WORK.SORTTEMPTABLESORTED
Response Variable	TG_x
Number of Response Levels	2
Model	binary logit
Optimization Technique	Fisher's scoring

Number of Observations Read	1701
Number of Observations Used	1701

Response Profile		
Ordered Value	TG_x	Total Frequency
1	0	1614
2	1	87

Probability modeled is TG_x='1'.

Model Convergence Status
Convergence criterion (GCONV=1E-8) satisfied.

Model Fit Statistics		
Criterion	Intercept Only	Intercept and Covariates
AIC	688.785	588.796
SC	694.224	637.746
-2 Log L	686.785	570.796

Testing Global Null Hypothesis: BETA=0			
Test	Chi-Square	DF	Pr > ChiSq
Likelihood Ratio	115.9898	8	<.0001
Score	167.2923	8	<.0001
Wald	113.5265	8	<.0001

Logistic Regression Results

The LOGISTIC Procedure

Analysis of Maximum Likelihood Estimates					
Parameter	DF	Estimate	Standard Error	Wald Chi-Square	Pr > ChiSq
Intercept	1	-10.4648	1.4069	55.3276	<.0001
N6	1	0.0367	0.0546	0.4509	0.5019
age	1	0.0432	0.0238	3.2984	0.0693
BMI	1	0.2505	0.0289	75.1992	<.0001
SBP	1	0.00152	0.0118	0.0167	0.8973
D1_al	1	0.1052	0.3238	0.1056	0.7452
D2_al	1	0.4143	0.3414	1.4729	0.2249
D1_sm	1	-0.1987	0.6182	0.1033	0.7479
D2_sm	1	0.3421	0.4072	0.7057	0.4009

Odds Ratio Estimates			
Effect	Point Estimate	95% Wald Confidence Limits	
N6	1.037	0.932	1.155
age	1.044	0.997	1.094
BMI	1.285	1.214	1.359
SBP	1.002	0.979	1.025
D1_al	1.111	0.589	2.096
D2_al	1.513	0.775	2.955
D1_sm	0.820	0.244	2.754
D2_sm	1.408	0.634	3.127

Association of Predicted Probabilities and Observed Responses			
Percent Concordant	77.4	Somers' D	0.563
Percent Discordant	21.1	Gamma	0.571
Percent Tied	1.5	Tau-a	0.055
Pairs	140418	c	0.781

Logistic Regression Results

The LOGISTIC Procedure

Model Information	
Data Set	WORK.SORTTEMPTABLESORTED
Response Variable	TC_x
Number of Response Levels	2
Model	binary logit
Optimization Technique	Fisher's scoring

Number of Observations Read	1701
Number of Observations Used	1701

Response Profile		
Ordered Value	TC_x	Total Frequency
1	0	1386
2	1	315

Probability modeled is TC_x='1'.

Model Convergence Status
Convergence criterion (GCONV=1E-8) satisfied.

Model Fit Statistics		
Criterion	Intercept Only	Intercept and Covariates
AIC	1632.121	1583.012
SC	1637.560	1631.963
-2 Log L	1630.121	1565.012

Testing Global Null Hypothesis: BETA=0			
Test	Chi-Square	DF	Pr > ChiSq
Likelihood Ratio	65.1091	8	<.0001
Score	69.7448	8	<.0001
Wald	64.4819	8	<.0001

Logistic Regression Results

The LOGISTIC Procedure

Analysis of Maximum Likelihood Estimates					
Parameter	DF	Estimate	Standard Error	Wald Chi-Square	Pr > ChiSq
Intercept	1	-4.4947	0.8385	28.7320	<.0001
E1	1	-0.0190	0.0310	0.3756	0.5400
age	1	0.0603	0.0136	19.7304	<.0001
BMI	1	0.1147	0.0187	37.7629	<.0001
SBP	1	-0.00832	0.00700	1.4118	0.2348
D1_al	1	0.1672	0.1741	0.9218	0.3370
D2_al	1	0.2977	0.1979	2.2638	0.1324
D1_sm	1	0.1621	0.2809	0.3329	0.5639
D2_sm	1	-0.0984	0.2857	0.1186	0.7305

Odds Ratio Estimates			
Effect	Point Estimate	95% Wald Confidence Limits	
E1	0.981	0.923	1.043
age	1.062	1.034	1.091
BMI	1.122	1.081	1.163
SBP	0.992	0.978	1.005
D1_al	1.182	0.840	1.663
D2_al	1.347	0.914	1.985
D1_sm	1.176	0.678	2.039
D2_sm	0.906	0.518	1.587

Association of Predicted Probabilities and Observed Responses			
Percent Concordant	61.9	Somers' D	0.248
Percent Discordant	37.2	Gamma	0.250
Percent Tied	0.9	Tau-a	0.075
Pairs	436590	c	0.624

Logistic Regression Results

The LOGISTIC Procedure

Model Information	
Data Set	WORK.SORTTEMPTABLESORTED
Response Variable	HDL_x
Number of Response Levels	2
Model	binary logit
Optimization Technique	Fisher's scoring

Number of Observations Read	1701
Number of Observations Used	1701

Response Profile		
Ordered Value	HDL_x	Total Frequency
1	0	1410
2	1	291

Probability modeled is HDL_x='1'.

Model Convergence Status
Convergence criterion (GCONV=1E-8) satisfied.

Model Fit Statistics		
Criterion	Intercept Only	Intercept and Covariates
AIC	1558.714	1413.258
SC	1564.153	1462.209
-2 Log L	1556.714	1395.258

Testing Global Null Hypothesis: BETA=0			
Test	Chi-Square	DF	Pr > ChiSq
Likelihood Ratio	161.4565	8	<.0001
Score	184.5435	8	<.0001
Wald	144.8977	8	<.0001

Logistic Regression Results

The LOGISTIC Procedure

Analysis of Maximum Likelihood Estimates					
Parameter	DF	Estimate	Standard Error	Wald Chi-Square	Pr > ChiSq
Intercept	1	-6.2342	0.8923	48.8175	<.0001
E1	1	-0.00732	0.0330	0.0493	0.8243
age	1	0.00920	0.0145	0.4019	0.5261
BMI	1	0.2244	0.0203	122.1152	<.0001
SBP	1	-0.00571	0.00734	0.6056	0.4364
D1_al	1	0.2775	0.1829	2.3031	0.1291
D2_al	1	0.3881	0.2108	3.3881	0.0657
D1_sm	1	0.2408	0.3008	0.6410	0.4233
D2_sm	1	-0.0858	0.3047	0.0793	0.7782

Odds Ratio Estimates			
Effect	Point Estimate	95% Wald Confidence Limits	
E1	0.993	0.931	1.059
age	1.009	0.981	1.038
BMI	1.252	1.203	1.302
SBP	0.994	0.980	1.009
D1_al	1.320	0.922	1.889
D2_al	1.474	0.975	2.228
D1_sm	1.272	0.706	2.294
D2_sm	0.918	0.505	1.668

Association of Predicted Probabilities and Observed Responses			
Percent Concordant	72.1	Somers' D	0.449
Percent Discordant	27.2	Gamma	0.452
Percent Tied	0.6	Tau-a	0.127
Pairs	410310	c	0.724

Logistic Regression Results

The LOGISTIC Procedure

Model Information	
Data Set	WORK.SORTTEMPTABLESORTED
Response Variable	LDL_x
Number of Response Levels	2
Model	binary logit
Optimization Technique	Fisher's scoring

Number of Observations Read	1701
Number of Observations Used	1701

Response Profile		
Ordered Value	LDL_x	Total Frequency
1	0	1384
2	1	317

Probability modeled is LDL_x='1'.

Model Convergence Status
Convergence criterion (GCONV=1E-8) satisfied.

Model Fit Statistics		
Criterion	Intercept Only	Intercept and Covariates
AIC	1638.032	1542.570
SC	1643.471	1591.521
-2 Log L	1636.032	1524.570

Testing Global Null Hypothesis: BETA=0			
Test	Chi-Square	DF	Pr > ChiSq
Likelihood Ratio	111.4624	8	<.0001
Score	121.0613	8	<.0001
Wald	104.9360	8	<.0001

Logistic Regression Results

The LOGISTIC Procedure

Analysis of Maximum Likelihood Estimates					
Parameter	DF	Estimate	Standard Error	Wald Chi-Square	Pr > ChiSq
Intercept	1	-6.5258	0.8565	58.0545	<.0001
E1	1	-0.0313	0.0315	0.9880	0.3202
age	1	0.0833	0.0137	36.7937	<.0001
BMI	1	0.1355	0.0189	51.6242	<.0001
SBP	1	0.00222	0.00701	0.0999	0.7520
D1_al	1	0.0388	0.1817	0.0455	0.8310
D2_al	1	0.1574	0.2043	0.5930	0.4413
D1_sm	1	0.0938	0.2896	0.1049	0.7461
D2_sm	1	0.1891	0.2713	0.4855	0.4859

Odds Ratio Estimates			
Effect	Point Estimate	95% Wald Confidence Limits	
E1	0.969	0.911	1.031
age	1.087	1.058	1.117
BMI	1.145	1.104	1.188
SBP	1.002	0.989	1.016
D1_al	1.040	0.728	1.484
D2_al	1.170	0.784	1.747
D1_sm	1.098	0.623	1.938
D2_sm	1.208	0.710	2.056

Association of Predicted Probabilities and Observed Responses			
Percent Concordant	67.1	Somers' D	0.349
Percent Discordant	32.2	Gamma	0.351
Percent Tied	0.7	Tau-a	0.106
Pairs	438728	c	0.675

Logistic Regression Results

The LOGISTIC Procedure

Model Information	
Data Set	WORK.SORTTEMPTABLESORTED
Response Variable	TG_x
Number of Response Levels	2
Model	binary logit
Optimization Technique	Fisher's scoring

Number of Observations Read	1701
Number of Observations Used	1701

Response Profile		
Ordered Value	TG_x	Total Frequency
1	0	1614
2	1	87

Probability modeled is TG_x='1'.

Model Convergence Status
Convergence criterion (GCONV=1E-8) satisfied.

Model Fit Statistics		
Criterion	Intercept Only	Intercept and Covariates
AIC	688.785	586.680
SC	694.224	635.631
-2 Log L	686.785	568.680

Testing Global Null Hypothesis: BETA=0			
Test	Chi-Square	DF	Pr > ChiSq
Likelihood Ratio	118.1052	8	<.0001
Score	169.0093	8	<.0001
Wald	113.8478	8	<.0001

Logistic Regression Results

The LOGISTIC Procedure

Analysis of Maximum Likelihood Estimates					
Parameter	DF	Estimate	Standard Error	Wald Chi-Square	Pr > ChiSq
Intercept	1	-9.1648	1.3852	43.7736	<.0001
E1	1	-0.0892	0.0552	2.6080	0.1063
age	1	0.0409	0.0233	3.0770	0.0794
BMI	1	0.2549	0.0291	76.4772	<.0001
SBP	1	0.00143	0.0116	0.0150	0.9026
D1_al	1	0.0969	0.3246	0.0890	0.7654
D2_al	1	0.4118	0.3414	1.4547	0.2278
D1_sm	1	-0.2338	0.6211	0.1417	0.7066
D2_sm	1	0.3493	0.4101	0.7257	0.3943

Odds Ratio Estimates			
Effect	Point Estimate	95% Wald Confidence Limits	
E1	0.915	0.821	1.019
age	1.042	0.995	1.090
BMI	1.290	1.219	1.366
SBP	1.001	0.979	1.025
D1_al	1.102	0.583	2.081
D2_al	1.510	0.773	2.948
D1_sm	0.792	0.234	2.674
D2_sm	1.418	0.635	3.168

Association of Predicted Probabilities and Observed Responses			
Percent Concordant	78.3	Somers' D	0.580
Percent Discordant	20.3	Gamma	0.589
Percent Tied	1.4	Tau-a	0.056
Pairs	140418	c	0.790

Logistic Regression Results

The LOGISTIC Procedure

Model Information	
Data Set	WORK.SORTTEMPTABLESORTED
Response Variable	TC_x
Number of Response Levels	2
Model	binary logit
Optimization Technique	Fisher's scoring

Number of Observations Read	1701
Number of Observations Used	1701

Response Profile		
Ordered Value	TC_x	Total Frequency
1	0	1386
2	1	315

Probability modeled is TC_x='1'.

Model Convergence Status
Convergence criterion (GCONV=1E-8) satisfied.

Model Fit Statistics		
Criterion	Intercept Only	Intercept and Covariates
AIC	1632.121	1583.282
SC	1637.560	1632.232
-2 Log L	1630.121	1565.282

Testing Global Null Hypothesis: BETA=0			
Test	Chi-Square	DF	Pr > ChiSq
Likelihood Ratio	64.8397	8	<.0001
Score	69.5449	8	<.0001
Wald	64.2510	8	<.0001

Logistic Regression Results

The LOGISTIC Procedure

Analysis of Maximum Likelihood Estimates					
Parameter	DF	Estimate	Standard Error	Wald Chi-Square	Pr > ChiSq
Intercept	1	-4.6032	0.8257	31.0769	<.0001
E2	1	-0.00968	0.0298	0.1052	0.7457
age	1	0.0603	0.0136	19.5763	<.0001
BMI	1	0.1140	0.0186	37.4156	<.0001
SBP	1	-0.00828	0.00701	1.3962	0.2374
D1_al	1	0.1663	0.1743	0.9108	0.3399
D2_al	1	0.2993	0.1979	2.2882	0.1304
D1_sm	1	0.1629	0.2807	0.3368	0.5617
D2_sm	1	-0.1014	0.2858	0.1257	0.7229

Odds Ratio Estimates			
Effect	Point Estimate	95% Wald Confidence Limits	
E2	0.990	0.934	1.050
age	1.062	1.034	1.091
BMI	1.121	1.081	1.162
SBP	0.992	0.978	1.005
D1_al	1.181	0.839	1.662
D2_al	1.349	0.915	1.988
D1_sm	1.177	0.679	2.040
D2_sm	0.904	0.516	1.582

Association of Predicted Probabilities and Observed Responses			
Percent Concordant	62.1	Somers' D	0.250
Percent Discordant	37.0	Gamma	0.252
Percent Tied	0.9	Tau-a	0.076
Pairs	436590	c	0.625

Logistic Regression Results

The LOGISTIC Procedure

Model Information	
Data Set	WORK.SORTTEMPTABLESORTED
Response Variable	HDL_x
Number of Response Levels	2
Model	binary logit
Optimization Technique	Fisher's scoring

Number of Observations Read	1701
Number of Observations Used	1701

Response Profile		
Ordered Value	HDL_x	Total Frequency
1	0	1410
2	1	291

Probability modeled is HDL_x='1'.

Model Convergence Status
Convergence criterion (GCONV=1E-8) satisfied.

Model Fit Statistics		
Criterion	Intercept Only	Intercept and Covariates
AIC	1558.714	1412.385
SC	1564.153	1461.336
-2 Log L	1556.714	1394.385

Testing Global Null Hypothesis: BETA=0			
Test	Chi-Square	DF	Pr > ChiSq
Likelihood Ratio	162.3289	8	<.0001
Score	185.2709	8	<.0001
Wald	145.3863	8	<.0001

Logistic Regression Results

The LOGISTIC Procedure

Analysis of Maximum Likelihood Estimates					
Parameter	DF	Estimate	Standard Error	Wald Chi-Square	Pr > ChiSq
Intercept	1	-6.0065	0.8738	47.2541	<.0001
E2	1	-0.0306	0.0319	0.9216	0.3371
age	1	0.00788	0.0146	0.2926	0.5886
BMI	1	0.2243	0.0203	122.2306	<.0001
SBP	1	-0.00561	0.00735	0.5826	0.4453
D1_al	1	0.2714	0.1831	2.1968	0.1383
D2_al	1	0.3844	0.2108	3.3265	0.0682
D1_sm	1	0.2431	0.3010	0.6524	0.4193
D2_sm	1	-0.0837	0.3051	0.0753	0.7838

Odds Ratio Estimates			
Effect	Point Estimate	95% Wald Confidence Limits	
E2	0.970	0.911	1.032
age	1.008	0.980	1.037
BMI	1.251	1.203	1.302
SBP	0.994	0.980	1.009
D1_al	1.312	0.916	1.878
D2_al	1.469	0.972	2.220
D1_sm	1.275	0.707	2.300
D2_sm	0.920	0.506	1.673

Association of Predicted Probabilities and Observed Responses			
Percent Concordant	72.1	Somers' D	0.447
Percent Discordant	27.3	Gamma	0.450
Percent Tied	0.6	Tau-a	0.127
Pairs	410310	c	0.724

Logistic Regression Results

The LOGISTIC Procedure

Model Information	
Data Set	WORK.SORTTEMPTABLESORTED
Response Variable	LDL_x
Number of Response Levels	2
Model	binary logit
Optimization Technique	Fisher's scoring

Number of Observations Read	1701
Number of Observations Used	1701

Response Profile		
Ordered Value	LDL_x	Total Frequency
1	0	1384
2	1	317

Probability modeled is LDL_x='1'.

Model Convergence Status
Convergence criterion (GCONV=1E-8) satisfied.

Model Fit Statistics		
Criterion	Intercept Only	Intercept and Covariates
AIC	1638.032	1543.485
SC	1643.471	1592.436
-2 Log L	1636.032	1525.485

Testing Global Null Hypothesis: BETA=0			
Test	Chi-Square	DF	Pr > ChiSq
Likelihood Ratio	110.5471	8	<.0001
Score	120.3618	8	<.0001
Wald	104.1363	8	<.0001

Logistic Regression Results

The LOGISTIC Procedure

Analysis of Maximum Likelihood Estimates					
Parameter	DF	Estimate	Standard Error	Wald Chi-Square	Pr > ChiSq
Intercept	1	-6.7844	0.8449	64.4743	<.0001
E2	1	-0.00797	0.0304	0.0689	0.7929
age	1	0.0837	0.0138	36.7640	<.0001
BMI	1	0.1343	0.0188	50.9109	<.0001
SBP	1	0.00226	0.00702	0.1038	0.7473
D1_al	1	0.0394	0.1819	0.0469	0.8286
D2_al	1	0.1620	0.2043	0.6290	0.4277
D1_sm	1	0.0951	0.2892	0.1082	0.7422
D2_sm	1	0.1828	0.2715	0.4534	0.5007

Odds Ratio Estimates			
Effect	Point Estimate	95% Wald Confidence Limits	
E2	0.992	0.935	1.053
age	1.087	1.058	1.117
BMI	1.144	1.102	1.187
SBP	1.002	0.989	1.016
D1_al	1.040	0.728	1.486
D2_al	1.176	0.788	1.755
D1_sm	1.100	0.624	1.939
D2_sm	1.201	0.705	2.044

Association of Predicted Probabilities and Observed Responses			
Percent Concordant	67.0	Somers' D	0.347
Percent Discordant	32.3	Gamma	0.350
Percent Tied	0.7	Tau-a	0.105
Pairs	438728	c	0.674

Logistic Regression Results

The LOGISTIC Procedure

Model Information	
Data Set	WORK.SORTTEMPTABLESORTED
Response Variable	TG_x
Number of Response Levels	2
Model	binary logit
Optimization Technique	Fisher's scoring

Number of Observations Read	1701
Number of Observations Used	1701

Response Profile		
Ordered Value	TG_x	Total Frequency
1	0	1614
2	1	87

Probability modeled is TG_x='1'.

Model Convergence Status
Convergence criterion (GCONV=1E-8) satisfied.

Model Fit Statistics		
Criterion	Intercept Only	Intercept and Covariates
AIC	688.785	589.233
SC	694.224	638.183
-2 Log L	686.785	571.233

Testing Global Null Hypothesis: BETA=0			
Test	Chi-Square	DF	Pr > ChiSq
Likelihood Ratio	115.5527	8	<.0001
Score	166.9618	8	<.0001
Wald	113.0006	8	<.0001

Logistic Regression Results

The LOGISTIC Procedure

Analysis of Maximum Likelihood Estimates					
Parameter	DF	Estimate	Standard Error	Wald Chi-Square	Pr > ChiSq
Intercept	1	-10.1455	1.4026	52.3217	<.0001
E2	1	0.00666	0.0548	0.0148	0.9033
age	1	0.0406	0.0236	2.9739	0.0846
BMI	1	0.2506	0.0289	75.1949	<.0001
SBP	1	0.00185	0.0117	0.0250	0.8745
D1_al	1	0.1058	0.3241	0.1065	0.7442
D2_al	1	0.4271	0.3414	1.5656	0.2108
D1_sm	1	-0.2001	0.6179	0.1049	0.7460
D2_sm	1	0.3360	0.4070	0.6814	0.4091

Odds Ratio Estimates			
Effect	Point Estimate	95% Wald Confidence Limits	
E2	1.007	0.904	1.121
age	1.041	0.994	1.091
BMI	1.285	1.214	1.360
SBP	1.002	0.979	1.025
D1_al	1.112	0.589	2.098
D2_al	1.533	0.785	2.993
D1_sm	0.819	0.244	2.748
D2_sm	1.399	0.630	3.107

Association of Predicted Probabilities and Observed Responses			
Percent Concordant	77.3	Somers' D	0.560
Percent Discordant	21.2	Gamma	0.569
Percent Tied	1.5	Tau-a	0.054
Pairs	140418	c	0.780

Logistic Regression Results

The LOGISTIC Procedure

Model Information	
Data Set	WORK.SORTTEMPTABLESORTED
Response Variable	TC_x
Number of Response Levels	2
Model	binary logit
Optimization Technique	Fisher's scoring

Number of Observations Read	1701
Number of Observations Used	1701

Response Profile		
Ordered Value	TC_x	Total Frequency
1	0	1386
2	1	315

Probability modeled is TC_x='1'.

Model Convergence Status
Convergence criterion (GCONV=1E-8) satisfied.

Model Fit Statistics		
Criterion	Intercept Only	Intercept and Covariates
AIC	1632.121	1583.190
SC	1637.560	1632.140
-2 Log L	1630.121	1565.190

Testing Global Null Hypothesis: BETA=0			
Test	Chi-Square	DF	Pr > ChiSq
Likelihood Ratio	64.9318	8	<.0001
Score	69.6009	8	<.0001
Wald	64.2997	8	<.0001

Logistic Regression Results

The LOGISTIC Procedure

Analysis of Maximum Likelihood Estimates					
Parameter	DF	Estimate	Standard Error	Wald Chi-Square	Pr > ChiSq
Intercept	1	-4.8228	0.8177	34.7882	<.0001
E3	1	0.0120	0.0270	0.1969	0.6572
age	1	0.0612	0.0136	20.2719	<.0001
BMI	1	0.1136	0.0187	37.0651	<.0001
SBP	1	-0.00834	0.00701	1.4162	0.2340
D1_al	1	0.1718	0.1742	0.9726	0.3240
D2_al	1	0.3048	0.1979	2.3726	0.1235
D1_sm	1	0.1589	0.2807	0.3206	0.5713
D2_sm	1	-0.1034	0.2858	0.1310	0.7174

Odds Ratio Estimates			
Effect	Point Estimate	95% Wald Confidence Limits	
E3	1.012	0.960	1.067
age	1.063	1.035	1.092
BMI	1.120	1.080	1.162
SBP	0.992	0.978	1.005
D1_al	1.187	0.844	1.671
D2_al	1.356	0.920	1.999
D1_sm	1.172	0.676	2.032
D2_sm	0.902	0.515	1.579

Association of Predicted Probabilities and Observed Responses			
Percent Concordant	62.2	Somers' D	0.252
Percent Discordant	36.9	Gamma	0.255
Percent Tied	0.9	Tau-a	0.076
Pairs	436590	c	0.626

Logistic Regression Results

The LOGISTIC Procedure

Model Information	
Data Set	WORK.SORTTEMPTABLESORTED
Response Variable	HDL_x
Number of Response Levels	2
Model	binary logit
Optimization Technique	Fisher's scoring

Number of Observations Read	1701
Number of Observations Used	1701

Response Profile		
Ordered Value	HDL_x	Total Frequency
1	0	1410
2	1	291

Probability modeled is HDL_x='1'.

Model Convergence Status
Convergence criterion (GCONV=1E-8) satisfied.

Model Fit Statistics		
Criterion	Intercept Only	Intercept and Covariates
AIC	1558.714	1413.160
SC	1564.153	1462.111
-2 Log L	1556.714	1395.160

Testing Global Null Hypothesis: BETA=0			
Test	Chi-Square	DF	Pr > ChiSq
Likelihood Ratio	161.5541	8	<.0001
Score	184.5401	8	<.0001
Wald	144.8483	8	<.0001

Logistic Regression Results

The LOGISTIC Procedure

Analysis of Maximum Likelihood Estimates					
Parameter	DF	Estimate	Standard Error	Wald Chi-Square	Pr > ChiSq
Intercept	1	-6.4311	0.8683	54.8536	<.0001
E3	1	0.0110	0.0287	0.1466	0.7018
age	1	0.00972	0.0145	0.4470	0.5037
BMI	1	0.2239	0.0203	121.7126	<.0001
SBP	1	-0.00568	0.00734	0.5993	0.4389
D1_al	1	0.2811	0.1830	2.3596	0.1245
D2_al	1	0.3921	0.2109	3.4560	0.0630
D1_sm	1	0.2378	0.3007	0.6255	0.4290
D2_sm	1	-0.0878	0.3046	0.0831	0.7731

Odds Ratio Estimates			
Effect	Point Estimate	95% Wald Confidence Limits	
E3	1.011	0.956	1.070
age	1.010	0.981	1.039
BMI	1.251	1.202	1.302
SBP	0.994	0.980	1.009
D1_al	1.325	0.925	1.896
D2_al	1.480	0.979	2.238
D1_sm	1.268	0.704	2.287
D2_sm	0.916	0.504	1.664

Association of Predicted Probabilities and Observed Responses			
Percent Concordant	72.1	Somers' D	0.449
Percent Discordant	27.2	Gamma	0.452
Percent Tied	0.6	Tau-a	0.127
Pairs	410310	c	0.724

Logistic Regression Results

The LOGISTIC Procedure

Model Information	
Data Set	WORK.SORTTEMPTABLESORTED
Response Variable	LDL_x
Number of Response Levels	2
Model	binary logit
Optimization Technique	Fisher's scoring

Number of Observations Read	1701
Number of Observations Used	1701

Response Profile		
Ordered Value	LDL_x	Total Frequency
1	0	1384
2	1	317

Probability modeled is LDL_x='1'.

Model Convergence Status
Convergence criterion (GCONV=1E-8) satisfied.

Model Fit Statistics		
Criterion	Intercept Only	Intercept and Covariates
AIC	1638.032	1543.295
SC	1643.471	1592.246
-2 Log L	1636.032	1525.295

Testing Global Null Hypothesis: BETA=0			
Test	Chi-Square	DF	Pr > ChiSq
Likelihood Ratio	110.7374	8	<.0001
Score	120.4882	8	<.0001
Wald	104.2326	8	<.0001

Logistic Regression Results

The LOGISTIC Procedure

Analysis of Maximum Likelihood Estimates					
Parameter	DF	Estimate	Standard Error	Wald Chi-Square	Pr > ChiSq
Intercept	1	-7.0074	0.8389	69.7730	<.0001
E3	1	0.0140	0.0275	0.2587	0.6110
age	1	0.0846	0.0138	37.6848	<.0001
BMI	1	0.1339	0.0188	50.4811	<.0001
SBP	1	0.00220	0.00702	0.0983	0.7539
D1_al	1	0.0453	0.1819	0.0621	0.8032
D2_al	1	0.1675	0.2043	0.6727	0.4121
D1_sm	1	0.0905	0.2891	0.0980	0.7543
D2_sm	1	0.1802	0.2715	0.4402	0.5070

Odds Ratio Estimates			
Effect	Point Estimate	95% Wald Confidence Limits	
E3	1.014	0.961	1.070
age	1.088	1.059	1.118
BMI	1.143	1.102	1.186
SBP	1.002	0.989	1.016
D1_al	1.046	0.733	1.495
D2_al	1.182	0.792	1.765
D1_sm	1.095	0.621	1.929
D2_sm	1.197	0.703	2.039

Association of Predicted Probabilities and Observed Responses			
Percent Concordant	67.1	Somers' D	0.348
Percent Discordant	32.2	Gamma	0.351
Percent Tied	0.7	Tau-a	0.106
Pairs	438728	c	0.674

Logistic Regression Results

The LOGISTIC Procedure

Model Information	
Data Set	WORK.SORTTEMPTABLESORTED
Response Variable	TG_x
Number of Response Levels	2
Model	binary logit
Optimization Technique	Fisher's scoring

Number of Observations Read	1701
Number of Observations Used	1701

Response Profile		
Ordered Value	TG_x	Total Frequency
1	0	1614
2	1	87

Probability modeled is TG_x='1'.

Model Convergence Status
Convergence criterion (GCONV=1E-8) satisfied.

Model Fit Statistics		
Criterion	Intercept Only	Intercept and Covariates
AIC	688.785	588.556
SC	694.224	637.506
-2 Log L	686.785	570.556

Testing Global Null Hypothesis: BETA=0			
Test	Chi-Square	DF	Pr > ChiSq
Likelihood Ratio	116.2297	8	<.0001
Score	167.7733	8	<.0001
Wald	113.7278	8	<.0001

Logistic Regression Results

The LOGISTIC Procedure

Analysis of Maximum Likelihood Estimates					
Parameter	DF	Estimate	Standard Error	Wald Chi-Square	Pr > ChiSq
Intercept	1	-9.6689	1.3587	50.6444	<.0001
E3	1	-0.0403	0.0483	0.6957	0.4042
age	1	0.0394	0.0234	2.8381	0.0921
BMI	1	0.2519	0.0290	75.6747	<.0001
SBP	1	0.00176	0.0117	0.0226	0.8804
D1_al	1	0.0852	0.3247	0.0689	0.7929
D2_al	1	0.4142	0.3417	1.4692	0.2255
D1_sm	1	-0.2038	0.6201	0.1080	0.7425
D2_sm	1	0.3378	0.4067	0.6898	0.4062

Odds Ratio Estimates			
Effect	Point Estimate	95% Wald Confidence Limits	
E3	0.961	0.874	1.056
age	1.040	0.994	1.089
BMI	1.286	1.215	1.362
SBP	1.002	0.979	1.025
D1_al	1.089	0.576	2.058
D2_al	1.513	0.774	2.956
D1_sm	0.816	0.242	2.750
D2_sm	1.402	0.632	3.111

Association of Predicted Probabilities and Observed Responses			
Percent Concordant	77.6	Somers' D	0.567
Percent Discordant	20.9	Gamma	0.576
Percent Tied	1.5	Tau-a	0.055
Pairs	140418	c	0.784

Logistic Regression Results

The LOGISTIC Procedure

Model Information	
Data Set	WORK.SORTTEMPTABLESORTED
Response Variable	TC_x
Number of Response Levels	2
Model	binary logit
Optimization Technique	Fisher's scoring

Number of Observations Read	1701
Number of Observations Used	1701

Response Profile		
Ordered Value	TC_x	Total Frequency
1	0	1386
2	1	315

Probability modeled is TC_x='1'.

Model Convergence Status
Convergence criterion (GCONV=1E-8) satisfied.

Model Fit Statistics		
Criterion	Intercept Only	Intercept and Covariates
AIC	1632.121	1582.573
SC	1637.560	1631.524
-2 Log L	1630.121	1564.573

Testing Global Null Hypothesis: BETA=0			
Test	Chi-Square	DF	Pr > ChiSq
Likelihood Ratio	65.5484	8	<.0001
Score	70.3035	8	<.0001
Wald	64.9324	8	<.0001

Logistic Regression Results

The LOGISTIC Procedure

Analysis of Maximum Likelihood Estimates					
Parameter	DF	Estimate	Standard Error	Wald Chi-Square	Pr > ChiSq
Intercept	1	-4.4323	0.8232	28.9881	<.0001
E4	1	-0.0253	0.0281	0.8130	0.3672
age	1	0.0603	0.0136	19.8065	<.0001
BMI	1	0.1136	0.0186	37.2264	<.0001
SBP	1	-0.00846	0.00701	1.4554	0.2277
D1_al	1	0.1673	0.1741	0.9232	0.3366
D2_al	1	0.2977	0.1978	2.2653	0.1323
D1_sm	1	0.1557	0.2811	0.3066	0.5798
D2_sm	1	-0.0935	0.2858	0.1071	0.7435

Odds Ratio Estimates			
Effect	Point Estimate	95% Wald Confidence Limits	
E4	0.975	0.923	1.030
age	1.062	1.034	1.091
BMI	1.120	1.080	1.162
SBP	0.992	0.978	1.005
D1_al	1.182	0.840	1.663
D2_al	1.347	0.914	1.985
D1_sm	1.168	0.673	2.027
D2_sm	0.911	0.520	1.595

Association of Predicted Probabilities and Observed Responses			
Percent Concordant	62.1	Somers' D	0.252
Percent Discordant	37.0	Gamma	0.254
Percent Tied	0.9	Tau-a	0.076
Pairs	436590	c	0.626

Logistic Regression Results

The LOGISTIC Procedure

Model Information	
Data Set	WORK.SORTTEMPTABLESORTED
Response Variable	HDL_x
Number of Response Levels	2
Model	binary logit
Optimization Technique	Fisher's scoring

Number of Observations Read	1701
Number of Observations Used	1701

Response Profile		
Ordered Value	HDL_x	Total Frequency
1	0	1410
2	1	291

Probability modeled is HDL_x='1'.

Model Convergence Status
Convergence criterion (GCONV=1E-8) satisfied.

Model Fit Statistics		
Criterion	Intercept Only	Intercept and Covariates
AIC	1558.714	1412.061
SC	1564.153	1461.012
-2 Log L	1556.714	1394.061

Testing Global Null Hypothesis: BETA=0			
Test	Chi-Square	DF	Pr > ChiSq
Likelihood Ratio	162.6532	8	<.0001
Score	186.1575	8	<.0001
Wald	145.6170	8	<.0001

Logistic Regression Results

The LOGISTIC Procedure

Analysis of Maximum Likelihood Estimates					
Parameter	DF	Estimate	Standard Error	Wald Chi-Square	Pr > ChiSq
Intercept	1	-5.9550	0.8727	46.5579	<.0001
E4	1	-0.0335	0.0300	1.2442	0.2647
age	1	0.00879	0.0145	0.3673	0.5445
BMI	1	0.2237	0.0203	121.5220	<.0001
SBP	1	-0.00591	0.00735	0.6472	0.4211
D1_al	1	0.2759	0.1829	2.2744	0.1315
D2_al	1	0.3838	0.2109	3.3108	0.0688
D1_sm	1	0.2316	0.3016	0.5900	0.4424
D2_sm	1	-0.0780	0.3050	0.0655	0.7981

Odds Ratio Estimates			
Effect	Point Estimate	95% Wald Confidence Limits	
E4	0.967	0.912	1.026
age	1.009	0.981	1.038
BMI	1.251	1.202	1.301
SBP	0.994	0.980	1.009
D1_al	1.318	0.921	1.886
D2_al	1.468	0.971	2.219
D1_sm	1.261	0.698	2.277
D2_sm	0.925	0.509	1.682

Association of Predicted Probabilities and Observed Responses			
Percent Concordant	72.0	Somers' D	0.446
Percent Discordant	27.4	Gamma	0.449
Percent Tied	0.6	Tau-a	0.127
Pairs	410310	c	0.723

Logistic Regression Results

The LOGISTIC Procedure

Model Information	
Data Set	WORK.SORTTEMPTABLESORTED
Response Variable	LDL_x
Number of Response Levels	2
Model	binary logit
Optimization Technique	Fisher's scoring

Number of Observations Read	1701
Number of Observations Used	1701

Response Profile		
Ordered Value	LDL_x	Total Frequency
1	0	1384
2	1	317

Probability modeled is LDL_x='1'.

Model Convergence Status
Convergence criterion (GCONV=1E-8) satisfied.

Model Fit Statistics		
Criterion	Intercept Only	Intercept and Covariates
AIC	1638.032	1542.469
SC	1643.471	1591.420
-2 Log L	1636.032	1524.469

Testing Global Null Hypothesis: BETA=0			
Test	Chi-Square	DF	Pr > ChiSq
Likelihood Ratio	111.5631	8	<.0001
Score	121.4631	8	<.0001
Wald	105.0523	8	<.0001

Logistic Regression Results

The LOGISTIC Procedure

Analysis of Maximum Likelihood Estimates					
Parameter	DF	Estimate	Standard Error	Wald Chi-Square	Pr > ChiSq
Intercept	1	-6.5479	0.8426	60.3939	<.0001
E4	1	-0.0297	0.0285	1.0835	0.2979
age	1	0.0836	0.0137	37.0517	<.0001
BMI	1	0.1339	0.0188	50.5895	<.0001
SBP	1	0.00206	0.00702	0.0858	0.7696
D1_al	1	0.0391	0.1817	0.0464	0.8294
D2_al	1	0.1592	0.2042	0.6078	0.4356
D1_sm	1	0.0861	0.2898	0.0884	0.7663
D2_sm	1	0.1929	0.2714	0.5049	0.4774

Odds Ratio Estimates			
Effect	Point Estimate	95% Wald Confidence Limits	
E4	0.971	0.918	1.027
age	1.087	1.058	1.117
BMI	1.143	1.102	1.186
SBP	1.002	0.988	1.016
D1_al	1.040	0.728	1.485
D2_al	1.173	0.786	1.750
D1_sm	1.090	0.618	1.923
D2_sm	1.213	0.712	2.065

Association of Predicted Probabilities and Observed Responses			
Percent Concordant	67.1	Somers' D	0.349
Percent Discordant	32.2	Gamma	0.351
Percent Tied	0.7	Tau-a	0.106
Pairs	438728	c	0.675

Logistic Regression Results

The LOGISTIC Procedure

Model Information	
Data Set	WORK.SORTTEMPTABLESORTED
Response Variable	TG_x
Number of Response Levels	2
Model	binary logit
Optimization Technique	Fisher's scoring

Number of Observations Read	1701
Number of Observations Used	1701

Response Profile		
Ordered Value	TG_x	Total Frequency
1	0	1614
2	1	87

Probability modeled is TG_x='1'.

Model Convergence Status
Convergence criterion (GCONV=1E-8) satisfied.

Model Fit Statistics		
Criterion	Intercept Only	Intercept and Covariates
AIC	688.785	587.279
SC	694.224	636.230
-2 Log L	686.785	569.279

Testing Global Null Hypothesis: BETA=0			
Test	Chi-Square	DF	Pr > ChiSq
Likelihood Ratio	117.5059	8	<.0001
Score	169.9939	8	<.0001
Wald	114.3154	8	<.0001

Logistic Regression Results

The LOGISTIC Procedure

Analysis of Maximum Likelihood Estimates					
Parameter	DF	Estimate	Standard Error	Wald Chi-Square	Pr > ChiSq
Intercept	1	-9.2874	1.3827	45.1160	<.0001
E4	1	-0.0719	0.0514	1.9540	0.1622
age	1	0.0404	0.0234	2.9880	0.0839
BMI	1	0.2484	0.0290	73.5880	<.0001
SBP	1	0.00115	0.0117	0.0096	0.9218
D1_al	1	0.1058	0.3239	0.1068	0.7438
D2_al	1	0.4169	0.3408	1.4967	0.2212
D1_sm	1	-0.2438	0.6216	0.1538	0.6949
D2_sm	1	0.3631	0.4083	0.7908	0.3739

Odds Ratio Estimates			
Effect	Point Estimate	95% Wald Confidence Limits	
E4	0.931	0.841	1.029
age	1.041	0.995	1.090
BMI	1.282	1.211	1.357
SBP	1.001	0.978	1.024
D1_al	1.112	0.589	2.097
D2_al	1.517	0.778	2.959
D1_sm	0.784	0.232	2.650
D2_sm	1.438	0.646	3.201

Association of Predicted Probabilities and Observed Responses			
Percent Concordant	77.6	Somers' D	0.568
Percent Discordant	20.8	Gamma	0.577
Percent Tied	1.6	Tau-a	0.055
Pairs	140418	c	0.784

Logistic Regression Results

The LOGISTIC Procedure

Model Information	
Data Set	WORK.SORTTEMPTABLESORTED
Response Variable	TC_x
Number of Response Levels	2
Model	binary logit
Optimization Technique	Fisher's scoring

Number of Observations Read	1701
Number of Observations Used	1701

Response Profile		
Ordered Value	TC_x	Total Frequency
1	0	1386
2	1	315

Probability modeled is TC_x='1'.

Model Convergence Status
Convergence criterion (GCONV=1E-8) satisfied.

Model Fit Statistics		
Criterion	Intercept Only	Intercept and Covariates
AIC	1632.121	1583.365
SC	1637.560	1632.316
-2 Log L	1630.121	1565.365

Testing Global Null Hypothesis: BETA=0			
Test	Chi-Square	DF	Pr > ChiSq
Likelihood Ratio	64.7561	8	<.0001
Score	69.4635	8	<.0001
Wald	64.1840	8	<.0001

Logistic Regression Results

The LOGISTIC Procedure

Analysis of Maximum Likelihood Estimates					
Parameter	DF	Estimate	Standard Error	Wald Chi-Square	Pr > ChiSq
Intercept	1	-4.7458	0.8281	32.8452	<.0001
E5	1	0.00394	0.0269	0.0215	0.8833
age	1	0.0609	0.0136	20.1091	<.0001
BMI	1	0.1140	0.0186	37.4403	<.0001
SBP	1	-0.00834	0.00701	1.4151	0.2342
D1_al	1	0.1700	0.1742	0.9516	0.3293
D2_al	1	0.3027	0.1979	2.3398	0.1261
D1_sm	1	0.1624	0.2807	0.3349	0.5628
D2_sm	1	-0.1036	0.2859	0.1313	0.7171

Odds Ratio Estimates			
Effect	Point Estimate	95% Wald Confidence Limits	
E5	1.004	0.952	1.058
age	1.063	1.035	1.091
BMI	1.121	1.081	1.162
SBP	0.992	0.978	1.005
D1_al	1.185	0.842	1.668
D2_al	1.353	0.918	1.995
D1_sm	1.176	0.679	2.039
D2_sm	0.902	0.515	1.579

Association of Predicted Probabilities and Observed Responses			
Percent Concordant	62.0	Somers' D	0.250
Percent Discordant	37.1	Gamma	0.252
Percent Tied	0.9	Tau-a	0.075
Pairs	436590	c	0.625

Logistic Regression Results

The LOGISTIC Procedure

Model Information	
Data Set	WORK.SORTTEMPTABLESORTED
Response Variable	HDL_x
Number of Response Levels	2
Model	binary logit
Optimization Technique	Fisher's scoring

Number of Observations Read	1701
Number of Observations Used	1701

Response Profile		
Ordered Value	HDL_x	Total Frequency
1	0	1410
2	1	291

Probability modeled is HDL_x='1'.

Model Convergence Status
Convergence criterion (GCONV=1E-8) satisfied.

Model Fit Statistics		
Criterion	Intercept Only	Intercept and Covariates
AIC	1558.714	1413.306
SC	1564.153	1462.256
-2 Log L	1556.714	1395.306

Testing Global Null Hypothesis: BETA=0			
Test	Chi-Square	DF	Pr > ChiSq
Likelihood Ratio	161.4088	8	<.0001
Score	184.4891	8	<.0001
Wald	144.8538	8	<.0001

Logistic Regression Results

The LOGISTIC Procedure

Analysis of Maximum Likelihood Estimates					
Parameter	DF	Estimate	Standard Error	Wald Chi-Square	Pr > ChiSq
Intercept	1	-6.3030	0.8751	51.8726	<.0001
E5	1	-0.00114	0.0285	0.0016	0.9680
age	1	0.00930	0.0145	0.4105	0.5217
BMI	1	0.2241	0.0203	122.1629	<.0001
SBP	1	-0.00569	0.00734	0.6007	0.4383
D1_al	1	0.2777	0.1829	2.3047	0.1290
D2_al	1	0.3891	0.2109	3.4042	0.0650
D1_sm	1	0.2414	0.3007	0.6443	0.4222
D2_sm	1	-0.0871	0.3049	0.0816	0.7752

Odds Ratio Estimates			
Effect	Point Estimate	95% Wald Confidence Limits	
E5	0.999	0.945	1.056
age	1.009	0.981	1.038
BMI	1.251	1.202	1.302
SBP	0.994	0.980	1.009
D1_al	1.320	0.922	1.889
D2_al	1.476	0.976	2.231
D1_sm	1.273	0.706	2.295
D2_sm	0.917	0.504	1.666

Association of Predicted Probabilities and Observed Responses			
Percent Concordant	72.1	Somers' D	0.449
Percent Discordant	27.2	Gamma	0.452
Percent Tied	0.6	Tau-a	0.127
Pairs	410310	c	0.724

Logistic Regression Results

The LOGISTIC Procedure

Model Information	
Data Set	WORK.SORTTEMPTABLESORTED
Response Variable	LDL_x
Number of Response Levels	2
Model	binary logit
Optimization Technique	Fisher's scoring

Number of Observations Read	1701
Number of Observations Used	1701

Response Profile		
Ordered Value	LDL_x	Total Frequency
1	0	1384
2	1	317

Probability modeled is LDL_x='1'.

Model Convergence Status
Convergence criterion (GCONV=1E-8) satisfied.

Model Fit Statistics		
Criterion	Intercept Only	Intercept and Covariates
AIC	1638.032	1541.993
SC	1643.471	1590.944
-2 Log L	1636.032	1523.993

Testing Global Null Hypothesis: BETA=0			
Test	Chi-Square	DF	Pr > ChiSq
Likelihood Ratio	112.0390	8	<.0001
Score	121.6229	8	<.0001
Wald	105.2760	8	<.0001

Logistic Regression Results

The LOGISTIC Procedure

Analysis of Maximum Likelihood Estimates					
Parameter	DF	Estimate	Standard Error	Wald Chi-Square	Pr > ChiSq
Intercept	1	-6.4884	0.8427	59.2877	<.0001
E5	1	-0.0339	0.0271	1.5614	0.2115
age	1	0.0831	0.0137	36.5389	<.0001
BMI	1	0.1342	0.0188	50.8192	<.0001
SBP	1	0.00229	0.00702	0.1066	0.7440
D1_al	1	0.0328	0.1819	0.0325	0.8569
D2_al	1	0.1554	0.2043	0.5781	0.4470
D1_sm	1	0.0981	0.2896	0.1147	0.7349
D2_sm	1	0.1973	0.2720	0.5262	0.4682

Odds Ratio Estimates			
Effect	Point Estimate	95% Wald Confidence Limits	
E5	0.967	0.917	1.019
age	1.087	1.058	1.116
BMI	1.144	1.102	1.187
SBP	1.002	0.989	1.016
D1_al	1.033	0.723	1.476
D2_al	1.168	0.783	1.743
D1_sm	1.103	0.625	1.946
D2_sm	1.218	0.715	2.076

Association of Predicted Probabilities and Observed Responses			
Percent Concordant	67.3	Somers' D	0.352
Percent Discordant	32.1	Gamma	0.354
Percent Tied	0.7	Tau-a	0.107
Pairs	438728	c	0.676

Logistic Regression Results

The LOGISTIC Procedure

Model Information	
Data Set	WORK.SORTTEMPTABLESORTED
Response Variable	TG_x
Number of Response Levels	2
Model	binary logit
Optimization Technique	Fisher's scoring

Number of Observations Read	1701
Number of Observations Used	1701

Response Profile		
Ordered Value	TG_x	Total Frequency
1	0	1614
2	1	87

Probability modeled is TG_x='1'.

Model Convergence Status
Convergence criterion (GCONV=1E-8) satisfied.

Model Fit Statistics		
Criterion	Intercept Only	Intercept and Covariates
AIC	688.785	589.242
SC	694.224	638.193
-2 Log L	686.785	571.242

Testing Global Null Hypothesis: BETA=0			
Test	Chi-Square	DF	Pr > ChiSq
Likelihood Ratio	115.5434	8	<.0001
Score	167.0385	8	<.0001
Wald	112.9825	8	<.0001

Logistic Regression Results

The LOGISTIC Procedure

Analysis of Maximum Likelihood Estimates					
Parameter	DF	Estimate	Standard Error	Wald Chi-Square	Pr > ChiSq
Intercept	1	-10.1117	1.3689	54.5620	<.0001
E5	1	0.00352	0.0476	0.0055	0.9411
age	1	0.0404	0.0234	2.9723	0.0847
BMI	1	0.2506	0.0289	75.1913	<.0001
SBP	1	0.00183	0.0117	0.0244	0.8759
D1_al	1	0.1044	0.3238	0.1039	0.7472
D2_al	1	0.4266	0.3413	1.5621	0.2114
D1_sm	1	-0.2008	0.6179	0.1056	0.7452
D2_sm	1	0.3348	0.4072	0.6760	0.4110

Odds Ratio Estimates			
Effect	Point Estimate	95% Wald Confidence Limits	
E5	1.004	0.914	1.102
age	1.041	0.994	1.090
BMI	1.285	1.214	1.360
SBP	1.002	0.979	1.025
D1_al	1.110	0.588	2.094
D2_al	1.532	0.785	2.991
D1_sm	0.818	0.244	2.746
D2_sm	1.398	0.629	3.105

Association of Predicted Probabilities and Observed Responses			
Percent Concordant	77.3	Somers' D	0.561
Percent Discordant	21.2	Gamma	0.570
Percent Tied	1.5	Tau-a	0.054
Pairs	140418	c	0.781

Logistic Regression Results

The LOGISTIC Procedure

Model Information	
Data Set	WORK.SORTTEMPTABLESORTED
Response Variable	TC_x
Number of Response Levels	2
Model	binary logit
Optimization Technique	Fisher's scoring

Number of Observations Read	1701
Number of Observations Used	1701

Response Profile		
Ordered Value	TC_x	Total Frequency
1	0	1386
2	1	315

Probability modeled is TC_x='1'.

Model Convergence Status
Convergence criterion (GCONV=1E-8) satisfied.

Model Fit Statistics		
Criterion	Intercept Only	Intercept and Covariates
AIC	1632.121	1582.776
SC	1637.560	1631.727
-2 Log L	1630.121	1564.776

Testing Global Null Hypothesis: BETA=0			
Test	Chi-Square	DF	Pr > ChiSq
Likelihood Ratio	65.3455	8	<.0001
Score	69.9677	8	<.0001
Wald	64.7626	8	<.0001

Logistic Regression Results

The LOGISTIC Procedure

Analysis of Maximum Likelihood Estimates					
Parameter	DF	Estimate	Standard Error	Wald Chi-Square	Pr > ChiSq
Intercept	1	-4.4577	0.8282	28.9689	<.0001
E6	1	-0.0219	0.0280	0.6123	0.4339
age	1	0.0598	0.0136	19.3002	<.0001
BMI	1	0.1140	0.0186	37.5475	<.0001
SBP	1	-0.00830	0.00700	1.4037	0.2361
D1_al	1	0.1739	0.1742	0.9963	0.3182
D2_al	1	0.2980	0.1977	2.2716	0.1318
D1_sm	1	0.1559	0.2811	0.3074	0.5793
D2_sm	1	-0.0942	0.2855	0.1089	0.7414

Odds Ratio Estimates			
Effect	Point Estimate	95% Wald Confidence Limits	
E6	0.978	0.926	1.034
age	1.062	1.034	1.090
BMI	1.121	1.081	1.162
SBP	0.992	0.978	1.005
D1_al	1.190	0.846	1.674
D2_al	1.347	0.914	1.985
D1_sm	1.169	0.674	2.028
D2_sm	0.910	0.520	1.593

Association of Predicted Probabilities and Observed Responses			
Percent Concordant	62.1	Somers' D	0.250
Percent Discordant	37.0	Gamma	0.252
Percent Tied	0.9	Tau-a	0.076
Pairs	436590	c	0.625

Logistic Regression Results

The LOGISTIC Procedure

Model Information	
Data Set	WORK.SORTTEMPTABLESORTED
Response Variable	HDL_x
Number of Response Levels	2
Model	binary logit
Optimization Technique	Fisher's scoring

Number of Observations Read	1701
Number of Observations Used	1701

Response Profile		
Ordered Value	HDL_x	Total Frequency
1	0	1410
2	1	291

Probability modeled is HDL_x='1'.

Model Convergence Status
Convergence criterion (GCONV=1E-8) satisfied.

Model Fit Statistics		
Criterion	Intercept Only	Intercept and Covariates
AIC	1558.714	1412.241
SC	1564.153	1461.192
-2 Log L	1556.714	1394.241

Testing Global Null Hypothesis: BETA=0			
Test	Chi-Square	DF	Pr > ChiSq
Likelihood Ratio	162.4734	8	<.0001
Score	185.4665	8	<.0001
Wald	145.9512	8	<.0001

Logistic Regression Results

The LOGISTIC Procedure

Analysis of Maximum Likelihood Estimates					
Parameter	DF	Estimate	Standard Error	Wald Chi-Square	Pr > ChiSq
Intercept	1	-5.9678	0.8777	46.2293	<.0001
E6	1	-0.0308	0.0298	1.0687	0.3012
age	1	0.00797	0.0146	0.2994	0.5842
BMI	1	0.2241	0.0203	122.3856	<.0001
SBP	1	-0.00568	0.00734	0.5979	0.4394
D1_al	1	0.2849	0.1831	2.4220	0.1196
D2_al	1	0.3854	0.2105	3.3518	0.0671
D1_sm	1	0.2308	0.3013	0.5871	0.4436
D2_sm	1	-0.0736	0.3045	0.0584	0.8091

Odds Ratio Estimates			
Effect	Point Estimate	95% Wald Confidence Limits	
E6	0.970	0.915	1.028
age	1.008	0.980	1.037
BMI	1.251	1.202	1.302
SBP	0.994	0.980	1.009
D1_al	1.330	0.929	1.904
D2_al	1.470	0.973	2.221
D1_sm	1.260	0.698	2.273
D2_sm	0.929	0.512	1.687

Association of Predicted Probabilities and Observed Responses			
Percent Concordant	72.1	Somers' D	0.449
Percent Discordant	27.3	Gamma	0.451
Percent Tied	0.6	Tau-a	0.127
Pairs	410310	c	0.724

Logistic Regression Results

The LOGISTIC Procedure

Model Information	
Data Set	WORK.SORTTEMPTABLESORTED
Response Variable	LDL_x
Number of Response Levels	2
Model	binary logit
Optimization Technique	Fisher's scoring

Number of Observations Read	1701
Number of Observations Used	1701

Response Profile		
Ordered Value	LDL_x	Total Frequency
1	0	1384
2	1	317

Probability modeled is LDL_x='1'.

Model Convergence Status
Convergence criterion (GCONV=1E-8) satisfied.

Model Fit Statistics		
Criterion	Intercept Only	Intercept and Covariates
AIC	1638.032	1543.123
SC	1643.471	1592.074
-2 Log L	1636.032	1525.123

Testing Global Null Hypothesis: BETA=0			
Test	Chi-Square	DF	Pr > ChiSq
Likelihood Ratio	110.9090	8	<.0001
Score	120.6198	8	<.0001
Wald	104.5988	8	<.0001

Logistic Regression Results

The LOGISTIC Procedure

Analysis of Maximum Likelihood Estimates					
Parameter	DF	Estimate	Standard Error	Wald Chi-Square	Pr > ChiSq
Intercept	1	-6.6541	0.8495	61.3605	<.0001
E6	1	-0.0187	0.0285	0.4316	0.5112
age	1	0.0832	0.0138	36.4387	<.0001
BMI	1	0.1343	0.0188	51.0229	<.0001
SBP	1	0.00223	0.00701	0.1015	0.7500
D1_al	1	0.0461	0.1818	0.0643	0.7998
D2_al	1	0.1610	0.2041	0.6227	0.4301
D1_sm	1	0.0890	0.2896	0.0944	0.7587
D2_sm	1	0.1882	0.2712	0.4817	0.4877

Odds Ratio Estimates			
Effect	Point Estimate	95% Wald Confidence Limits	
E6	0.981	0.928	1.038
age	1.087	1.058	1.116
BMI	1.144	1.102	1.187
SBP	1.002	0.989	1.016
D1_al	1.047	0.733	1.495
D2_al	1.175	0.787	1.753
D1_sm	1.093	0.620	1.928
D2_sm	1.207	0.709	2.054

Association of Predicted Probabilities and Observed Responses			
Percent Concordant	67.1	Somers' D	0.349
Percent Discordant	32.2	Gamma	0.351
Percent Tied	0.7	Tau-a	0.106
Pairs	438728	c	0.674

Logistic Regression Results

The LOGISTIC Procedure

Model Information	
Data Set	WORK.SORTTEMPTABLESORTED
Response Variable	TG_x
Number of Response Levels	2
Model	binary logit
Optimization Technique	Fisher's scoring

Number of Observations Read	1701
Number of Observations Used	1701

Response Profile		
Ordered Value	TG_x	Total Frequency
1	0	1614
2	1	87

Probability modeled is TG_x='1'.

Model Convergence Status
Convergence criterion (GCONV=1E-8) satisfied.

Model Fit Statistics		
Criterion	Intercept Only	Intercept and Covariates
AIC	688.785	588.649
SC	694.224	637.599
-2 Log L	686.785	570.649

Testing Global Null Hypothesis: BETA=0			
Test	Chi-Square	DF	Pr > ChiSq
Likelihood Ratio	116.1368	8	<.0001
Score	167.1699	8	<.0001
Wald	113.4659	8	<.0001

Logistic Regression Results

The LOGISTIC Procedure

Analysis of Maximum Likelihood Estimates					
Parameter	DF	Estimate	Standard Error	Wald Chi-Square	Pr > ChiSq
Intercept	1	-9.6572	1.3798	48.9892	<.0001
E6	1	-0.0395	0.0509	0.6019	0.4378
age	1	0.0390	0.0234	2.7676	0.0962
BMI	1	0.2509	0.0289	75.5525	<.0001
SBP	1	0.00190	0.0117	0.0265	0.8707
D1_al	1	0.1164	0.3243	0.1289	0.7196
D2_al	1	0.4322	0.3402	1.6139	0.2040
D1_sm	1	-0.2179	0.6187	0.1240	0.7247
D2_sm	1	0.3557	0.4080	0.7601	0.3833

Odds Ratio Estimates			
Effect	Point Estimate	95% Wald Confidence Limits	
E6	0.961	0.870	1.062
age	1.040	0.993	1.089
BMI	1.285	1.214	1.360
SBP	1.002	0.979	1.025
D1_al	1.123	0.595	2.121
D2_al	1.541	0.791	3.001
D1_sm	0.804	0.239	2.704
D2_sm	1.427	0.642	3.175

Association of Predicted Probabilities and Observed Responses			
Percent Concordant	77.7	Somers' D	0.568
Percent Discordant	20.8	Gamma	0.577
Percent Tied	1.5	Tau-a	0.055
Pairs	140418	c	0.784

Logistic Regression Results

The LOGISTIC Procedure

Model Information	
Data Set	WORK.SORTTEMPTABLESORTED
Response Variable	TC_x
Number of Response Levels	2
Model	binary logit
Optimization Technique	Fisher's scoring

Number of Observations Read	1701
Number of Observations Used	1701

Response Profile		
Ordered Value	TC_x	Total Frequency
1	0	1386
2	1	315

Probability modeled is TC_x='1'.

Model Convergence Status
Convergence criterion (GCONV=1E-8) satisfied.

Model Fit Statistics		
Criterion	Intercept Only	Intercept and Covariates
AIC	1632.121	1582.879
SC	1637.560	1631.829
-2 Log L	1630.121	1564.879

Testing Global Null Hypothesis: BETA=0			
Test	Chi-Square	DF	Pr > ChiSq
Likelihood Ratio	65.2428	8	<.0001
Score	69.9386	8	<.0001
Wald	64.5381	8	<.0001

Logistic Regression Results

The LOGISTIC Procedure

Analysis of Maximum Likelihood Estimates					
Parameter	DF	Estimate	Standard Error	Wald Chi-Square	Pr > ChiSq
Intercept	1	-4.9110	0.8249	35.4462	<.0001
O1	1	0.0186	0.0262	0.5060	0.4769
age	1	0.0617	0.0136	20.4812	<.0001
BMI	1	0.1134	0.0187	36.9446	<.0001
SBP	1	-0.00839	0.00700	1.4347	0.2310
D1_al	1	0.1715	0.1742	0.9696	0.3248
D2_al	1	0.3077	0.1979	2.4174	0.1200
D1_sm	1	0.1650	0.2807	0.3455	0.5567
D2_sm	1	-0.1145	0.2865	0.1599	0.6893

Odds Ratio Estimates			
Effect	Point Estimate	95% Wald Confidence Limits	
O1	1.019	0.968	1.072
age	1.064	1.036	1.092
BMI	1.120	1.080	1.162
SBP	0.992	0.978	1.005
D1_al	1.187	0.844	1.670
D2_al	1.360	0.923	2.005
D1_sm	1.179	0.680	2.045
D2_sm	0.892	0.509	1.564

Association of Predicted Probabilities and Observed Responses			
Percent Concordant	62.1	Somers' D	0.251
Percent Discordant	37.0	Gamma	0.253
Percent Tied	0.9	Tau-a	0.076
Pairs	436590	c	0.625

Logistic Regression Results

The LOGISTIC Procedure

Model Information	
Data Set	WORK.SORTTEMPTABLESORTED
Response Variable	HDL_x
Number of Response Levels	2
Model	binary logit
Optimization Technique	Fisher's scoring

Number of Observations Read	1701
Number of Observations Used	1701

Response Profile		
Ordered Value	HDL_x	Total Frequency
1	0	1410
2	1	291

Probability modeled is HDL_x='1'.

Model Convergence Status
Convergence criterion (GCONV=1E-8) satisfied.

Model Fit Statistics		
Criterion	Intercept Only	Intercept and Covariates
AIC	1558.714	1413.290
SC	1564.153	1462.241
-2 Log L	1556.714	1395.290

Testing Global Null Hypothesis: BETA=0			
Test	Chi-Square	DF	Pr > ChiSq
Likelihood Ratio	161.4244	8	<.0001
Score	184.4950	8	<.0001
Wald	144.8859	8	<.0001

Logistic Regression Results

The LOGISTIC Procedure

Analysis of Maximum Likelihood Estimates					
Parameter	DF	Estimate	Standard Error	Wald Chi-Square	Pr > ChiSq
Intercept	1	-6.2755	0.8697	52.0655	<.0001
O1	1	-0.00365	0.0278	0.0172	0.8956
age	1	0.00918	0.0145	0.3982	0.5280
BMI	1	0.2243	0.0203	122.0823	<.0001
SBP	1	-0.00568	0.00734	0.5984	0.4392
D1_al	1	0.2774	0.1829	2.3006	0.1293
D2_al	1	0.3880	0.2110	3.3817	0.0659
D1_sm	1	0.2404	0.3008	0.6390	0.4241
D2_sm	1	-0.0855	0.3050	0.0785	0.7793

Odds Ratio Estimates			
Effect	Point Estimate	95% Wald Confidence Limits	
O1	0.996	0.943	1.052
age	1.009	0.981	1.038
BMI	1.251	1.203	1.302
SBP	0.994	0.980	1.009
D1_al	1.320	0.922	1.889
D2_al	1.474	0.975	2.229
D1_sm	1.272	0.705	2.293
D2_sm	0.918	0.505	1.669

Association of Predicted Probabilities and Observed Responses			
Percent Concordant	72.1	Somers' D	0.449
Percent Discordant	27.2	Gamma	0.452
Percent Tied	0.6	Tau-a	0.127
Pairs	410310	c	0.724

Logistic Regression Results

The LOGISTIC Procedure

Model Information	
Data Set	WORK.SORTTEMPTABLESORTED
Response Variable	LDL_x
Number of Response Levels	2
Model	binary logit
Optimization Technique	Fisher's scoring

Number of Observations Read	1701
Number of Observations Used	1701

Response Profile		
Ordered Value	LDL_x	Total Frequency
1	0	1384
2	1	317

Probability modeled is LDL_x='1'.

Model Convergence Status
Convergence criterion (GCONV=1E-8) satisfied.

Model Fit Statistics		
Criterion	Intercept Only	Intercept and Covariates
AIC	1638.032	1543.367
SC	1643.471	1592.318
-2 Log L	1636.032	1525.367

Testing Global Null Hypothesis: BETA=0			
Test	Chi-Square	DF	Pr > ChiSq
Likelihood Ratio	110.6649	8	<.0001
Score	120.4884	8	<.0001
Wald	104.2071	8	<.0001

Logistic Regression Results

The LOGISTIC Procedure

Analysis of Maximum Likelihood Estimates					
Parameter	DF	Estimate	Standard Error	Wald Chi-Square	Pr > ChiSq
Intercept	1	-6.9918	0.8434	68.7289	<.0001
O1	1	0.0115	0.0266	0.1862	0.6661
age	1	0.0846	0.0138	37.5759	<.0001
BMI	1	0.1339	0.0188	50.5333	<.0001
SBP	1	0.00216	0.00702	0.0950	0.7580
D1_al	1	0.0438	0.1818	0.0580	0.8096
D2_al	1	0.1681	0.2043	0.6764	0.4108
D1_sm	1	0.0970	0.2892	0.1124	0.7374
D2_sm	1	0.1741	0.2720	0.4095	0.5222

Odds Ratio Estimates			
Effect	Point Estimate	95% Wald Confidence Limits	
O1	1.012	0.960	1.066
age	1.088	1.059	1.118
BMI	1.143	1.102	1.186
SBP	1.002	0.988	1.016
D1_al	1.045	0.732	1.492
D2_al	1.183	0.793	1.766
D1_sm	1.102	0.625	1.942
D2_sm	1.190	0.698	2.028

Association of Predicted Probabilities and Observed Responses			
Percent Concordant	67.1	Somers' D	0.348
Percent Discordant	32.2	Gamma	0.351
Percent Tied	0.7	Tau-a	0.106
Pairs	438728	c	0.674

Logistic Regression Results

The LOGISTIC Procedure

Model Information	
Data Set	WORK.SORTTEMPTABLESORTED
Response Variable	TG_x
Number of Response Levels	2
Model	binary logit
Optimization Technique	Fisher's scoring

Number of Observations Read	1701
Number of Observations Used	1701

Response Profile		
Ordered Value	TG_x	Total Frequency
1	0	1614
2	1	87

Probability modeled is TG_x='1'.

Model Convergence Status
Convergence criterion (GCONV=1E-8) satisfied.

Model Fit Statistics		
Criterion	Intercept Only	Intercept and Covariates
AIC	688.785	587.369
SC	694.224	636.320
-2 Log L	686.785	569.369

Testing Global Null Hypothesis: BETA=0			
Test	Chi-Square	DF	Pr > ChiSq
Likelihood Ratio	117.4162	8	<.0001
Score	168.1221	8	<.0001
Wald	113.8738	8	<.0001

Logistic Regression Results

The LOGISTIC Procedure

Analysis of Maximum Likelihood Estimates					
Parameter	DF	Estimate	Standard Error	Wald Chi-Square	Pr > ChiSq
Intercept	1	-9.4374	1.3540	48.5819	<.0001
O1	1	-0.0647	0.0470	1.9007	0.1680
age	1	0.0381	0.0234	2.6599	0.1029
BMI	1	0.2540	0.0291	76.0938	<.0001
SBP	1	0.00228	0.0118	0.0375	0.8465
D1_al	1	0.0881	0.3240	0.0740	0.7856
D2_al	1	0.4030	0.3410	1.3960	0.2374
D1_sm	1	-0.2251	0.6188	0.1323	0.7161
D2_sm	1	0.3696	0.4093	0.8153	0.3666

Odds Ratio Estimates			
Effect	Point Estimate	95% Wald Confidence Limits	
O1	0.937	0.855	1.028
age	1.039	0.992	1.087
BMI	1.289	1.218	1.365
SBP	1.002	0.979	1.026
D1_al	1.092	0.579	2.061
D2_al	1.496	0.767	2.919
D1_sm	0.798	0.237	2.685
D2_sm	1.447	0.649	3.228

Association of Predicted Probabilities and Observed Responses			
Percent Concordant	77.5	Somers' D	0.566
Percent Discordant	21.0	Gamma	0.574
Percent Tied	1.5	Tau-a	0.055
Pairs	140418	c	0.783

Logistic Regression Results

The LOGISTIC Procedure

Model Information	
Data Set	WORK.SORTTEMPTABLESORTED
Response Variable	TC_x
Number of Response Levels	2
Model	binary logit
Optimization Technique	Fisher's scoring

Number of Observations Read	1701
Number of Observations Used	1701

Response Profile		
Ordered Value	TC_x	Total Frequency
1	0	1386
2	1	315

Probability modeled is TC_x='1'.

Model Convergence Status
Convergence criterion (GCONV=1E-8) satisfied.

Model Fit Statistics		
Criterion	Intercept Only	Intercept and Covariates
AIC	1632.121	1582.594
SC	1637.560	1631.545
-2 Log L	1630.121	1564.594

Testing Global Null Hypothesis: BETA=0			
Test	Chi-Square	DF	Pr > ChiSq
Likelihood Ratio	65.5274	8	<.0001
Score	70.1362	8	<.0001
Wald	64.8242	8	<.0001

Logistic Regression Results

The LOGISTIC Procedure

Analysis of Maximum Likelihood Estimates					
Parameter	DF	Estimate	Standard Error	Wald Chi-Square	Pr > ChiSq
Intercept	1	-4.4620	0.8140	30.0512	<.0001
O2	1	-0.0221	0.0248	0.7964	0.3722
age	1	0.0614	0.0136	20.5026	<.0001
BMI	1	0.1134	0.0186	37.0683	<.0001
SBP	1	-0.00830	0.00701	1.4029	0.2362
D1_al	1	0.1719	0.1741	0.9753	0.3234
D2_al	1	0.2989	0.1978	2.2828	0.1308
D1_sm	1	0.1609	0.2805	0.3288	0.5664
D2_sm	1	-0.1004	0.2859	0.1233	0.7255

Odds Ratio Estimates			
Effect	Point Estimate	95% Wald Confidence Limits	
O2	0.978	0.932	1.027
age	1.063	1.035	1.092
BMI	1.120	1.080	1.162
SBP	0.992	0.978	1.005
D1_al	1.188	0.844	1.671
D2_al	1.348	0.915	1.987
D1_sm	1.175	0.678	2.035
D2_sm	0.904	0.516	1.584

Association of Predicted Probabilities and Observed Responses			
Percent Concordant	62.3	Somers' D	0.254
Percent Discordant	36.9	Gamma	0.256
Percent Tied	0.9	Tau-a	0.077
Pairs	436590	c	0.627

Logistic Regression Results

The LOGISTIC Procedure

Model Information	
Data Set	WORK.SORTTEMPTABLESORTED
Response Variable	HDL_x
Number of Response Levels	2
Model	binary logit
Optimization Technique	Fisher's scoring

Number of Observations Read	1701
Number of Observations Used	1701

Response Profile		
Ordered Value	HDL_x	Total Frequency
1	0	1410
2	1	291

Probability modeled is HDL_x='1'.

Model Convergence Status
Convergence criterion (GCONV=1E-8) satisfied.

Model Fit Statistics		
Criterion	Intercept Only	Intercept and Covariates
AIC	1558.714	1413.300
SC	1564.153	1462.251
-2 Log L	1556.714	1395.300

Testing Global Null Hypothesis: BETA=0			
Test	Chi-Square	DF	Pr > ChiSq
Likelihood Ratio	161.4144	8	<.0001
Score	184.4903	8	<.0001
Wald	144.8536	8	<.0001

Logistic Regression Results

The LOGISTIC Procedure

Analysis of Maximum Likelihood Estimates					
Parameter	DF	Estimate	Standard Error	Wald Chi-Square	Pr > ChiSq
Intercept	1	-6.2918	0.8616	53.3207	<.0001
O2	1	-0.00225	0.0265	0.0072	0.9323
age	1	0.00942	0.0145	0.4203	0.5168
BMI	1	0.2241	0.0203	121.9687	<.0001
SBP	1	-0.00569	0.00734	0.6000	0.4386
D1_al	1	0.2781	0.1829	2.3123	0.1284
D2_al	1	0.3890	0.2108	3.4051	0.0650
D1_sm	1	0.2411	0.3007	0.6430	0.4226
D2_sm	1	-0.0874	0.3046	0.0823	0.7742

Odds Ratio Estimates			
Effect	Point Estimate	95% Wald Confidence Limits	
O2	0.998	0.947	1.051
age	1.009	0.981	1.039
BMI	1.251	1.202	1.302
SBP	0.994	0.980	1.009
D1_al	1.321	0.923	1.890
D2_al	1.475	0.976	2.230
D1_sm	1.273	0.706	2.294
D2_sm	0.916	0.504	1.665

Association of Predicted Probabilities and Observed Responses			
Percent Concordant	72.1	Somers' D	0.449
Percent Discordant	27.3	Gamma	0.452
Percent Tied	0.6	Tau-a	0.127
Pairs	410310	c	0.724

Logistic Regression Results

The LOGISTIC Procedure

Model Information	
Data Set	WORK.SORTTEMPTABLESORTED
Response Variable	LDL_x
Number of Response Levels	2
Model	binary logit
Optimization Technique	Fisher's scoring

Number of Observations Read	1701
Number of Observations Used	1701

Response Profile		
Ordered Value	LDL_x	Total Frequency
1	0	1384
2	1	317

Probability modeled is LDL_x='1'.

Model Convergence Status
Convergence criterion (GCONV=1E-8) satisfied.

Model Fit Statistics		
Criterion	Intercept Only	Intercept and Covariates
AIC	1638.032	1543.285
SC	1643.471	1592.236
-2 Log L	1636.032	1525.285

Testing Global Null Hypothesis: BETA=0			
Test	Chi-Square	DF	Pr > ChiSq
Likelihood Ratio	110.7470	8	<.0001
Score	120.4721	8	<.0001
Wald	104.2521	8	<.0001

Logistic Regression Results

The LOGISTIC Procedure

Analysis of Maximum Likelihood Estimates					
Parameter	DF	Estimate	Standard Error	Wald Chi-Square	Pr > ChiSq
Intercept	1	-6.7246	0.8329	65.1823	<.0001
O2	1	-0.0131	0.0253	0.2695	0.6037
age	1	0.0844	0.0137	37.7362	<.0001
BMI	1	0.1340	0.0188	50.6859	<.0001
SBP	1	0.00225	0.00702	0.1030	0.7482
D1_al	1	0.0434	0.1817	0.0571	0.8111
D2_al	1	0.1626	0.2042	0.6343	0.4258
D1_sm	1	0.0942	0.2891	0.1062	0.7445
D2_sm	1	0.1838	0.2714	0.4585	0.4983

Odds Ratio Estimates			
Effect	Point Estimate	95% Wald Confidence Limits	
O2	0.987	0.939	1.037
age	1.088	1.059	1.118
BMI	1.143	1.102	1.186
SBP	1.002	0.989	1.016
D1_al	1.044	0.731	1.491
D2_al	1.177	0.789	1.756
D1_sm	1.099	0.624	1.936
D2_sm	1.202	0.706	2.046

Association of Predicted Probabilities and Observed Responses			
Percent Concordant	67.1	Somers' D	0.348
Percent Discordant	32.2	Gamma	0.351
Percent Tied	0.7	Tau-a	0.106
Pairs	438728	c	0.674

Logistic Regression Results

The LOGISTIC Procedure

Model Information	
Data Set	WORK.SORTTEMPTABLESORTED
Response Variable	TG_x
Number of Response Levels	2
Model	binary logit
Optimization Technique	Fisher's scoring

Number of Observations Read	1701
Number of Observations Used	1701

Response Profile		
Ordered Value	TG_x	Total Frequency
1	0	1614
2	1	87

Probability modeled is TG_x='1'.

Model Convergence Status
Convergence criterion (GCONV=1E-8) satisfied.

Model Fit Statistics		
Criterion	Intercept Only	Intercept and Covariates
AIC	688.785	585.794
SC	694.224	634.745
-2 Log L	686.785	567.794

Testing Global Null Hypothesis: BETA=0			
Test	Chi-Square	DF	Pr > ChiSq
Likelihood Ratio	118.9913	8	<.0001
Score	169.7518	8	<.0001
Wald	113.8178	8	<.0001

Logistic Regression Results

The LOGISTIC Procedure

Analysis of Maximum Likelihood Estimates					
Parameter	DF	Estimate	Standard Error	Wald Chi-Square	Pr > ChiSq
Intercept	1	-9.3016	1.3420	48.0387	<.0001
O2	1	-0.0837	0.0446	3.5167	0.0608
age	1	0.0443	0.0235	3.5426	0.0598
BMI	1	0.2499	0.0290	74.2516	<.0001
SBP	1	0.00241	0.0118	0.0419	0.8378
D1_al	1	0.1107	0.3249	0.1161	0.7333
D2_al	1	0.4118	0.3418	1.4509	0.2284
D1_sm	1	-0.1949	0.6177	0.0995	0.7524
D2_sm	1	0.3222	0.4128	0.6094	0.4350

Odds Ratio Estimates			
Effect	Point Estimate	95% Wald Confidence Limits	
O2	0.920	0.843	1.004
age	1.045	0.998	1.095
BMI	1.284	1.213	1.359
SBP	1.002	0.980	1.026
D1_al	1.117	0.591	2.112
D2_al	1.509	0.772	2.950
D1_sm	0.823	0.245	2.762
D2_sm	1.380	0.615	3.100

Association of Predicted Probabilities and Observed Responses			
Percent Concordant	77.9	Somers' D	0.572
Percent Discordant	20.6	Gamma	0.581
Percent Tied	1.5	Tau-a	0.056
Pairs	140418	c	0.786

Logistic Regression Results

The LOGISTIC Procedure

Model Information	
Data Set	WORK.SORTTEMPTABLESORTED
Response Variable	TC_x
Number of Response Levels	2
Model	binary logit
Optimization Technique	Fisher's scoring

Number of Observations Read	1701
Number of Observations Used	1701

Response Profile		
Ordered Value	TC_x	Total Frequency
1	0	1386
2	1	315

Probability modeled is TC_x='1'.

Model Convergence Status
Convergence criterion (GCONV=1E-8) satisfied.

Model Fit Statistics		
Criterion	Intercept Only	Intercept and Covariates
AIC	1632.121	1583.113
SC	1637.560	1632.064
-2 Log L	1630.121	1565.113

Testing Global Null Hypothesis: BETA=0			
Test	Chi-Square	DF	Pr > ChiSq
Likelihood Ratio	65.0083	8	<.0001
Score	69.7041	8	<.0001
Wald	64.3710	8	<.0001

Logistic Regression Results

The LOGISTIC Procedure

Analysis of Maximum Likelihood Estimates					
Parameter	DF	Estimate	Standard Error	Wald Chi-Square	Pr > ChiSq
Intercept	1	-4.9405	0.8965	30.3679	<.0001
O3	1	0.0186	0.0356	0.2731	0.6013
age	1	0.0616	0.0137	20.3467	<.0001
BMI	1	0.1137	0.0186	37.2776	<.0001
SBP	1	-0.00826	0.00700	1.3905	0.2383
D1_al	1	0.1703	0.1742	0.9564	0.3281
D2_al	1	0.3031	0.1978	2.3491	0.1254
D1_sm	1	0.1573	0.2810	0.3136	0.5755
D2_sm	1	-0.1094	0.2862	0.1462	0.7022

Odds Ratio Estimates			
Effect	Point Estimate	95% Wald Confidence Limits	
O3	1.019	0.950	1.092
age	1.064	1.035	1.092
BMI	1.120	1.080	1.162
SBP	0.992	0.978	1.005
D1_al	1.186	0.843	1.668
D2_al	1.354	0.919	1.995
D1_sm	1.170	0.675	2.030
D2_sm	0.896	0.512	1.571

Association of Predicted Probabilities and Observed Responses			
Percent Concordant	62.0	Somers' D	0.248
Percent Discordant	37.2	Gamma	0.250
Percent Tied	0.9	Tau-a	0.075
Pairs	436590	c	0.624

Logistic Regression Results

The LOGISTIC Procedure

Model Information	
Data Set	WORK.SORTTEMPTABLESORTED
Response Variable	HDL_x
Number of Response Levels	2
Model	binary logit
Optimization Technique	Fisher's scoring

Number of Observations Read	1701
Number of Observations Used	1701

Response Profile		
Ordered Value	HDL_x	Total Frequency
1	0	1410
2	1	291

Probability modeled is HDL_x='1'.

Model Convergence Status
Convergence criterion (GCONV=1E-8) satisfied.

Model Fit Statistics		
Criterion	Intercept Only	Intercept and Covariates
AIC	1558.714	1411.876
SC	1564.153	1460.826
-2 Log L	1556.714	1393.876

Testing Global Null Hypothesis: BETA=0			
Test	Chi-Square	DF	Pr > ChiSq
Likelihood Ratio	162.8388	8	<.0001
Score	185.6345	8	<.0001
Wald	145.8961	8	<.0001

Logistic Regression Results

The LOGISTIC Procedure

Analysis of Maximum Likelihood Estimates					
Parameter	DF	Estimate	Standard Error	Wald Chi-Square	Pr > ChiSq
Intercept	1	-5.7465	0.9394	37.4234	<.0001
O3	1	-0.0453	0.0378	1.4356	0.2308
age	1	0.00741	0.0146	0.2582	0.6113
BMI	1	0.2251	0.0203	122.7234	<.0001
SBP	1	-0.00587	0.00736	0.6373	0.4247
D1_al	1	0.2736	0.1829	2.2382	0.1346
D2_al	1	0.3843	0.2108	3.3227	0.0683
D1_sm	1	0.2539	0.3009	0.7125	0.3986
D2_sm	1	-0.0759	0.3054	0.0617	0.8038

Odds Ratio Estimates			
Effect	Point Estimate	95% Wald Confidence Limits	
O3	0.956	0.887	1.029
age	1.007	0.979	1.037
BMI	1.252	1.204	1.303
SBP	0.994	0.980	1.009
D1_al	1.315	0.919	1.881
D2_al	1.469	0.972	2.220
D1_sm	1.289	0.715	2.325
D2_sm	0.927	0.509	1.687

Association of Predicted Probabilities and Observed Responses			
Percent Concordant	72.3	Somers' D	0.452
Percent Discordant	27.1	Gamma	0.455
Percent Tied	0.6	Tau-a	0.128
Pairs	410310	c	0.726

Logistic Regression Results

The LOGISTIC Procedure

Model Information	
Data Set	WORK.SORTTEMPTABLESORTED
Response Variable	LDL_x
Number of Response Levels	2
Model	binary logit
Optimization Technique	Fisher's scoring

Number of Observations Read	1701
Number of Observations Used	1701

Response Profile		
Ordered Value	LDL_x	Total Frequency
1	0	1384
2	1	317

Probability modeled is LDL_x='1'.

Model Convergence Status
Convergence criterion (GCONV=1E-8) satisfied.

Model Fit Statistics		
Criterion	Intercept Only	Intercept and Covariates
AIC	1638.032	1543.161
SC	1643.471	1592.112
-2 Log L	1636.032	1525.161

Testing Global Null Hypothesis: BETA=0			
Test	Chi-Square	DF	Pr > ChiSq
Likelihood Ratio	110.8710	8	<.0001
Score	120.6410	8	<.0001
Wald	104.3586	8	<.0001

Logistic Regression Results

The LOGISTIC Procedure

Analysis of Maximum Likelihood Estimates					
Parameter	DF	Estimate	Standard Error	Wald Chi-Square	Pr > ChiSq
Intercept	1	-7.1563	0.9185	60.7099	<.0001
O3	1	0.0227	0.0363	0.3916	0.5315
age	1	0.0851	0.0139	37.7540	<.0001
BMI	1	0.1341	0.0188	50.7752	<.0001
SBP	1	0.00228	0.00701	0.1058	0.7449
D1_al	1	0.0430	0.1819	0.0558	0.8132
D2_al	1	0.1661	0.2042	0.6613	0.4161
D1_sm	1	0.0886	0.2894	0.0938	0.7595
D2_sm	1	0.1721	0.2719	0.4006	0.5268

Odds Ratio Estimates			
Effect	Point Estimate	95% Wald Confidence Limits	
O3	1.023	0.953	1.098
age	1.089	1.060	1.119
BMI	1.144	1.102	1.186
SBP	1.002	0.989	1.016
D1_al	1.044	0.731	1.491
D2_al	1.181	0.791	1.762
D1_sm	1.093	0.620	1.927
D2_sm	1.188	0.697	2.024

Association of Predicted Probabilities and Observed Responses			
Percent Concordant	67.0	Somers' D	0.347
Percent Discordant	32.3	Gamma	0.350
Percent Tied	0.7	Tau-a	0.105
Pairs	438728	c	0.674

Logistic Regression Results

The LOGISTIC Procedure

Model Information	
Data Set	WORK.SORTTEMPTABLESORTED
Response Variable	TG_x
Number of Response Levels	2
Model	binary logit
Optimization Technique	Fisher's scoring

Number of Observations Read	1701
Number of Observations Used	1701

Response Profile		
Ordered Value	TG_x	Total Frequency
1	0	1614
2	1	87

Probability modeled is TG_x='1'.

Model Convergence Status
Convergence criterion (GCONV=1E-8) satisfied.

Model Fit Statistics		
Criterion	Intercept Only	Intercept and Covariates
AIC	688.785	589.211
SC	694.224	638.161
-2 Log L	686.785	571.211

Testing Global Null Hypothesis: BETA=0			
Test	Chi-Square	DF	Pr > ChiSq
Likelihood Ratio	115.5748	8	<.0001
Score	167.0365	8	<.0001
Wald	112.9079	8	<.0001

Logistic Regression Results

The LOGISTIC Procedure

Analysis of Maximum Likelihood Estimates					
Parameter	DF	Estimate	Standard Error	Wald Chi-Square	Pr > ChiSq
Intercept	1	-10.2336	1.5220	45.2084	<.0001
O3	1	0.0124	0.0648	0.0368	0.8479
age	1	0.0408	0.0236	3.0023	0.0831
BMI	1	0.2504	0.0289	75.0602	<.0001
SBP	1	0.00191	0.0117	0.0266	0.8704
D1_al	1	0.1030	0.3239	0.1010	0.7506
D2_al	1	0.4272	0.3415	1.5652	0.2109
D1_sm	1	-0.2054	0.6185	0.1103	0.7398
D2_sm	1	0.3330	0.4074	0.6680	0.4137

Odds Ratio Estimates			
Effect	Point Estimate	95% Wald Confidence Limits	
O3	1.013	0.892	1.150
age	1.042	0.995	1.091
BMI	1.285	1.214	1.359
SBP	1.002	0.979	1.025
D1_al	1.108	0.588	2.091
D2_al	1.533	0.785	2.994
D1_sm	0.814	0.242	2.737
D2_sm	1.395	0.628	3.100

Association of Predicted Probabilities and Observed Responses			
Percent Concordant	77.2	Somers' D	0.560
Percent Discordant	21.2	Gamma	0.569
Percent Tied	1.6	Tau-a	0.054
Pairs	140418	c	0.780

Logistic Regression Results

The LOGISTIC Procedure

Model Information	
Data Set	WORK.SORTTEMPTABLESORTED
Response Variable	TC_x
Number of Response Levels	2
Model	binary logit
Optimization Technique	Fisher's scoring

Number of Observations Read	1701
Number of Observations Used	1701

Response Profile		
Ordered Value	TC_x	Total Frequency
1	0	1386
2	1	315

Probability modeled is TC_x='1'.

Model Convergence Status
Convergence criterion (GCONV=1E-8) satisfied.

Model Fit Statistics		
Criterion	Intercept Only	Intercept and Covariates
AIC	1632.121	1582.460
SC	1637.560	1631.411
-2 Log L	1630.121	1564.460

Testing Global Null Hypothesis: BETA=0			
Test	Chi-Square	DF	Pr > ChiSq
Likelihood Ratio	65.6612	8	<.0001
Score	70.4485	8	<.0001
Wald	64.9515	8	<.0001

Logistic Regression Results

The LOGISTIC Procedure

Analysis of Maximum Likelihood Estimates					
Parameter	DF	Estimate	Standard Error	Wald Chi-Square	Pr > ChiSq
Intercept	1	-4.3817	0.8368	27.4154	<.0001
O4	1	-0.0285	0.0296	0.9273	0.3356
age	1	0.0606	0.0135	20.0350	<.0001
BMI	1	0.1132	0.0186	36.8607	<.0001
SBP	1	-0.00858	0.00702	1.4923	0.2219
D1_al	1	0.1595	0.1744	0.8370	0.3603
D2_al	1	0.2959	0.1979	2.2356	0.1349
D1_sm	1	0.1506	0.2813	0.2865	0.5925
D2_sm	1	-0.0980	0.2857	0.1177	0.7315

Odds Ratio Estimates			
Effect	Point Estimate	95% Wald Confidence Limits	
O4	0.972	0.917	1.030
age	1.062	1.035	1.091
BMI	1.120	1.080	1.161
SBP	0.991	0.978	1.005
D1_al	1.173	0.833	1.651
D2_al	1.344	0.912	1.981
D1_sm	1.163	0.670	2.018
D2_sm	0.907	0.518	1.587

Association of Predicted Probabilities and Observed Responses			
Percent Concordant	61.9	Somers' D	0.248
Percent Discordant	37.2	Gamma	0.250
Percent Tied	0.9	Tau-a	0.075
Pairs	436590	c	0.624

Logistic Regression Results

The LOGISTIC Procedure

Model Information	
Data Set	WORK.SORTTEMPTABLESORTED
Response Variable	HDL_x
Number of Response Levels	2
Model	binary logit
Optimization Technique	Fisher's scoring

Number of Observations Read	1701
Number of Observations Used	1701

Response Profile		
Ordered Value	HDL_x	Total Frequency
1	0	1410
2	1	291

Probability modeled is HDL_x='1'.

Model Convergence Status
Convergence criterion (GCONV=1E-8) satisfied.

Model Fit Statistics		
Criterion	Intercept Only	Intercept and Covariates
AIC	1558.714	1411.579
SC	1564.153	1460.529
-2 Log L	1556.714	1393.579

Testing Global Null Hypothesis: BETA=0			
Test	Chi-Square	DF	Pr > ChiSq
Likelihood Ratio	163.1358	8	<.0001
Score	186.4278	8	<.0001
Wald	146.2209	8	<.0001

Logistic Regression Results

The LOGISTIC Procedure

Analysis of Maximum Likelihood Estimates					
Parameter	DF	Estimate	Standard Error	Wald Chi-Square	Pr > ChiSq
Intercept	1	-5.8573	0.8834	43.9639	<.0001
O4	1	-0.0414	0.0315	1.7294	0.1885
age	1	0.00932	0.0145	0.4133	0.5203
BMI	1	0.2229	0.0203	120.8126	<.0001
SBP	1	-0.00602	0.00736	0.6699	0.4131
D1_al	1	0.2613	0.1835	2.0283	0.1544
D2_al	1	0.3814	0.2109	3.2716	0.0705
D1_sm	1	0.2230	0.3017	0.5462	0.4599
D2_sm	1	-0.0797	0.3043	0.0686	0.7933

Odds Ratio Estimates			
Effect	Point Estimate	95% Wald Confidence Limits	
O4	0.959	0.902	1.021
age	1.009	0.981	1.038
BMI	1.250	1.201	1.300
SBP	0.994	0.980	1.008
D1_al	1.299	0.906	1.861
D2_al	1.464	0.969	2.214
D1_sm	1.250	0.692	2.258
D2_sm	0.923	0.509	1.676

Association of Predicted Probabilities and Observed Responses			
Percent Concordant	72.3	Somers' D	0.453
Percent Discordant	27.0	Gamma	0.456
Percent Tied	0.6	Tau-a	0.129
Pairs	410310	c	0.726

Logistic Regression Results

The LOGISTIC Procedure

Model Information	
Data Set	WORK.SORTTEMPTABLESORTED
Response Variable	LDL_x
Number of Response Levels	2
Model	binary logit
Optimization Technique	Fisher's scoring

Number of Observations Read	1701
Number of Observations Used	1701

Response Profile		
Ordered Value	LDL_x	Total Frequency
1	0	1384
2	1	317

Probability modeled is LDL_x='1'.

Model Convergence Status
Convergence criterion (GCONV=1E-8) satisfied.

Model Fit Statistics		
Criterion	Intercept Only	Intercept and Covariates
AIC	1638.032	1542.079
SC	1643.471	1591.030
-2 Log L	1636.032	1524.079

Testing Global Null Hypothesis: BETA=0			
Test	Chi-Square	DF	Pr > ChiSq
Likelihood Ratio	111.9535	8	<.0001
Score	121.8281	8	<.0001
Wald	105.1022	8	<.0001

Logistic Regression Results

The LOGISTIC Procedure

Analysis of Maximum Likelihood Estimates					
Parameter	DF	Estimate	Standard Error	Wald Chi-Square	Pr > ChiSq
Intercept	1	-6.4632	0.8535	57.3447	<.0001
O4	1	-0.0365	0.0300	1.4761	0.2244
age	1	0.0839	0.0137	37.3773	<.0001
BMI	1	0.1334	0.0188	50.1603	<.0001
SBP	1	0.00195	0.00703	0.0773	0.7810
D1_al	1	0.0297	0.1820	0.0266	0.8705
D2_al	1	0.1572	0.2043	0.5923	0.4415
D1_sm	1	0.0790	0.2901	0.0742	0.7854
D2_sm	1	0.1889	0.2713	0.4849	0.4862

Odds Ratio Estimates			
Effect	Point Estimate	95% Wald Confidence Limits	
O4	0.964	0.909	1.023
age	1.087	1.059	1.117
BMI	1.143	1.101	1.186
SBP	1.002	0.988	1.016
D1_al	1.030	0.721	1.472
D2_al	1.170	0.784	1.747
D1_sm	1.082	0.613	1.911
D2_sm	1.208	0.710	2.056

Association of Predicted Probabilities and Observed Responses			
Percent Concordant	67.0	Somers' D	0.347
Percent Discordant	32.3	Gamma	0.350
Percent Tied	0.7	Tau-a	0.105
Pairs	438728	c	0.674

Logistic Regression Results

The LOGISTIC Procedure

Model Information	
Data Set	WORK.SORTTEMPTABLESORTED
Response Variable	TG_x
Number of Response Levels	2
Model	binary logit
Optimization Technique	Fisher's scoring

Number of Observations Read	1701
Number of Observations Used	1701

Response Profile		
Ordered Value	TG_x	Total Frequency
1	0	1614
2	1	87

Probability modeled is TG_x='1'.

Model Convergence Status
Convergence criterion (GCONV=1E-8) satisfied.

Model Fit Statistics		
Criterion	Intercept Only	Intercept and Covariates
AIC	688.785	585.974
SC	694.224	634.925
-2 Log L	686.785	567.974

Testing Global Null Hypothesis: BETA=0			
Test	Chi-Square	DF	Pr > ChiSq
Likelihood Ratio	118.8114	8	<.0001
Score	170.5375	8	<.0001
Wald	114.0585	8	<.0001

Logistic Regression Results

The LOGISTIC Procedure

Analysis of Maximum Likelihood Estimates					
Parameter	DF	Estimate	Standard Error	Wald Chi-Square	Pr > ChiSq
Intercept	1	-9.0272	1.3970	41.7530	<.0001
O4	1	-0.0973	0.0538	3.2711	0.0705
age	1	0.0393	0.0234	2.8333	0.0923
BMI	1	0.2475	0.0289	73.4550	<.0001
SBP	1	0.00147	0.0118	0.0157	0.9004
D1_al	1	0.0664	0.3248	0.0418	0.8380
D2_al	1	0.4125	0.3426	1.4502	0.2285
D1_sm	1	-0.2707	0.6234	0.1886	0.6641
D2_sm	1	0.3669	0.4087	0.8060	0.3693

Odds Ratio Estimates			
Effect	Point Estimate	95% Wald Confidence Limits	
O4	0.907	0.816	1.008
age	1.040	0.994	1.089
BMI	1.281	1.210	1.355
SBP	1.001	0.979	1.025
D1_al	1.069	0.565	2.020
D2_al	1.511	0.772	2.956
D1_sm	0.763	0.225	2.588
D2_sm	1.443	0.648	3.215

Association of Predicted Probabilities and Observed Responses			
Percent Concordant	78.1	Somers' D	0.576
Percent Discordant	20.5	Gamma	0.584
Percent Tied	1.4	Tau-a	0.056
Pairs	140418	c	0.788

Logistic Regression Results

The LOGISTIC Procedure

Model Information	
Data Set	WORK.SORTTEMPTABLESORTED
Response Variable	TC_x
Number of Response Levels	2
Model	binary logit
Optimization Technique	Fisher's scoring

Number of Observations Read	1701
Number of Observations Used	1701

Response Profile		
Ordered Value	TC_x	Total Frequency
1	0	1386
2	1	315

Probability modeled is TC_x='1'.

Model Convergence Status
Convergence criterion (GCONV=1E-8) satisfied.

Model Fit Statistics		
Criterion	Intercept Only	Intercept and Covariates
AIC	1632.121	1582.460
SC	1637.560	1631.411
-2 Log L	1630.121	1564.460

Testing Global Null Hypothesis: BETA=0			
Test	Chi-Square	DF	Pr > ChiSq
Likelihood Ratio	65.6614	8	<.0001
Score	70.2925	8	<.0001
Wald	64.9460	8	<.0001

Logistic Regression Results

The LOGISTIC Procedure

Analysis of Maximum Likelihood Estimates					
Parameter	DF	Estimate	Standard Error	Wald Chi-Square	Pr > ChiSq
Intercept	1	-4.4107	0.8251	28.5791	<.0001
O5	1	-0.0265	0.0276	0.9259	0.3359
age	1	0.0600	0.0136	19.5225	<.0001
BMI	1	0.1136	0.0186	37.2270	<.0001
SBP	1	-0.00844	0.00701	1.4507	0.2284
D1_al	1	0.1652	0.1742	0.8992	0.3430
D2_al	1	0.3001	0.1978	2.3013	0.1293
D1_sm	1	0.1622	0.2808	0.3336	0.5635
D2_sm	1	-0.0868	0.2857	0.0922	0.7614

Odds Ratio Estimates			
Effect	Point Estimate	95% Wald Confidence Limits	
O5	0.974	0.923	1.028
age	1.062	1.034	1.090
BMI	1.120	1.080	1.162
SBP	0.992	0.978	1.005
D1_al	1.180	0.838	1.660
D2_al	1.350	0.916	1.989
D1_sm	1.176	0.678	2.039
D2_sm	0.917	0.524	1.605

Association of Predicted Probabilities and Observed Responses			
Percent Concordant	62.3	Somers' D	0.254
Percent Discordant	36.9	Gamma	0.256
Percent Tied	0.9	Tau-a	0.077
Pairs	436590	c	0.627

Logistic Regression Results

The LOGISTIC Procedure

Model Information	
Data Set	WORK.SORTTEMPTABLESORTED
Response Variable	HDL_x
Number of Response Levels	2
Model	binary logit
Optimization Technique	Fisher's scoring

Number of Observations Read	1701
Number of Observations Used	1701

Response Profile		
Ordered Value	HDL_x	Total Frequency
1	0	1410
2	1	291

Probability modeled is HDL_x='1'.

Model Convergence Status
Convergence criterion (GCONV=1E-8) satisfied.

Model Fit Statistics		
Criterion	Intercept Only	Intercept and Covariates
AIC	1558.714	1412.798
SC	1564.153	1461.749
-2 Log L	1556.714	1394.798

Testing Global Null Hypothesis: BETA=0			
Test	Chi-Square	DF	Pr > ChiSq
Likelihood Ratio	161.9164	8	<.0001
Score	185.0034	8	<.0001
Wald	145.3659	8	<.0001

Logistic Regression Results

The LOGISTIC Procedure

Analysis of Maximum Likelihood Estimates					
Parameter	DF	Estimate	Standard Error	Wald Chi-Square	Pr > ChiSq
Intercept	1	-6.0847	0.8745	48.4148	<.0001
O5	1	-0.0210	0.0294	0.5086	0.4757
age	1	0.00874	0.0145	0.3617	0.5476
BMI	1	0.2238	0.0203	121.8471	<.0001
SBP	1	-0.00578	0.00735	0.6182	0.4317
D1_al	1	0.2737	0.1831	2.2364	0.1348
D2_al	1	0.3861	0.2109	3.3527	0.0671
D1_sm	1	0.2421	0.3008	0.6481	0.4208
D2_sm	1	-0.0790	0.3047	0.0672	0.7955

Odds Ratio Estimates			
Effect	Point Estimate	95% Wald Confidence Limits	
O5	0.979	0.924	1.037
age	1.009	0.980	1.038
BMI	1.251	1.202	1.302
SBP	0.994	0.980	1.009
D1_al	1.315	0.918	1.882
D2_al	1.471	0.973	2.224
D1_sm	1.274	0.707	2.297
D2_sm	0.924	0.509	1.679

Association of Predicted Probabilities and Observed Responses			
Percent Concordant	72.2	Somers' D	0.450
Percent Discordant	27.2	Gamma	0.452
Percent Tied	0.6	Tau-a	0.128
Pairs	410310	c	0.725

Logistic Regression Results

The LOGISTIC Procedure

Model Information	
Data Set	WORK.SORTTEMPTABLESORTED
Response Variable	LDL_x
Number of Response Levels	2
Model	binary logit
Optimization Technique	Fisher's scoring

Number of Observations Read	1701
Number of Observations Used	1701

Response Profile		
Ordered Value	LDL_x	Total Frequency
1	0	1384
2	1	317

Probability modeled is LDL_x='1'.

Model Convergence Status
Convergence criterion (GCONV=1E-8) satisfied.

Model Fit Statistics		
Criterion	Intercept Only	Intercept and Covariates
AIC	1638.032	1542.907
SC	1643.471	1591.858
-2 Log L	1636.032	1524.907

Testing Global Null Hypothesis: BETA=0			
Test	Chi-Square	DF	Pr > ChiSq
Likelihood Ratio	111.1253	8	<.0001
Score	120.7850	8	<.0001
Wald	104.5494	8	<.0001

Logistic Regression Results

The LOGISTIC Procedure

Analysis of Maximum Likelihood Estimates					
Parameter	DF	Estimate	Standard Error	Wald Chi-Square	Pr > ChiSq
Intercept	1	-6.6199	0.8440	61.5186	<.0001
O5	1	-0.0225	0.0280	0.6465	0.4214
age	1	0.0834	0.0137	36.8427	<.0001
BMI	1	0.1341	0.0188	50.7764	<.0001
SBP	1	0.00212	0.00702	0.0917	0.7621
D1_al	1	0.0382	0.1818	0.0442	0.8334
D2_al	1	0.1631	0.2041	0.6386	0.4242
D1_sm	1	0.0941	0.2893	0.1059	0.7449
D2_sm	1	0.1953	0.2715	0.5176	0.4719

Odds Ratio Estimates			
Effect	Point Estimate	95% Wald Confidence Limits	
O5	0.978	0.926	1.033
age	1.087	1.058	1.117
BMI	1.143	1.102	1.186
SBP	1.002	0.988	1.016
D1_al	1.039	0.728	1.484
D2_al	1.177	0.789	1.756
D1_sm	1.099	0.623	1.937
D2_sm	1.216	0.714	2.070

Association of Predicted Probabilities and Observed Responses			
Percent Concordant	67.1	Somers' D	0.350
Percent Discordant	32.1	Gamma	0.353
Percent Tied	0.7	Tau-a	0.106
Pairs	438728	c	0.675

Logistic Regression Results

The LOGISTIC Procedure

Model Information	
Data Set	WORK.SORTTEMPTABLESORTED
Response Variable	TG_x
Number of Response Levels	2
Model	binary logit
Optimization Technique	Fisher's scoring

Number of Observations Read	1701
Number of Observations Used	1701

Response Profile		
Ordered Value	TG_x	Total Frequency
1	0	1614
2	1	87

Probability modeled is TG_x='1'.

Model Convergence Status
Convergence criterion (GCONV=1E-8) satisfied.

Model Fit Statistics		
Criterion	Intercept Only	Intercept and Covariates
AIC	688.785	588.980
SC	694.224	637.931
-2 Log L	686.785	570.980

Testing Global Null Hypothesis: BETA=0			
Test	Chi-Square	DF	Pr > ChiSq
Likelihood Ratio	115.8055	8	<.0001
Score	167.0912	8	<.0001
Wald	113.1717	8	<.0001

Logistic Regression Results

The LOGISTIC Procedure

Analysis of Maximum Likelihood Estimates					
Parameter	DF	Estimate	Standard Error	Wald Chi-Square	Pr > ChiSq
Intercept	1	-9.8023	1.3788	50.5393	<.0001
O5	1	-0.0257	0.0497	0.2674	0.6051
age	1	0.0398	0.0234	2.8991	0.0886
BMI	1	0.2504	0.0289	75.1294	<.0001
SBP	1	0.00173	0.0117	0.0219	0.8824
D1_al	1	0.0964	0.3242	0.0884	0.7662
D2_al	1	0.4223	0.3411	1.5329	0.2157
D1_sm	1	-0.2039	0.6187	0.1086	0.7417
D2_sm	1	0.3481	0.4077	0.7290	0.3932

Odds Ratio Estimates			
Effect	Point Estimate	95% Wald Confidence Limits	
O5	0.975	0.884	1.074
age	1.041	0.994	1.089
BMI	1.285	1.214	1.359
SBP	1.002	0.979	1.025
D1_al	1.101	0.583	2.079
D2_al	1.525	0.782	2.977
D1_sm	0.816	0.243	2.742
D2_sm	1.416	0.637	3.149

Association of Predicted Probabilities and Observed Responses			
Percent Concordant	77.4	Somers' D	0.563
Percent Discordant	21.1	Gamma	0.572
Percent Tied	1.5	Tau-a	0.055
Pairs	140418	c	0.782

Logistic Regression Results

The LOGISTIC Procedure

Model Information	
Data Set	WORK.SORTTEMPTABLESORTED
Response Variable	TC_x
Number of Response Levels	2
Model	binary logit
Optimization Technique	Fisher's scoring

Number of Observations Read	1701
Number of Observations Used	1701

Response Profile		
Ordered Value	TC_x	Total Frequency
1	0	1386
2	1	315

Probability modeled is TC_x='1'.

Model Convergence Status
Convergence criterion (GCONV=1E-8) satisfied.

Model Fit Statistics		
Criterion	Intercept Only	Intercept and Covariates
AIC	1632.121	1583.279
SC	1637.560	1632.229
-2 Log L	1630.121	1565.279

Testing Global Null Hypothesis: BETA=0			
Test	Chi-Square	DF	Pr > ChiSq
Likelihood Ratio	64.8429	8	<.0001
Score	69.5314	8	<.0001
Wald	64.2894	8	<.0001

Logistic Regression Results

The LOGISTIC Procedure

Analysis of Maximum Likelihood Estimates					
Parameter	DF	Estimate	Standard Error	Wald Chi-Square	Pr > ChiSq
Intercept	1	-4.5555	0.8866	26.4012	<.0001
O6	1	-0.0123	0.0373	0.1085	0.7419
age	1	0.0604	0.0136	19.7761	<.0001
BMI	1	0.1140	0.0186	37.4717	<.0001
SBP	1	-0.00833	0.00701	1.4140	0.2344
D1_al	1	0.1648	0.1746	0.8918	0.3450
D2_al	1	0.2974	0.1981	2.2523	0.1334
D1_sm	1	0.1633	0.2807	0.3385	0.5607
D2_sm	1	-0.0997	0.2856	0.1218	0.7271

Odds Ratio Estimates			
Effect	Point Estimate	95% Wald Confidence Limits	
O6	0.988	0.918	1.063
age	1.062	1.034	1.091
BMI	1.121	1.081	1.162
SBP	0.992	0.978	1.005
D1_al	1.179	0.838	1.660
D2_al	1.346	0.913	1.985
D1_sm	1.177	0.679	2.041
D2_sm	0.905	0.517	1.584

Association of Predicted Probabilities and Observed Responses			
Percent Concordant	62.1	Somers' D	0.252
Percent Discordant	37.0	Gamma	0.254
Percent Tied	0.9	Tau-a	0.076
Pairs	436590	c	0.626

Logistic Regression Results

The LOGISTIC Procedure

Model Information	
Data Set	WORK.SORTTEMPTABLESORTED
Response Variable	HDL_x
Number of Response Levels	2
Model	binary logit
Optimization Technique	Fisher's scoring

Number of Observations Read	1701
Number of Observations Used	1701

Response Profile		
Ordered Value	HDL_x	Total Frequency
1	0	1410
2	1	291

Probability modeled is HDL_x='1'.

Model Convergence Status
Convergence criterion (GCONV=1E-8) satisfied.

Model Fit Statistics		
Criterion	Intercept Only	Intercept and Covariates
AIC	1558.714	1411.624
SC	1564.153	1460.575
-2 Log L	1556.714	1393.624

Testing Global Null Hypothesis: BETA=0			
Test	Chi-Square	DF	Pr > ChiSq
Likelihood Ratio	163.0900	8	<.0001
Score	186.0487	8	<.0001
Wald	146.4426	8	<.0001

Logistic Regression Results

The LOGISTIC Procedure

Analysis of Maximum Likelihood Estimates					
Parameter	DF	Estimate	Standard Error	Wald Chi-Square	Pr > ChiSq
Intercept	1	-5.7058	0.9370	37.0809	<.0001
O6	1	-0.0513	0.0395	1.6871	0.1940
age	1	0.00790	0.0145	0.2952	0.5869
BMI	1	0.2245	0.0203	122.5089	<.0001
SBP	1	-0.00576	0.00735	0.6131	0.4336
D1_al	1	0.2612	0.1835	2.0270	0.1545
D2_al	1	0.3727	0.2110	3.1210	0.0773
D1_sm	1	0.2399	0.3013	0.6340	0.4259
D2_sm	1	-0.0850	0.3054	0.0774	0.7808

Odds Ratio Estimates			
Effect	Point Estimate	95% Wald Confidence Limits	
O6	0.950	0.879	1.026
age	1.008	0.980	1.037
BMI	1.252	1.203	1.302
SBP	0.994	0.980	1.009
D1_al	1.298	0.906	1.860
D2_al	1.452	0.960	2.195
D1_sm	1.271	0.704	2.294
D2_sm	0.919	0.505	1.671

Association of Predicted Probabilities and Observed Responses			
Percent Concordant	72.4	Somers' D	0.454
Percent Discordant	27.0	Gamma	0.456
Percent Tied	0.6	Tau-a	0.129
Pairs	410310	c	0.727

Logistic Regression Results

The LOGISTIC Procedure

Model Information	
Data Set	WORK.SORTTEMPTABLESORTED
Response Variable	LDL_x
Number of Response Levels	2
Model	binary logit
Optimization Technique	Fisher's scoring

Number of Observations Read	1701
Number of Observations Used	1701

Response Profile		
Ordered Value	LDL_x	Total Frequency
1	0	1384
2	1	317

Probability modeled is LDL_x='1'.

Model Convergence Status
Convergence criterion (GCONV=1E-8) satisfied.

Model Fit Statistics		
Criterion	Intercept Only	Intercept and Covariates
AIC	1638.032	1543.549
SC	1643.471	1592.500
-2 Log L	1636.032	1525.549

Testing Global Null Hypothesis: BETA=0			
Test	Chi-Square	DF	Pr > ChiSq
Likelihood Ratio	110.4829	8	<.0001
Score	120.2876	8	<.0001
Wald	104.1231	8	<.0001

Logistic Regression Results

The LOGISTIC Procedure

Analysis of Maximum Likelihood Estimates					
Parameter	DF	Estimate	Standard Error	Wald Chi-Square	Pr > ChiSq
Intercept	1	-6.8332	0.9076	56.6877	<.0001
O6	1	-0.00260	0.0379	0.0047	0.9452
age	1	0.0840	0.0138	37.2348	<.0001
BMI	1	0.1343	0.0188	50.9366	<.0001
SBP	1	0.00221	0.00702	0.0996	0.7524
D1_al	1	0.0407	0.1822	0.0498	0.8234
D2_al	1	0.1630	0.2046	0.6352	0.4255
D1_sm	1	0.0952	0.2892	0.1083	0.7420
D2_sm	1	0.1821	0.2714	0.4501	0.5023

Odds Ratio Estimates			
Effect	Point Estimate	95% Wald Confidence Limits	
O6	0.997	0.926	1.074
age	1.088	1.059	1.117
BMI	1.144	1.102	1.187
SBP	1.002	0.989	1.016
D1_al	1.042	0.729	1.489
D2_al	1.177	0.788	1.758
D1_sm	1.100	0.624	1.939
D2_sm	1.200	0.705	2.042

Association of Predicted Probabilities and Observed Responses			
Percent Concordant	67.0	Somers' D	0.347
Percent Discordant	32.3	Gamma	0.350
Percent Tied	0.7	Tau-a	0.105
Pairs	438728	c	0.674

Logistic Regression Results

The LOGISTIC Procedure

Model Information	
Data Set	WORK.SORTTEMPTABLESORTED
Response Variable	TG_x
Number of Response Levels	2
Model	binary logit
Optimization Technique	Fisher's scoring

Number of Observations Read	1701
Number of Observations Used	1701

Response Profile		
Ordered Value	TG_x	Total Frequency
1	0	1614
2	1	87

Probability modeled is TG_x='1'.

Model Convergence Status
Convergence criterion (GCONV=1E-8) satisfied.

Model Fit Statistics		
Criterion	Intercept Only	Intercept and Covariates
AIC	688.785	589.228
SC	694.224	638.178
-2 Log L	686.785	571.228

Testing Global Null Hypothesis: BETA=0			
Test	Chi-Square	DF	Pr > ChiSq
Likelihood Ratio	115.5578	8	<.0001
Score	167.0616	8	<.0001
Wald	112.8663	8	<.0001

Logistic Regression Results

The LOGISTIC Procedure

Analysis of Maximum Likelihood Estimates					
Parameter	DF	Estimate	Standard Error	Wald Chi-Square	Pr > ChiSq
Intercept	1	-10.1868	1.5043	45.8568	<.0001
O6	1	0.00948	0.0674	0.0198	0.8882
age	1	0.0405	0.0234	2.9838	0.0841
BMI	1	0.2505	0.0289	75.1391	<.0001
SBP	1	0.00188	0.0117	0.0256	0.8728
D1_al	1	0.1069	0.3246	0.1085	0.7418
D2_al	1	0.4276	0.3417	1.5665	0.2107
D1_sm	1	-0.1997	0.6179	0.1044	0.7466
D2_sm	1	0.3356	0.4073	0.6791	0.4099

Odds Ratio Estimates			
Effect	Point Estimate	95% Wald Confidence Limits	
O6	1.010	0.885	1.152
age	1.041	0.995	1.090
BMI	1.285	1.214	1.360
SBP	1.002	0.979	1.025
D1_al	1.113	0.589	2.102
D2_al	1.534	0.785	2.996
D1_sm	0.819	0.244	2.750
D2_sm	1.399	0.630	3.108

Association of Predicted Probabilities and Observed Responses			
Percent Concordant	77.3	Somers' D	0.562
Percent Discordant	21.1	Gamma	0.570
Percent Tied	1.5	Tau-a	0.055
Pairs	140418	c	0.781

Logistic Regression Results

The LOGISTIC Procedure

Model Information	
Data Set	WORK.SORTTEMPTABLESORTED
Response Variable	TC_x
Number of Response Levels	2
Model	binary logit
Optimization Technique	Fisher's scoring

Number of Observations Read	1701
Number of Observations Used	1701

Response Profile		
Ordered Value	TC_x	Total Frequency
1	0	1386
2	1	315

Probability modeled is TC_x='1'.

Model Convergence Status
Convergence criterion (GCONV=1E-8) satisfied.

Model Fit Statistics		
Criterion	Intercept Only	Intercept and Covariates
AIC	1632.121	1583.251
SC	1637.560	1632.202
-2 Log L	1630.121	1565.251

Testing Global Null Hypothesis: BETA=0			
Test	Chi-Square	DF	Pr > ChiSq
Likelihood Ratio	64.8705	8	<.0001
Score	69.5324	8	<.0001
Wald	64.3396	8	<.0001

Logistic Regression Results

The LOGISTIC Procedure

Analysis of Maximum Likelihood Estimates					
Parameter	DF	Estimate	Standard Error	Wald Chi-Square	Pr > ChiSq
Intercept	1	-4.6005	0.8155	31.8254	<.0001
A1	1	-0.0123	0.0333	0.1360	0.7123
age	1	0.0610	0.0136	20.2375	<.0001
BMI	1	0.1138	0.0186	37.3240	<.0001
SBP	1	-0.00824	0.00701	1.3809	0.2400
D1_al	1	0.1682	0.1741	0.9332	0.3340
D2_al	1	0.3020	0.1977	2.3329	0.1267
D1_sm	1	0.1623	0.2807	0.3341	0.5632
D2_sm	1	-0.1061	0.2858	0.1378	0.7105

Odds Ratio Estimates			
Effect	Point Estimate	95% Wald Confidence Limits	
A1	0.988	0.925	1.054
age	1.063	1.035	1.092
BMI	1.121	1.080	1.162
SBP	0.992	0.978	1.006
D1_al	1.183	0.841	1.664
D2_al	1.353	0.918	1.993
D1_sm	1.176	0.678	2.039
D2_sm	0.899	0.514	1.575

Association of Predicted Probabilities and Observed Responses			
Percent Concordant	62.0	Somers' D	0.249
Percent Discordant	37.1	Gamma	0.251
Percent Tied	0.9	Tau-a	0.075
Pairs	436590	c	0.624

Logistic Regression Results

The LOGISTIC Procedure

Model Information	
Data Set	WORK.SORTTEMPTABLESORTED
Response Variable	HDL_x
Number of Response Levels	2
Model	binary logit
Optimization Technique	Fisher's scoring

Number of Observations Read	1701
Number of Observations Used	1701

Response Profile		
Ordered Value	HDL_x	Total Frequency
1	0	1410
2	1	291

Probability modeled is HDL_x='1'.

Model Convergence Status
Convergence criterion (GCONV=1E-8) satisfied.

Model Fit Statistics		
Criterion	Intercept Only	Intercept and Covariates
AIC	1558.714	1413.102
SC	1564.153	1462.053
-2 Log L	1556.714	1395.102

Testing Global Null Hypothesis: BETA=0			
Test	Chi-Square	DF	Pr > ChiSq
Likelihood Ratio	161.6122	8	<.0001
Score	184.5945	8	<.0001
Wald	145.2179	8	<.0001

Logistic Regression Results

The LOGISTIC Procedure

Analysis of Maximum Likelihood Estimates					
Parameter	DF	Estimate	Standard Error	Wald Chi-Square	Pr > ChiSq
Intercept	1	-6.1845	0.8625	51.4155	<.0001
A1	1	-0.0160	0.0354	0.2051	0.6506
age	1	0.00972	0.0145	0.4484	0.5031
BMI	1	0.2238	0.0203	121.8525	<.0001
SBP	1	-0.00555	0.00734	0.5717	0.4496
D1_al	1	0.2767	0.1829	2.2877	0.1304
D2_al	1	0.3902	0.2108	3.4281	0.0641
D1_sm	1	0.2405	0.3008	0.6395	0.4239
D2_sm	1	-0.0927	0.3046	0.0927	0.7607

Odds Ratio Estimates			
Effect	Point Estimate	95% Wald Confidence Limits	
A1	0.984	0.918	1.055
age	1.010	0.981	1.039
BMI	1.251	1.202	1.302
SBP	0.994	0.980	1.009
D1_al	1.319	0.921	1.887
D2_al	1.477	0.977	2.233
D1_sm	1.272	0.705	2.293
D2_sm	0.911	0.502	1.656

Association of Predicted Probabilities and Observed Responses			
Percent Concordant	72.1	Somers' D	0.449
Percent Discordant	27.2	Gamma	0.452
Percent Tied	0.6	Tau-a	0.127
Pairs	410310	c	0.725

Logistic Regression Results

The LOGISTIC Procedure

Model Information	
Data Set	WORK.SORTTEMPTABLESORTED
Response Variable	LDL_x
Number of Response Levels	2
Model	binary logit
Optimization Technique	Fisher's scoring

Number of Observations Read	1701
Number of Observations Used	1701

Response Profile		
Ordered Value	LDL_x	Total Frequency
1	0	1384
2	1	317

Probability modeled is LDL_x='1'.

Model Convergence Status
Convergence criterion (GCONV=1E-8) satisfied.

Model Fit Statistics		
Criterion	Intercept Only	Intercept and Covariates
AIC	1638.032	1543.367
SC	1643.471	1592.318
-2 Log L	1636.032	1525.367

Testing Global Null Hypothesis: BETA=0			
Test	Chi-Square	DF	Pr > ChiSq
Likelihood Ratio	110.6649	8	<.0001
Score	120.3992	8	<.0001
Wald	104.4097	8	<.0001

Logistic Regression Results

The LOGISTIC Procedure

Analysis of Maximum Likelihood Estimates					
Parameter	DF	Estimate	Standard Error	Wald Chi-Square	Pr > ChiSq
Intercept	1	-6.7427	0.8362	65.0163	<.0001
A1	1	-0.0146	0.0338	0.1868	0.6656
age	1	0.0844	0.0137	37.6718	<.0001
BMI	1	0.1341	0.0188	50.8150	<.0001
SBP	1	0.00231	0.00702	0.1087	0.7416
D1_al	1	0.0403	0.1818	0.0492	0.8245
D2_al	1	0.1640	0.2042	0.6453	0.4218
D1_sm	1	0.0947	0.2892	0.1071	0.7434
D2_sm	1	0.1761	0.2716	0.4204	0.5167

Odds Ratio Estimates			
Effect	Point Estimate	95% Wald Confidence Limits	
A1	0.986	0.922	1.053
age	1.088	1.059	1.118
BMI	1.144	1.102	1.186
SBP	1.002	0.989	1.016
D1_al	1.041	0.729	1.487
D2_al	1.178	0.790	1.758
D1_sm	1.099	0.624	1.938
D2_sm	1.193	0.700	2.031

Association of Predicted Probabilities and Observed Responses			
Percent Concordant	67.0	Somers' D	0.347
Percent Discordant	32.3	Gamma	0.349
Percent Tied	0.7	Tau-a	0.105
Pairs	438728	c	0.673

Logistic Regression Results

The LOGISTIC Procedure

Model Information	
Data Set	WORK.SORTTEMPTABLESORTED
Response Variable	TG_x
Number of Response Levels	2
Model	binary logit
Optimization Technique	Fisher's scoring

Number of Observations Read	1701
Number of Observations Used	1701

Response Profile		
Ordered Value	TG_x	Total Frequency
1	0	1614
2	1	87

Probability modeled is TG_x='1'.

Model Convergence Status
Convergence criterion (GCONV=1E-8) satisfied.

Model Fit Statistics		
Criterion	Intercept Only	Intercept and Covariates
AIC	688.785	588.930
SC	694.224	637.880
-2 Log L	686.785	570.930

Testing Global Null Hypothesis: BETA=0			
Test	Chi-Square	DF	Pr > ChiSq
Likelihood Ratio	115.8559	8	<.0001
Score	166.9578	8	<.0001
Wald	113.5482	8	<.0001

Logistic Regression Results

The LOGISTIC Procedure

Analysis of Maximum Likelihood Estimates					
Parameter	DF	Estimate	Standard Error	Wald Chi-Square	Pr > ChiSq
Intercept	1	-9.8325	1.3444	53.4904	<.0001
A1	1	-0.0337	0.0597	0.3188	0.5723
age	1	0.0414	0.0234	3.1164	0.0775
BMI	1	0.2507	0.0288	75.5536	<.0001
SBP	1	0.00221	0.0117	0.0355	0.8506
D1_al	1	0.0992	0.3237	0.0940	0.7591
D2_al	1	0.4297	0.3409	1.5892	0.2074
D1_sm	1	-0.2011	0.6177	0.1060	0.7447
D2_sm	1	0.3248	0.4069	0.6370	0.4248

Odds Ratio Estimates			
Effect	Point Estimate	95% Wald Confidence Limits	
A1	0.967	0.860	1.087
age	1.042	0.995	1.091
BMI	1.285	1.214	1.360
SBP	1.002	0.979	1.025
D1_al	1.104	0.586	2.083
D2_al	1.537	0.788	2.998
D1_sm	0.818	0.244	2.744
D2_sm	1.384	0.623	3.072

Association of Predicted Probabilities and Observed Responses			
Percent Concordant	77.3	Somers' D	0.561
Percent Discordant	21.2	Gamma	0.570
Percent Tied	1.5	Tau-a	0.054
Pairs	140418	c	0.781

Logistic Regression Results

The LOGISTIC Procedure

Model Information	
Data Set	WORK.SORTTEMPTABLESORTED
Response Variable	TC_x
Number of Response Levels	2
Model	binary logit
Optimization Technique	Fisher's scoring

Number of Observations Read	1701
Number of Observations Used	1701

Response Profile		
Ordered Value	TC_x	Total Frequency
1	0	1386
2	1	315

Probability modeled is TC_x='1'.

Model Convergence Status
Convergence criterion (GCONV=1E-8) satisfied.

Model Fit Statistics		
Criterion	Intercept Only	Intercept and Covariates
AIC	1632.121	1583.030
SC	1637.560	1631.981
-2 Log L	1630.121	1565.030

Testing Global Null Hypothesis: BETA=0			
Test	Chi-Square	DF	Pr > ChiSq
Likelihood Ratio	65.0912	8	<.0001
Score	69.7230	8	<.0001
Wald	64.4890	8	<.0001

Logistic Regression Results

The LOGISTIC Procedure

Analysis of Maximum Likelihood Estimates					
Parameter	DF	Estimate	Standard Error	Wald Chi-Square	Pr > ChiSq
Intercept	1	-4.6125	0.7834	34.6625	<.0001
A2	1	-0.0164	0.0275	0.3566	0.5504
age	1	0.0627	0.0139	20.2225	<.0001
BMI	1	0.1132	0.0187	36.7793	<.0001
SBP	1	-0.00800	0.00703	1.2948	0.2552
D1_al	1	0.1756	0.1745	1.0126	0.3143
D2_al	1	0.3055	0.1979	2.3820	0.1227
D1_sm	1	0.1569	0.2807	0.3124	0.5762
D2_sm	1	-0.1082	0.2860	0.1431	0.7052

Odds Ratio Estimates			
Effect	Point Estimate	95% Wald Confidence Limits	
A2	0.984	0.932	1.038
age	1.065	1.036	1.094
BMI	1.120	1.080	1.162
SBP	0.992	0.978	1.006
D1_al	1.192	0.847	1.678
D2_al	1.357	0.921	2.001
D1_sm	1.170	0.675	2.028
D2_sm	0.897	0.512	1.572

Association of Predicted Probabilities and Observed Responses			
Percent Concordant	61.9	Somers' D	0.248
Percent Discordant	37.2	Gamma	0.250
Percent Tied	0.9	Tau-a	0.075
Pairs	436590	c	0.624

Logistic Regression Results

The LOGISTIC Procedure

Model Information	
Data Set	WORK.SORTTEMPTABLESORTED
Response Variable	HDL_x
Number of Response Levels	2
Model	binary logit
Optimization Technique	Fisher's scoring

Number of Observations Read	1701
Number of Observations Used	1701

Response Profile

Ordered Value	HDL_x	Total Frequency
1	0	1410
2	1	291

Probability modeled is HDL_x='1'.

Model Convergence Status
Convergence criterion (GCONV=1E-8) satisfied.

Model Fit Statistics

Criterion	Intercept Only	Intercept and Covariates
AIC	1558.714	1408.350
SC	1564.153	1457.300
-2 Log L	1556.714	1390.350

Testing Global Null Hypothesis: BETA=0

Test	Chi-Square	DF	Pr > ChiSq
Likelihood Ratio	166.3647	8	<.0001
Score	188.5460	8	<.0001
Wald	148.3266	8	<.0001

Logistic Regression Results

The LOGISTIC Procedure

Analysis of Maximum Likelihood Estimates					
Parameter	DF	Estimate	Standard Error	Wald Chi-Square	Pr > ChiSq
Intercept	1	-5.9882	0.8281	52.2900	<.0001
A2	1	-0.0659	0.0297	4.9299	0.0264
age	1	0.0174	0.0150	1.3565	0.2441
BMI	1	0.2216	0.0203	119.1043	<.0001
SBP	1	-0.00435	0.00739	0.3460	0.5564
D1_al	1	0.3063	0.1838	2.7780	0.0956
D2_al	1	0.4053	0.2118	3.6607	0.0557
D1_sm	1	0.2159	0.3008	0.5149	0.4730
D2_sm	1	-0.1158	0.3060	0.1432	0.7051

Odds Ratio Estimates			
Effect	Point Estimate	95% Wald Confidence Limits	
A2	0.936	0.883	0.992
age	1.018	0.988	1.048
BMI	1.248	1.199	1.299
SBP	0.996	0.981	1.010
D1_al	1.358	0.948	1.947
D2_al	1.500	0.990	2.272
D1_sm	1.241	0.688	2.238
D2_sm	0.891	0.489	1.623

Association of Predicted Probabilities and Observed Responses			
Percent Concordant	72.6	Somers' D	0.459
Percent Discordant	26.8	Gamma	0.461
Percent Tied	0.6	Tau-a	0.130
Pairs	410310	c	0.729

Logistic Regression Results

The LOGISTIC Procedure

Model Information	
Data Set	WORK.SORTTEMPTABLESORTED
Response Variable	LDL_x
Number of Response Levels	2
Model	binary logit
Optimization Technique	Fisher's scoring

Number of Observations Read	1701
Number of Observations Used	1701

Response Profile		
Ordered Value	LDL_x	Total Frequency
1	0	1384
2	1	317

Probability modeled is LDL_x='1'.

Model Convergence Status
Convergence criterion (GCONV=1E-8) satisfied.

Model Fit Statistics		
Criterion	Intercept Only	Intercept and Covariates
AIC	1638.032	1542.880
SC	1643.471	1591.831
-2 Log L	1636.032	1524.880

Testing Global Null Hypothesis: BETA=0			
Test	Chi-Square	DF	Pr > ChiSq
Likelihood Ratio	111.1521	8	<.0001
Score	120.7951	8	<.0001
Wald	104.6354	8	<.0001

Logistic Regression Results

The LOGISTIC Procedure

Analysis of Maximum Likelihood Estimates					
Parameter	DF	Estimate	Standard Error	Wald Chi-Square	Pr > ChiSq
Intercept	1	-6.7460	0.8021	70.7380	<.0001
A2	1	-0.0230	0.0280	0.6736	0.4118
age	1	0.0868	0.0141	37.6478	<.0001
BMI	1	0.1333	0.0189	49.9841	<.0001
SBP	1	0.00271	0.00705	0.1479	0.7005
D1_al	1	0.0512	0.1822	0.0789	0.7788
D2_al	1	0.1687	0.2044	0.6815	0.4091
D1_sm	1	0.0868	0.2891	0.0902	0.7639
D2_sm	1	0.1729	0.2718	0.4047	0.5246

Odds Ratio Estimates			
Effect	Point Estimate	95% Wald Confidence Limits	
A2	0.977	0.925	1.032
age	1.091	1.061	1.121
BMI	1.143	1.101	1.186
SBP	1.003	0.989	1.017
D1_al	1.052	0.736	1.504
D2_al	1.184	0.793	1.767
D1_sm	1.091	0.619	1.922
D2_sm	1.189	0.698	2.025

Association of Predicted Probabilities and Observed Responses			
Percent Concordant	66.9	Somers' D	0.345
Percent Discordant	32.4	Gamma	0.347
Percent Tied	0.7	Tau-a	0.105
Pairs	438728	c	0.672

Logistic Regression Results

The LOGISTIC Procedure

Model Information	
Data Set	WORK.SORTTEMPTABLESORTED
Response Variable	TG_x
Number of Response Levels	2
Model	binary logit
Optimization Technique	Fisher's scoring

Number of Observations Read	1701
Number of Observations Used	1701

Response Profile		
Ordered Value	TG_x	Total Frequency
1	0	1614
2	1	87

Probability modeled is TG_x='1'.

Model Convergence Status
Convergence criterion (GCONV=1E-8) satisfied.

Model Fit Statistics		
Criterion	Intercept Only	Intercept and Covariates
AIC	688.785	586.420
SC	694.224	635.371
-2 Log L	686.785	568.420

Testing Global Null Hypothesis: BETA=0			
Test	Chi-Square	DF	Pr > ChiSq
Likelihood Ratio	118.3653	8	<.0001
Score	168.7480	8	<.0001
Wald	114.5695	8	<.0001

Logistic Regression Results

The LOGISTIC Procedure

Analysis of Maximum Likelihood Estimates					
Parameter	DF	Estimate	Standard Error	Wald Chi-Square	Pr > ChiSq
Intercept	1	-9.7147	1.3049	55.4261	<.0001
A2	1	-0.0859	0.0513	2.8112	0.0936
age	1	0.0512	0.0243	4.4308	0.0353
BMI	1	0.2486	0.0289	74.0467	<.0001
SBP	1	0.00381	0.0119	0.1028	0.7485
D1_al	1	0.1289	0.3256	0.1566	0.6923
D2_al	1	0.4288	0.3419	1.5729	0.2098
D1_sm	1	-0.2201	0.6162	0.1276	0.7210
D2_sm	1	0.2886	0.4096	0.4964	0.4811

Odds Ratio Estimates			
Effect	Point Estimate	95% Wald Confidence Limits	
A2	0.918	0.830	1.015
age	1.053	1.004	1.104
BMI	1.282	1.212	1.357
SBP	1.004	0.981	1.027
D1_al	1.138	0.601	2.153
D2_al	1.535	0.786	3.001
D1_sm	0.802	0.240	2.685
D2_sm	1.335	0.598	2.978

Association of Predicted Probabilities and Observed Responses			
Percent Concordant	77.7	Somers' D	0.568
Percent Discordant	20.8	Gamma	0.577
Percent Tied	1.5	Tau-a	0.055
Pairs	140418	c	0.784

Logistic Regression Results

The LOGISTIC Procedure

Model Information	
Data Set	WORK.SORTTEMPTABLESORTED
Response Variable	TC_x
Number of Response Levels	2
Model	binary logit
Optimization Technique	Fisher's scoring

Number of Observations Read	1701
Number of Observations Used	1701

Response Profile		
Ordered Value	TC_x	Total Frequency
1	0	1386
2	1	315

Probability modeled is TC_x='1'.

Model Convergence Status
Convergence criterion (GCONV=1E-8) satisfied.

Model Fit Statistics		
Criterion	Intercept Only	Intercept and Covariates
AIC	1632.121	1582.996
SC	1637.560	1631.947
-2 Log L	1630.121	1564.996

Testing Global Null Hypothesis: BETA=0			
Test	Chi-Square	DF	Pr > ChiSq
Likelihood Ratio	65.1251	8	<.0001
Score	69.6595	8	<.0001
Wald	64.4585	8	<.0001

Logistic Regression Results

The LOGISTIC Procedure

Analysis of Maximum Likelihood Estimates					
Parameter	DF	Estimate	Standard Error	Wald Chi-Square	Pr > ChiSq
Intercept	1	-4.4592	0.8605	26.8520	<.0001
A3	1	-0.0222	0.0356	0.3913	0.5316
age	1	0.0607	0.0135	20.0828	<.0001
BMI	1	0.1145	0.0187	37.6723	<.0001
SBP	1	-0.00835	0.00701	1.4210	0.2332
D1_al	1	0.1733	0.1742	0.9895	0.3199
D2_al	1	0.3028	0.1977	2.3462	0.1256
D1_sm	1	0.1641	0.2807	0.3417	0.5588
D2_sm	1	-0.1081	0.2858	0.1431	0.7052

Odds Ratio Estimates			
Effect	Point Estimate	95% Wald Confidence Limits	
A3	0.978	0.912	1.049
age	1.063	1.035	1.091
BMI	1.121	1.081	1.163
SBP	0.992	0.978	1.005
D1_al	1.189	0.845	1.673
D2_al	1.354	0.919	1.994
D1_sm	1.178	0.680	2.043
D2_sm	0.898	0.513	1.572

Association of Predicted Probabilities and Observed Responses			
Percent Concordant	62.2	Somers' D	0.253
Percent Discordant	36.9	Gamma	0.255
Percent Tied	0.9	Tau-a	0.076
Pairs	436590	c	0.626

Logistic Regression Results

The LOGISTIC Procedure

Model Information	
Data Set	WORK.SORTTEMPTABLESORTED
Response Variable	HDL_x
Number of Response Levels	2
Model	binary logit
Optimization Technique	Fisher's scoring

Number of Observations Read	1701
Number of Observations Used	1701

Response Profile		
Ordered Value	HDL_x	Total Frequency
1	0	1410
2	1	291

Probability modeled is HDL_x='1'.

Model Convergence Status
Convergence criterion (GCONV=1E-8) satisfied.

Model Fit Statistics		
Criterion	Intercept Only	Intercept and Covariates
AIC	1558.714	1411.676
SC	1564.153	1460.627
-2 Log L	1556.714	1393.676

Testing Global Null Hypothesis: BETA=0			
Test	Chi-Square	DF	Pr > ChiSq
Likelihood Ratio	163.0379	8	<.0001
Score	185.5534	8	<.0001
Wald	146.2731	8	<.0001

Logistic Regression Results

The LOGISTIC Procedure

Analysis of Maximum Likelihood Estimates					
Parameter	DF	Estimate	Standard Error	Wald Chi-Square	Pr > ChiSq
Intercept	1	-5.7876	0.9112	40.3445	<.0001
A3	1	-0.0484	0.0379	1.6335	0.2012
age	1	0.00950	0.0145	0.4290	0.5125
BMI	1	0.2253	0.0203	123.1245	<.0001
SBP	1	-0.00585	0.00735	0.6320	0.4266
D1_al	1	0.2891	0.1831	2.4916	0.1145
D2_al	1	0.3914	0.2107	3.4508	0.0632
D1_sm	1	0.2418	0.3015	0.6431	0.4226
D2_sm	1	-0.1058	0.3057	0.1198	0.7292

Odds Ratio Estimates			
Effect	Point Estimate	95% Wald Confidence Limits	
A3	0.953	0.885	1.026
age	1.010	0.981	1.039
BMI	1.253	1.204	1.304
SBP	0.994	0.980	1.009
D1_al	1.335	0.933	1.912
D2_al	1.479	0.979	2.235
D1_sm	1.273	0.705	2.299
D2_sm	0.900	0.494	1.638

Association of Predicted Probabilities and Observed Responses			
Percent Concordant	72.3	Somers' D	0.452
Percent Discordant	27.1	Gamma	0.455
Percent Tied	0.6	Tau-a	0.128
Pairs	410310	c	0.726

Logistic Regression Results

The LOGISTIC Procedure

Model Information	
Data Set	WORK.SORTTEMPTABLESORTED
Response Variable	LDL_x
Number of Response Levels	2
Model	binary logit
Optimization Technique	Fisher's scoring

Number of Observations Read	1701
Number of Observations Used	1701

Response Profile		
Ordered Value	LDL_x	Total Frequency
1	0	1384
2	1	317

Probability modeled is LDL_x='1'.

Model Convergence Status
Convergence criterion (GCONV=1E-8) satisfied.

Model Fit Statistics		
Criterion	Intercept Only	Intercept and Covariates
AIC	1638.032	1543.318
SC	1643.471	1592.269
-2 Log L	1636.032	1525.318

Testing Global Null Hypothesis: BETA=0			
Test	Chi-Square	DF	Pr > ChiSq
Likelihood Ratio	110.7142	8	<.0001
Score	120.3502	8	<.0001
Wald	104.2827	8	<.0001

Logistic Regression Results

The LOGISTIC Procedure

Analysis of Maximum Likelihood Estimates					
Parameter	DF	Estimate	Standard Error	Wald Chi-Square	Pr > ChiSq
Intercept	1	-6.6758	0.8782	57.7820	<.0001
A3	1	-0.0175	0.0360	0.2362	0.6269
age	1	0.0840	0.0137	37.5047	<.0001
BMI	1	0.1347	0.0188	51.1275	<.0001
SBP	1	0.00221	0.00702	0.0988	0.7532
D1_al	1	0.0451	0.1818	0.0616	0.8040
D2_al	1	0.1647	0.2041	0.6512	0.4197
D1_sm	1	0.0963	0.2892	0.1109	0.7392
D2_sm	1	0.1768	0.2715	0.4241	0.5149

Odds Ratio Estimates			
Effect	Point Estimate	95% Wald Confidence Limits	
A3	0.983	0.916	1.054
age	1.088	1.059	1.117
BMI	1.144	1.103	1.187
SBP	1.002	0.989	1.016
D1_al	1.046	0.733	1.494
D2_al	1.179	0.790	1.759
D1_sm	1.101	0.625	1.941
D2_sm	1.193	0.701	2.032

Association of Predicted Probabilities and Observed Responses			
Percent Concordant	67.1	Somers' D	0.349
Percent Discordant	32.2	Gamma	0.351
Percent Tied	0.7	Tau-a	0.106
Pairs	438728	c	0.674

Logistic Regression Results

The LOGISTIC Procedure

Model Information	
Data Set	WORK.SORTTEMPTABLESORTED
Response Variable	TG_x
Number of Response Levels	2
Model	binary logit
Optimization Technique	Fisher's scoring

Number of Observations Read	1701
Number of Observations Used	1701

Response Profile		
Ordered Value	TG_x	Total Frequency
1	0	1614
2	1	87

Probability modeled is TG_x='1'.

Model Convergence Status
Convergence criterion (GCONV=1E-8) satisfied.

Model Fit Statistics		
Criterion	Intercept Only	Intercept and Covariates
AIC	688.785	588.463
SC	694.224	637.414
-2 Log L	686.785	570.463

Testing Global Null Hypothesis: BETA=0			
Test	Chi-Square	DF	Pr > ChiSq
Likelihood Ratio	116.3220	8	<.0001
Score	167.0560	8	<.0001
Wald	113.4129	8	<.0001

Logistic Regression Results

The LOGISTIC Procedure

Analysis of Maximum Likelihood Estimates					
Parameter	DF	Estimate	Standard Error	Wald Chi-Square	Pr > ChiSq
Intercept	1	-9.5040	1.4266	44.3852	<.0001
A3	1	-0.0562	0.0633	0.7902	0.3740
age	1	0.0415	0.0234	3.1428	0.0763
BMI	1	0.2533	0.0292	75.4682	<.0001
SBP	1	0.00152	0.0117	0.0167	0.8971
D1_al	1	0.1178	0.3242	0.1321	0.7163
D2_al	1	0.4322	0.3404	1.6125	0.2041
D1_sm	1	-0.2119	0.6194	0.1171	0.7322
D2_sm	1	0.3060	0.4088	0.5603	0.4541

Odds Ratio Estimates			
Effect	Point Estimate	95% Wald Confidence Limits	
A3	0.945	0.835	1.070
age	1.042	0.996	1.091
BMI	1.288	1.217	1.364
SBP	1.002	0.979	1.025
D1_al	1.125	0.596	2.124
D2_al	1.541	0.791	3.002
D1_sm	0.809	0.240	2.724
D2_sm	1.358	0.609	3.026

Association of Predicted Probabilities and Observed Responses			
Percent Concordant	77.6	Somers' D	0.568
Percent Discordant	20.9	Gamma	0.576
Percent Tied	1.5	Tau-a	0.055
Pairs	140418	c	0.784

Logistic Regression Results

The LOGISTIC Procedure

Model Information	
Data Set	WORK.SORTTEMPTABLESORTED
Response Variable	TC_x
Number of Response Levels	2
Model	binary logit
Optimization Technique	Fisher's scoring

Number of Observations Read	1701
Number of Observations Used	1701

Response Profile		
Ordered Value	TC_x	Total Frequency
1	0	1386
2	1	315

Probability modeled is TC_x='1'.

Model Convergence Status
Convergence criterion (GCONV=1E-8) satisfied.

Model Fit Statistics		
Criterion	Intercept Only	Intercept and Covariates
AIC	1632.121	1583.286
SC	1637.560	1632.236
-2 Log L	1630.121	1565.286

Testing Global Null Hypothesis: BETA=0			
Test	Chi-Square	DF	Pr > ChiSq
Likelihood Ratio	64.8357	8	<.0001
Score	69.5642	8	<.0001
Wald	64.3045	8	<.0001

Logistic Regression Results

The LOGISTIC Procedure

Analysis of Maximum Likelihood Estimates					
Parameter	DF	Estimate	Standard Error	Wald Chi-Square	Pr > ChiSq
Intercept	1	-4.7797	0.8083	34.9656	<.0001
A4	1	0.0107	0.0336	0.1012	0.7504
age	1	0.0603	0.0136	19.6468	<.0001
BMI	1	0.1141	0.0186	37.5025	<.0001
SBP	1	-0.00841	0.00701	1.4393	0.2303
D1_al	1	0.1657	0.1744	0.9027	0.3421
D2_al	1	0.2993	0.1979	2.2872	0.1304
D1_sm	1	0.1658	0.2809	0.3487	0.5548
D2_sm	1	-0.0956	0.2863	0.1114	0.7386

Odds Ratio Estimates			
Effect	Point Estimate	95% Wald Confidence Limits	
A4	1.011	0.946	1.079
age	1.062	1.034	1.091
BMI	1.121	1.081	1.163
SBP	0.992	0.978	1.005
D1_al	1.180	0.839	1.661
D2_al	1.349	0.915	1.988
D1_sm	1.180	0.681	2.047
D2_sm	0.909	0.519	1.593

Association of Predicted Probabilities and Observed Responses			
Percent Concordant	62.0	Somers' D	0.249
Percent Discordant	37.1	Gamma	0.251
Percent Tied	0.9	Tau-a	0.075
Pairs	436590	c	0.624

Logistic Regression Results

The LOGISTIC Procedure

Model Information	
Data Set	WORK.SORTTEMPTABLESORTED
Response Variable	HDL_x
Number of Response Levels	2
Model	binary logit
Optimization Technique	Fisher's scoring

Number of Observations Read	1701
Number of Observations Used	1701

Response Profile		
Ordered Value	HDL_x	Total Frequency
1	0	1410
2	1	291

Probability modeled is HDL_x='1'.

Model Convergence Status
Convergence criterion (GCONV=1E-8) satisfied.

Model Fit Statistics		
Criterion	Intercept Only	Intercept and Covariates
AIC	1558.714	1412.194
SC	1564.153	1461.144
-2 Log L	1556.714	1394.194

Testing Global Null Hypothesis: BETA=0			
Test	Chi-Square	DF	Pr > ChiSq
Likelihood Ratio	162.5207	8	<.0001
Score	185.5152	8	<.0001
Wald	145.2862	8	<.0001

Logistic Regression Results

The LOGISTIC Procedure

Analysis of Maximum Likelihood Estimates					
Parameter	DF	Estimate	Standard Error	Wald Chi-Square	Pr > ChiSq
Intercept	1	-6.0401	0.8543	49.9855	<.0001
A4	1	-0.0380	0.0361	1.1102	0.2920
age	1	0.0110	0.0146	0.5660	0.4519
BMI	1	0.2237	0.0203	121.4577	<.0001
SBP	1	-0.00544	0.00735	0.5470	0.4595
D1_al	1	0.2910	0.1835	2.5162	0.1127
D2_al	1	0.3986	0.2108	3.5760	0.0586
D1_sm	1	0.2296	0.3013	0.5808	0.4460
D2_sm	1	-0.1069	0.3053	0.1225	0.7263

Odds Ratio Estimates			
Effect	Point Estimate	95% Wald Confidence Limits	
A4	0.963	0.897	1.033
age	1.011	0.983	1.040
BMI	1.251	1.202	1.301
SBP	0.995	0.980	1.009
D1_al	1.338	0.934	1.917
D2_al	1.490	0.986	2.252
D1_sm	1.258	0.697	2.271
D2_sm	0.899	0.494	1.635

Association of Predicted Probabilities and Observed Responses			
Percent Concordant	72.2	Somers' D	0.450
Percent Discordant	27.2	Gamma	0.453
Percent Tied	0.6	Tau-a	0.128
Pairs	410310	c	0.725

Logistic Regression Results

The LOGISTIC Procedure

Model Information	
Data Set	WORK.SORTTEMPTABLESORTED
Response Variable	LDL_x
Number of Response Levels	2
Model	binary logit
Optimization Technique	Fisher's scoring

Number of Observations Read	1701
Number of Observations Used	1701

Response Profile

Ordered Value	LDL_x	Total Frequency
1	0	1384
2	1	317

Probability modeled is LDL_x='1'.

Model Convergence Status
Convergence criterion (GCONV=1E-8) satisfied.

Model Fit Statistics

Criterion	Intercept Only	Intercept and Covariates
AIC	1638.032	1543.192
SC	1643.471	1592.143
-2 Log L	1636.032	1525.192

Testing Global Null Hypothesis: BETA=0

Test	Chi-Square	DF	Pr > ChiSq
Likelihood Ratio	110.8403	8	<.0001
Score	120.7544	8	<.0001
Wald	104.6178	8	<.0001

Logistic Regression Results

The LOGISTIC Procedure

Analysis of Maximum Likelihood Estimates					
Parameter	DF	Estimate	Standard Error	Wald Chi-Square	Pr > ChiSq
Intercept	1	-7.0126	0.8271	71.8838	<.0001
A4	1	0.0205	0.0341	0.3621	0.5473
age	1	0.0832	0.0138	36.3451	<.0001
BMI	1	0.1347	0.0188	51.1123	<.0001
SBP	1	0.00204	0.00702	0.0845	0.7713
D1_al	1	0.0351	0.1820	0.0373	0.8469
D2_al	1	0.1591	0.2045	0.6054	0.4365
D1_sm	1	0.1010	0.2893	0.1219	0.7270
D2_sm	1	0.1932	0.2720	0.5046	0.4775

Odds Ratio Estimates			
Effect	Point Estimate	95% Wald Confidence Limits	
A4	1.021	0.955	1.091
age	1.087	1.058	1.116
BMI	1.144	1.103	1.187
SBP	1.002	0.988	1.016
D1_al	1.036	0.725	1.480
D2_al	1.172	0.785	1.750
D1_sm	1.106	0.627	1.951
D2_sm	1.213	0.712	2.067

Association of Predicted Probabilities and Observed Responses			
Percent Concordant	67.2	Somers' D	0.350
Percent Discordant	32.2	Gamma	0.352
Percent Tied	0.7	Tau-a	0.106
Pairs	438728	c	0.675

Logistic Regression Results

The LOGISTIC Procedure

Model Information	
Data Set	WORK.SORTTEMPTABLESORTED
Response Variable	TG_x
Number of Response Levels	2
Model	binary logit
Optimization Technique	Fisher's scoring

Number of Observations Read	1701
Number of Observations Used	1701

Response Profile		
Ordered Value	TG_x	Total Frequency
1	0	1614
2	1	87

Probability modeled is TG_x='1'.

Model Convergence Status
Convergence criterion (GCONV=1E-8) satisfied.

Model Fit Statistics		
Criterion	Intercept Only	Intercept and Covariates
AIC	688.785	587.357
SC	694.224	636.307
-2 Log L	686.785	569.357

Testing Global Null Hypothesis: BETA=0			
Test	Chi-Square	DF	Pr > ChiSq
Likelihood Ratio	117.4288	8	<.0001
Score	168.5478	8	<.0001
Wald	114.1176	8	<.0001

Logistic Regression Results

The LOGISTIC Procedure

Analysis of Maximum Likelihood Estimates					
Parameter	DF	Estimate	Standard Error	Wald Chi-Square	Pr > ChiSq
Intercept	1	-10.7193	1.3656	61.6179	<.0001
A4	1	0.0834	0.0607	1.8866	0.1696
age	1	0.0367	0.0235	2.4508	0.1175
BMI	1	0.2535	0.0291	75.7487	<.0001
SBP	1	0.00111	0.0118	0.0089	0.9247
D1_al	1	0.0796	0.3238	0.0605	0.8057
D2_al	1	0.3943	0.3429	1.3220	0.2502
D1_sm	1	-0.1650	0.6171	0.0715	0.7892
D2_sm	1	0.3809	0.4086	0.8689	0.3513

Odds Ratio Estimates			
Effect	Point Estimate	95% Wald Confidence Limits	
A4	1.087	0.965	1.224
age	1.037	0.991	1.086
BMI	1.289	1.217	1.364
SBP	1.001	0.978	1.024
D1_al	1.083	0.574	2.043
D2_al	1.483	0.757	2.905
D1_sm	0.848	0.253	2.842
D2_sm	1.464	0.657	3.260

Association of Predicted Probabilities and Observed Responses			
Percent Concordant	77.9	Somers' D	0.573
Percent Discordant	20.6	Gamma	0.582
Percent Tied	1.6	Tau-a	0.056
Pairs	140418	c	0.787

Logistic Regression Results

The LOGISTIC Procedure

Model Information	
Data Set	WORK.SORTTEMPTABLESORTED
Response Variable	TC_x
Number of Response Levels	2
Model	binary logit
Optimization Technique	Fisher's scoring

Number of Observations Read	1701
Number of Observations Used	1701

Response Profile		
Ordered Value	TC_x	Total Frequency
1	0	1386
2	1	315

Probability modeled is TC_x='1'.

Model Convergence Status
Convergence criterion (GCONV=1E-8) satisfied.

Model Fit Statistics		
Criterion	Intercept Only	Intercept and Covariates
AIC	1632.121	1581.011
SC	1637.560	1629.962
-2 Log L	1630.121	1563.011

Testing Global Null Hypothesis: BETA=0			
Test	Chi-Square	DF	Pr > ChiSq
Likelihood Ratio	67.1107	8	<.0001
Score	71.4699	8	<.0001
Wald	66.0767	8	<.0001

Logistic Regression Results

The LOGISTIC Procedure

Analysis of Maximum Likelihood Estimates					
Parameter	DF	Estimate	Standard Error	Wald Chi-Square	Pr > ChiSq
Intercept	1	-4.2203	0.8288	25.9284	<.0001
A5	1	-0.0526	0.0341	2.3863	0.1224
age	1	0.0629	0.0136	21.2427	<.0001
BMI	1	0.1130	0.0187	36.6990	<.0001
SBP	1	-0.00804	0.00701	1.3149	0.2515
D1_al	1	0.1812	0.1744	1.0805	0.2986
D2_al	1	0.3151	0.1981	2.5303	0.1117
D1_sm	1	0.1478	0.2808	0.2771	0.5986
D2_sm	1	-0.1492	0.2882	0.2679	0.6048

Odds Ratio Estimates			
Effect	Point Estimate	95% Wald Confidence Limits	
A5	0.949	0.887	1.014
age	1.065	1.037	1.094
BMI	1.120	1.079	1.161
SBP	0.992	0.978	1.006
D1_al	1.199	0.852	1.687
D2_al	1.370	0.929	2.020
D1_sm	1.159	0.669	2.010
D2_sm	0.861	0.490	1.516

Association of Predicted Probabilities and Observed Responses			
Percent Concordant	62.5	Somers' D	0.258
Percent Discordant	36.6	Gamma	0.261
Percent Tied	0.9	Tau-a	0.078
Pairs	436590	c	0.629

Logistic Regression Results

The LOGISTIC Procedure

Model Information	
Data Set	WORK.SORTTEMPTABLESORTED
Response Variable	HDL_x
Number of Response Levels	2
Model	binary logit
Optimization Technique	Fisher's scoring

Number of Observations Read	1701
Number of Observations Used	1701

Response Profile		
Ordered Value	HDL_x	Total Frequency
1	0	1410
2	1	291

Probability modeled is HDL_x='1'.

Model Convergence Status
Convergence criterion (GCONV=1E-8) satisfied.

Model Fit Statistics		
Criterion	Intercept Only	Intercept and Covariates
AIC	1558.714	1413.286
SC	1564.153	1462.237
-2 Log L	1556.714	1395.286

Testing Global Null Hypothesis: BETA=0			
Test	Chi-Square	DF	Pr > ChiSq
Likelihood Ratio	161.4280	8	<.0001
Score	184.4978	8	<.0001
Wald	144.8706	8	<.0001

Logistic Regression Results

The LOGISTIC Procedure

Analysis of Maximum Likelihood Estimates					
Parameter	DF	Estimate	Standard Error	Wald Chi-Square	Pr > ChiSq
Intercept	1	-6.2674	0.8802	50.7034	<.0001
A5	1	-0.00523	0.0362	0.0208	0.8853
age	1	0.00956	0.0146	0.4299	0.5121
BMI	1	0.2240	0.0203	121.8217	<.0001
SBP	1	-0.00566	0.00735	0.5946	0.4406
D1_al	1	0.2794	0.1831	2.3275	0.1271
D2_al	1	0.3905	0.2109	3.4271	0.0641
D1_sm	1	0.2393	0.3010	0.6322	0.4266
D2_sm	1	-0.0926	0.3067	0.0912	0.7626

Odds Ratio Estimates			
Effect	Point Estimate	95% Wald Confidence Limits	
A5	0.995	0.927	1.068
age	1.010	0.981	1.039
BMI	1.251	1.202	1.302
SBP	0.994	0.980	1.009
D1_al	1.322	0.924	1.893
D2_al	1.478	0.977	2.234
D1_sm	1.270	0.704	2.292
D2_sm	0.912	0.500	1.663

Association of Predicted Probabilities and Observed Responses			
Percent Concordant	72.1	Somers' D	0.449
Percent Discordant	27.3	Gamma	0.452
Percent Tied	0.6	Tau-a	0.127
Pairs	410310	c	0.724

Logistic Regression Results

The LOGISTIC Procedure

Model Information	
Data Set	WORK.SORTTEMPTABLESORTED
Response Variable	LDL_x
Number of Response Levels	2
Model	binary logit
Optimization Technique	Fisher's scoring

Number of Observations Read	1701
Number of Observations Used	1701

Response Profile		
Ordered Value	LDL_x	Total Frequency
1	0	1384
2	1	317

Probability modeled is LDL_x='1'.

Model Convergence Status
Convergence criterion (GCONV=1E-8) satisfied.

Model Fit Statistics		
Criterion	Intercept Only	Intercept and Covariates
AIC	1638.032	1543.310
SC	1643.471	1592.260
-2 Log L	1636.032	1525.310

Testing Global Null Hypothesis: BETA=0			
Test	Chi-Square	DF	Pr > ChiSq
Likelihood Ratio	110.7227	8	<.0001
Score	120.4373	8	<.0001
Wald	104.2481	8	<.0001

Logistic Regression Results

The LOGISTIC Procedure

Analysis of Maximum Likelihood Estimates					
Parameter	DF	Estimate	Standard Error	Wald Chi-Square	Pr > ChiSq
Intercept	1	-6.7057	0.8507	62.1395	<.0001
A5	1	-0.0172	0.0347	0.2449	0.6207
age	1	0.0847	0.0138	37.6885	<.0001
BMI	1	0.1340	0.0188	50.6036	<.0001
SBP	1	0.00231	0.00702	0.1080	0.7425
D1_al	1	0.0456	0.1819	0.0628	0.8021
D2_al	1	0.1681	0.2043	0.6770	0.4106
D1_sm	1	0.0900	0.2893	0.0968	0.7557
D2_sm	1	0.1665	0.2733	0.3712	0.5424

Odds Ratio Estimates			
Effect	Point Estimate	95% Wald Confidence Limits	
A5	0.983	0.918	1.052
age	1.088	1.059	1.118
BMI	1.143	1.102	1.186
SBP	1.002	0.989	1.016
D1_al	1.047	0.733	1.495
D2_al	1.183	0.793	1.766
D1_sm	1.094	0.621	1.929
D2_sm	1.181	0.691	2.018

Association of Predicted Probabilities and Observed Responses			
Percent Concordant	67.0	Somers' D	0.348
Percent Discordant	32.3	Gamma	0.350
Percent Tied	0.7	Tau-a	0.106
Pairs	438728	c	0.674

Logistic Regression Results

The LOGISTIC Procedure

Model Information	
Data Set	WORK.SORTTEMPTABLESORTED
Response Variable	TG_x
Number of Response Levels	2
Model	binary logit
Optimization Technique	Fisher's scoring

Number of Observations Read	1701
Number of Observations Used	1701

Response Profile		
Ordered Value	TG_x	Total Frequency
1	0	1614
2	1	87

Probability modeled is TG_x='1'.

Model Convergence Status
Convergence criterion (GCONV=1E-8) satisfied.

Model Fit Statistics		
Criterion	Intercept Only	Intercept and Covariates
AIC	688.785	588.631
SC	694.224	637.582
-2 Log L	686.785	570.631

Testing Global Null Hypothesis: BETA=0			
Test	Chi-Square	DF	Pr > ChiSq
Likelihood Ratio	116.1542	8	<.0001
Score	167.5794	8	<.0001
Wald	113.1282	8	<.0001

Logistic Regression Results

The LOGISTIC Procedure

Analysis of Maximum Likelihood Estimates					
Parameter	DF	Estimate	Standard Error	Wald Chi-Square	Pr > ChiSq
Intercept	1	-10.5458	1.4167	55.4084	<.0001
A5	1	0.0493	0.0631	0.6100	0.4348
age	1	0.0378	0.0236	2.5819	0.1081
BMI	1	0.2515	0.0290	75.4191	<.0001
SBP	1	0.00182	0.0117	0.0241	0.8767
D1_al	1	0.0861	0.3248	0.0702	0.7910
D2_al	1	0.4181	0.3417	1.4978	0.2210
D1_sm	1	-0.1847	0.6193	0.0890	0.7655
D2_sm	1	0.3954	0.4132	0.9157	0.3386

Odds Ratio Estimates			
Effect	Point Estimate	95% Wald Confidence Limits	
A5	1.051	0.928	1.189
age	1.039	0.992	1.088
BMI	1.286	1.215	1.361
SBP	1.002	0.979	1.025
D1_al	1.090	0.577	2.060
D2_al	1.519	0.778	2.968
D1_sm	0.831	0.247	2.798
D2_sm	1.485	0.661	3.338

Association of Predicted Probabilities and Observed Responses			
Percent Concordant	77.5	Somers' D	0.566
Percent Discordant	20.9	Gamma	0.575
Percent Tied	1.6	Tau-a	0.055
Pairs	140418	c	0.783

Logistic Regression Results

The LOGISTIC Procedure

Model Information	
Data Set	WORK.SORTTEMPTABLESORTED
Response Variable	TC_x
Number of Response Levels	2
Model	binary logit
Optimization Technique	Fisher's scoring

Number of Observations Read	1701
Number of Observations Used	1701

Response Profile		
Ordered Value	TC_x	Total Frequency
1	0	1386
2	1	315

Probability modeled is TC_x='1'.

Model Convergence Status
Convergence criterion (GCONV=1E-8) satisfied.

Model Fit Statistics		
Criterion	Intercept Only	Intercept and Covariates
AIC	1632.121	1581.921
SC	1637.560	1630.872
-2 Log L	1630.121	1563.921

Testing Global Null Hypothesis: BETA=0			
Test	Chi-Square	DF	Pr > ChiSq
Likelihood Ratio	66.2001	8	<.0001
Score	70.7110	8	<.0001
Wald	65.3284	8	<.0001

Logistic Regression Results

The LOGISTIC Procedure

Analysis of Maximum Likelihood Estimates					
Parameter	DF	Estimate	Standard Error	Wald Chi-Square	Pr > ChiSq
Intercept	1	-4.3432	0.8227	27.8673	<.0001
A6	1	-0.0346	0.0285	1.4693	0.2255
age	1	0.0604	0.0136	19.8725	<.0001
BMI	1	0.1136	0.0187	37.1125	<.0001
SBP	1	-0.00827	0.00700	1.3941	0.2377
D1_al	1	0.1845	0.1745	1.1169	0.2906
D2_al	1	0.3085	0.1979	2.4293	0.1191
D1_sm	1	0.1549	0.2810	0.3039	0.5815
D2_sm	1	-0.1102	0.2861	0.1484	0.7001

Odds Ratio Estimates			
Effect	Point Estimate	95% Wald Confidence Limits	
A6	0.966	0.914	1.022
age	1.062	1.034	1.091
BMI	1.120	1.080	1.162
SBP	0.992	0.978	1.005
D1_al	1.203	0.854	1.693
D2_al	1.361	0.924	2.006
D1_sm	1.168	0.673	2.025
D2_sm	0.896	0.511	1.569

Association of Predicted Probabilities and Observed Responses			
Percent Concordant	62.5	Somers' D	0.258
Percent Discordant	36.7	Gamma	0.260
Percent Tied	0.9	Tau-a	0.078
Pairs	436590	c	0.629

Logistic Regression Results

The LOGISTIC Procedure

Model Information	
Data Set	WORK.SORTTEMPTABLESORTED
Response Variable	HDL_x
Number of Response Levels	2
Model	binary logit
Optimization Technique	Fisher's scoring

Number of Observations Read	1701
Number of Observations Used	1701

Response Profile		
Ordered Value	HDL_x	Total Frequency
1	0	1410
2	1	291

Probability modeled is HDL_x='1'.

Model Convergence Status
Convergence criterion (GCONV=1E-8) satisfied.

Model Fit Statistics		
Criterion	Intercept Only	Intercept and Covariates
AIC	1558.714	1413.185
SC	1564.153	1462.135
-2 Log L	1556.714	1395.185

Testing Global Null Hypothesis: BETA=0			
Test	Chi-Square	DF	Pr > ChiSq
Likelihood Ratio	161.5297	8	<.0001
Score	184.5512	8	<.0001
Wald	144.9560	8	<.0001

Logistic Regression Results

The LOGISTIC Procedure

Analysis of Maximum Likelihood Estimates					
Parameter	DF	Estimate	Standard Error	Wald Chi-Square	Pr > ChiSq
Intercept	1	-6.2023	0.8753	50.2069	<.0001
A6	1	-0.0107	0.0306	0.1225	0.7263
age	1	0.00926	0.0145	0.4076	0.5232
BMI	1	0.2240	0.0203	122.0563	<.0001
SBP	1	-0.00570	0.00734	0.6027	0.4375
D1_al	1	0.2834	0.1835	2.3843	0.1226
D2_al	1	0.3919	0.2109	3.4536	0.0631
D1_sm	1	0.2384	0.3009	0.6279	0.4281
D2_sm	1	-0.0911	0.3049	0.0892	0.7653

Odds Ratio Estimates			
Effect	Point Estimate	95% Wald Confidence Limits	
A6	0.989	0.932	1.050
age	1.009	0.981	1.038
BMI	1.251	1.202	1.302
SBP	0.994	0.980	1.009
D1_al	1.328	0.927	1.902
D2_al	1.480	0.979	2.237
D1_sm	1.269	0.704	2.289
D2_sm	0.913	0.502	1.660

Association of Predicted Probabilities and Observed Responses			
Percent Concordant	72.2	Somers' D	0.450
Percent Discordant	27.2	Gamma	0.452
Percent Tied	0.6	Tau-a	0.128
Pairs	410310	c	0.725

Logistic Regression Results

The LOGISTIC Procedure

Model Information	
Data Set	WORK.SORTTEMPTABLESORTED
Response Variable	LDL_x
Number of Response Levels	2
Model	binary logit
Optimization Technique	Fisher's scoring

Number of Observations Read	1701
Number of Observations Used	1701

Response Profile		
Ordered Value	LDL_x	Total Frequency
1	0	1384
2	1	317

Probability modeled is LDL_x='1'.

Model Convergence Status
Convergence criterion (GCONV=1E-8) satisfied.

Model Fit Statistics		
Criterion	Intercept Only	Intercept and Covariates
AIC	1638.032	1541.934
SC	1643.471	1590.885
-2 Log L	1636.032	1523.934

Testing Global Null Hypothesis: BETA=0			
Test	Chi-Square	DF	Pr > ChiSq
Likelihood Ratio	112.0984	8	<.0001
Score	121.6137	8	<.0001
Wald	105.2426	8	<.0001

Logistic Regression Results

The LOGISTIC Procedure

Analysis of Maximum Likelihood Estimates					
Parameter	DF	Estimate	Standard Error	Wald Chi-Square	Pr > ChiSq
Intercept	1	-6.4853	0.8413	59.4293	<.0001
A6	1	-0.0368	0.0289	1.6242	0.2025
age	1	0.0838	0.0137	37.2491	<.0001
BMI	1	0.1339	0.0188	50.5070	<.0001
SBP	1	0.00228	0.00702	0.1052	0.7457
D1_al	1	0.0593	0.1822	0.1058	0.7450
D2_al	1	0.1711	0.2043	0.7016	0.4023
D1_sm	1	0.0866	0.2895	0.0896	0.7647
D2_sm	1	0.1739	0.2717	0.4097	0.5221

Odds Ratio Estimates			
Effect	Point Estimate	95% Wald Confidence Limits	
A6	0.964	0.911	1.020
age	1.087	1.059	1.117
BMI	1.143	1.102	1.186
SBP	1.002	0.989	1.016
D1_al	1.061	0.742	1.516
D2_al	1.187	0.795	1.771
D1_sm	1.091	0.618	1.923
D2_sm	1.190	0.699	2.027

Association of Predicted Probabilities and Observed Responses			
Percent Concordant	67.2	Somers' D	0.351
Percent Discordant	32.1	Gamma	0.354
Percent Tied	0.7	Tau-a	0.107
Pairs	438728	c	0.676

Logistic Regression Results

The LOGISTIC Procedure

Model Information	
Data Set	WORK.SORTTEMPTABLESORTED
Response Variable	TG_x
Number of Response Levels	2
Model	binary logit
Optimization Technique	Fisher's scoring

Number of Observations Read	1701
Number of Observations Used	1701

Response Profile		
Ordered Value	TG_x	Total Frequency
1	0	1614
2	1	87

Probability modeled is TG_x='1'.

Model Convergence Status
Convergence criterion (GCONV=1E-8) satisfied.

Model Fit Statistics		
Criterion	Intercept Only	Intercept and Covariates
AIC	688.785	589.147
SC	694.224	638.097
-2 Log L	686.785	571.147

Testing Global Null Hypothesis: BETA=0			
Test	Chi-Square	DF	Pr > ChiSq
Likelihood Ratio	115.6388	8	<.0001
Score	167.1065	8	<.0001
Wald	113.1015	8	<.0001

Logistic Regression Results

The LOGISTIC Procedure

Analysis of Maximum Likelihood Estimates					
Parameter	DF	Estimate	Standard Error	Wald Chi-Square	Pr > ChiSq
Intercept	1	-10.2574	1.4031	53.4421	<.0001
A6	1	0.0169	0.0533	0.1006	0.7511
age	1	0.0403	0.0234	2.9768	0.0845
BMI	1	0.2506	0.0289	75.3611	<.0001
SBP	1	0.00193	0.0117	0.0270	0.8694
D1_al	1	0.0939	0.3252	0.0834	0.7728
D2_al	1	0.4219	0.3415	1.5258	0.2167
D1_sm	1	-0.1937	0.6181	0.0982	0.7540
D2_sm	1	0.3432	0.4078	0.7084	0.4000

Odds Ratio Estimates			
Effect	Point Estimate	95% Wald Confidence Limits	
A6	1.017	0.916	1.129
age	1.041	0.995	1.090
BMI	1.285	1.214	1.360
SBP	1.002	0.979	1.025
D1_al	1.098	0.581	2.078
D2_al	1.525	0.781	2.978
D1_sm	0.824	0.245	2.767
D2_sm	1.409	0.634	3.134

Association of Predicted Probabilities and Observed Responses			
Percent Concordant	77.5	Somers' D	0.564
Percent Discordant	21.0	Gamma	0.573
Percent Tied	1.5	Tau-a	0.055
Pairs	140418	c	0.782

Logistic Regression Results

The LOGISTIC Procedure

Model Information	
Data Set	WORK.SORTTEMPTABLESORTED
Response Variable	TC_x
Number of Response Levels	2
Model	binary logit
Optimization Technique	Fisher's scoring

Number of Observations Read	1701
Number of Observations Used	1701

Response Profile		
Ordered Value	TC_x	Total Frequency
1	0	1386
2	1	315

Probability modeled is TC_x='1'.

Model Convergence Status
Convergence criterion (GCONV=1E-8) satisfied.

Model Fit Statistics		
Criterion	Intercept Only	Intercept and Covariates
AIC	1632.121	1581.979
SC	1637.560	1630.930
-2 Log L	1630.121	1563.979

Testing Global Null Hypothesis: BETA=0			
Test	Chi-Square	DF	Pr > ChiSq
Likelihood Ratio	66.1425	8	<.0001
Score	70.8127	8	<.0001
Wald	65.4328	8	<.0001

Logistic Regression Results

The LOGISTIC Procedure

Analysis of Maximum Likelihood Estimates					
Parameter	DF	Estimate	Standard Error	Wald Chi-Square	Pr > ChiSq
Intercept	1	-4.2804	0.8456	25.6241	<.0001
C1	1	-0.0406	0.0342	1.4097	0.2351
age	1	0.0609	0.0135	20.1891	<.0001
BMI	1	0.1144	0.0186	37.7180	<.0001
SBP	1	-0.00864	0.00702	1.5149	0.2184
D1_al	1	0.1607	0.1743	0.8499	0.3566
D2_al	1	0.2860	0.1984	2.0781	0.1494
D1_sm	1	0.1624	0.2812	0.3336	0.5636
D2_sm	1	-0.0868	0.2853	0.0925	0.7611

Odds Ratio Estimates			
Effect	Point Estimate	95% Wald Confidence Limits	
C1	0.960	0.898	1.027
age	1.063	1.035	1.091
BMI	1.121	1.081	1.163
SBP	0.991	0.978	1.005
D1_al	1.174	0.835	1.652
D2_al	1.331	0.902	1.964
D1_sm	1.176	0.678	2.041
D2_sm	0.917	0.524	1.604

Association of Predicted Probabilities and Observed Responses			
Percent Concordant	62.1	Somers' D	0.250
Percent Discordant	37.1	Gamma	0.252
Percent Tied	0.9	Tau-a	0.075
Pairs	436590	c	0.625

Logistic Regression Results

The LOGISTIC Procedure

Model Information	
Data Set	WORK.SORTTEMPTABLESORTED
Response Variable	HDL_x
Number of Response Levels	2
Model	binary logit
Optimization Technique	Fisher's scoring

Number of Observations Read	1701
Number of Observations Used	1701

Response Profile		
Ordered Value	HDL_x	Total Frequency
1	0	1410
2	1	291

Probability modeled is HDL_x='1'.

Model Convergence Status
Convergence criterion (GCONV=1E-8) satisfied.

Model Fit Statistics		
Criterion	Intercept Only	Intercept and Covariates
AIC	1558.714	1412.752
SC	1564.153	1461.703
-2 Log L	1556.714	1394.752

Testing Global Null Hypothesis: BETA=0			
Test	Chi-Square	DF	Pr > ChiSq
Likelihood Ratio	161.9623	8	<.0001
Score	185.0786	8	<.0001
Wald	145.3599	8	<.0001

Logistic Regression Results

The LOGISTIC Procedure

Analysis of Maximum Likelihood Estimates					
Parameter	DF	Estimate	Standard Error	Wald Chi-Square	Pr > ChiSq
Intercept	1	-6.0316	0.8970	45.2118	<.0001
C1	1	-0.0272	0.0364	0.5557	0.4560
age	1	0.00946	0.0145	0.4256	0.5141
BMI	1	0.2243	0.0203	122.4059	<.0001
SBP	1	-0.00591	0.00735	0.6449	0.4219
D1_al	1	0.2710	0.1832	2.1881	0.1391
D2_al	1	0.3786	0.2113	3.2115	0.0731
D1_sm	1	0.2399	0.3013	0.6339	0.4259
D2_sm	1	-0.0758	0.3045	0.0619	0.8036

Odds Ratio Estimates			
Effect	Point Estimate	95% Wald Confidence Limits	
C1	0.973	0.906	1.045
age	1.010	0.981	1.039
BMI	1.251	1.203	1.302
SBP	0.994	0.980	1.009
D1_al	1.311	0.916	1.878
D2_al	1.460	0.965	2.209
D1_sm	1.271	0.704	2.294
D2_sm	0.927	0.510	1.684

Association of Predicted Probabilities and Observed Responses			
Percent Concordant	72.2	Somers' D	0.450
Percent Discordant	27.2	Gamma	0.453
Percent Tied	0.6	Tau-a	0.128
Pairs	410310	c	0.725

Logistic Regression Results

The LOGISTIC Procedure

Model Information	
Data Set	WORK.SORTTEMPTABLESORTED
Response Variable	LDL_x
Number of Response Levels	2
Model	binary logit
Optimization Technique	Fisher's scoring

Number of Observations Read	1701
Number of Observations Used	1701

Response Profile		
Ordered Value	LDL_x	Total Frequency
1	0	1384
2	1	317

Probability modeled is LDL_x='1'.

Model Convergence Status
Convergence criterion (GCONV=1E-8) satisfied.

Model Fit Statistics		
Criterion	Intercept Only	Intercept and Covariates
AIC	1638.032	1540.050
SC	1643.471	1589.001
-2 Log L	1636.032	1522.050

Testing Global Null Hypothesis: BETA=0			
Test	Chi-Square	DF	Pr > ChiSq
Likelihood Ratio	113.9823	8	<.0001
Score	123.4895	8	<.0001
Wald	106.9030	8	<.0001

Logistic Regression Results

The LOGISTIC Procedure

Analysis of Maximum Likelihood Estimates					
Parameter	DF	Estimate	Standard Error	Wald Chi-Square	Pr > ChiSq
Intercept	1	-6.2053	0.8609	51.9504	<.0001
C1	1	-0.0649	0.0347	3.5042	0.0612
age	1	0.0843	0.0137	37.7222	<.0001
BMI	1	0.1352	0.0188	51.5344	<.0001
SBP	1	0.00178	0.00704	0.0637	0.8008
D1_al	1	0.0276	0.1820	0.0230	0.8795
D2_al	1	0.1386	0.2051	0.4569	0.4991
D1_sm	1	0.0959	0.2900	0.1092	0.7410
D2_sm	1	0.2055	0.2706	0.5767	0.4476

Odds Ratio Estimates			
Effect	Point Estimate	95% Wald Confidence Limits	
C1	0.937	0.876	1.003
age	1.088	1.059	1.118
BMI	1.145	1.103	1.188
SBP	1.002	0.988	1.016
D1_al	1.028	0.720	1.469
D2_al	1.149	0.769	1.717
D1_sm	1.101	0.623	1.943
D2_sm	1.228	0.723	2.087

Association of Predicted Probabilities and Observed Responses			
Percent Concordant	67.4	Somers' D	0.354
Percent Discordant	32.0	Gamma	0.357
Percent Tied	0.7	Tau-a	0.108
Pairs	438728	c	0.677

Logistic Regression Results

The LOGISTIC Procedure

Model Information	
Data Set	WORK.SORTTEMPTABLESORTED
Response Variable	TG_x
Number of Response Levels	2
Model	binary logit
Optimization Technique	Fisher's scoring

Number of Observations Read	1701
Number of Observations Used	1701

Response Profile		
Ordered Value	TG_x	Total Frequency
1	0	1614
2	1	87

Probability modeled is TG_x='1'.

Model Convergence Status
Convergence criterion (GCONV=1E-8) satisfied.

Model Fit Statistics		
Criterion	Intercept Only	Intercept and Covariates
AIC	688.785	588.368
SC	694.224	637.318
-2 Log L	686.785	570.368

Testing Global Null Hypothesis: BETA=0			
Test	Chi-Square	DF	Pr > ChiSq
Likelihood Ratio	116.4177	8	<.0001
Score	167.5810	8	<.0001
Wald	113.6261	8	<.0001

Logistic Regression Results

The LOGISTIC Procedure

Analysis of Maximum Likelihood Estimates					
Parameter	DF	Estimate	Standard Error	Wald Chi-Square	Pr > ChiSq
Intercept	1	-9.4885	1.4153	44.9489	<.0001
C1	1	-0.0573	0.0609	0.8852	0.3468
age	1	0.0405	0.0234	2.9992	0.0833
BMI	1	0.2512	0.0289	75.4438	<.0001
SBP	1	0.00147	0.0118	0.0155	0.9008
D1_al	1	0.0841	0.3250	0.0669	0.7958
D2_al	1	0.4050	0.3415	1.4065	0.2356
D1_sm	1	-0.2182	0.6199	0.1239	0.7249
D2_sm	1	0.3676	0.4080	0.8117	0.3676

Odds Ratio Estimates			
Effect	Point Estimate	95% Wald Confidence Limits	
C1	0.944	0.838	1.064
age	1.041	0.995	1.090
BMI	1.286	1.215	1.360
SBP	1.001	0.979	1.025
D1_al	1.088	0.575	2.056
D2_al	1.499	0.768	2.928
D1_sm	0.804	0.239	2.710
D2_sm	1.444	0.649	3.213

Association of Predicted Probabilities and Observed Responses			
Percent Concordant	77.9	Somers' D	0.572
Percent Discordant	20.6	Gamma	0.581
Percent Tied	1.5	Tau-a	0.056
Pairs	140418	c	0.786

Logistic Regression Results

The LOGISTIC Procedure

Model Information	
Data Set	WORK.SORTTEMPTABLESORTED
Response Variable	TC_x
Number of Response Levels	2
Model	binary logit
Optimization Technique	Fisher's scoring

Number of Observations Read	1701
Number of Observations Used	1701

Response Profile		
Ordered Value	TC_x	Total Frequency
1	0	1386
2	1	315

Probability modeled is TC_x='1'.

Model Convergence Status
Convergence criterion (GCONV=1E-8) satisfied.

Model Fit Statistics		
Criterion	Intercept Only	Intercept and Covariates
AIC	1632.121	1581.268
SC	1637.560	1630.218
-2 Log L	1630.121	1563.268

Testing Global Null Hypothesis: BETA=0			
Test	Chi-Square	DF	Pr > ChiSq
Likelihood Ratio	66.8537	8	<.0001
Score	71.4109	8	<.0001
Wald	66.1204	8	<.0001

Logistic Regression Results

The LOGISTIC Procedure

Analysis of Maximum Likelihood Estimates					
Parameter	DF	Estimate	Standard Error	Wald Chi-Square	Pr > ChiSq
Intercept	1	-4.3532	0.8051	29.2358	<.0001
C2	1	-0.0424	0.0291	2.1186	0.1455
age	1	0.0635	0.0137	21.5041	<.0001
BMI	1	0.1126	0.0187	36.3987	<.0001
SBP	1	-0.00801	0.00702	1.3004	0.2541
D1_al	1	0.1736	0.1743	0.9924	0.3192
D2_al	1	0.3061	0.1979	2.3922	0.1219
D1_sm	1	0.1534	0.2809	0.2981	0.5851
D2_sm	1	-0.1041	0.2854	0.1331	0.7153

Odds Ratio Estimates			
Effect	Point Estimate	95% Wald Confidence Limits	
C2	0.959	0.905	1.015
age	1.066	1.037	1.095
BMI	1.119	1.079	1.161
SBP	0.992	0.978	1.006
D1_al	1.190	0.845	1.674
D2_al	1.358	0.921	2.002
D1_sm	1.166	0.672	2.022
D2_sm	0.901	0.515	1.577

Association of Predicted Probabilities and Observed Responses			
Percent Concordant	62.2	Somers' D	0.253
Percent Discordant	36.9	Gamma	0.255
Percent Tied	0.9	Tau-a	0.076
Pairs	436590	c	0.626

Logistic Regression Results

The LOGISTIC Procedure

Model Information	
Data Set	WORK.SORTTEMPTABLESORTED
Response Variable	HDL_x
Number of Response Levels	2
Model	binary logit
Optimization Technique	Fisher's scoring

Number of Observations Read	1701
Number of Observations Used	1701

Response Profile		
Ordered Value	HDL_x	Total Frequency
1	0	1410
2	1	291

Probability modeled is HDL_x='1'.

Model Convergence Status
Convergence criterion (GCONV=1E-8) satisfied.

Model Fit Statistics		
Criterion	Intercept Only	Intercept and Covariates
AIC	1558.714	1413.170
SC	1564.153	1462.121
-2 Log L	1556.714	1395.170

Testing Global Null Hypothesis: BETA=0			
Test	Chi-Square	DF	Pr > ChiSq
Likelihood Ratio	161.5443	8	<.0001
Score	184.6394	8	<.0001
Wald	144.8140	8	<.0001

Logistic Regression Results

The LOGISTIC Procedure

Analysis of Maximum Likelihood Estimates					
Parameter	DF	Estimate	Standard Error	Wald Chi-Square	Pr > ChiSq
Intercept	1	-6.4124	0.8549	56.2662	<.0001
C2	1	0.0115	0.0310	0.1369	0.7114
age	1	0.00861	0.0146	0.3464	0.5562
BMI	1	0.2246	0.0203	122.0728	<.0001
SBP	1	-0.00580	0.00735	0.6225	0.4301
D1_al	1	0.2774	0.1828	2.3011	0.1293
D2_al	1	0.3893	0.2107	3.4133	0.0647
D1_sm	1	0.2443	0.3008	0.6596	0.4167
D2_sm	1	-0.0860	0.3048	0.0796	0.7779

Odds Ratio Estimates			
Effect	Point Estimate	95% Wald Confidence Limits	
C2	1.012	0.952	1.075
age	1.009	0.980	1.038
BMI	1.252	1.203	1.303
SBP	0.994	0.980	1.009
D1_al	1.320	0.922	1.888
D2_al	1.476	0.977	2.231
D1_sm	1.277	0.708	2.302
D2_sm	0.918	0.505	1.668

Association of Predicted Probabilities and Observed Responses			
Percent Concordant	72.1	Somers' D	0.448
Percent Discordant	27.3	Gamma	0.451
Percent Tied	0.6	Tau-a	0.127
Pairs	410310	c	0.724

Logistic Regression Results

The LOGISTIC Procedure

Model Information	
Data Set	WORK.SORTTEMPTABLESORTED
Response Variable	LDL_x
Number of Response Levels	2
Model	binary logit
Optimization Technique	Fisher's scoring

Number of Observations Read	1701
Number of Observations Used	1701

Response Profile		
Ordered Value	LDL_x	Total Frequency
1	0	1384
2	1	317

Probability modeled is LDL_x='1'.

Model Convergence Status
Convergence criterion (GCONV=1E-8) satisfied.

Model Fit Statistics		
Criterion	Intercept Only	Intercept and Covariates
AIC	1638.032	1543.532
SC	1643.471	1592.482
-2 Log L	1636.032	1525.532

Testing Global Null Hypothesis: BETA=0			
Test	Chi-Square	DF	Pr > ChiSq
Likelihood Ratio	110.5007	8	<.0001
Score	120.3333	8	<.0001
Wald	104.1127	8	<.0001

Logistic Regression Results

The LOGISTIC Procedure

Analysis of Maximum Likelihood Estimates					
Parameter	DF	Estimate	Standard Error	Wald Chi-Square	Pr > ChiSq
Intercept	1	-6.9010	0.8266	69.6969	<.0001
C2	1	0.00444	0.0297	0.0225	0.8809
age	1	0.0838	0.0138	36.5928	<.0001
BMI	1	0.1345	0.0189	50.8692	<.0001
SBP	1	0.00217	0.00702	0.0959	0.7568
D1_al	1	0.0412	0.1817	0.0514	0.8207
D2_al	1	0.1636	0.2042	0.6418	0.4231
D1_sm	1	0.0961	0.2893	0.1104	0.7397
D2_sm	1	0.1818	0.2714	0.4490	0.5028

Odds Ratio Estimates			
Effect	Point Estimate	95% Wald Confidence Limits	
C2	1.004	0.948	1.065
age	1.087	1.058	1.117
BMI	1.144	1.102	1.187
SBP	1.002	0.988	1.016
D1_al	1.042	0.730	1.488
D2_al	1.178	0.789	1.757
D1_sm	1.101	0.624	1.941
D2_sm	1.199	0.705	2.042

Association of Predicted Probabilities and Observed Responses			
Percent Concordant	67.0	Somers' D	0.348
Percent Discordant	32.3	Gamma	0.350
Percent Tied	0.7	Tau-a	0.105
Pairs	438728	c	0.674

Logistic Regression Results

The LOGISTIC Procedure

Model Information	
Data Set	WORK.SORTTEMPTABLESORTED
Response Variable	TG_x
Number of Response Levels	2
Model	binary logit
Optimization Technique	Fisher's scoring

Number of Observations Read	1701
Number of Observations Used	1701

Response Profile		
Ordered Value	TG_x	Total Frequency
1	0	1614
2	1	87

Probability modeled is TG_x='1'.

Model Convergence Status
Convergence criterion (GCONV=1E-8) satisfied.

Model Fit Statistics		
Criterion	Intercept Only	Intercept and Covariates
AIC	688.785	589.247
SC	694.224	638.198
-2 Log L	686.785	571.247

Testing Global Null Hypothesis: BETA=0			
Test	Chi-Square	DF	Pr > ChiSq
Likelihood Ratio	115.5383	8	<.0001
Score	167.0600	8	<.0001
Wald	112.9254	8	<.0001

Logistic Regression Results

The LOGISTIC Procedure

Analysis of Maximum Likelihood Estimates					
Parameter	DF	Estimate	Standard Error	Wald Chi-Square	Pr > ChiSq
Intercept	1	-10.0831	1.3400	56.6245	<.0001
C2	1	0.000987	0.0522	0.0004	0.9849
age	1	0.0402	0.0236	2.9023	0.0885
BMI	1	0.2506	0.0289	75.0115	<.0001
SBP	1	0.00185	0.0117	0.0248	0.8749
D1_al	1	0.1038	0.3238	0.1028	0.7485
D2_al	1	0.4264	0.3414	1.5606	0.2116
D1_sm	1	-0.2007	0.6182	0.1054	0.7455
D2_sm	1	0.3356	0.4073	0.6787	0.4100

Odds Ratio Estimates			
Effect	Point Estimate	95% Wald Confidence Limits	
C2	1.001	0.904	1.109
age	1.041	0.994	1.090
BMI	1.285	1.214	1.360
SBP	1.002	0.979	1.025
D1_al	1.109	0.588	2.093
D2_al	1.532	0.785	2.991
D1_sm	0.818	0.244	2.748
D2_sm	1.399	0.630	3.108

Association of Predicted Probabilities and Observed Responses			
Percent Concordant	77.2	Somers' D	0.559
Percent Discordant	21.3	Gamma	0.567
Percent Tied	1.5	Tau-a	0.054
Pairs	140418	c	0.779

Logistic Regression Results

The LOGISTIC Procedure

Model Information	
Data Set	WORK.SORTTEMPTABLESORTED
Response Variable	TC_x
Number of Response Levels	2
Model	binary logit
Optimization Technique	Fisher's scoring

Number of Observations Read	1701
Number of Observations Used	1701

Response Profile		
Ordered Value	TC_x	Total Frequency
1	0	1386
2	1	315

Probability modeled is TC_x='1'.

Model Convergence Status
Convergence criterion (GCONV=1E-8) satisfied.

Model Fit Statistics		
Criterion	Intercept Only	Intercept and Covariates
AIC	1632.121	1577.116
SC	1637.560	1626.067
-2 Log L	1630.121	1559.116

Testing Global Null Hypothesis: BETA=0			
Test	Chi-Square	DF	Pr > ChiSq
Likelihood Ratio	71.0056	8	<.0001
Score	75.0090	8	<.0001
Wald	69.6001	8	<.0001

Logistic Regression Results

The LOGISTIC Procedure

Analysis of Maximum Likelihood Estimates					
Parameter	DF	Estimate	Standard Error	Wald Chi-Square	Pr > ChiSq
Intercept	1	-3.9821	0.8190	23.6415	<.0001
C3	1	-0.0860	0.0343	6.2702	0.0123
age	1	0.0672	0.0138	23.5640	<.0001
BMI	1	0.1137	0.0187	37.1346	<.0001
SBP	1	-0.00775	0.00701	1.2204	0.2693
D1_al	1	0.2079	0.1749	1.4126	0.2346
D2_al	1	0.3003	0.1985	2.2898	0.1302
D1_sm	1	0.1305	0.2809	0.2157	0.6423
D2_sm	1	-0.1585	0.2868	0.3054	0.5805

Odds Ratio Estimates			
Effect	Point Estimate	95% Wald Confidence Limits	
C3	0.918	0.858	0.981
age	1.070	1.041	1.099
BMI	1.120	1.080	1.162
SBP	0.992	0.979	1.006
D1_al	1.231	0.874	1.734
D2_al	1.350	0.915	1.992
D1_sm	1.139	0.657	1.976
D2_sm	0.853	0.487	1.497

Association of Predicted Probabilities and Observed Responses			
Percent Concordant	62.5	Somers' D	0.258
Percent Discordant	36.7	Gamma	0.260
Percent Tied	0.8	Tau-a	0.078
Pairs	436590	c	0.629

Logistic Regression Results

The LOGISTIC Procedure

Model Information	
Data Set	WORK.SORTTEMPTABLESORTED
Response Variable	HDL_x
Number of Response Levels	2
Model	binary logit
Optimization Technique	Fisher's scoring

Number of Observations Read	1701
Number of Observations Used	1701

Response Profile		
Ordered Value	HDL_x	Total Frequency
1	0	1410
2	1	291

Probability modeled is HDL_x='1'.

Model Convergence Status
Convergence criterion (GCONV=1E-8) satisfied.

Model Fit Statistics		
Criterion	Intercept Only	Intercept and Covariates
AIC	1558.714	1413.070
SC	1564.153	1462.021
-2 Log L	1556.714	1395.070

Testing Global Null Hypothesis: BETA=0			
Test	Chi-Square	DF	Pr > ChiSq
Likelihood Ratio	161.6445	8	<.0001
Score	184.7589	8	<.0001
Wald	144.7941	8	<.0001

Logistic Regression Results

The LOGISTIC Procedure

Analysis of Maximum Likelihood Estimates					
Parameter	DF	Estimate	Standard Error	Wald Chi-Square	Pr > ChiSq
Intercept	1	-6.4765	0.8790	54.2933	<.0001
C3	1	0.0178	0.0365	0.2368	0.6266
age	1	0.00796	0.0148	0.2902	0.5901
BMI	1	0.2244	0.0203	122.2336	<.0001
SBP	1	-0.00576	0.00735	0.6148	0.4330
D1_al	1	0.2703	0.1836	2.1687	0.1408
D2_al	1	0.3911	0.2107	3.4461	0.0634
D1_sm	1	0.2498	0.3012	0.6879	0.4069
D2_sm	1	-0.0742	0.3059	0.0588	0.8083

Odds Ratio Estimates			
Effect	Point Estimate	95% Wald Confidence Limits	
C3	1.018	0.948	1.094
age	1.008	0.979	1.038
BMI	1.252	1.203	1.302
SBP	0.994	0.980	1.009
D1_al	1.310	0.914	1.878
D2_al	1.479	0.978	2.235
D1_sm	1.284	0.711	2.317
D2_sm	0.928	0.510	1.691

Association of Predicted Probabilities and Observed Responses			
Percent Concordant	72.2	Somers' D	0.449
Percent Discordant	27.2	Gamma	0.452
Percent Tied	0.6	Tau-a	0.127
Pairs	410310	c	0.725

Logistic Regression Results

The LOGISTIC Procedure

Model Information	
Data Set	WORK.SORTTEMPTABLESORTED
Response Variable	LDL_x
Number of Response Levels	2
Model	binary logit
Optimization Technique	Fisher's scoring

Number of Observations Read	1701
Number of Observations Used	1701

Response Profile		
Ordered Value	LDL_x	Total Frequency
1	0	1384
2	1	317

Probability modeled is LDL_x='1'.

Model Convergence Status
Convergence criterion (GCONV=1E-8) satisfied.

Model Fit Statistics		
Criterion	Intercept Only	Intercept and Covariates
AIC	1638.032	1541.243
SC	1643.471	1590.194
-2 Log L	1636.032	1523.243

Testing Global Null Hypothesis: BETA=0			
Test	Chi-Square	DF	Pr > ChiSq
Likelihood Ratio	112.7894	8	<.0001
Score	122.0057	8	<.0001
Wald	105.9391	8	<.0001

Logistic Regression Results

The LOGISTIC Procedure

Analysis of Maximum Likelihood Estimates					
Parameter	DF	Estimate	Standard Error	Wald Chi-Square	Pr > ChiSq
Intercept	1	-6.4220	0.8385	58.6610	<.0001
C3	1	-0.0530	0.0348	2.3149	0.1281
age	1	0.0881	0.0140	39.5424	<.0001
BMI	1	0.1341	0.0188	50.7147	<.0001
SBP	1	0.00260	0.00702	0.1374	0.7109
D1_al	1	0.0647	0.1823	0.1260	0.7226
D2_al	1	0.1610	0.2045	0.6198	0.4311
D1_sm	1	0.0745	0.2893	0.0663	0.7968
D2_sm	1	0.1483	0.2721	0.2970	0.5858

Odds Ratio Estimates			
Effect	Point Estimate	95% Wald Confidence Limits	
C3	0.948	0.886	1.015
age	1.092	1.062	1.122
BMI	1.143	1.102	1.186
SBP	1.003	0.989	1.016
D1_al	1.067	0.746	1.525
D2_al	1.175	0.787	1.754
D1_sm	1.077	0.611	1.899
D2_sm	1.160	0.680	1.977

Association of Predicted Probabilities and Observed Responses			
Percent Concordant	67.0	Somers' D	0.347
Percent Discordant	32.3	Gamma	0.349
Percent Tied	0.7	Tau-a	0.105
Pairs	438728	c	0.674

Logistic Regression Results

The LOGISTIC Procedure

Model Information	
Data Set	WORK.SORTTEMPTABLESORTED
Response Variable	TG_x
Number of Response Levels	2
Model	binary logit
Optimization Technique	Fisher's scoring

Number of Observations Read	1701
Number of Observations Used	1701

Response Profile		
Ordered Value	TG_x	Total Frequency
1	0	1614
2	1	87

Probability modeled is TG_x='1'.

Model Convergence Status
Convergence criterion (GCONV=1E-8) satisfied.

Model Fit Statistics		
Criterion	Intercept Only	Intercept and Covariates
AIC	688.785	587.261
SC	694.224	636.212
-2 Log L	686.785	569.261

Testing Global Null Hypothesis: BETA=0			
Test	Chi-Square	DF	Pr > ChiSq
Likelihood Ratio	117.5243	8	<.0001
Score	168.0482	8	<.0001
Wald	114.8223	8	<.0001

Logistic Regression Results

The LOGISTIC Procedure

Analysis of Maximum Likelihood Estimates					
Parameter	DF	Estimate	Standard Error	Wald Chi-Square	Pr > ChiSq
Intercept	1	-9.3612	1.3639	47.1112	<.0001
C3	1	-0.0869	0.0614	2.0082	0.1564
age	1	0.0481	0.0241	3.9971	0.0456
BMI	1	0.2519	0.0290	75.6827	<.0001
SBP	1	0.00192	0.0117	0.0268	0.8700
D1_al	1	0.1450	0.3250	0.1990	0.6555
D2_al	1	0.4023	0.3412	1.3897	0.2385
D1_sm	1	-0.2399	0.6180	0.1506	0.6979
D2_sm	1	0.2705	0.4085	0.4384	0.5079

Odds Ratio Estimates			
Effect	Point Estimate	95% Wald Confidence Limits	
C3	0.917	0.813	1.034
age	1.049	1.001	1.100
BMI	1.286	1.215	1.362
SBP	1.002	0.979	1.025
D1_al	1.156	0.611	2.186
D2_al	1.495	0.766	2.919
D1_sm	0.787	0.234	2.642
D2_sm	1.311	0.588	2.919

Association of Predicted Probabilities and Observed Responses			
Percent Concordant	77.4	Somers' D	0.564
Percent Discordant	21.1	Gamma	0.572
Percent Tied	1.5	Tau-a	0.055
Pairs	140418	c	0.782

Logistic Regression Results

The LOGISTIC Procedure

Model Information	
Data Set	WORK.SORTTEMPTABLESORTED
Response Variable	TC_x
Number of Response Levels	2
Model	binary logit
Optimization Technique	Fisher's scoring

Number of Observations Read	1701
Number of Observations Used	1701

Response Profile		
Ordered Value	TC_x	Total Frequency
1	0	1386
2	1	315

Probability modeled is TC_x='1'.

Model Convergence Status
Convergence criterion (GCONV=1E-8) satisfied.

Model Fit Statistics		
Criterion	Intercept Only	Intercept and Covariates
AIC	1632.121	1581.096
SC	1637.560	1630.047
-2 Log L	1630.121	1563.096

Testing Global Null Hypothesis: BETA=0			
Test	Chi-Square	DF	Pr > ChiSq
Likelihood Ratio	67.0256	8	<.0001
Score	71.5172	8	<.0001
Wald	66.2321	8	<.0001

Logistic Regression Results

The LOGISTIC Procedure

Analysis of Maximum Likelihood Estimates					
Parameter	DF	Estimate	Standard Error	Wald Chi-Square	Pr > ChiSq
Intercept	1	-4.2030	0.8350	25.3335	<.0001
C4	1	-0.0459	0.0303	2.2936	0.1299
age	1	0.0615	0.0136	20.5559	<.0001
BMI	1	0.1110	0.0187	35.1652	<.0001
SBP	1	-0.00838	0.00702	1.4233	0.2329
D1_al	1	0.1821	0.1743	1.0915	0.2961
D2_al	1	0.3136	0.1981	2.5055	0.1135
D1_sm	1	0.1511	0.2809	0.2894	0.5906
D2_sm	1	-0.1057	0.2848	0.1376	0.7107

Odds Ratio Estimates			
Effect	Point Estimate	95% Wald Confidence Limits	
C4	0.955	0.900	1.014
age	1.063	1.036	1.092
BMI	1.117	1.077	1.159
SBP	0.992	0.978	1.005
D1_al	1.200	0.853	1.688
D2_al	1.368	0.928	2.018
D1_sm	1.163	0.671	2.017
D2_sm	0.900	0.515	1.572

Association of Predicted Probabilities and Observed Responses			
Percent Concordant	62.3	Somers' D	0.255
Percent Discordant	36.8	Gamma	0.257
Percent Tied	0.9	Tau-a	0.077
Pairs	436590	c	0.627

Logistic Regression Results

The LOGISTIC Procedure

Model Information	
Data Set	WORK.SORTTEMPTABLESORTED
Response Variable	HDL_x
Number of Response Levels	2
Model	binary logit
Optimization Technique	Fisher's scoring

Number of Observations Read	1701
Number of Observations Used	1701

Response Profile		
Ordered Value	HDL_x	Total Frequency
1	0	1410
2	1	291

Probability modeled is HDL_x='1'.

Model Convergence Status
Convergence criterion (GCONV=1E-8) satisfied.

Model Fit Statistics		
Criterion	Intercept Only	Intercept and Covariates
AIC	1558.714	1409.765
SC	1564.153	1458.716
-2 Log L	1556.714	1391.765

Testing Global Null Hypothesis: BETA=0			
Test	Chi-Square	DF	Pr > ChiSq
Likelihood Ratio	164.9495	8	<.0001
Score	187.7148	8	<.0001
Wald	147.7606	8	<.0001

Logistic Regression Results

The LOGISTIC Procedure

Analysis of Maximum Likelihood Estimates					
Parameter	DF	Estimate	Standard Error	Wald Chi-Square	Pr > ChiSq
Intercept	1	-5.6588	0.8825	41.1130	<.0001
C4	1	-0.0614	0.0326	3.5462	0.0597
age	1	0.0104	0.0145	0.5178	0.4718
BMI	1	0.2201	0.0204	116.8890	<.0001
SBP	1	-0.00569	0.00737	0.5970	0.4397
D1_al	1	0.2908	0.1834	2.5149	0.1128
D2_al	1	0.4013	0.2113	3.6066	0.0576
D1_sm	1	0.2303	0.3010	0.5855	0.4442
D2_sm	1	-0.0924	0.3029	0.0930	0.7604

Odds Ratio Estimates			
Effect	Point Estimate	95% Wald Confidence Limits	
C4	0.940	0.882	1.003
age	1.010	0.982	1.040
BMI	1.246	1.197	1.297
SBP	0.994	0.980	1.009
D1_al	1.337	0.934	1.916
D2_al	1.494	0.987	2.260
D1_sm	1.259	0.698	2.271
D2_sm	0.912	0.504	1.651

Association of Predicted Probabilities and Observed Responses			
Percent Concordant	72.5	Somers' D	0.455
Percent Discordant	26.9	Gamma	0.458
Percent Tied	0.6	Tau-a	0.129
Pairs	410310	c	0.728

Logistic Regression Results

The LOGISTIC Procedure

Model Information	
Data Set	WORK.SORTTEMPTABLESORTED
Response Variable	LDL_x
Number of Response Levels	2
Model	binary logit
Optimization Technique	Fisher's scoring

Number of Observations Read	1701
Number of Observations Used	1701

Response Profile		
Ordered Value	LDL_x	Total Frequency
1	0	1384
2	1	317

Probability modeled is LDL_x='1'.

Model Convergence Status
Convergence criterion (GCONV=1E-8) satisfied.

Model Fit Statistics		
Criterion	Intercept Only	Intercept and Covariates
AIC	1638.032	1542.051
SC	1643.471	1591.002
-2 Log L	1636.032	1524.051

Testing Global Null Hypothesis: BETA=0			
Test	Chi-Square	DF	Pr > ChiSq
Likelihood Ratio	111.9816	8	<.0001
Score	121.5269	8	<.0001
Wald	105.4341	8	<.0001

Logistic Regression Results

The LOGISTIC Procedure

Analysis of Maximum Likelihood Estimates					
Parameter	DF	Estimate	Standard Error	Wald Chi-Square	Pr > ChiSq
Intercept	1	-6.4559	0.8540	57.1407	<.0001
C4	1	-0.0377	0.0308	1.5054	0.2198
age	1	0.0845	0.0137	37.9285	<.0001
BMI	1	0.1318	0.0189	48.6531	<.0001
SBP	1	0.00221	0.00703	0.0985	0.7536
D1_al	1	0.0523	0.1818	0.0827	0.7736
D2_al	1	0.1743	0.2045	0.7267	0.3939
D1_sm	1	0.0873	0.2892	0.0911	0.7627
D2_sm	1	0.1789	0.2705	0.4373	0.5084

Odds Ratio Estimates			
Effect	Point Estimate	95% Wald Confidence Limits	
C4	0.963	0.907	1.023
age	1.088	1.059	1.118
BMI	1.141	1.099	1.184
SBP	1.002	0.989	1.016
D1_al	1.054	0.738	1.505
D2_al	1.190	0.797	1.777
D1_sm	1.091	0.619	1.924
D2_sm	1.196	0.704	2.032

Association of Predicted Probabilities and Observed Responses			
Percent Concordant	67.1	Somers' D	0.348
Percent Discordant	32.3	Gamma	0.350
Percent Tied	0.7	Tau-a	0.106
Pairs	438728	c	0.674

Logistic Regression Results

The LOGISTIC Procedure

Model Information	
Data Set	WORK.SORTTEMPTABLESORTED
Response Variable	TG_x
Number of Response Levels	2
Model	binary logit
Optimization Technique	Fisher's scoring

Number of Observations Read	1701
Number of Observations Used	1701

Response Profile		
Ordered Value	TG_x	Total Frequency
1	0	1614
2	1	87

Probability modeled is TG_x='1'.

Model Convergence Status
Convergence criterion (GCONV=1E-8) satisfied.

Model Fit Statistics		
Criterion	Intercept Only	Intercept and Covariates
AIC	688.785	588.017
SC	694.224	636.968
-2 Log L	686.785	570.017

Testing Global Null Hypothesis: BETA=0			
Test	Chi-Square	DF	Pr > ChiSq
Likelihood Ratio	116.7685	8	<.0001
Score	167.6335	8	<.0001
Wald	113.9813	8	<.0001

Logistic Regression Results

The LOGISTIC Procedure

Analysis of Maximum Likelihood Estimates					
Parameter	DF	Estimate	Standard Error	Wald Chi-Square	Pr > ChiSq
Intercept	1	-9.4302	1.3989	45.4418	<.0001
C4	1	-0.0624	0.0561	1.2348	0.2665
age	1	0.0417	0.0234	3.1771	0.0747
BMI	1	0.2466	0.0291	71.9542	<.0001
SBP	1	0.00192	0.0118	0.0264	0.8709
D1_al	1	0.1163	0.3242	0.1286	0.7199
D2_al	1	0.4366	0.3411	1.6384	0.2005
D1_sm	1	-0.2133	0.6195	0.1185	0.7306
D2_sm	1	0.3500	0.4044	0.7490	0.3868

Odds Ratio Estimates			
Effect	Point Estimate	95% Wald Confidence Limits	
C4	0.940	0.842	1.049
age	1.043	0.996	1.091
BMI	1.280	1.209	1.355
SBP	1.002	0.979	1.025
D1_al	1.123	0.595	2.121
D2_al	1.547	0.793	3.019
D1_sm	0.808	0.240	2.721
D2_sm	1.419	0.642	3.135

Association of Predicted Probabilities and Observed Responses			
Percent Concordant	77.9	Somers' D	0.572
Percent Discordant	20.6	Gamma	0.581
Percent Tied	1.5	Tau-a	0.056
Pairs	140418	c	0.786

Logistic Regression Results

The LOGISTIC Procedure

Model Information	
Data Set	WORK.SORTTEMPTABLESORTED
Response Variable	TC_x
Number of Response Levels	2
Model	binary logit
Optimization Technique	Fisher's scoring

Number of Observations Read	1701
Number of Observations Used	1701

Response Profile		
Ordered Value	TC_x	Total Frequency
1	0	1386
2	1	315

Probability modeled is TC_x='1'.

Model Convergence Status
Convergence criterion (GCONV=1E-8) satisfied.

Model Fit Statistics		
Criterion	Intercept Only	Intercept and Covariates
AIC	1632.121	1582.242
SC	1637.560	1631.193
-2 Log L	1630.121	1564.242

Testing Global Null Hypothesis: BETA=0			
Test	Chi-Square	DF	Pr > ChiSq
Likelihood Ratio	65.8796	8	<.0001
Score	70.4358	8	<.0001
Wald	65.1807	8	<.0001

Logistic Regression Results

The LOGISTIC Procedure

Analysis of Maximum Likelihood Estimates					
Parameter	DF	Estimate	Standard Error	Wald Chi-Square	Pr > ChiSq
Intercept	1	-4.4369	0.8075	30.1936	<.0001
C5	1	-0.0327	0.0306	1.1457	0.2845
age	1	0.0631	0.0137	21.1193	<.0001
BMI	1	0.1121	0.0187	35.9228	<.0001
SBP	1	-0.00795	0.00702	1.2820	0.2575
D1_al	1	0.1726	0.1742	0.9814	0.3219
D2_al	1	0.2959	0.1980	2.2328	0.1351
D1_sm	1	0.1545	0.2810	0.3023	0.5824
D2_sm	1	-0.1033	0.2853	0.1311	0.7173

Odds Ratio Estimates			
Effect	Point Estimate	95% Wald Confidence Limits	
C5	0.968	0.912	1.028
age	1.065	1.037	1.094
BMI	1.119	1.078	1.160
SBP	0.992	0.979	1.006
D1_al	1.188	0.845	1.672
D2_al	1.344	0.912	1.982
D1_sm	1.167	0.673	2.024
D2_sm	0.902	0.516	1.577

Association of Predicted Probabilities and Observed Responses			
Percent Concordant	62.1	Somers' D	0.252
Percent Discordant	37.0	Gamma	0.254
Percent Tied	0.9	Tau-a	0.076
Pairs	436590	c	0.626

Logistic Regression Results

The LOGISTIC Procedure

Model Information	
Data Set	WORK.SORTTEMPTABLESORTED
Response Variable	HDL_x
Number of Response Levels	2
Model	binary logit
Optimization Technique	Fisher's scoring

Number of Observations Read	1701
Number of Observations Used	1701

Response Profile		
Ordered Value	HDL_x	Total Frequency
1	0	1410
2	1	291

Probability modeled is HDL_x='1'.

Model Convergence Status
Convergence criterion (GCONV=1E-8) satisfied.

Model Fit Statistics		
Criterion	Intercept Only	Intercept and Covariates
AIC	1558.714	1410.227
SC	1564.153	1459.178
-2 Log L	1556.714	1392.227

Testing Global Null Hypothesis: BETA=0			
Test	Chi-Square	DF	Pr > ChiSq
Likelihood Ratio	164.4873	8	<.0001
Score	187.4930	8	<.0001
Wald	147.1971	8	<.0001

Logistic Regression Results

The LOGISTIC Procedure

Analysis of Maximum Likelihood Estimates					
Parameter	DF	Estimate	Standard Error	Wald Chi-Square	Pr > ChiSq
Intercept	1	-5.8684	0.8512	47.5269	<.0001
C5	1	-0.0572	0.0326	3.0797	0.0793
age	1	0.0138	0.0147	0.8843	0.3470
BMI	1	0.2207	0.0204	117.6214	<.0001
SBP	1	-0.00498	0.00736	0.4568	0.4991
D1_al	1	0.2791	0.1834	2.3170	0.1280
D2_al	1	0.3763	0.2115	3.1661	0.0752
D1_sm	1	0.2274	0.3016	0.5684	0.4509
D2_sm	1	-0.0912	0.3038	0.0902	0.7639

Odds Ratio Estimates			
Effect	Point Estimate	95% Wald Confidence Limits	
C5	0.944	0.886	1.007
age	1.014	0.985	1.044
BMI	1.247	1.198	1.298
SBP	0.995	0.981	1.010
D1_al	1.322	0.923	1.894
D2_al	1.457	0.963	2.205
D1_sm	1.255	0.695	2.267
D2_sm	0.913	0.503	1.656

Association of Predicted Probabilities and Observed Responses			
Percent Concordant	72.4	Somers' D	0.455
Percent Discordant	26.9	Gamma	0.458
Percent Tied	0.6	Tau-a	0.129
Pairs	410310	c	0.727

Logistic Regression Results

The LOGISTIC Procedure

Model Information	
Data Set	WORK.SORTTEMPTABLESORTED
Response Variable	LDL_x
Number of Response Levels	2
Model	binary logit
Optimization Technique	Fisher's scoring

Number of Observations Read	1701
Number of Observations Used	1701

Response Profile		
Ordered Value	LDL_x	Total Frequency
1	0	1384
2	1	317

Probability modeled is LDL_x='1'.

Model Convergence Status
Convergence criterion (GCONV=1E-8) satisfied.

Model Fit Statistics		
Criterion	Intercept Only	Intercept and Covariates
AIC	1638.032	1543.216
SC	1643.471	1592.167
-2 Log L	1636.032	1525.216

Testing Global Null Hypothesis: BETA=0			
Test	Chi-Square	DF	Pr > ChiSq
Likelihood Ratio	110.8161	8	<.0001
Score	120.4838	8	<.0001
Wald	104.4004	8	<.0001

Logistic Regression Results

The LOGISTIC Procedure

Analysis of Maximum Likelihood Estimates					
Parameter	DF	Estimate	Standard Error	Wald Chi-Square	Pr > ChiSq
Intercept	1	-6.7194	0.8266	66.0822	<.0001
C5	1	-0.0180	0.0310	0.3381	0.5610
age	1	0.0853	0.0139	37.6813	<.0001
BMI	1	0.1333	0.0189	49.7854	<.0001
SBP	1	0.00244	0.00703	0.1200	0.7290
D1_al	1	0.0432	0.1818	0.0564	0.8122
D2_al	1	0.1606	0.2043	0.6181	0.4318
D1_sm	1	0.0911	0.2893	0.0991	0.7529
D2_sm	1	0.1806	0.2710	0.4440	0.5052

Odds Ratio Estimates			
Effect	Point Estimate	95% Wald Confidence Limits	
C5	0.982	0.924	1.044
age	1.089	1.060	1.119
BMI	1.143	1.101	1.186
SBP	1.002	0.989	1.016
D1_al	1.044	0.731	1.491
D2_al	1.174	0.787	1.753
D1_sm	1.095	0.621	1.931
D2_sm	1.198	0.704	2.038

Association of Predicted Probabilities and Observed Responses			
Percent Concordant	67.0	Somers' D	0.347
Percent Discordant	32.3	Gamma	0.350
Percent Tied	0.7	Tau-a	0.105
Pairs	438728	c	0.674

Logistic Regression Results

The LOGISTIC Procedure

Model Information	
Data Set	WORK.SORTTEMPTABLESORTED
Response Variable	TG_x
Number of Response Levels	2
Model	binary logit
Optimization Technique	Fisher's scoring

Number of Observations Read	1701
Number of Observations Used	1701

Response Profile		
Ordered Value	TG_x	Total Frequency
1	0	1614
2	1	87

Probability modeled is TG_x='1'.

Model Convergence Status
Convergence criterion (GCONV=1E-8) satisfied.

Model Fit Statistics		
Criterion	Intercept Only	Intercept and Covariates
AIC	688.785	588.441
SC	694.224	637.392
-2 Log L	686.785	570.441

Testing Global Null Hypothesis: BETA=0			
Test	Chi-Square	DF	Pr > ChiSq
Likelihood Ratio	116.3441	8	<.0001
Score	167.4457	8	<.0001
Wald	113.4535	8	<.0001

Logistic Regression Results

The LOGISTIC Procedure

Analysis of Maximum Likelihood Estimates					
Parameter	DF	Estimate	Standard Error	Wald Chi-Square	Pr > ChiSq
Intercept	1	-9.7142	1.3362	52.8567	<.0001
C5	1	-0.0506	0.0563	0.8083	0.3686
age	1	0.0450	0.0240	3.5257	0.0604
BMI	1	0.2475	0.0291	72.5944	<.0001
SBP	1	0.00263	0.0118	0.0499	0.8233
D1_al	1	0.0964	0.3242	0.0884	0.7662
D2_al	1	0.4062	0.3423	1.4084	0.2353
D1_sm	1	-0.2198	0.6193	0.1260	0.7226
D2_sm	1	0.3393	0.4061	0.6980	0.4034

Odds Ratio Estimates			
Effect	Point Estimate	95% Wald Confidence Limits	
C5	0.951	0.851	1.062
age	1.046	0.998	1.096
BMI	1.281	1.210	1.356
SBP	1.003	0.980	1.026
D1_al	1.101	0.583	2.079
D2_al	1.501	0.767	2.936
D1_sm	0.803	0.238	2.702
D2_sm	1.404	0.633	3.112

Association of Predicted Probabilities and Observed Responses			
Percent Concordant	77.5	Somers' D	0.565
Percent Discordant	21.1	Gamma	0.573
Percent Tied	1.4	Tau-a	0.055
Pairs	140418	c	0.782

Logistic Regression Results

The LOGISTIC Procedure

Model Information	
Data Set	WORK.SORTTEMPTABLESORTED
Response Variable	TC_x
Number of Response Levels	2
Model	binary logit
Optimization Technique	Fisher's scoring

Number of Observations Read	1701
Number of Observations Used	1701

Response Profile		
Ordered Value	TC_x	Total Frequency
1	0	1386
2	1	315

Probability modeled is TC_x='1'.

Model Convergence Status
Convergence criterion (GCONV=1E-8) satisfied.

Model Fit Statistics		
Criterion	Intercept Only	Intercept and Covariates
AIC	1632.121	1580.369
SC	1637.560	1629.320
-2 Log L	1630.121	1562.369

Testing Global Null Hypothesis: BETA=0			
Test	Chi-Square	DF	Pr > ChiSq
Likelihood Ratio	67.7520	8	<.0001
Score	72.1516	8	<.0001
Wald	66.6827	8	<.0001

Logistic Regression Results

The LOGISTIC Procedure

Analysis of Maximum Likelihood Estimates					
Parameter	DF	Estimate	Standard Error	Wald Chi-Square	Pr > ChiSq
Intercept	1	-4.1714	0.8266	25.4657	<.0001
C6	1	-0.0523	0.0301	3.0239	0.0820
age	1	0.0617	0.0136	20.6462	<.0001
BMI	1	0.1128	0.0186	36.5943	<.0001
SBP	1	-0.00825	0.00703	1.3783	0.2404
D1_al	1	0.1822	0.1744	1.0924	0.2959
D2_al	1	0.2914	0.1981	2.1632	0.1414
D1_sm	1	0.1454	0.2808	0.2681	0.6046
D2_sm	1	-0.1295	0.2859	0.2054	0.6504

Odds Ratio Estimates			
Effect	Point Estimate	95% Wald Confidence Limits	
C6	0.949	0.895	1.007
age	1.064	1.036	1.092
BMI	1.119	1.079	1.161
SBP	0.992	0.978	1.006
D1_al	1.200	0.853	1.689
D2_al	1.338	0.908	1.973
D1_sm	1.157	0.667	2.005
D2_sm	0.878	0.502	1.538

Association of Predicted Probabilities and Observed Responses			
Percent Concordant	62.4	Somers' D	0.256
Percent Discordant	36.8	Gamma	0.258
Percent Tied	0.9	Tau-a	0.077
Pairs	436590	c	0.628

Logistic Regression Results

The LOGISTIC Procedure

Model Information	
Data Set	WORK.SORTTEMPTABLESORTED
Response Variable	HDL_x
Number of Response Levels	2
Model	binary logit
Optimization Technique	Fisher's scoring

Number of Observations Read	1701
Number of Observations Used	1701

Response Profile		
Ordered Value	HDL_x	Total Frequency
1	0	1410
2	1	291

Probability modeled is HDL_x='1'.

Model Convergence Status
Convergence criterion (GCONV=1E-8) satisfied.

Model Fit Statistics		
Criterion	Intercept Only	Intercept and Covariates
AIC	1558.714	1412.823
SC	1564.153	1461.774
-2 Log L	1556.714	1394.823

Testing Global Null Hypothesis: BETA=0			
Test	Chi-Square	DF	Pr > ChiSq
Likelihood Ratio	161.8914	8	<.0001
Score	184.9149	8	<.0001
Wald	145.1591	8	<.0001

Logistic Regression Results

The LOGISTIC Procedure

Analysis of Maximum Likelihood Estimates					
Parameter	DF	Estimate	Standard Error	Wald Chi-Square	Pr > ChiSq
Intercept	1	-6.5497	0.8816	55.1919	<.0001
C6	1	0.0226	0.0325	0.4827	0.4872
age	1	0.00896	0.0145	0.3817	0.5367
BMI	1	0.2249	0.0203	122.4695	<.0001
SBP	1	-0.00577	0.00734	0.6186	0.4316
D1_al	1	0.2727	0.1831	2.2179	0.1364
D2_al	1	0.3949	0.2108	3.5092	0.0610
D1_sm	1	0.2505	0.3011	0.6921	0.4055
D2_sm	1	-0.0777	0.3053	0.0648	0.7991

Odds Ratio Estimates			
Effect	Point Estimate	95% Wald Confidence Limits	
C6	1.023	0.960	1.090
age	1.009	0.981	1.038
BMI	1.252	1.203	1.303
SBP	0.994	0.980	1.009
D1_al	1.314	0.917	1.881
D2_al	1.484	0.982	2.243
D1_sm	1.285	0.712	2.318
D2_sm	0.925	0.509	1.683

Association of Predicted Probabilities and Observed Responses			
Percent Concordant	72.1	Somers' D	0.448
Percent Discordant	27.3	Gamma	0.451
Percent Tied	0.6	Tau-a	0.127
Pairs	410310	c	0.724

Logistic Regression Results

The LOGISTIC Procedure

Model Information	
Data Set	WORK.SORTTEMPTABLESORTED
Response Variable	LDL_x
Number of Response Levels	2
Model	binary logit
Optimization Technique	Fisher's scoring

Number of Observations Read	1701
Number of Observations Used	1701

Response Profile		
Ordered Value	LDL_x	Total Frequency
1	0	1384
2	1	317

Probability modeled is LDL_x='1'.

Model Convergence Status
Convergence criterion (GCONV=1E-8) satisfied.

Model Fit Statistics		
Criterion	Intercept Only	Intercept and Covariates
AIC	1638.032	1543.321
SC	1643.471	1592.271
-2 Log L	1636.032	1525.321

Testing Global Null Hypothesis: BETA=0			
Test	Chi-Square	DF	Pr > ChiSq
Likelihood Ratio	110.7116	8	<.0001
Score	120.4275	8	<.0001
Wald	104.2570	8	<.0001

Logistic Regression Results

The LOGISTIC Procedure

Analysis of Maximum Likelihood Estimates					
Parameter	DF	Estimate	Standard Error	Wald Chi-Square	Pr > ChiSq
Intercept	1	-6.7140	0.8471	62.8188	<.0001
C6	1	-0.0148	0.0307	0.2336	0.6288
age	1	0.0843	0.0137	37.6411	<.0001
BMI	1	0.1340	0.0188	50.5903	<.0001
SBP	1	0.00226	0.00702	0.1033	0.7479
D1_al	1	0.0455	0.1819	0.0626	0.8024
D2_al	1	0.1608	0.2043	0.6194	0.4313
D1_sm	1	0.0904	0.2893	0.0976	0.7548
D2_sm	1	0.1743	0.2716	0.4118	0.5211

Odds Ratio Estimates			
Effect	Point Estimate	95% Wald Confidence Limits	
C6	0.985	0.928	1.046
age	1.088	1.059	1.118
BMI	1.143	1.102	1.186
SBP	1.002	0.989	1.016
D1_al	1.047	0.733	1.495
D2_al	1.174	0.787	1.753
D1_sm	1.095	0.621	1.930
D2_sm	1.190	0.699	2.027

Association of Predicted Probabilities and Observed Responses			
Percent Concordant	67.0	Somers' D	0.347
Percent Discordant	32.3	Gamma	0.350
Percent Tied	0.7	Tau-a	0.105
Pairs	438728	c	0.674

Logistic Regression Results

The LOGISTIC Procedure

Model Information	
Data Set	WORK.SORTTEMPTABLESORTED
Response Variable	TG_x
Number of Response Levels	2
Model	binary logit
Optimization Technique	Fisher's scoring

Number of Observations Read	1701
Number of Observations Used	1701

Response Profile		
Ordered Value	TG_x	Total Frequency
1	0	1614
2	1	87

Probability modeled is TG_x='1'.

Model Convergence Status
Convergence criterion (GCONV=1E-8) satisfied.

Model Fit Statistics		
Criterion	Intercept Only	Intercept and Covariates
AIC	688.785	588.789
SC	694.224	637.739
-2 Log L	686.785	570.789

Testing Global Null Hypothesis: BETA=0			
Test	Chi-Square	DF	Pr > ChiSq
Likelihood Ratio	115.9969	8	<.0001
Score	167.4426	8	<.0001
Wald	113.2485	8	<.0001

Logistic Regression Results

The LOGISTIC Procedure

Analysis of Maximum Likelihood Estimates					
Parameter	DF	Estimate	Standard Error	Wald Chi-Square	Pr > ChiSq
Intercept	1	-10.4712	1.4079	55.3168	<.0001
C6	1	0.0382	0.0566	0.4568	0.4991
age	1	0.0393	0.0234	2.8243	0.0929
BMI	1	0.2519	0.0290	75.5709	<.0001
SBP	1	0.00180	0.0117	0.0239	0.8772
D1_al	1	0.0898	0.3248	0.0764	0.7823
D2_al	1	0.4412	0.3418	1.6663	0.1968
D1_sm	1	-0.1896	0.6192	0.0938	0.7594
D2_sm	1	0.3495	0.4080	0.7338	0.3916

Odds Ratio Estimates			
Effect	Point Estimate	95% Wald Confidence Limits	
C6	1.039	0.930	1.161
age	1.040	0.993	1.089
BMI	1.286	1.215	1.362
SBP	1.002	0.979	1.025
D1_al	1.094	0.579	2.068
D2_al	1.555	0.796	3.038
D1_sm	0.827	0.246	2.784
D2_sm	1.418	0.638	3.155

Association of Predicted Probabilities and Observed Responses			
Percent Concordant	77.6	Somers' D	0.568
Percent Discordant	20.9	Gamma	0.577
Percent Tied	1.5	Tau-a	0.055
Pairs	140418	c	0.784
