# Supplementary material for: Association between Blood Lipid Levels and Personality Traits in Young Korean Women
Source: PLoS One. 2014 Sep 30;9(9):e108406. doi: 10.1371/journal.pone.0108406 (PMC4182467; doi:10.1371/journal.pone.0108406)
Supplement: T-Test Result S1 — The results of the t-test using SAS program. There are significant differences between the normal (“0”) and abnormal groups (“1”) when applying clinical criteria for the lipid level. (PDF) [file pone.0108406.s005.pdf]

t Test

The TTEST Procedure

Variable: N

TC_x	N	Mean	Std Dev	Std Err	Minimum	Maximum
0	1386	57.6551	9.7558	0.2620	30.0000	88.0000
1	315	57.1651	9.9674	0.5616	33.0000	86.0000
Diff (1-2)		0.4900	9.7952	0.6114		

TC_x	Method	Mean	95% CL Mean		Std Dev	95% CL Std Dev	
0		57.6551	57.1411	58.1692	9.7558	9.4056	10.1332
1		57.1651	56.0601	58.2700	9.9674	9.2451	10.8131
Diff (1-2)	Pooled	0.4900	-0.7091	1.6892	9.7952	9.4767	10.1361
Diff (1-2)	Satterthwaite	0.4900	-0.7278	1.7079			

Method	Variances	DF	t Value	Pr > |t|
Pooled	Equal	1699	0.80	0.4230
Satterthwaite	Unequal	460.67	0.79	0.4295

Equality of Variances				
Method	Num DF	Den DF	F Value	Pr > F
Folded F	314	1385	1.04	0.6128

Variable: E

TC_x	N	Mean	Std Dev	Std Err	Minimum	Maximum
0	1386	60.6833	9.5304	0.2560	28.0000	89.0000
1	315	60.1619	9.6540	0.5439	27.0000	85.0000
Diff (1-2)		0.5214	9.5534	0.5963		

t Test

The TTEST Procedure

Variable: E

TC_x	Method	Mean	95% CL Mean		Std Dev	95% CL Std Dev	
0		60.6833	60.1811	61.1854	9.5304	9.1884	9.8991
1		60.1619	59.0917	61.2321	9.6540	8.9544	10.4731
Diff (1-2)	Pooled	0.5214	-0.6482	1.6909	9.5534	9.2427	9.8858
Diff (1-2)	Satterthwaite	0.5214	-0.6600	1.7027			

Method	Variances	DF	t Value	Pr > |t|
Pooled	Equal	1699	0.87	0.3821
Satterthwaite	Unequal	463.35	0.87	0.3863

Equality of Variances				
Method	Num DF	Den DF	F Value	Pr > F
Folded F	314	1385	1.03	0.7560

Variable: O

TC_x	N	Mean	Std Dev	Std Err	Minimum	Maximum
0	1386	64.1255	7.9213	0.2128	30.0000	88.0000
1	315	63.6063	7.7770	0.4382	43.0000	83.0000
Diff (1-2)		0.5192	7.8948	0.4928		

t Test

The TTEST Procedure

Variable: O

TC_x	Method	Mean	95% CL Mean		Std Dev	95% CL Std Dev	
0		64.1255	63.7082	64.5429	7.9213	7.6370	8.2277
1		63.6063	62.7442	64.4685	7.7770	7.2134	8.4368
Diff (1-2)	Pooled	0.5192	-0.4473	1.4857	7.8948	7.6381	8.1695
Diff (1-2)	Satterthwaite	0.5192	-0.4380	1.4764			

Method	Variances	DF	t Value	Pr > |t|
Pooled	Equal	1699	1.05	0.2922
Satterthwaite	Unequal	473.56	1.07	0.2870

Equality of Variances				
Method	Num DF	Den DF	F Value	Pr > F
Folded F	1385	314	1.04	0.6924

Variable: A

TC_x	N	Mean	Std Dev	Std Err	Minimum	Maximum
0	1386	59.6789	7.4664	0.2006	30.0000	82.0000
1	315	59.3460	7.7844	0.4386	31.0000	81.0000
Diff (1-2)		0.3329	7.5262	0.4698		

t Test

The TTEST Procedure

Variable: A

TC_x	Method	Mean	95% CL Mean		Std Dev	95% CL Std Dev	
0		59.6789	59.2855	60.0724	7.4664	7.1984	7.7553
1		59.3460	58.4831	60.2090	7.7844	7.2203	8.4449
Diff (1-2)	Pooled	0.3329	-0.5885	1.2543	7.5262	7.2814	7.7881
Diff (1-2)	Satterthwaite	0.3329	-0.6149	1.2807			

Method	Variances	DF	t Value	Pr > |t|
Pooled	Equal	1699	0.71	0.4786
Satterthwaite	Unequal	454.53	0.69	0.4904

Equality of Variances				
Method	Num DF	Den DF	F Value	Pr > F
Folded F	314	1385	1.09	0.3320

Variable: C

TC_x	N	Mean	Std Dev	Std Err	Minimum	Maximum
0	1386	61.1248	8.3552	0.2244	37.0000	85.0000
1	315	59.9810	8.0516	0.4537	27.0000	80.0000
Diff (1-2)		1.1439	8.2999	0.5181		

t Test

The TTEST Procedure

Variable: C

TC_x	Method	Mean	95% CL Mean		Std Dev	95% CL Std Dev	
0		61.1248	60.6846	61.5651	8.3552	8.0553	8.6784
1		59.9810	59.0884	60.8735	8.0516	7.4681	8.7347
Diff (1-2)	Pooled	1.1439	0.1277	2.1600	8.2999	8.0300	8.5887
Diff (1-2)	Satterthwaite	1.1439	0.1494	2.1384			

Method	Variances	DF	t Value	Pr > |t|
Pooled	Equal	1699	2.21	0.0274
Satterthwaite	Unequal	479.98	2.26	0.0243

Equality of Variances				
Method	Num DF	Den DF	F Value	Pr > F
Folded F	1385	314	1.08	0.4167

t Test

The TTEST Procedure

Variable: N

HDL_x	N	Mean	Std Dev	Std Err	Minimum	Maximum
0	1410	57.3404	9.7687	0.2602	30.0000	87.0000
1	291	58.6495	9.8618	0.5781	34.0000	88.0000
Diff (1-2)		-1.3091	9.7846	0.6300		

HDL_x	Method	Mean	95% CL Mean		Std Dev	95% CL Std Dev	
0		57.3404	56.8301	57.8508	9.7687	9.4210	10.1432
1		58.6495	57.5117	59.7873	9.8618	9.1204	10.7355
Diff (1-2)	Pooled	-1.3091	-2.5447	-0.0734	9.7846	9.4665	10.1251
Diff (1-2)	Satterthwaite	-1.3091	-2.5552	-0.0629			

Method	Variances	DF	t Value	Pr > |t|
Pooled	Equal	1699	-2.08	0.0379
Satterthwaite	Unequal	415.83	-2.06	0.0395

Equality of Variances				
Method	Num DF	Den DF	F Value	Pr > F
Folded F	290	1409	1.02	0.8192

Variable: E

HDL_x	N	Mean	Std Dev	Std Err	Minimum	Maximum
0	1410	60.6546	9.5463	0.2542	27.0000	89.0000
1	291	60.2577	9.5934	0.5624	33.0000	84.0000
Diff (1-2)		0.3969	9.5544	0.6152		

t Test

The TTEST Procedure

Variable: E

HDL_x	Method	Mean	95% CL Mean		Std Dev	95% CL Std Dev	
0		60.6546	60.1559	61.1533	9.5463	9.2065	9.9123
1		60.2577	59.1509	61.3646	9.5934	8.8721	10.4433
Diff (1-2)	Pooled	0.3969	-0.8097	1.6035	9.5544	9.2437	9.8868
Diff (1-2)	Satterthwaite	0.3969	-0.8163	1.6100			

Method	Variances	DF	t Value	Pr > |t|
Pooled	Equal	1699	0.65	0.5189
Satterthwaite	Unequal	417.06	0.64	0.5205

Equality of Variances				
Method	Num DF	Den DF	F Value	Pr > F
Folded F	290	1409	1.01	0.8981

Variable: O

HDL_x	N	Mean	Std Dev	Std Err	Minimum	Maximum
0	1410	64.1589	7.8839	0.2100	30.0000	88.0000
1	291	63.4021	7.9325	0.4650	41.0000	85.0000
Diff (1-2)		0.7568	7.8922	0.5082		

t Test

The TTEST Procedure

Variable: O

HDL_x	Method	Mean	95% CL Mean		Std Dev	95% CL Std Dev	
0		64.1589	63.7470	64.5707	7.8839	7.6033	8.1862
1		63.4021	62.4868	64.3173	7.9325	7.3361	8.6352
Diff (1-2)	Pooled	0.7568	-0.2399	1.7535	7.8922	7.6356	8.1669
Diff (1-2)	Satterthwaite	0.7568	-0.2461	1.7597			

Method	Variances	DF	t Value	Pr > |t|
Pooled	Equal	1699	1.49	0.1366
Satterthwaite	Unequal	416.73	1.48	0.1387

Equality of Variances				
Method	Num DF	Den DF	F Value	Pr > F
Folded F	290	1409	1.01	0.8770

Variable: A

HDL_x	N	Mean	Std Dev	Std Err	Minimum	Maximum
0	1410	59.7589	7.5862	0.2020	30.0000	82.0000
1	291	58.9313	7.1950	0.4218	38.0000	78.0000
Diff (1-2)		0.8276	7.5208	0.4842		

t Test

The TTEST Procedure

Variable: A

HDL_x	Method	Mean	95% CL Mean		Std Dev	95% CL Std Dev	
0		59.7589	59.3626	60.1552	7.5862	7.3162	7.8770
1		58.9313	58.1011	59.7614	7.1950	6.6540	7.8324
Diff (1-2)	Pooled	0.8276	-0.1222	1.7774	7.5208	7.2763	7.7825
Diff (1-2)	Satterthwaite	0.8276	-0.0916	1.7468			

Method	Variances	DF	t Value	Pr > |t|
Pooled	Equal	1699	1.71	0.0876
Satterthwaite	Unequal	433.64	1.77	0.0775

Equality of Variances				
Method	Num DF	Den DF	F Value	Pr > F
Folded F	1409	290	1.11	0.2597

Variable: C

HDL_x	N	Mean	Std Dev	Std Err	Minimum	Maximum
0	1410	61.0596	8.1965	0.2183	27.0000	85.0000
1	291	60.2027	8.8159	0.5168	37.0000	84.0000
Diff (1-2)		0.8568	8.3055	0.5348		

t Test

The TTEST Procedure

Variable:
C

HDL_x	Method	Mean	95% CL Mean		Std Dev	95% CL Std Dev	
0		61.0596	60.6314	61.4878	8.1965	7.9048	8.5108
1		60.2027	59.1856	61.2199	8.8159	8.1531	9.5969
Diff (1-2)	Pooled	0.8568	-0.1920	1.9057	8.3055	8.0354	8.5945
Diff (1-2)	Satterthwaite	0.8568	-0.2461	1.9597			

Method	Variances	DF	t Value	Pr > |t|
Pooled	Equal	1699	1.60	0.1093
Satterthwaite	Unequal	400.08	1.53	0.1275

Equality of Variances				
Method	Num DF	Den DF	F Value	Pr > F
Folded F	290	1409	1.16	0.1005

t Test

The TTEST Procedure

Variable: N

LDL_x	N	Mean	Std Dev	Std Err	Minimum	Maximum
0	1384	57.6055	9.8018	0.2635	30.0000	88.0000
1	317	57.3849	9.7742	0.5490	33.0000	86.0000
Diff (1-2)		0.2206	9.7967	0.6100		

LDL_x	Method	Mean	95% CL Mean		Std Dev	95% CL Std Dev	
0		57.6055	57.0886	58.1223	9.8018	9.4498	10.1813
1		57.3849	56.3048	58.4650	9.7742	9.0680	10.6007
Diff (1-2)	Pooled	0.2206	-0.9758	1.4171	9.7967	9.4781	10.1376
Diff (1-2)	Satterthwaite	0.2206	-0.9759	1.4172			

Method	Variances	DF	t Value	Pr > |t|
Pooled	Equal	1699	0.36	0.7176
Satterthwaite	Unequal	472.61	0.36	0.7173

Equality of Variances				
Method	Num DF	Den DF	F Value	Pr > F
Folded F	1383	316	1.01	0.9637

Variable: E

LDL_x	N	Mean	Std Dev	Std Err	Minimum	Maximum
0	1384	60.7312	9.5777	0.2575	27.0000	89.0000
1	317	59.9558	9.4319	0.5297	33.0000	86.0000
Diff (1-2)		0.7754	9.5508	0.5947		

t Test

The TTEST Procedure

Variable: E

LDL_x	Method	Mean	95% CL Mean		Std Dev	95% CL Std Dev	
0		60.7312	60.2262	61.2362	9.5777	9.2337	9.9485
1		59.9558	58.9136	60.9981	9.4319	8.7504	10.2294
Diff (1-2)	Pooled	0.7754	-0.3910	1.9418	9.5508	9.2402	9.8831
Diff (1-2)	Satterthwaite	0.7754	-0.3820	1.9327			

Method	Variances	DF	t Value	Pr > |t|
Pooled	Equal	1699	1.30	0.1925
Satterthwaite	Unequal	476.82	1.32	0.1887

Equality of Variances				
Method	Num DF	Den DF	F Value	Pr > F
Folded F	1383	316	1.03	0.7427

Variable: O

LDL_x	N	Mean	Std Dev	Std Err	Minimum	Maximum
0	1384	64.1207	7.8463	0.2109	36.0000	88.0000
1	317	63.6309	8.1053	0.4552	30.0000	88.0000
Diff (1-2)		0.4897	7.8951	0.4916		

t Test

The TTEST Procedure

Variable: O

LDL_x	Method	Mean	95% CL Mean		Std Dev	95% CL Std Dev	
0		64.1207	63.7069	64.5344	7.8463	7.5645	8.1500
1		63.6309	62.7352	64.5266	8.1053	7.5196	8.7906
Diff (1-2)	Pooled	0.4897	-0.4745	1.4540	7.8951	7.6383	8.1698
Diff (1-2)	Satterthwaite	0.4897	-0.4962	1.4757			

Method	Variances	DF	t Value	Pr > |t|
Pooled	Equal	1699	1.00	0.3193
Satterthwaite	Unequal	461.35	0.98	0.3295

Equality of Variances				
Method	Num DF	Den DF	F Value	Pr > F
Folded F	316	1383	1.07	0.4473

Variable: A

LDL_x	N	Mean	Std Dev	Std Err	Minimum	Maximum
0	1384	59.6481	7.4622	0.2006	30.0000	82.0000
1	317	59.4826	7.8044	0.4383	31.0000	81.0000
Diff (1-2)		0.1655	7.5270	0.4687		

t Test

The TTEST Procedure

Variable: A

LDL_x	Method	Mean	95% CL Mean		Std Dev	95% CL Std Dev	
0		59.6481	59.2546	60.0416	7.4622	7.1942	7.7511
1		59.4826	58.6202	60.3451	7.8044	7.2405	8.4643
Diff (1-2)	Pooled	0.1655	-0.7538	1.0847	7.5270	7.2823	7.7890
Diff (1-2)	Satterthwaite	0.1655	-0.7818	1.1128			

Method	Variances	DF	t Value	Pr > |t|
Pooled	Equal	1699	0.35	0.7241
Satterthwaite	Unequal	457.61	0.34	0.7316

Equality of Variances				
Method	Num DF	Den DF	F Value	Pr > F
Folded F	316	1383	1.09	0.2962

Variable: C

LDL_x	N	Mean	Std Dev	Std Err	Minimum	Maximum
0	1384	61.0210	8.2903	0.2228	37.0000	85.0000
1	317	60.4416	8.3889	0.4712	27.0000	80.0000
Diff (1-2)		0.5793	8.3087	0.5174		

t Test

The TTEST Procedure

Variable: C

LDL_x	Method	Mean	95% CL Mean		Std Dev	95% CL Std Dev	
0		61.0210	60.5838	61.4581	8.2903	7.9926	8.6113
1		60.4416	59.5146	61.3687	8.3889	7.7828	9.0982
Diff (1-2)	Pooled	0.5793	-0.4354	1.5940	8.3087	8.0385	8.5979
Diff (1-2)	Satterthwaite	0.5793	-0.4449	1.6035			

Method	Variances	DF	t Value	Pr > |t|
Pooled	Equal	1699	1.12	0.2630
Satterthwaite	Unequal	467.84	1.11	0.2669

Equality of Variances				
Method	Num DF	Den DF	F Value	Pr > F
Folded F	316	1383	1.02	0.7739

t Test

The TTEST Procedure

Variable: N

TG_x	N	Mean	Std Dev	Std Err	Minimum	Maximum
0	1614	57.4926	9.7424	0.2425	30.0000	88.0000
1	87	58.8966	10.6836	1.1454	36.0000	82.0000
Diff (1-2)		-1.4040	9.7922	1.0778		

TG_x	Method	Mean	95% CL Mean		Std Dev	95% CL Std Dev	
0		57.4926	57.0169	57.9682	9.7424	9.4175	10.0906
1		58.8966	56.6196	61.1735	10.6836	9.2980	12.5585
Diff (1-2)	Pooled	-1.4040	-3.5179	0.7099	9.7922	9.4737	10.1329
Diff (1-2)	Satterthwaite	-1.4040	-3.7287	0.9207			

Method	Variances	DF	t Value	Pr > |t|
Pooled	Equal	1699	-1.30	0.1929
Satterthwaite	Unequal	93.872	-1.20	0.2335

Equality of Variances				
Method	Num DF	Den DF	F Value	Pr > F
Folded F	86	1613	1.20	0.2085

Variable: E

TG_x	N	Mean	Std Dev	Std Err	Minimum	Maximum
0	1614	60.6375	9.4609	0.2355	27.0000	89.0000
1	87	59.6437	11.1407	1.1944	33.0000	81.0000
Diff (1-2)		0.9939	9.5530	1.0514		

t Test

The TTEST Procedure

Variable:
E

TG_x	Method	Mean	95% CL Mean		Std Dev	95% CL Std Dev	
0		60.6375	60.1756	61.0995	9.4609	9.1454	9.7991
1		59.6437	57.2693	62.0181	11.1407	9.6957	13.0958
Diff (1-2)	Pooled	0.9939	-1.0684	3.0561	9.5530	9.2424	9.8854
Diff (1-2)	Satterthwaite	0.9939	-1.4237	3.4115			

Method	Variances	DF	t Value	Pr > |t|
Pooled	Equal	1699	0.95	0.3447
Satterthwaite	Unequal	92.809	0.82	0.4164

Equality of Variances				
Method	Num DF	Den DF	F Value	Pr > F
Folded F	86	1613	1.39	0.0251

Variable:
O

TG_x	N	Mean	Std Dev	Std Err	Minimum	Maximum
0	1614	64.1035	7.8783	0.1961	30.0000	88.0000
1	87	62.6552	8.1238	0.8710	41.0000	83.0000
Diff (1-2)		1.4483	7.8909	0.8685		

t Test

The TTEST Procedure

Variable: O

TG_x	Method	Mean	95% CL Mean		Std Dev	95% CL Std Dev	
0		64.1035	63.7188	64.4881	7.8783	7.6156	8.1599
1		62.6552	60.9238	64.3866	8.1238	7.0701	9.5494
Diff (1-2)	Pooled	1.4483	-0.2551	3.1517	7.8909	7.6343	8.1655
Diff (1-2)	Satterthwaite	1.4483	-0.3241	3.2207			

Method	Variances	DF	t Value	Pr > |t|
Pooled	Equal	1699	1.67	0.0956
Satterthwaite	Unequal	94.928	1.62	0.1081

Equality of Variances				
Method	Num DF	Den DF	F Value	Pr > F
Folded F	86	1613	1.06	0.6583

Variable: A

TG_x	N	Mean	Std Dev	Std Err	Minimum	Maximum
0	1614	59.6258	7.5426	0.1877	30.0000	82.0000
1	87	59.4598	7.2333	0.7755	40.0000	76.0000
Diff (1-2)		0.1660	7.5272	0.8285		

t Test

The TTEST Procedure

Variable: A

TG_x	Method	Mean	95% CL Mean		Std Dev	95% CL Std Dev	
0		59.6258	59.2575	59.9940	7.5426	7.2910	7.8122
1		59.4598	57.9181	61.0014	7.2333	6.2951	8.5027
Diff (1-2)	Pooled	0.1660	-1.4589	1.7909	7.5272	7.2824	7.7891
Diff (1-2)	Satterthwaite	0.1660	-1.4177	1.7497			

Method	Variances	DF	t Value	Pr > |t|
Pooled	Equal	1699	0.20	0.8412
Satterthwaite	Unequal	96.359	0.21	0.8356

Equality of Variances				
Method	Num DF	Den DF	F Value	Pr > F
Folded F	1613	86	1.09	0.6306

Variable: C

TG_x	N	Mean	Std Dev	Std Err	Minimum	Maximum
0	1614	60.9727	8.3235	0.2072	27.0000	85.0000
1	87	59.8046	8.0070	0.8584	42.0000	77.0000
Diff (1-2)		1.1681	8.3078	0.9144		

t Test

The TTEST Procedure

Variable: C

TG_x	Method	Mean	95% CL Mean		Std Dev	95% CL Std Dev	
0		60.9727	60.5664	61.3791	8.3235	8.0460	8.6211
1		59.8046	58.0981	61.5111	8.0070	6.9685	9.4122
Diff (1-2)	Pooled	1.1681	-0.6253	2.9616	8.3078	8.0376	8.5969
Diff (1-2)	Satterthwaite	1.1681	-0.5847	2.9210			

Method	Variances	DF	t Value	Pr > |t|
Pooled	Equal	1699	1.28	0.2016
Satterthwaite	Unequal	96.293	1.32	0.1890

Equality of Variances				
Method	Num DF	Den DF	F Value	Pr > F
Folded F	1613	86	1.08	0.6582
